# A Polymorphism in the Epstein-Barr Virus EBER2 Noncoding RNA Drives *In Vivo* Expansion of Latently Infected B Cells

**DOI:** 10.1128/mbio.00836-22

**Published:** 2022-06-01

**Authors:** Yiping Wang, Nathan Ungerleider, Brett A. Hoffman, Mehmet Kara, Paul J. Farrell, Erik K. Flemington, Nara Lee, Scott A. Tibbetts

**Affiliations:** a Department of Molecular Genetics and Microbiology, UF Health Cancer Center, UF Genetics Institute, College of Medicine, University of Florida, Gainesville, Florida, USA; b Department of Pathology, Tulane University School of Medicine, Tulane Cancer Center, New Orleans, Louisiana, USA; c Department of Infectious Disease, Imperial College London, London, United Kingdom; d Department of Microbiology and Molecular Genetics, University of Pittsburgh School of Medicine, Pittsburgh, Pennsylvania, USA; Dartmouth College

**Keywords:** EBV, Epstein-Barr virus, EBER, MHV68, noncoding RNA, B lymphocytes, gammaherpesviruses, latency, lymphoma

## Abstract

The oncogenic gammaherpesviruses, including human Epstein-Barr virus (EBV), human Kaposi’s sarcoma-associated herpesvirus (KSHV), and murine gammaherpesvirus 68 (MHV68, γHV68, MuHV-4), are associated with numerous malignancies, including B cell lymphomas and nasopharyngeal carcinoma. These viruses employ numerous molecular strategies to colonize the host, including the expression of noncoding RNAs (ncRNAs). As the first viral ncRNAs identified, EBV-encoded RNA 1 and 2 (EBER1 and EBER2, respectively) have been investigated extensively for decades; however, their specific *in vivo* functions remain largely unknown. In work here, we used chimeric MHV68 viruses in an *in vivo* complementation system to test whether EBV EBER2 contributes to acute and/or chronic phases of infection. Expression of EBER2 derived from EBV strain B95-8 resulted in a significant expansion of latently infected B cells *in vivo*, which was accompanied by a decrease in virus-infected plasma cells. EBV strains typically carry one of two variants of EBER2, which differ primarily by a 5-nucleotide core polymorphism identified initially in the EBV strain M81. Strikingly, mutation of the 5 nucleotides that define this core polymorphism resulted in the loss of the infected B cell expansion and restored plasma cell infection. This work reveals that the B95-8 variant of EBER2 promotes the expansion of the latently infected B cell pool *in vivo* and may do so in part through inhibition of terminal differentiation. These findings provide new insight into mechanisms by which viral ncRNAs promote *in vivo* colonization and further and provide further evidence of the inherent tumorigenic risks associated with gammaherpesvirus manipulation of B cell differentiation.

## OBSERVATION

**FIG 1:**
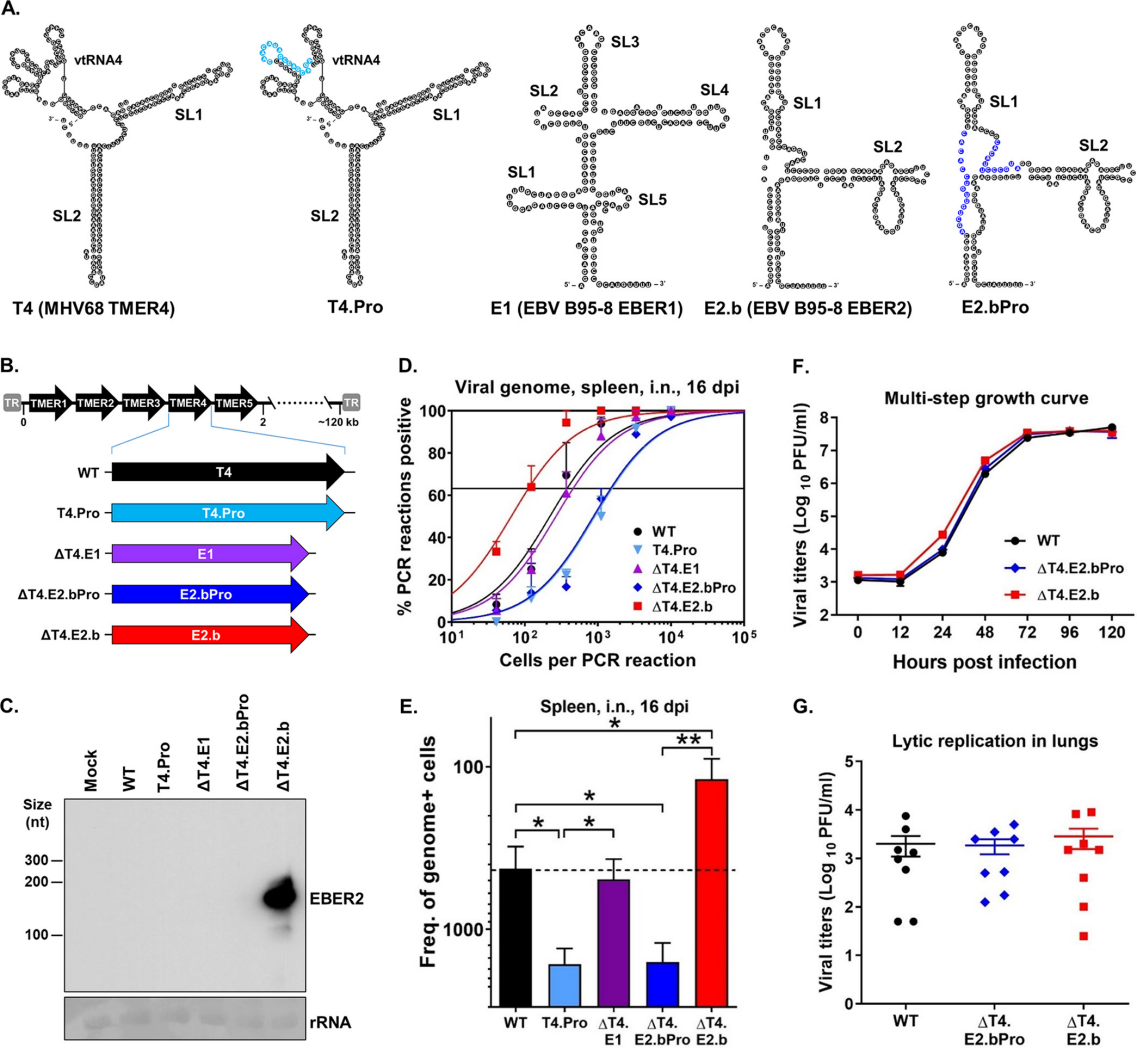
EBV B95-8 EBER2 rescued the *in vivo* attenuation of TMER4-deficient MHV68 and promoted an increased frequency of latently infected B cells. (A) RNA structure predictions for MHV68 TMER4 (8), TMER4 promoter mutant (T4.Pro) (8), EBV B95-8 EBER1 (E1) and EBER2 (E2.b) (2), and E2.b promoter mutant (E2.bPro). The sequence highlighted in light blue in T4.Pro is 5′-CAACAUAGCCAGCAGA-3′. The sequences highlighted in dark blue in E2.bPro are 5′-AGCUGUUGUCCACAC-3′ and 5′-CACAUUCCCGUA-3′. (B) Schematic diagram of MHV68 TMER4 promoter mutant or recombinant viruses carrying EBV E1, E2.b, or E2.bPro in place of TMER4. (C) Northern blot on RNA from mock- or virus-infected fibroblasts. Methylene blue staining of 18S rRNA is shown as a loading control. (D, E) Limiting dilution nested PCR (LDPCR) was performed to determine frequencies of latently infected splenocytes in mice i.n. infected with the indicated viruses at 16 days postinfection (dpi). Three mice were used per group per experiment. The values represent the means ± SEM of three independent experiments. (F) Viral titers during multistep lytic replication in fibroblasts *in vitro*. The values represent means ± SEM. Each growth curve was performed in duplicate in each of two independent experiments. (G) Viral titers during acute lytic replication in lungs from mice at 4 dpi. Each symbol represents an individual mouse. The values represent means ± SEM of a single experiment. *, *P* < 0.05; **, *P* < 0.01; determined by Student’s *t* test.

The oncogenic gammaherpesviruses, including human Epstein-Barr virus (EBV), human Kaposi’s sarcoma-associated herpesvirus (KSHV), and murine gammaherpesvirus 68 (MHV68, γHV68, MuHV-4), lead directly to the development of multiple types of malignancies, including B cell lymphomas (1). A central characteristic of gammaherpesviruses is the ability to establish lifelong latency in B cells, which is critical for tumorigenesis (1). However, the precise underlying mechanisms by which these viruses establish latency *in vivo* remain poorly understood. Gammaherpesviruses employ multiple means to promote latency establishment in B cells, including the expression of noncoding RNAs (ncRNAs) (1, 2). Notably, although the EBV-encoded RNAs 1 and 2 (EBER1 and EBER2) are among the first viral ncRNAs ever identified (3, 4), their specific *in vivo* functions remain largely unknown (2, 5). MHV68 encodes eight small ncRNAs termed tRNA-miRNA-encoded RNAs (TMERs) (6, 7), which share similarity to the EBV EBERs in terms of length, secondary structure, polymerase III usage, and expression profiles (8). Each 200- to 250-nucleotide (nt) TMER is comprised of a tRNA-like element (vtRNA) that harbors a polymerase III promoter, followed by one or two downstream pre-miRNA stem-loops (SL1 and SL2) (Fig. 1A) (6, 9). Both the EBV EBERs and the MHV68 TMERs are expressed extensively *in vivo* in latently infected B cells and tumor cells (2, 5, 6, 10), strongly suggesting that they contribute to chronic infection and pathogenesis. Consistent with this idea, we have demonstrated previously that TMER4 and EBER1 share a conserved *in vivo* function in promoting hematogenous dissemination of infected B cells to peripheral sites of latency (8, 11).

Here, we used a similar ncRNA complementation approach in the MHV68 *in vivo* system to determine whether EBER2 contributes to the acute and/or latent phases of infection. To first test the ability of EBER2 to promote hematogenous dissemination *in vivo* (11), we generated a recombinant virus in which TMER4 was fully replaced by a EBER2 sequence derived from the prototype EBV strain B95-8 (E2.b) (Fig. 1A and B) (12). In parallel, we generated a control virus carrying E2.b with mutations in the polymerase III promoter (E2.bPro). A Northern blot confirmed EBER2 expression following infection with E2.b virus but not E2.bPro virus (Fig. 1C). We then infected wild-type (WT) C57BL/6J mice intranasally (i.n.) with mutant and control viruses and determined their ability to establish latent infection in the spleen (Fig. 1D and E) (7, 13). Consistent with our previous reports (8, 11), the frequency of splenocytes harboring the viral genome was significantly decreased in mice infected with TMER4-defient virus (WT: 1 in 380; T4.Pro: 1 in 1,600) but restored to a frequency nearly equivalent to WT virus in mice infected with virus expressing EBER1 in place of TMER4 (ΔT4.E1: 1 in 460). As expected, the TMER4-deficient virus carrying E2.b with a promoter mutation was attenuated to a level (ΔT4.E2.bPro: 1 in 1,500) nearly equivalent to the TMER4 promoter mutant. Intriguingly though, the expression of native E2.b in place of TMER4 not only fully restored the dissemination defect but also induced a significant increase in the frequency of latently infected cells in the spleen (ΔT4.E2.b, 1 in 110) compared with the WT virus. Notably, increased numbers of latently infected cells were not a result of altered acute infection, as insertion of E2.b had no effect on lytic virus replication *in vitro* or *in vivo* (Fig. 1F and G). Together, these results demonstrated that EBER2 derived from B95-8 promotes latency establishment equivalently to TMER4 and EBER1 but, in addition, significantly enhances latent infection at peripheral sites compared with TMER4 or EBER1.

The 172-nt sequence of EBER2 is generally well-conserved among EBV strains recovered from B cell lymphomas and lymphoma-derived B cell lines. However, it was reported recently that some EBV strains, including strain M81, which displays differential association with nasopharyngeal carcinoma (14, 15), carry EBER2 single nucleotide polymorphisms (SNPs) that may contribute to the virus life cycle (16–19). Included among the M81 EBER2 SNPs are nucleotides at positions 44, 46, 57, 61, 93, 168, and 170 that are present in M81 and many EBV strains from China but differ from the nucleotides that are conserved among most EBV strains from other parts of the world (14, 15), including the B95-8 EBER2 expressed by the ΔT4.E2.b virus. Notably, 5 of these SNPs appear to be linked; in an alignment of the 1,144 EBV genome sequences in GenBank carrying full-length EBER2 sequences (see Fig. S1 and Text S1 in the supplemental material), 31.7% of the sequences carried all 5 of SNPs at positions 44, 46, 57, 61, and 93, whereas the SNPs at positions 168 and 170 were variable among strains, accounting for a variant frequency of 75.4% and 14.6%, respectively (see Table S1 in the supplemental material).

10.1128/mbio.00836-22.1 FIG S1 Alignment of EBER2 sequences from EBV genomes. Full-length EBER2 sequences from all GenBank-deposited EBV genomes were aligned with Geneious using the B95-8 sequence (accession V01555) as the reference. Download FIG S1, PDF file, 10.5 MB. Copyright © 2022 Wang et al. 2022 Wang et al. https://creativecommons.org/licenses/by/4.0/ This content is distributed under the terms of the Creative Commons Attribution 4.0 International license.

10.1128/mbio.00836-22.4 TABLE S1 Alignment of EBER2 sequences from EBV genomes. EBER2 SNPs (frequency, >0.1) were determined by Geneious following alignment of 1,144 EBV genomes. Download Table S1, PDF file, 0.1 MB. Copyright © 2022 Wang et al. 2022 Wang et al. https://creativecommons.org/licenses/by/4.0/ This content is distributed under the terms of the Creative Commons Attribution 4.0 International license.

10.1128/mbio.00836-22.5 TEXT S1 Alignment of EBER2 sequences from EBV genomes. FASTA file. Full-length EBER2 sequences from all GenBank-deposited EBV genomes were aligned with Geneious using the B95-8 sequence (accession V01555) as the reference. Download Text S1, DOCX file, 0.04 MB. Copyright © 2022 Wang et al. 2022 Wang et al. https://creativecommons.org/licenses/by/4.0/ This content is distributed under the terms of the Creative Commons Attribution 4.0 International license.

**FIG 2:**
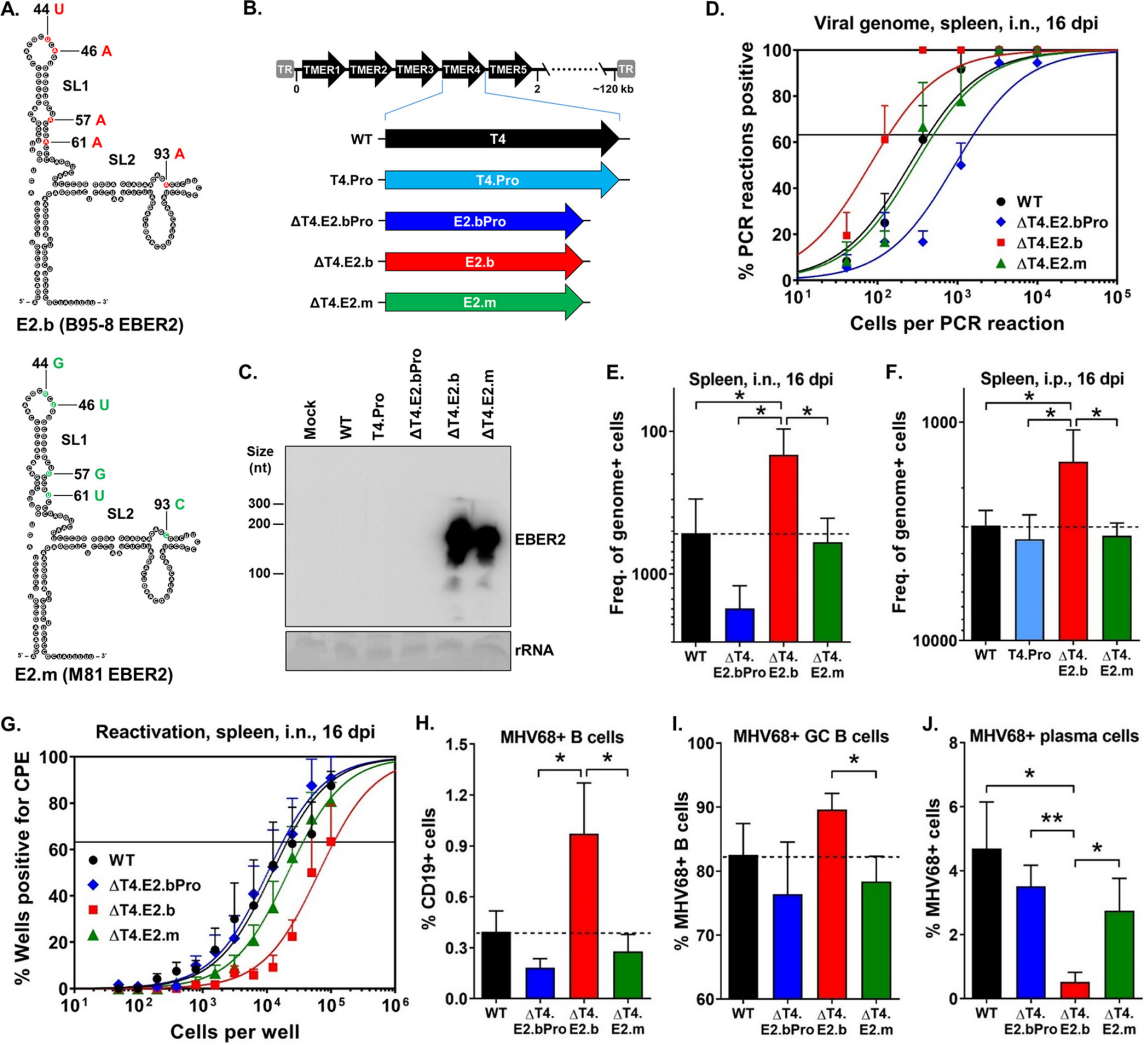
The EBV B95-8 EBER2 polymorphism promoted an increased frequency of latently infected B cells and an impairment of plasma cell infection *in vivo*. (A) RNA structure predictions for EBV B95-8 EBER2 (E2.b) and M81 EBER2 (E2.m) (2). (B) Schematic diagram of MHV68 TMER4 promoter mutant (T4.Pro) or recombinant viruses carrying EBV E2.b, E2.b promoter mutant (E2.bPro), or E2.m in place of TMER4. (C) Northern blot on RNA from mock- or virus-infected fibroblasts. Methylene blue staining of 18S rRNA is shown as a loading control. (D and E) LDPCR was performed on splenocytes exactly as described for Fig. 1D and E. Three mice were used per group per experiment. The values represent the means ± SEM of three independent experiments. (F) LDPCR was performed on splenocytes as described for Fig. 1D and E except that the viruses were i.p. inoculated. Three mice were used per group per experiment. The values represent the means ± SEM of three independent experiments. (G) A limiting dilution *ex vivo* reactivation assay was performed to determine the frequencies of splenocytes reactivating from latency in mice at 16 dpi. Three mice were used per group per experiment. The values represent the means ± SEM of five independent experiments. (H–J) The percentages of virus-positive B cells, GC B cells, and plasma cells in mice i.n. infected with the indicated viruses were analyzed by flow cytometry at 16 dpi. Three mice were used per group per experiment. The values represent the means ± SEM of three to five independent experiments. (H) Percent virus-positive B cells (CCF4-AM^+^ CD19^+^). (I) Percent virus-positive GC B cells (CCF4-AM^+^ CD19^+^ GL7^+^). (J) Percent virus-positive plasma cells (CCF4-AM^+^ CD19^low/−^ CD138^+^). *, *P* < 0.05; **, *P* < 0.01; determined by Student’s *t* test.

To determine whether the 5 nts present at positions 44, 46, 57, 61, and 93 in the B95-8 polymorphism contributed to EBER2 function in latency, we generated a version of the ΔT4.E2.b virus in which those 5 bases were mutated to match the M81 EBER2 polymorphism (ΔT4.E2.m) (Fig. 2A and B). A northern blot confirmed EBER2 expression during ΔT4.E2.m virus infection (Fig. 2C). We then determined the ability of the ΔT4.E2.m virus to establish splenic latency in mice following i.n. inoculation (Fig. 2D and E). While E2.m expression in place of TMER4 fully restored the dissemination defect of TMER4-deficient virus (WT: 1 in 430; ΔT4.E2.bPro: 1 in 1,600; ΔT4.E2.m: 1 in 500), it failed to promote the enhanced latency observed with E2.b (ΔT4.E2.b: 1 in 140). The ability of E2.b to promote splenic latency was not due to enhanced dissemination from mucosal sites because a similar increase in splenic latency was observed even when the ΔT4.E2.b virus was administered by intraperitoneal (i.p.) inoculation (Fig. 2F). Moreover, the increased number of latently infected cells present in ΔT4.E2.b-infected mice was not a result of increased reactivation, as cells carrying E2.b demonstrated decreased reactivation (WT: 1 in 21,000; ΔT4.E2.b: 1 in 112,000) (Fig. 2G). Thus, these findings demonstrated that EBER2 promotes increased *in vivo* latency through a mechanism dependent upon the 5-nt polymorphism present in EBV strain B95-8.

Gammaherpesviruses deploy multiple molecular strategies to modulate the balance between latency and reactivation and thereby establish lifelong infection in B cells (1, 5). For example, terminal differentiation of infected B cells to plasma cells results in reactivation of EBV, KSHV, and MHV68 (20–22). Because B cells are a primary reservoir for both EBV and MHV68 latency (1, 5) and plasma cells are a major source of reactivating virus in MHV68-infected mice (23), we determined whether B95-8 EBER2 promotes B cell latency through inhibition of plasma cell differentiation. To do so, we used multiparametric flow cytometry to analyze total and virus-positive B cells, germinal center (GC) B cells, and plasma cells in infected mice at 16 days postinfection (dpi) (Fig. 2H to J, see Fig. S2 and S3 in the supplemental material). We observed that the virus expressing E2.b in place of TMER4 had no significant impact on the numbers of total B cells, GC B cells, and plasma cells (Fig. S3), compared with the WT virus and virus expressing E2.m in place of TMER4. In contrast and as expected, the percentage of B cells infected with E2.b virus was significantly increased (Fig. 2H), compared with WT virus and E2.m virus. Consistent with the critical role of GC B cells as a major latency reservoir during latency establishment (24–26), this striking increase in B cell infection with the virus expressing E2.b correlated with a significantly increased frequency of latently infected GC B cells (Fig. 2I). Notably though, despite an overall increase in the number of B cells infected with E2.b virus, the percentage of plasma cells infected with E2.b virus was significantly reduced compared with that of control samples (Fig. 2J). Thus, these data indicate that B95-8 EBER2 promotes the expansion of the latently infected B cell pool, which is accompanied by a decrease in the infection of plasma cells.

10.1128/mbio.00836-22.2 FIG S2 The representative flow plots for showing the gating of total and MHV68 latently infected B cells, GC B cells, and plasma cells. (A) Gating total B cells (CD19^+^) and virus-positive B cells (CCF4-AM^+^ CD19^+^). (B) Gating total GC B cells (CD19^+^ GL7^+^) and virus-positive GC B cells (CCF4-AM^+^ CD19^+^ GL7^+^). Infection of GL7^+^ B cells is consistent with previous work demonstrating that MHV68 infects GC B cells defined with more conclusive evaluative markers, including GL7 and CD95 (24). (C) Gating total plasma cells (CD19^low/−^ CD138^+^) and virus-positive plasma cells (CCF4-AM^+^ CD19^low/−^ CD138^+^). Gates for CD19 populations were set at a point 0.1× the observed upper mean fluorescence intensity (MFI), which is consistent with the settings and results obtained from previous publications using B220/CD138 staining for MHV68 16-day infection (22). Download FIG S2, TIF file, 2.5 MB. Copyright © 2022 Wang et al. 2022 Wang et al. https://creativecommons.org/licenses/by/4.0/ This content is distributed under the terms of the Creative Commons Attribution 4.0 International license.

10.1128/mbio.00836-22.3 FIG S3 EBV B95-8 EBER2 polymorphism does not affect the distribution of total B cells, GC B cells, and plasma cells. The percentages of total B cells, GC B cells, and plasma cells in mice i.n. infected with the indicated viruses were analyzed by flow cytometry at 16 dpi. Three mice were used per group per experiment. The values represent the means ± SEM of three to five independent experiments. (A) Percent total B cells (CD19^+^). (B) Percent total GC B cells (CD19^+^ GL7^+^). (C) Percent total plasma cells (CD19^low/−^ CD138^+^). *, *P* < 0.05; determined by Student’s *t*-test. Download FIG S3, TIF file, 1.3 MB. Copyright © 2022 Wang et al. 2022 Wang et al. https://creativecommons.org/licenses/by/4.0/ This content is distributed under the terms of the Creative Commons Attribution 4.0 International license.

Together, these findings cumulatively demonstrate that B95-8 EBER2 promotes B cell latency *in vivo* through a mechanism that involves impaired terminal differentiation to plasma cells. EBV is well known to both induce and repress specific intracellular signaling pathways in B cells. Alteration of these processes is thought to drive the differentiation of infected naive follicular B cells, independent of antigen, through germinal center reactions and into the long-lived memory B cell compartment. Our finding that B95-8 EBER2 may promote B cell latency in part through suppressing differentiation to plasma cells is strongly supported by previous studies that have implicated EBV-encoded LMP1, EBNA3A, and EBNA3C in the repression of terminal differentiation to plasma cells (27, 28). It is notable that previous MHV68 studies have not observed increased GC B cell infection under genetic mutant conditions that reduce plasma cell infection (22, 23, 29). This discrepancy likely points to differences in the requirement of individual genes for highly specific stages in GC reactions. For example, if B95-8 EBER2 promotes recycling through the GC dark zone, this would likely simultaneously result in increased GC B cell proliferation and decreased plasma cell differentiation. In contrast, virus genes that contribute to GC entry would likely reduce infection of both GC B cells and plasma cells. An alternate interpretation is that, compared with M81 EBER2, B95-8 EBER2 viruses may have a reduced ability to promote or maintain infection in B cells that are able to become CD138 positive. Extensive further work will be required to dissect these differences.

Most importantly, our data indicate that the presence of B95-8 EBER2 drives expansion of the latently infected B cell pool, presumably due to additional rounds of proliferation that result from sustained retention of infected cells in the GC. The differences observed here between B95-8 EBER2 and M81 EBER2 contrast from the recently published observation (30) that both EBER2 variants can drive equivalent levels of proliferation in primary B cells *in vitro* and in an immunodeficient xenograft model *in vivo*. However, the difference from our results likely reflects the strength of utilizing a natural infection system in the context of a fully immunocompetent host. This scenario requires virus-infected B cells to navigate the complex, multistage environment of the GC while simultaneously evading immune detection and thus in some situations may reveal differentiation-stage phenotypes that cannot be mimicked accurately in other systems. Our findings with regard to the ability of B95-8 EBER2 to drive increased GC B cell infection is particularly notable due to the high-risk environment of the GC, with GC B cells demonstrating a high propensity for mutation due to the genetic mechanisms that operate during immunoglobulin affinity maturation (reviewed in reference 31). Accordingly, GC B cells are the cellular origins of the majority of B cell lymphomas, including EBV+ Burkitt B cell lymphomas (reviewed in reference 32). Consistent with this, the studies presented here link the ability of EBER2 to expand the latently infected B cell pool, dependent on a 5-nt polymorphism which is found in EBV strain B95-8 and is the predominant variant found in most parts of the world except China. Together, this work provides new insight into a critical role for EBV EBER2 in driving the expansion of the latently infected B cell pool *in vivo* through fine-tuning virus-induced B cell differentiation, provides further insight into key functional differences between polymorphic variants of EBER2, and may point to an unappreciated role for EBER2 in promoting B cell lymphomagenesis.

### Cell culture.

NIH 3T12 murine fibroblasts (ATCC; CCL-164) were grown and maintained in Dulbecco’s modified Eagle’s medium (DMEM; Corning 10-013-CM) containing 10% fetal bovine serum (FBS; Atlanta Biologicals; S12450) and 1× penicillin-streptomycin solution (Corning; 30-002-CI) at 37°C and 5% CO_2_.

### Generation of recombinant viruses.

A recombinant marker virus, MHV68.ORF73βla, expressing β-lactamase as a fusion to mLANA, derived from bacterial artificial chromosome (BAC) containing wild-type (WT) MHV68 (33), has been described previously (34) and used as the parental WT virus here. An MHV68 TMER4 promoter mutant (MHV68.T4.Pro) that is deficient in the expression of the TMER4 and MHV68.ΔT4.E1 virus that expresses EBV EBER1 in place of TMER4 was described previously (8). MHV68 TMER4-deficient viruses, namely, MHV68.ΔT4.E2.b and MHV68.ΔT4.E2.bPro, in which TMER4 was fully replaced by B95-8 EBER2 (E2.b; GenBank no. AJ507799.2) or E2.b with mutations in the polymerase III promoter (designated E2.bPro), respectively, were generated on the MHV68.ORF73βla BAC backbone by en passant mutagenesis, as described previously (13, 35). The sequence of E2.b plus 5′ 100 nucleotides (nts) and 3′ 30 nts was used to replace TMER4 plus 5′ 41 nts and 3′ 9 nts. To generate E2.bPro, the E2.b promoter region A and B boxes GTTGCCCTAGTGGTT and AGGTCAAGTCCC were mutated to AGCTGTTGTCCACAC and CACATTCCCGTA, respectively (Fig. 1A). To determine the contribution of the 5 nts present at positions 44, 46, 57, 61, and 93 in the B95-8 EBER2 to viral latency, a version of the ΔT4.E2.b virus in which those 5 nts were mutated to match the M81 EBER2 polymorphism (designated MHV68.ΔT4.E2.m) was generated on the MHV68.ΔT4.E2.b BAC backbone. Thus, the E2.b and E2.m viruses differed only at positions 44 (T → G), 46 (A → T), 57 (A → G), 61 (A → T), and 93 (A → C) of the EBER2 sequence (Fig. 2A). Mutations in all viruses were verified by enzymatic digestion and sequencing of the purified BAC clone.

### Northern blots.

NIH 3T12 murine fibroblasts were infected with MHV68 at a multiplicity of infection (MOI) of 5 and then harvested and subjected to RNA extraction using TRIzol reagent (Ambion; 15596018) at 18 h postinfection. Northern blots for identification of EBER2 expression were performed as described previously (11), using the RNA probe primer 5′-ATTAGAGAATCCTGACTTGCAAATGCTCT-3′.

### Mice infections.

Seven- to 8-week-old C57BL/6J mice were purchased from the Jackson Laboratory (Bar Harbor, ME) and housed at the University of Florida (Gainesville, FL) in accordance with all federal and university guidelines. All animal protocols were approved by the Institutional Animal Care and Use Committee at the University of Florida. Mice were inoculated intranasally (i.n.) or intraperitoneally (i.p.) with 10^4^ PFU of virus in 30 μL serum-free DMEM under isoflurane anesthesia.

### Latency assays.

Mice were i.n. or i.p. infected with the indicated viruses for 16 days and then the spleens from three mice per group per experiment were harvested, pooled, and homogenized. The red blood cells were removed by treating cells with red cell lysis buffer (0.144 M ammonium chloride and 0.017 M Tris [pH 7.2]). The resulting single-cell suspensions were subsequently filtered through a 100-μm nylon cell strainer (Corning; 352360). Limiting dilution nested PCR (LDPCR) was performed to determine the frequency of splenocytes harboring the MHV68 viral genome, as described previously (7, 13, 36). Briefly, single-cell suspensions were 3-fold diluted serially in a background of RAW 264.7 murine macrophages to maintain a total of 10^4^ cells per PCR. Dilutions were then plated in 96-well PCR plates (Eppendorf; 951020460) at 12 wells per dilution. A positive-control plasmid carrying the MHV68 ORF72 gene was plated at 10, 1, or 0.1 copies per reaction on a background of 10^4^ RAW 264.7 cells. RAW 264.7 cells with no plasmid were also plated as a negative control. Following plating, the cells were lysed with proteinase K at 56°C for 8 h, followed by inactivation at 95°C for 20 min. Two rounds of PCR were then performed using primers specific for MHV68 ORF72 (primers for round 1 PCR, 5′-GAGATCTGTACTCAGGCACCTGT-3′ and 5′-GGATTTCTTGACAGCTCCCTG-3′; and primers for round 2 PCR, 5′-TGTCAGCTGTTGTTGCTCCT-3′ and 5′-CTCCGTCAGGATAACAACGTC-3′), as described previously (7, 36). The resulting 195-bp amplicons were visualized on a 3% agarose gel. Data are expressed as the mean and standard errors of the percentages of PCRs positive for the viral genome at each cell dilution.

### Viral lytic replication assays.

For viral multistep growth curve assays *in vitro*, NIH 3T12 murine fibroblasts were seeded at 2 × 10^5^ cells/well in 6-well plates (Corning; 3506). Twenty-four hours later, the cells were infected with the indicated viruses at an MOI of 0.05 in duplicate. At the indicated time points postinfection, the cells and supernatants were harvested and stored at −80°C. For acute lytic replication assays *in vivo*, mice were i.n. infected with the indicated viruses and then the lungs were harvested at 4 days postinfection (dpi) and stored at −80°C. Subsequently, samples were homogenized, and viral titers were then determined by plaque assays.

### Plaque assays.

Plaque assays were performed as described previously (7, 11, 13). Briefly, NIH 3T12 murine fibroblasts were seeded at 2 × 10^5^ cells/well in 6-well plates 1 day before infection. Samples from infected NIH 3T12 cells (for viral growth curve *in vitro*) or lungs (for acute lytic replication *in vivo*) were 10-fold diluted serially in serum-free DMEM, added to the plates in duplicate, and then overlaid with a 1:1 mixture of methyl cellulose (Acros Organics; 258115000) and 2× modified Eagle medium (Gibco; 11935-046) containing 10% FBS and 1× penicillin-streptomycin solution. Seven days later, neutral red stain was added and plaques were then counted.

### Reactivation assays.

The spleens from three mice per group per experiment were harvested and pooled, and splenocytes were prepared at 16 dpi. Limiting dilution *ex vivo* reactivation assays were then performed to determine the frequency of splenocytes reactivating from latency, as described previously (7, 36). Briefly, the splenocytes were resuspended at 10^6^ cells/mL and subjected to a serial 2-fold dilution. The diluted cells were plated onto permissive mouse embryonic fibroblast monolayers in 96-well tissue culture plates (Corning; 3585) at 24 wells per dilution, starting at 10^5^ cells/well. Cell monolayers were scored for cytopathic effect due to the presence of reactivating virus at 14 to 21 days after plating.

### Flow cytometry.

The infection of B cells, GC B cells, and plasma cells were quantified by flow cytometric analysis using staining with the β-lactamase substrate CCF4-AM (Thermo Fisher Scientific; K1096) for virus-infected cells and staining with a combination of antibodies directed against specific B cell, GC B cell, and plasma cell surface markers, as described previously (13, 34). Briefly, splenocytes from infected mice at 16 dpi were blocked in fluorescence-activated cell sorting (FACS) buffer (phosphate-buffered saline [PBS] supplemented with 2% FBS and 0.1% sodium azide) containing rat anti-mouse CD16/CD32 at 1:50 (BD Biosciences; 553141). The cells were then stained with Alexa Fluor 700 (BD Biosciences; 557958) or APC-R700 (BD Biosciences; 565473) rat anti-mouse CD19 at 1:200, Alexa Fluor 647 rat anti-mouse T- and B-cell activation antigen GL7 at 1:200 (BD Biosciences; 561529), and APC rat anti-mouse CD138 at 1:200 (BD Biosciences; 558626). Following cell surface marker staining, the cells were resuspended and incubated in freshly prepared staining buffer containing the CCF4-AM substrate. FACS acquisition was performed on a BD LSR II or Symphony A3 flow cytometer (BD Biosciences). The cell subsets were gated as follows: total B cells (CD19^+^), virus-positive B cells (CCF4-AM^+^ CD19^+^), total GC B cells (CD19^+^ GL7^+^), virus-positive GC B cells (CCF4-AM^+^ CD19^+^ GL7^+^), total plasma cells (CD19^low/−^ CD138^+^), and virus-positive plasma cells (CCF4-AM^+^ CD19^low/−^ CD138^+^). Data were analyzed using FlowJo v10 software (FlowJo LLC, Ashland, OR).

### Statistical analysis.

All data were analyzed using Prism 8 software (GraphPad, San Diego, CA). The frequencies of cells positive for the viral genome or reactivating virus were determined by Poisson distribution from nonlinear regression, as indicated by the line at 63.2%. Statistical significance was determined by the Student’s *t* test.
