# Supplementary material for: A Polymorphism in the Epstein-Barr Virus EBER2 Noncoding RNA Drives In Vivo Expansion of Latently Infected B Cells
Source: mBio. 2022 Jun 1;13(3):e00836-22. doi: 10.1128/mbio.00836-22 (PMC9239156; doi:10.1128/mbio.00836-22)
Supplement: FIG S1 [file mbio.00836-22-s0001.pdf]

Figure S1. Alignment of EBER2 sequences from EBV genomes. Full-length EBER2 sequences from all GenBank-deposited EBV genomes were aligned with Geneious using the B95-8 sequence (accession V01555) as the reference.

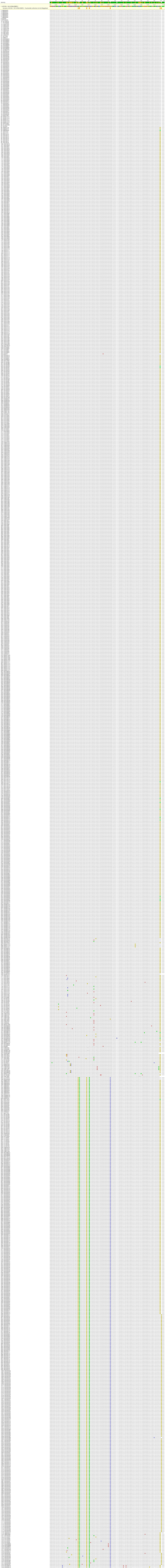
Identity
1. V01555 - Pol III RNA EBER 2
Variants: V01555 - Pol III RNA EBER 2 - Nucleotide collection (nr/nt) Megablast
