# Supplementary material for: A Polymorphism in the Epstein-Barr Virus EBER2 Noncoding RNA Drives In Vivo Expansion of Latently Infected B Cells
Source: mBio. 2022 Jun 1;13(3):e00836-22. doi: 10.1128/mbio.00836-22 (PMC9239156; doi:10.1128/mbio.00836-22)
Supplement: TABLE S1 [file mbio.00836-22-s0004.pdf]

Supplementary Table 1. Location and frequency of EBER2 SNPs.

Name	Minimum	Maximum	Change	Polymorphism Type	Variant Frequency
G	44	44	T -> G	SNP (transversion)	31.70%
T	46	46	A -> T	SNP (transversion)	31.70%
G	57	57	A -> G	SNP (transition)	31.70%
T	61	61	A -> T	SNP (transversion)	31.70%
C	93	93	A -> C	SNP (transversion)	31.80%
G	168	168	A -> G	SNP (transition)	75.40%
G	170	169	+G	Insertion	14.60%
