# Supplementary material for: A Polymorphism in the Epstein-Barr Virus EBER2 Noncoding RNA Drives In Vivo Expansion of Latently Infected B Cells
Source: mBio. 2022 Jun 1;13(3):e00836-22. doi: 10.1128/mbio.00836-22 (PMC9239156; doi:10.1128/mbio.00836-22)
Supplement: TEXT S1 [file mbio.00836-22-s0005.docx]

>V01555_-_Pol_III_RNA_EBER_2 Epstein-Barr virus (EBV) genome, strain B95-8

AGGACAGCCGTTGCCCTAGTGGTTTCGGACACACCGCCAACGCTCAGTGCGGTGCTACCGACCCGAGGTCAAGTCCCGGGGGAGGAGAAGAGAGGCTTCCCG-CCTAGAGCATTTGCAAGTCAGGATTCTCTAATCCCTCTGGGAGAAGGGTATTCGGCTTGTCCGCTAT-TTTT

>AB850645 Human herpesvirus 4 DNA, complete genome, strain: HN10

AGGACAGCCGTTGCCCTAGTGGTTTCGGACACACCGCCAACGCTCAGTGCGGTGCTACCGACCCGAGGTCAAGTCCCGGGGGAGGAGAAGAGAGGCTTCCCG-CCTAGAGCATTTGCAAGTCAGGATTCTCTAATCCCTCTGGGAGAAGGGTATTCGGCTTGTCCGCTAT-TTTT

>AB850647 Human herpesvirus 4 DNA, complete genome, strain: HN12

AGGACAGCCGTTGCCCTAGTGGTTTCGGACACACCGCCAACGCTCAGTGCGGTGCTACCGACCCGAGGTCAAGTCCCGGGGGAGGAGAAGAGAGGCTTCCCG-CCTAGAGCATTTGCAAGTCAGGATTCTCTAATCCCTCTGGGAGAAGGGTATTCGGCTTGTCCGCTAT-TTTT

>AB850648 Human herpesvirus 4 DNA, complete genome, strain: HN3

AGGACAGCCGTTGCCCTAGTGGTTTCGGACACACCGCCAACGCTCAGTGCGGTGCTACCGACCCGAGGTCAAGTCCCGGGGGAGGAGAAGAGAGGCTTCCCG-CCTAGAGCATTTGCAAGTCAGGATTCTCTAATCCCTCTGGGAGAAGGGTATTCGGCTTGTCCGCTAT-TTTT

>AB850654 Human herpesvirus 4 DNA, complete genome, strain: HN15

AGGACAGCCGTTGCCCTAGTGGTTTCGGACACACCGCCAACGCTCAGTGCGGTGCTACCGACCCGAGGTCAAGTCCCGGGGGAGGAGAAGAGAGGCTTCCCG-CCTAGAGCATTTGCAAGTCAGGATTCTCTAATCCCTCTGGGAGAAGGGTATTCGGCTTGTCCGCTAT-TTTT

>AB850655 Human herpesvirus 4 DNA, complete genome, strain: HN7

AGGACAGCCGTTGCCCTAGTGGTTTCGGACACACCGCCAACGCTCAGTGCGGTGCTACCGACCCGAGGTCAAGTCCCGGGGGAGGAGAAGAGAGGCTTCCCG-CCTAGAGCATTTGCAAGTCAGGATTCTCTAATCCCTCTGGGAGAAGGGTATTCGGCTTGTCCGCTAT-TTTT

>AB850658 Human herpesvirus 4 DNA, complete genome, strain: HN17

AGGACAGCCGTTGCCCTAGTGGTTTCGGACACACCGCCAACGCTCAGTGCGGTGCTACCGACCCGAGGTCAAGTCCCGGGGGAGGAGAAGAGAGGCTTCCCG-CCTAGAGCATTTGCAAGTCAGGATTCTCTAATCCCTCTGGGAGAAGGGTATTCGGCTTGTCCGCTAT-TTTT

>J02078 epstein-barr virus small encoded rna genes eber 1 & eber 2

AGGACAGCCGTTGCCCTAGTGGTTTCGGACACACCGCCAACGCTCAGTGCGGTGCTACCGACCCGAGGTCAAGTCCCGGGGGAGGAGAAGAGAGGCTTCCCG-CCTAGAGCATTTGCAAGTCAGGATTCTCTAATCCCTCTGGGAGAAGGGTATTCGGCTTGTCCGCTAT-TTTT

>KP195533 Human herpesvirus 4 isolate GDTW9 EBER1 and EBER2 genes, complete sequence

AGGACAGCCGTTGCCCTAGTGGTTTCGGACACACCGCCAACGCTCAGTGCGGTGCTACCGACCCGAGGTCAAGTCCCGGGGGAGGAGAAGAGAGGCTTCCCG-CCTAGAGCATTTGCAAGTCAGGATTCTCTAATCCCTCTGGGAGAAGGGTATTCGGCTTGTCCGCTAT-TTTT

>KP195623 Human herpesvirus 4 isolate SDTW83 EBER1 and EBER2 genes, complete sequence

AGGACAGCCGTTGCCCTAGTGGTTTCGGACACACCGCCAACGCTCAGTGCGGTGCTACCGACCCGAGGTCAAGTCCCGGGGGAGGAGAAGAGAGGCTTCCCG-CCTAGAGCATTTGCAAGTCAGGATTCTCTAATCCCTCTGGGAGAAGGGTATTCGGCTTGTCCGCTAT-TTTT

>KP195658 Human herpesvirus 4 isolate SDTW156 EBER1 and EBER2 genes, complete sequence

AGGACAGCCGTTGCCCTAGTGGTTTCGGACACACCGCCAACGCTCAGTGCGGTGCTACCGACCCGAGGTCAAGTCCCGGGGGAGGAGAAGAGAGGCTTCCCG-CCTAGAGCATTTGCAAGTCAGGATTCTCTAATCCCTCTGGGAGAAGGGTATTCGGCTTGTCCGCTAT-TTTT

>LN827550 Human herpesvirus 4 genome assembly sLCL-1.11, segment : I

AGGACAGCCGTTGCCCTAGTGGTTTCGGACACACCGCCAACGCTCAGTGCGGTGCTACCGACCCGAGGTCAAGTCCCGGGGGAGGAGAAGAGAGGCTTCCCG-CCTAGAGCATTTGCAAGTCAGGATTCTCTAATCCCTCTGGGAGAAGGGTATTCGGCTTGTCCGCTAT-TTTT

>LN827555 Human herpesvirus 4 genome assembly X50-7, segment : I

AGGACAGCCGTTGCCCTAGTGGTTTCGGACACACCGCCAACGCTCAGTGCGGTGCTACCGACCCGAGGTCAAGTCCCGGGGGAGGAGAAGAGAGGCTTCCCG-CCTAGAGCATTTGCAAGTCAGGATTCTCTAATCCCTCTGGGAGAAGGGTATTCGGCTTGTCCGCTAT-TTTT

>LN827557 Human herpesvirus 4 genome assembly BL36, segment : I

AGGACAGCCGTTGCCCTAGTGGTTTCGGACACACCGCCAACGCTCAGTGCGGTGCTACCGACCCGAGGTCAAGTCCCGGGGGAGGAGAAGAGAGGCTTCCCG-CCTAGAGCATTTGCAAGTCAGGATTCTCTAATCCCTCTGGGAGAAGGGTATTCGGCTTGTCCGCTAT-TTTT

>LN827558 Human herpesvirus 4 genome assembly sLCL-1.02, segment : I

AGGACAGCCGTTGCCCTAGTGGTTTCGGACACACCGCCAACGCTCAGTGCGGTGCTACCGACCCGAGGTCAAGTCCCGGGGGAGGAGAAGAGAGGCTTCCCG-CCTAGAGCATTTGCAAGTCAGGATTCTCTAATCCCTCTGGGAGAAGGGTATTCGGCTTGTCCGCTAT-TTTT

>LN827572 Human herpesvirus 4 genome assembly sLCL-IS1.18, segment : I

AGGACAGCCGTTGCCCTAGTGGTTTCGGACACACCGCCAACGCTCAGTGCGGTGCTACCGACCCGAGGTCAAGTCCCGGGGGAGGAGAAGAGAGGCTTCCCG-CCTAGAGCATTTGCAAGTCAGGATTCTCTAATCCCTCTGGGAGAAGGGTATTCGGCTTGTCCGCTAT-TTTT

>LN827597 Human herpesvirus 4 genome assembly sLCL-IS1.04, segment : I

AGGACAGCCGTTGCCCTAGTGGTTTCGGACACACCGCCAACGCTCAGTGCGGTGCTACCGACCCGAGGTCAAGTCCCGGGGGAGGAGAAGAGAGGCTTCCCG-CCTAGAGCATTTGCAAGTCAGGATTCTCTAATCCCTCTGGGAGAAGGGTATTCGGCTTGTCCGCTAT-TTTT

>LR812925 Human gammaherpesvirus 4 isolate eBL_CL-01 genome assembly, chromosome: EBV

AGGACAGCCGTTGCCCTAGTGGTTTCGGACACACCGCCAACGCTCAGTGCGGTGCTACCGACCCGAGGTCAAGTCCCGGGGGAGGAGAAGAGAGGCTTCCCG-CCTAGAGCATTTGCAAGTCAGGATTCTCTAATCCCTCTGGGAGAAGGGTATTCGGCTTGTCCGCTAT-TTTT

>LR813032 Human gammaherpesvirus 4 isolate eBL-Tumor-0014 genome assembly, chromosome: EBV

AGGACAGCCGTTGCCCTAGTGGTTTCGGACACACCGCCAACGCTCAGTGCGGTGCTACCGACCCGAGGTCAAGTCCCGGGGGAGGAGAAGAGAGGCTTCCCG-CCTAGAGCATTTGCAAGTCAGGATTCTCTAATCCCTCTGGGAGAAGGGTATTCGGCTTGTCCGCTAT-TTTT

>LS992257 Human gammaherpesvirus 4 isolate Human herpesvirus 4 genome assembly, chromosome: I

AGGACAGCCGTTGCCCTAGTGGTTTCGGACACACCGCCAACGCTCAGTGCGGTGCTACCGACCCGAGGTCAAGTCCCGGGGGAGGAGAAGAGAGGCTTCCCG-CCTAGAGCATTTGCAAGTCAGGATTCTCTAATCCCTCTGGGAGAAGGGTATTCGGCTTGTCCGCTAT-TTTT

>M11924 Epstein-Barr virus B95-8, 12kb short unique region

AGGACAGCCGTTGCCCTAGTGGTTTCGGACACACCGCCAACGCTCAGTGCGGTGCTACCGACCCGAGGTCAAGTCCCGGGGGAGGAGAAGAGAGGCTTCCCG-CCTAGAGCATTTGCAAGTCAGGATTCTCTAATCCCTCTGGGAGAAGGGTATTCGGCTTGTCCGCTAT-TTTT

>M80517 Epstein-Barr virus, artifactual joining of B95-8 complete genome and the sequences from Raji of the large deletion found in B95-8

AGGACAGCCGTTGCCCTAGTGGTTTCGGACACACCGCCAACGCTCAGTGCGGTGCTACCGACCCGAGGTCAAGTCCCGGGGGAGGAGAAGAGAGGCTTCCCG-CCTAGAGCATTTGCAAGTCAGGATTCTCTAATCCCTCTGGGAGAAGGGTATTCGGCTTGTCCGCTAT-TTTT

>MG298833 Human gammaherpesvirus 4 isolate GK_BL36

AGGACAGCCGTTGCCCTAGTGGTTTCGGACACACCGCCAACGCTCAGTGCGGTGCTACCGACCCGAGGTCAAGTCCCGGGGGAGGAGAAGAGAGGCTTCCCG-CCTAGAGCATTTGCAAGTCAGGATTCTCTAATCCCTCTGGGAGAAGGGTATTCGGCTTGTCCGCTAT-TTTT

>MG298834 Human gammaherpesvirus 4 isolate GK_BL42

AGGACAGCCGTTGCCCTAGTGGTTTCGGACACACCGCCAACGCTCAGTGCGGTGCTACCGACCCGAGGTCAAGTCCCGGGGGAGGAGAAGAGAGGCTTCCCG-CCTAGAGCATTTGCAAGTCAGGATTCTCTAATCCCTCTGGGAGAAGGGTATTCGGCTTGTCCGCTAT-TTTT

>MG298835 Human gammaherpesvirus 4 isolate GK_BL44

AGGACAGCCGTTGCCCTAGTGGTTTCGGACACACCGCCAACGCTCAGTGCGGTGCTACCGACCCGAGGTCAAGTCCCGGGGGAGGAGAAGAGAGGCTTCCCG-CCTAGAGCATTTGCAAGTCAGGATTCTCTAATCCCTCTGGGAGAAGGGTATTCGGCTTGTCCGCTAT-TTTT

>MG298841 Human gammaherpesvirus 4 isolate GK_LY65

AGGACAGCCGTTGCCCTAGTGGTTTCGGACACACCGCCAACGCTCAGTGCGGTGCTACCGACCCGAGGTCAAGTCCCGGGGGAGGAGAAGAGAGGCTTCCCG-CCTAGAGCATTTGCAAGTCAGGATTCTCTAATCCCTCTGGGAGAAGGGTATTCGGCTTGTCCGCTAT-TTTT

>MG298844 Human gammaherpesvirus 4 isolate IMS_Saliva_10

AGGACAGCCGTTGCCCTAGTGGTTTCGGACACACCGCCAACGCTCAGTGCGGTGCTACCGACCCGAGGTCAAGTCCCGGGGGAGGAGAAGAGAGGCTTCCCG-CCTAGAGCATTTGCAAGTCAGGATTCTCTAATCCCTCTGGGAGAAGGGTATTCGGCTTGTCCGCTAT-TTTT

>MG298847 Human gammaherpesvirus 4 isolate IMS_Saliva_162

AGGACAGCCGTTGCCCTAGTGGTTTCGGACACACCGCCAACGCTCAGTGCGGTGCTACCGACCCGAGGTCAAGTCCCGGGGGAGGAGAAGAGAGGCTTCCCG-CCTAGAGCATTTGCAAGTCAGGATTCTCTAATCCCTCTGGGAGAAGGGTATTCGGCTTGTCCGCTAT-TTTT

>MG298856 Human gammaherpesvirus 4 isolate IMS_Saliva_250

AGGACAGCCGTTGCCCTAGTGGTTTCGGACACACCGCCAACGCTCAGTGCGGTGCTACCGACCCGAGGTCAAGTCCCGGGGGAGGAGAAGAGAGGCTTCCCG-CCTAGAGCATTTGCAAGTCAGGATTCTCTAATCCCTCTGGGAGAAGGGTATTCGGCTTGTCCGCTAT-TTTT

>MG298905 Human gammaherpesvirus 4 isolate JWBL43B

AGGACAGCCGTTGCCCTAGTGGTTTCGGACACACCGCCAACGCTCAGTGCGGTGCTACCGACCCGAGGTCAAGTCCCGGGGGAGGAGAAGAGAGGCTTCCCG-CCTAGAGCATTTGCAAGTCAGGATTCTCTAATCCCTCTGGGAGAAGGGTATTCGGCTTGTCCGCTAT-TTTT

>MH883775 Human gammaherpesvirus 4 isolate P2-T1

AGGACAGCCGTTGCCCTAGTGGTTTCGGACACACCGCCAACGCTCAGTGCGGTGCTACCGACCCGAGGTCAAGTCCCGGGGGAGGAGAAGAGAGGCTTCCCG-CCTAGAGCATTTGCAAGTCAGGATTCTCTAATCCCTCTGGGAGAAGGGTATTCGGCTTGTCCGCTAT-TTTT

>MK540244 Human gammaherpesvirus 4 isolate GCT001

AGGACAGCCGTTGCCCTAGTGGTTTCGGACACACCGCCAACGCTCAGTGCGGTGCTACCGACCCGAGGTCAAGTCCCGGGGGAGGAGAAGAGAGGCTTCCCG-CCTAGAGCATTTGCAAGTCAGGATTCTCTAATCCCTCTGGGAGAAGGGTATTCGGCTTGTCCGCTAT-TTTT

>MK540245 Human gammaherpesvirus 4 isolate GCT002

AGGACAGCCGTTGCCCTAGTGGTTTCGGACACACCGCCAACGCTCAGTGCGGTGCTACCGACCCGAGGTCAAGTCCCGGGGGAGGAGAAGAGAGGCTTCCCG-CCTAGAGCATTTGCAAGTCAGGATTCTCTAATCCCTCTGGGAGAAGGGTATTCGGCTTGTCCGCTAT-TTTT

>MK540246 Human gammaherpesvirus 4 isolate GCT003

AGGACAGCCGTTGCCCTAGTGGTTTCGGACACACCGCCAACGCTCAGTGCGGTGCTACCGACCCGAGGTCAAGTCCCGGGGGAGGAGAAGAGAGGCTTCCCG-CCTAGAGCATTTGCAAGTCAGGATTCTCTAATCCCTCTGGGAGAAGGGTATTCGGCTTGTCCGCTAT-TTTT

>MK540247 Human gammaherpesvirus 4 isolate GCT004

AGGACAGCCGTTGCCCTAGTGGTTTCGGACACACCGCCAACGCTCAGTGCGGTGCTACCGACCCGAGGTCAAGTCCCGGGGGAGGAGAAGAGAGGCTTCCCG-CCTAGAGCATTTGCAAGTCAGGATTCTCTAATCCCTCTGGGAGAAGGGTATTCGGCTTGTCCGCTAT-TTTT

>MK540248 Human gammaherpesvirus 4 isolate GCT005

AGGACAGCCGTTGCCCTAGTGGTTTCGGACACACCGCCAACGCTCAGTGCGGTGCTACCGACCCGAGGTCAAGTCCCGGGGGAGGAGAAGAGAGGCTTCCCG-CCTAGAGCATTTGCAAGTCAGGATTCTCTAATCCCTCTGGGAGAAGGGTATTCGGCTTGTCCGCTAT-TTTT

>MK540249 Human gammaherpesvirus 4 isolate GCT006

AGGACAGCCGTTGCCCTAGTGGTTTCGGACACACCGCCAACGCTCAGTGCGGTGCTACCGACCCGAGGTCAAGTCCCGGGGGAGGAGAAGAGAGGCTTCCCG-CCTAGAGCATTTGCAAGTCAGGATTCTCTAATCCCTCTGGGAGAAGGGTATTCGGCTTGTCCGCTAT-TTTT

>MK540250 Human gammaherpesvirus 4 isolate GCT007

AGGACAGCCGTTGCCCTAGTGGTTTCGGACACACCGCCAACGCTCAGTGCGGTGCTACCGACCCGAGGTCAAGTCCCGGGGGAGGAGAAGAGAGGCTTCCCG-CCTAGAGCATTTGCAAGTCAGGATTCTCTAATCCCTCTGGGAGAAGGGTATTCGGCTTGTCCGCTAT-TTTT

>MK540251 Human gammaherpesvirus 4 isolate GCT009

AGGACAGCCGTTGCCCTAGTGGTTTCGGACACACCGCCAACGCTCAGTGCGGTGCTACCGACCCGAGGTCAAGTCCCGGGGGAGGAGAAGAGAGGCTTCCCG-CCTAGAGCATTTGCAAGTCAGGATTCTCTAATCCCTCTGGGAGAAGGGTATTCGGCTTGTCCGCTAT-TTTT

>MK540252 Human gammaherpesvirus 4 isolate GCT010

AGGACAGCCGTTGCCCTAGTGGTTTCGGACACACCGCCAACGCTCAGTGCGGTGCTACCGACCCGAGGTCAAGTCCCGGGGGAGGAGAAGAGAGGCTTCCCG-CCTAGAGCATTTGCAAGTCAGGATTCTCTAATCCCTCTGGGAGAAGGGTATTCGGCTTGTCCGCTAT-TTTT

>MK540253 Human gammaherpesvirus 4 isolate GCT011

AGGACAGCCGTTGCCCTAGTGGTTTCGGACACACCGCCAACGCTCAGTGCGGTGCTACCGACCCGAGGTCAAGTCCCGGGGGAGGAGAAGAGAGGCTTCCCG-CCTAGAGCATTTGCAAGTCAGGATTCTCTAATCCCTCTGGGAGAAGGGTATTCGGCTTGTCCGCTAT-TTTT

>MK540254 Human gammaherpesvirus 4 isolate GCT012

AGGACAGCCGTTGCCCTAGTGGTTTCGGACACACCGCCAACGCTCAGTGCGGTGCTACCGACCCGAGGTCAAGTCCCGGGGGAGGAGAAGAGAGGCTTCCCG-CCTAGAGCATTTGCAAGTCAGGATTCTCTAATCCCTCTGGGAGAAGGGTATTCGGCTTGTCCGCTAT-TTTT

>MK540255 Human gammaherpesvirus 4 isolate GCT013

AGGACAGCCGTTGCCCTAGTGGTTTCGGACACACCGCCAACGCTCAGTGCGGTGCTACCGACCCGAGGTCAAGTCCCGGGGGAGGAGAAGAGAGGCTTCCCG-CCTAGAGCATTTGCAAGTCAGGATTCTCTAATCCCTCTGGGAGAAGGGTATTCGGCTTGTCCGCTAT-TTTT

>MK540257 Human gammaherpesvirus 4 isolate HLT001

AGGACAGCCGTTGCCCTAGTGGTTTCGGACACACCGCCAACGCTCAGTGCGGTGCTACCGACCCGAGGTCAAGTCCCGGGGGAGGAGAAGAGAGGCTTCCCG-CCTAGAGCATTTGCAAGTCAGGATTCTCTAATCCCTCTGGGAGAAGGGTATTCGGCTTGTCCGCTAT-TTTT

>MK540259 Human gammaherpesvirus 4 isolate HLT005

AGGACAGCCGTTGCCCTAGTGGTTTCGGACACACCGCCAACGCTCAGTGCGGTGCTACCGACCCGAGGTCAAGTCCCGGGGGAGGAGAAGAGAGGCTTCCCG-CCTAGAGCATTTGCAAGTCAGGATTCTCTAATCCCTCTGGGAGAAGGGTATTCGGCTTGTCCGCTAT-TTTT

>MK540260 Human gammaherpesvirus 4 isolate HLT006

AGGACAGCCGTTGCCCTAGTGGTTTCGGACACACCGCCAACGCTCAGTGCGGTGCTACCGACCCGAGGTCAAGTCCCGGGGGAGGAGAAGAGAGGCTTCCCG-CCTAGAGCATTTGCAAGTCAGGATTCTCTAATCCCTCTGGGAGAAGGGTATTCGGCTTGTCCGCTAT-TTTT

>MK540261 Human gammaherpesvirus 4 isolate HLT007

AGGACAGCCGTTGCCCTAGTGGTTTCGGACACACCGCCAACGCTCAGTGCGGTGCTACCGACCCGAGGTCAAGTCCCGGGGGAGGAGAAGAGAGGCTTCCCG-CCTAGAGCATTTGCAAGTCAGGATTCTCTAATCCCTCTGGGAGAAGGGTATTCGGCTTGTCCGCTAT-TTTT

>MK540262 Human gammaherpesvirus 4 isolate HLT010

AGGACAGCCGTTGCCCTAGTGGTTTCGGACACACCGCCAACGCTCAGTGCGGTGCTACCGACCCGAGGTCAAGTCCCGGGGGAGGAGAAGAGAGGCTTCCCG-CCTAGAGCATTTGCAAGTCAGGATTCTCTAATCCCTCTGGGAGAAGGGTATTCGGCTTGTCCGCTAT-TTTT

>MK540263 Human gammaherpesvirus 4 isolate HLT011

AGGACAGCCGTTGCCCTAGTGGTTTCGGACACACCGCCAACGCTCAGTGCGGTGCTACCGACCCGAGGTCAAGTCCCGGGGGAGGAGAAGAGAGGCTTCCCG-CCTAGAGCATTTGCAAGTCAGGATTCTCTAATCCCTCTGGGAGAAGGGTATTCGGCTTGTCCGCTAT-TTTT

>MK540264 Human gammaherpesvirus 4 isolate HS001

AGGACAGCCGTTGCCCTAGTGGTTTCGGACACACCGCCAACGCTCAGTGCGGTGCTACCGACCCGAGGTCAAGTCCCGGGGGAGGAGAAGAGAGGCTTCCCG-CCTAGAGCATTTGCAAGTCAGGATTCTCTAATCCCTCTGGGAGAAGGGTATTCGGCTTGTCCGCTAT-TTTT

>MK540269 Human gammaherpesvirus 4 isolate HS011

AGGACAGCCGTTGCCCTAGTGGTTTCGGACACACCGCCAACGCTCAGTGCGGTGCTACCGACCCGAGGTCAAGTCCCGGGGGAGGAGAAGAGAGGCTTCCCG-CCTAGAGCATTTGCAAGTCAGGATTCTCTAATCCCTCTGGGAGAAGGGTATTCGGCTTGTCCGCTAT-TTTT

>MK540273 Human gammaherpesvirus 4 isolate HS015

AGGACAGCCGTTGCCCTAGTGGTTTCGGACACACCGCCAACGCTCAGTGCGGTGCTACCGACCCGAGGTCAAGTCCCGGGGGAGGAGAAGAGAGGCTTCCCG-CCTAGAGCATTTGCAAGTCAGGATTCTCTAATCCCTCTGGGAGAAGGGTATTCGGCTTGTCCGCTAT-TTTT

>MK540279 Human gammaherpesvirus 4 isolate HS023

AGGACAGCCGTTGCCCTAGTGGTTTCGGACACACCGCCAACGCTCAGTGCGGTGCTACCGACCCGAGGTCAAGTCCCGGGGGAGGAGAAGAGAGGCTTCCCG-CCTAGAGCATTTGCAAGTCAGGATTCTCTAATCCCTCTGGGAGAAGGGTATTCGGCTTGTCCGCTAT-TTTT

>MK540280 Human gammaherpesvirus 4 isolate HS024

AGGACAGCCGTTGCCCTAGTGGTTTCGGACACACCGCCAACGCTCAGTGCGGTGCTACCGACCCGAGGTCAAGTCCCGGGGGAGGAGAAGAGAGGCTTCCCG-CCTAGAGCATTTGCAAGTCAGGATTCTCTAATCCCTCTGGGAGAAGGGTATTCGGCTTGTCCGCTAT-TTTT

>MK540284 Human gammaherpesvirus 4 isolate HS032

AGGACAGCCGTTGCCCTAGTGGTTTCGGACACACCGCCAACGCTCAGTGCGGTGCTACCGACCCGAGGTCAAGTCCCGGGGGAGGAGAAGAGAGGCTTCCCG-CCTAGAGCATTTGCAAGTCAGGATTCTCTAATCCCTCTGGGAGAAGGGTATTCGGCTTGTCCGCTAT-TTTT

>MK540285 Human gammaherpesvirus 4 isolate HS033

AGGACAGCCGTTGCCCTAGTGGTTTCGGACACACCGCCAACGCTCAGTGCGGTGCTACCGACCCGAGGTCAAGTCCCGGGGGAGGAGAAGAGAGGCTTCCCG-CCTAGAGCATTTGCAAGTCAGGATTCTCTAATCCCTCTGGGAGAAGGGTATTCGGCTTGTCCGCTAT-TTTT

>MK540286 Human gammaherpesvirus 4 isolate HS034

AGGACAGCCGTTGCCCTAGTGGTTTCGGACACACCGCCAACGCTCAGTGCGGTGCTACCGACCCGAGGTCAAGTCCCGGGGGAGGAGAAGAGAGGCTTCCCG-CCTAGAGCATTTGCAAGTCAGGATTCTCTAATCCCTCTGGGAGAAGGGTATTCGGCTTGTCCGCTAT-TTTT

>MK540287 Human gammaherpesvirus 4 isolate HS035

AGGACAGCCGTTGCCCTAGTGGTTTCGGACACACCGCCAACGCTCAGTGCGGTGCTACCGACCCGAGGTCAAGTCCCGGGGGAGGAGAAGAGAGGCTTCCCG-CCTAGAGCATTTGCAAGTCAGGATTCTCTAATCCCTCTGGGAGAAGGGTATTCGGCTTGTCCGCTAT-TTTT

>MK540288 Human gammaherpesvirus 4 isolate HS036

AGGACAGCCGTTGCCCTAGTGGTTTCGGACACACCGCCAACGCTCAGTGCGGTGCTACCGACCCGAGGTCAAGTCCCGGGGGAGGAGAAGAGAGGCTTCCCG-CCTAGAGCATTTGCAAGTCAGGATTCTCTAATCCCTCTGGGAGAAGGGTATTCGGCTTGTCCGCTAT-TTTT

>MK540291 Human gammaherpesvirus 4 isolate HS039

AGGACAGCCGTTGCCCTAGTGGTTTCGGACACACCGCCAACGCTCAGTGCGGTGCTACCGACCCGAGGTCAAGTCCCGGGGGAGGAGAAGAGAGGCTTCCCG-CCTAGAGCATTTGCAAGTCAGGATTCTCTAATCCCTCTGGGAGAAGGGTATTCGGCTTGTCCGCTAT-TTTT

>MK540292 Human gammaherpesvirus 4 isolate HS041

AGGACAGCCGTTGCCCTAGTGGTTTCGGACACACCGCCAACGCTCAGTGCGGTGCTACCGACCCGAGGTCAAGTCCCGGGGGAGGAGAAGAGAGGCTTCCCG-CCTAGAGCATTTGCAAGTCAGGATTCTCTAATCCCTCTGGGAGAAGGGTATTCGGCTTGTCCGCTAT-TTTT

>MK540294 Human gammaherpesvirus 4 isolate HS048

AGGACAGCCGTTGCCCTAGTGGTTTCGGACACACCGCCAACGCTCAGTGCGGTGCTACCGACCCGAGGTCAAGTCCCGGGGGAGGAGAAGAGAGGCTTCCCG-CCTAGAGCATTTGCAAGTCAGGATTCTCTAATCCCTCTGGGAGAAGGGTATTCGGCTTGTCCGCTAT-TTTT

>MK540300 Human gammaherpesvirus 4 isolate HS057

AGGACAGCCGTTGCCCTAGTGGTTTCGGACACACCGCCAACGCTCAGTGCGGTGCTACCGACCCGAGGTCAAGTCCCGGGGGAGGAGAAGAGAGGCTTCCCG-CCTAGAGCATTTGCAAGTCAGGATTCTCTAATCCCTCTGGGAGAAGGGTATTCGGCTTGTCCGCTAT-TTTT

>MK540301 Human gammaherpesvirus 4 isolate NHS002

AGGACAGCCGTTGCCCTAGTGGTTTCGGACACACCGCCAACGCTCAGTGCGGTGCTACCGACCCGAGGTCAAGTCCCGGGGGAGGAGAAGAGAGGCTTCCCG-CCTAGAGCATTTGCAAGTCAGGATTCTCTAATCCCTCTGGGAGAAGGGTATTCGGCTTGTCCGCTAT-TTTT

>MK540302 Human gammaherpesvirus 4 isolate NHS004

AGGACAGCCGTTGCCCTAGTGGTTTCGGACACACCGCCAACGCTCAGTGCGGTGCTACCGACCCGAGGTCAAGTCCCGGGGGAGGAGAAGAGAGGCTTCCCG-CCTAGAGCATTTGCAAGTCAGGATTCTCTAATCCCTCTGGGAGAAGGGTATTCGGCTTGTCCGCTAT-TTTT

>MK540303 Human gammaherpesvirus 4 isolate NKLT002

AGGACAGCCGTTGCCCTAGTGGTTTCGGACACACCGCCAACGCTCAGTGCGGTGCTACCGACCCGAGGTCAAGTCCCGGGGGAGGAGAAGAGAGGCTTCCCG-CCTAGAGCATTTGCAAGTCAGGATTCTCTAATCCCTCTGGGAGAAGGGTATTCGGCTTGTCCGCTAT-TTTT

>MK540304 Human gammaherpesvirus 4 isolate NKLT003-2

AGGACAGCCGTTGCCCTAGTGGTTTCGGACACACCGCCAACGCTCAGTGCGGTGCTACCGACCCGAGGTCAAGTCCCGGGGGAGGAGAAGAGAGGCTTCCCG-CCTAGAGCATTTGCAAGTCAGGATTCTCTAATCCCTCTGGGAGAAGGGTATTCGGCTTGTCCGCTAT-TTTT

>MK540307 Human gammaherpesvirus 4 isolate NKLT007

AGGACAGCCGTTGCCCTAGTGGTTTCGGACACACCGCCAACGCTCAGTGCGGTGCTACCGACCCGAGGTCAAGTCCCGGGGGAGGAGAAGAGAGGCTTCCCG-CCTAGAGCATTTGCAAGTCAGGATTCTCTAATCCCTCTGGGAGAAGGGTATTCGGCTTGTCCGCTAT-TTTT

>MK540312 Human gammaherpesvirus 4 isolate NNPCT005

AGGACAGCCGTTGCCCTAGTGGTTTCGGACACACCGCCAACGCTCAGTGCGGTGCTACCGACCCGAGGTCAAGTCCCGGGGGAGGAGAAGAGAGGCTTCCCG-CCTAGAGCATTTGCAAGTCAGGATTCTCTAATCCCTCTGGGAGAAGGGTATTCGGCTTGTCCGCTAT-TTTT

>MK540341 Human gammaherpesvirus 4 isolate NPCS031

AGGACAGCCGTTGCCCTAGTGGTTTCGGACACACCGCCAACGCTCAGTGCGGTGCTACCGACCCGAGGTCAAGTCCCGGGGGAGGAGAAGAGAGGCTTCCCG-CCTAGAGCATTTGCAAGTCAGGATTCTCTAATCCCTCTGGGAGAAGGGTATTCGGCTTGTCCGCTAT-TTTT

>MK540354 Human gammaherpesvirus 4 isolate NPCS049

AGGACAGCCGTTGCCCTAGTGGTTTCGGACACACCGCCAACGCTCAGTGCGGTGCTACCGACCCGAGGTCAAGTCCCGGGGGAGGAGAAGAGAGGCTTCCCG-CCTAGAGCATTTGCAAGTCAGGATTCTCTAATCCCTCTGGGAGAAGGGTATTCGGCTTGTCCGCTAT-TTTT

>MK540367 Human gammaherpesvirus 4 isolate NPCT009

AGGACAGCCGTTGCCCTAGTGGTTTCGGACACACCGCCAACGCTCAGTGCGGTGCTACCGACCCGAGGTCAAGTCCCGGGGGAGGAGAAGAGAGGCTTCCCG-CCTAGAGCATTTGCAAGTCAGGATTCTCTAATCCCTCTGGGAGAAGGGTATTCGGCTTGTCCGCTAT-TTTT

>MK540369 Human gammaherpesvirus 4 isolate NPCT011

AGGACAGCCGTTGCCCTAGTGGTTTCGGACACACCGCCAACGCTCAGTGCGGTGCTACCGACCCGAGGTCAAGTCCCGGGGGAGGAGAAGAGAGGCTTCCCG-CCTAGAGCATTTGCAAGTCAGGATTCTCTAATCCCTCTGGGAGAAGGGTATTCGGCTTGTCCGCTAT-TTTT

>MK540378 Human gammaherpesvirus 4 isolate NPCT021

AGGACAGCCGTTGCCCTAGTGGTTTCGGACACACCGCCAACGCTCAGTGCGGTGCTACCGACCCGAGGTCAAGTCCCGGGGGAGGAGAAGAGAGGCTTCCCG-CCTAGAGCATTTGCAAGTCAGGATTCTCTAATCCCTCTGGGAGAAGGGTATTCGGCTTGTCCGCTAT-TTTT

>MK540402 Human gammaherpesvirus 4 isolate NPCT049

AGGACAGCCGTTGCCCTAGTGGTTTCGGACACACCGCCAACGCTCAGTGCGGTGCTACCGACCCGAGGTCAAGTCCCGGGGGAGGAGAAGAGAGGCTTCCCG-CCTAGAGCATTTGCAAGTCAGGATTCTCTAATCCCTCTGGGAGAAGGGTATTCGGCTTGTCCGCTAT-TTTT

>MK540417 Human gammaherpesvirus 4 isolate NPCT060

AGGACAGCCGTTGCCCTAGTGGTTTCGGACACACCGCCAACGCTCAGTGCGGTGCTACCGACCCGAGGTCAAGTCCCGGGGGAGGAGAAGAGAGGCTTCCCG-CCTAGAGCATTTGCAAGTCAGGATTCTCTAATCCCTCTGGGAGAAGGGTATTCGGCTTGTCCGCTAT-TTTT

>MK540418 Human gammaherpesvirus 4 isolate NPCT061

AGGACAGCCGTTGCCCTAGTGGTTTCGGACACACCGCCAACGCTCAGTGCGGTGCTACCGACCCGAGGTCAAGTCCCGGGGGAGGAGAAGAGAGGCTTCCCG-CCTAGAGCATTTGCAAGTCAGGATTCTCTAATCCCTCTGGGAGAAGGGTATTCGGCTTGTCCGCTAT-TTTT

>MK540446 Human gammaherpesvirus 4 isolate NPCT089

AGGACAGCCGTTGCCCTAGTGGTTTCGGACACACCGCCAACGCTCAGTGCGGTGCTACCGACCCGAGGTCAAGTCCCGGGGGAGGAGAAGAGAGGCTTCCCG-CCTAGAGCATTTGCAAGTCAGGATTCTCTAATCCCTCTGGGAGAAGGGTATTCGGCTTGTCCGCTAT-TTTT

>MK973061 Human gammaherpesvirus 4 strain IM-3

AGGACAGCCGTTGCCCTAGTGGTTTCGGACACACCGCCAACGCTCAGTGCGGTGCTACCGACCCGAGGTCAAGTCCCGGGGGAGGAGAAGAGAGGCTTCCCG-CCTAGAGCATTTGCAAGTCAGGATTCTCTAATCCCTCTGGGAGAAGGGTATTCGGCTTGTCCGCTAT-TTTT

>MN921215 Human gammaherpesvirus 4 isolate OSCC-57 EBER snRNA gene, complete sequence

AGGACAGCCGTTGCCCTAGTGGTTTCGGACACACCGCCAACGCTCAGTGCGGTGCTACCGACCCGAGGTCAAGTCCCGGGGGAGGAGAAGAGAGGCTTCCCG-CCTAGAGCATTTGCAAGTCAGGATTCTCTAATCCCTCTGGGAGAAGGGTATTCGGCTTGTCCGCTAT-TTTT

>MN921216 Human gammaherpesvirus 4 isolate OSCC-36 EBER snRNA gene, complete sequence

AGGACAGCCGTTGCCCTAGTGGTTTCGGACACACCGCCAACGCTCAGTGCGGTGCTACCGACCCGAGGTCAAGTCCCGGGGGAGGAGAAGAGAGGCTTCCCG-CCTAGAGCATTTGCAAGTCAGGATTCTCTAATCCCTCTGGGAGAAGGGTATTCGGCTTGTCCGCTAT-TTTT

>MN921217 Human gammaherpesvirus 4 isolate OSCC-237 EBER snRNA gene, complete sequence

AGGACAGCCGTTGCCCTAGTGGTTTCGGACACACCGCCAACGCTCAGTGCGGTGCTACCGACCCGAGGTCAAGTCCCGGGGGAGGAGAAGAGAGGCTTCCCG-CCTAGAGCATTTGCAAGTCAGGATTCTCTAATCCCTCTGGGAGAAGGGTATTCGGCTTGTCCGCTAT-TTTT

>MN921218 Human gammaherpesvirus 4 isolate OSCC-91 EBER snRNA gene, complete sequence

AGGACAGCCGTTGCCCTAGTGGTTTCGGACACACCGCCAACGCTCAGTGCGGTGCTACCGACCCGAGGTCAAGTCCCGGGGGAGGAGAAGAGAGGCTTCCCG-CCTAGAGCATTTGCAAGTCAGGATTCTCTAATCCCTCTGGGAGAAGGGTATTCGGCTTGTCCGCTAT-TTTT

>MN921219 Human gammaherpesvirus 4 isolate OLP-5 EBER snRNA gene, complete sequence

AGGACAGCCGTTGCCCTAGTGGTTTCGGACACACCGCCAACGCTCAGTGCGGTGCTACCGACCCGAGGTCAAGTCCCGGGGGAGGAGAAGAGAGGCTTCCCG-CCTAGAGCATTTGCAAGTCAGGATTCTCTAATCCCTCTGGGAGAAGGGTATTCGGCTTGTCCGCTAT-TTTT

>MZ337823 Human gammaherpesvirus 4 strain B95-8-LT

AGGACAGCCGTTGCCCTAGTGGTTTCGGACACACCGCCAACGCTCAGTGCGGTGCTACCGACCCGAGGTCAAGTCCCGGGGGAGGAGAAGAGAGGCTTCCCG-CCTAGAGCATTTGCAAGTCAGGATTCTCTAATCCCTCTGGGAGAAGGGTATTCGGCTTGTCCGCTAT-TTTT

>NC_007605 Human gammaherpesvirus 4, complete genome

AGGACAGCCGTTGCCCTAGTGGTTTCGGACACACCGCCAACGCTCAGTGCGGTGCTACCGACCCGAGGTCAAGTCCCGGGGGAGGAGAAGAGAGGCTTCCCG-CCTAGAGCATTTGCAAGTCAGGATTCTCTAATCCCTCTGGGAGAAGGGTATTCGGCTTGTCCGCTAT-TTTT

>V01555 Epstein-Barr virus (EBV) genome, strain B95-8

AGGACAGCCGTTGCCCTAGTGGTTTCGGACACACCGCCAACGCTCAGTGCGGTGCTACCGACCCGAGGTCAAGTCCCGGGGGAGGAGAAGAGAGGCTTCCCG-CCTAGAGCATTTGCAAGTCAGGATTCTCTAATCCCTCTGGGAGAAGGGTATTCGGCTTGTCCGCTAT-TTTT

>AB065136 Human herpesvirus 4 gene for EBER 2 small RNA, complete sequence

AGGACAGCCGTTGCCCTAGTGGTTTCGGACACACCGCCAACGCTCAGTGCGGTGCTACCGACCCGAGGTCAAGTCCCGGGGGAGGAGAAGAGAGGCTTCCCG-CCTAGAGCATTTGCAAGTCAGGATTCTCTAATCCCTCTGGGAGAAGGGTATTCGGCTTGTCCGCTGT-TTTT

>AB828191 Human herpesvirus 4 DNA, complete genome, strain: 1 LGY-Raji

AGGACAGCCGTTGCCCTAGTGGTTTCGGACACACCGCCAACGCTCAGTGCGGTGCTACCGACCCGAGGTCAAGTCCCGGGGGAGGAGAAGAGAGGCTTCCCG-CCTAGAGCATTTGCAAGTCAGGATTCTCTAATCCCTCTGGGAGAAGGGTATTCGGCTTGTCCGCTGT-TTTT

>AB850644 Human herpesvirus 4 DNA, complete genome, strain: HN2

AGGACAGCCGTTGCCCTAGTGGTTTCGGACACACCGCCAACGCTCAGTGCGGTGCTACCGACCCGAGGTCAAGTCCCGGGGGAGGAGAAGAGAGGCTTCCCG-CCTAGAGCATTTGCAAGTCAGGATTCTCTAATCCCTCTGGGAGAAGGGTATTCGGCTTGTCCGCTTT-TTTT

>AJ315772 Human herpesvirus type 4 partial proviral DNA for the EBER-1 and EBER-2 promoters, cell line Raji

AGGACAGCCGTTGCCCTAGTGGTTTCGGACACACCGCCAACGCTCAGTGCGGTGCTACCGACCCGAGGTCAAGTCCCGGGGGAGGAGAAGAGAGGCTTCCCG-CCTAGAGCATTTGCAAGTCAGGATTCTCTAATCCCTCTGGGAGAAGGGTATTCGGCTTGTCCGCTGT-TTTT

>AJ315773 Human herpesvirus type 4 partial proviral DNA for the EBER-1 and EBER-2 promoters, cell line Rael

AGGACAGCCGTTGCCCTAGTGGTTTCGGACACACCGCCAACGCTCAGTGCGGTGCTACCGACCCGAGGTCAAGTCCCGGGGGAGGAGAAGAGAGGCTTCCCG-CCTAGAGCATTTGCAAGTCAGGATTCTCTAATCCCTCTGGGAGAAGGGTATTCGGCTTGTCCGCTGT-TTTT

>AP015015 Human herpesvirus 4 DNA, complete genome, strain: SNU-719

AGGACAGCCGTTGCCCTAGTGGTTTCGGACACACCGCCAACGCTCAGTGCGGTGCTACCGACCCGAGGTCAAGTCCCGGGGGAGGAGAAGAGAGGCTTCCCG-CCTAGAGCATTTGCAAGTCAGGATTCTCTAATCCCTCTGGGAGAAGGGTATTCGGCTTGTCCGCTGT-TTTT

>AP019012 Human gammaherpesvirus 4 KAI3_cell DNA, nearly complete genome

AGGACAGCCGTTGCCCTAGTGGTTTCGGACACACCGCCAACGCTCAGTGCGGTGCTACCGACCCGAGGTCAAGTCCCGGGGGAGGAGAAGAGAGGCTTCCCG-CCTAGAGCATTTGCAAGTCAGGATTCTCTAATCCCTCTGGGAGAAGGGTATTCGGCTTGTCCGCTGT-TTTT

>AP019013 Human gammaherpesvirus 4 SNK1_cell DNA, complete genome

AGGACAGCCGTTGCCCTAGTGGTTTCGGACACACCGCCAACGCTCAGTGCGGTGCTACCGACCCGAGGTCAAGTCCCGGGGGAGGAGAAGAGAGGCTTCCCG-CCTAGAGCATTTGCAAGTCAGGATTCTCTAATCCCTCTGGGAGAAGGGTATTCGGCTTGTCCGCTGT-TTTT

>AP019014 Human gammaherpesvirus 4 SNK10_cell DNA, nearly complete genome

AGGACAGCCGTTGCCCTAGTGGTTTCGGACACACCGCCAACGCTCAGTGCGGTGCTACCGACCCGAGGTCAAGTCCCGGGGGAGGAGAAGAGAGGCTTCCCG-CCTAGAGCATTTGCAAGTCAGGATTCTCTAATCCCTCTGGGAGAAGGGTATTCGGCTTGTCCGCTGT-TTTT

>AP019015 Human gammaherpesvirus 4 SNT13_cell DNA, nearly complete genome

AGGACAGCCGTTGCCCTAGTGGTTTCGGACACACCGCCAACGCTCAGTGCGGTGCTACCGACCCGAGGTCAAGTCCCGGGGGAGGAGAAGAGAGGCTTCCCG-CCTAGAGCATTTGCAAGTCAGGATTCTCTAATCCCTCTGGGAGAAGGGTATTCGGCTTGTCCGCTGT-TTTT

>AP019017 Human gammaherpesvirus 4 SNT16_cell DNA, nearly complete genome

AGGACAGCCGTTGCCCTAGTGGTTTCGGACACACCGCCAACGCTCAGTGCGGTGCTACCGACCCGAGGTCAAGTCCCGGGGGAGGAGAAGAGAGGCTTCCCG-CCTAGAGCATTTGCAAGTCAGGATTCTCTAATCCCTCTGGGAGAAGGGTATTCGGCTTGTCCGCTGT-TTTT

>AP019018 Human gammaherpesvirus 4 SNT8_cell DNA, nearly complete genome

AGGACAGCCGTTGCCCTAGTGGTTTCGGACACACCGCCAACGCTCAGTGCGGTGCTACCGACCCGAGGTCAAGTCCCGGGGGAGGAGAAGAGAGGCTTCCCG-CCTAGAGCATTTGCAAGTCAGGATTCTCTAATCCCTCTGGGAGAAGGGTATTCGGCTTGTCCGCTGT-TTTT

>AP019019 Human gammaherpesvirus 4 UPN1002_PBMC DNA, nearly complete genome

AGGACAGCCGTTGCCCTAGTGGTTTCGGACACACCGCCAACGCTCAGTGCGGTGCTACCGACCCGAGGTCAAGTCCCGGGGGAGGAGAAGAGAGGCTTCCCG-CCTAGAGCATTTGCAAGTCAGGATTCTCTAATCCCTCTGGGAGAAGGGTATTCGGCTTGTCCGCTGT-TTTT

>AP019020 Human gammaherpesvirus 4 UPN1003_PBMC DNA, nearly complete genome

AGGACAGCCGTTGCCCTAGTGGTTTCGGACACACCGCCAACGCTCAGTGCGGTGCTACCGACCCGAGGTCAAGTCCCGGGGGAGGAGAAGAGAGGCTTCCCG-CCTAGAGCATTTGCAAGTCAGGATTCTCTAATCCCTCTGGGAGAAGGGTATTCGGCTTGTCCGCTGT-TTTT

>AP019021 Human gammaherpesvirus 4 UPN1004_PBMC DNA, nearly complete genome

AGGACAGCCGTTGCCCTAGTGGTTTCGGACACACCGCCAACGCTCAGTGCGGTGCTACCGACCCGAGGTCAAGTCCCGGGGGAGGAGAAGAGAGGCTTCCCG-CCTAGAGCATTTGCAAGTCAGGATTCTCTAATCCCTCTGGGAGAAGGGTATTCGGCTTGTCCGCTGT-TTTT

>AP019022 Human gammaherpesvirus 4 UPN1005_PBMC DNA, nearly complete genome

AGGACAGCCGTTGCCCTAGTGGTTTCGGACACACCGCCAACGCTCAGTGCGGTGCTACCGACCCGAGGTCAAGTCCCGGGGGAGGAGAAGAGAGGCTTCCCG-CCTAGAGCATTTGCAAGTCAGGATTCTCTAATCCCTCTGGGAGAAGGGTATTCGGCTTGTCCGCTGT-TTTT

>AP019024 Human gammaherpesvirus 4 UPN1008_PBMC DNA, nearly complete genome

AGGACAGCCGTTGCCCTAGTGGTTTCGGACACACCGCCAACGCTCAGTGCGGTGCTACCGACCCGAGGTCAAGTCCCGGGGGAGGAGAAGAGAGGCTTCCCG-CCTAGAGCATTTGCAAGTCAGGATTCTCTAATCCCTCTGGGAGAAGGGTATTCGGCTTGTCCGCTGT-TTTT

>AP019025 Human gammaherpesvirus 4 UPN102_PBMC DNA, nearly complete genome

AGGACAGCCGTTGCCCTAGTGGTTTCGGACACACCGCCAACGCTCAGTGCGGTGCTACCGACCCGAGGTCAAGTCCCGGGGGAGGAGAAGAGAGGCTTCCCG-CCTAGAGCATTTGCAAGTCAGGATTCTCTAATCCCTCTGGGAGAAGGGTATTCGGCTTGTCCGCTGT-TTTT

>AP019026 Human gammaherpesvirus 4 UPN104_PBMC DNA, nearly complete genome

AGGACAGCCGTTGCCCTAGTGGTTTCGGACACACCGCCAACGCTCAGTGCGGTGCTACCGACCCGAGGTCAAGTCCCGGGGGAGGAGAAGAGAGGCTTCCCG-CCTAGAGCATTTGCAAGTCAGGATTCTCTAATCCCTCTGGGAGAAGGGTATTCGGCTTGTCCGCTGT-TTTT

>AP019029 Human gammaherpesvirus 4 UPN106_PBMC DNA, nearly complete genome

AGGACAGCCGTTGCCCTAGTGGTTTCGGACACACCGCCAACGCTCAGTGCGGTGCTACCGACCCGAGGTCAAGTCCCGGGGGAGGAGAAGAGAGGCTTCCCG-CCTAGAGCATTTGCAAGTCAGGATTCTCTAATCCCTCTGGGAGAAGGGTATTCGGCTTGTCCGCTGT-TTTT

>AP019030 Human gammaherpesvirus 4 UPN107_PBMC DNA, nearly complete genome

AGGACAGCCGTTGCCCTAGTGGTTTCGGACACACCGCCAACGCTCAGTGCGGTGCTACCGACCCGAGGTCAAGTCCCGGGGGAGGAGAAGAGAGGCTTCCCG-CCTAGAGCATTTGCAAGTCAGGATTCTCTAATCCCTCTGGGAGAAGGGTATTCGGCTTGTCCGCTGT-TTTT

>AP019031 Human gammaherpesvirus 4 UPN108_PBMC DNA, nearly complete genome

AGGACAGCCGTTGCCCTAGTGGTTTCGGACACACCGCCAACGCTCAGTGCGGTGCTACCGACCCGAGGTCAAGTCCCGGGGGAGGAGAAGAGAGGCTTCCCG-CCTAGAGCATTTGCAAGTCAGGATTCTCTAATCCCTCTGGGAGAAGGGTATTCGGCTTGTCCGCTGT-TTTT

>AP019032 Human gammaherpesvirus 4 UPN108_tumor DNA, nearly complete genome

AGGACAGCCGTTGCCCTAGTGGTTTCGGACACACCGCCAACGCTCAGTGCGGTGCTACCGACCCGAGGTCAAGTCCCGGGGGAGGAGAAGAGAGGCTTCCCG-CCTAGAGCATTTGCAAGTCAGGATTCTCTAATCCCTCTGGGAGAAGGGTATTCGGCTTGTCCGCTGT-TTTT

>AP019033 Human gammaherpesvirus 4 UPN109_PBMC DNA, nearly complete genome

AGGACAGCCGTTGCCCTAGTGGTTTCGGACACACCGCCAACGCTCAGTGCGGTGCTACCGACCCGAGGTCAAGTCCCGGGGGAGGAGAAGAGAGGCTTCCCG-CCTAGAGCATTTGCAAGTCAGGATTCTCTAATCCCTCTGGGAGAAGGGTATTCGGCTTGTCCGCTGT-TTTT

>AP019034 Human gammaherpesvirus 4 UPN110_PBMC DNA, nearly complete genome

AGGACAGCCGTTGCCCTAGTGGTTTCGGACACACCGCCAACGCTCAGTGCGGTGCTACCGACCCGAGGTCAAGTCCCGGGGGAGGAGAAGAGAGGCTTCCCG-CCTAGAGCATTTGCAAGTCAGGATTCTCTAATCCCTCTGGGAGAAGGGTATTCGGCTTGTCCGCTGT-TTTT

>AP019035 Human gammaherpesvirus 4 UPN1101_PBMC DNA, nearly complete genome

AGGACAGCCGTTGCCCTAGTGGTTTCGGACACACCGCCAACGCTCAGTGCGGTGCTACCGACCCGAGGTCAAGTCCCGGGGGAGGAGAAGAGAGGCTTCCCG-CCTAGAGCATTTGCAAGTCAGGATTCTCTAATCCCTCTGGGAGAAGGGTATTCGGCTTGTCCGCTGT-TTTT

>AP019036 Human gammaherpesvirus 4 UPN1102_PBMC DNA, nearly complete genome

AGGACAGCCGTTGCCCTAGTGGTTTCGGACACACCGCCAACGCTCAGTGCGGTGCTACCGACCCGAGGTCAAGTCCCGGGGGAGGAGAAGAGAGGCTTCCCG-CCTAGAGCATTTGCAAGTCAGGATTCTCTAATCCCTCTGGGAGAAGGGTATTCGGCTTGTCCGCTGT-TTTT

>AP019037 Human gammaherpesvirus 4 UPN1104_PBMC DNA, nearly complete genome

AGGACAGCCGTTGCCCTAGTGGTTTCGGACACACCGCCAACGCTCAGTGCGGTGCTACCGACCCGAGGTCAAGTCCCGGGGGAGGAGAAGAGAGGCTTCCCG-CCTAGAGCATTTGCAAGTCAGGATTCTCTAATCCCTCTGGGAGAAGGGTATTCGGCTTGTCCGCTGT-TTTT

>AP019038 Human gammaherpesvirus 4 UPN1106_PBMC DNA, nearly complete genome

AGGACAGCCGTTGCCCTAGTGGTTTCGGACACACCGCCAACGCTCAGTGCGGTGCTACCGACCCGAGGTCAAGTCCCGGGGGAGGAGAAGAGAGGCTTCCCG-CCTAGAGCATTTGCAAGTCAGGATTCTCTAATCCCTCTGGGAGAAGGGTATTCGGCTTGTCCGCTGT-TTTT

>AP019039 Human gammaherpesvirus 4 UPN111_PBMC DNA, nearly complete genome

AGGACAGCCGTTGCCCTAGTGGTTTCGGACACACCGCCAACGCTCAGTGCGGTGCTACCGACCCGAGGTCAAGTCCCGGGGGAGGAGAAGAGAGGCTTCCCG-CCTAGAGCATTTGCAAGTCAGGATTCTCTAATCCCTCTGGGAGAAGGGTATTCGGCTTGTCCGCTGT-TTTT

>AP019040 Human gammaherpesvirus 4 UPN1110_PBMC DNA, complete genome

AGGACAGCCGTTGCCCTAGTGGTTTCGGACACACCGCCAACGCTCAGTGCGGTGCTACCGACCCGAGGTCAAGTCCCGGGGGAGGAGAAGAGAGGCTTCCCG-CCTAGAGCATTTGCAAGTCAGGATTCTCTAATCCCTCTGGGAGAAGGGTATTCGGCTTGTCCGCTGT-TTTT

>AP019045 Human gammaherpesvirus 4 UPN1120_PBMC DNA, nearly complete genome

AGGACAGCCGTTGCCCTAGTGGTTTCGGACACACCGCCAACGCTCAGTGCGGTGCTACCGACCCGAGGTCAAGTCCCGGGGGAGGAGAAGAGAGGCTTCCCG-CCTAGAGCATTTGCAAGTCAGGATTCTCTAATCCCTCTGGGAGAAGGGTATTCGGCTTGTCCGCTGT-TTTT

>AP019046 Human gammaherpesvirus 4 UPN1124_PBMC DNA, nearly complete genome

AGGACAGCCGTTGCCCTAGTGGTTTCGGACACACCGCCAACGCTCAGTGCGGTGCTACCGACCCGAGGTCAAGTCCCGGGGGAGGAGAAGAGAGGCTTCCCG-CCTAGAGCATTTGCAAGTCAGGATTCTCTAATCCCTCTGGGAGAAGGGTATTCGGCTTGTCCGCTGT-TTTT

>AP019047 Human gammaherpesvirus 4 UPN113_PBMC DNA, nearly complete genome

AGGACAGCCGTTGCCCTAGTGGTTTCGGACACACCGCCAACGCTCAGTGCGGTGCTACCGACCCGAGGTCAAGTCCCGGGGGAGGAGAAGAGAGGCTTCCCG-CCTAGAGCATTTGCAAGTCAGGATTCTCTAATCCCTCTGGGAGAAGGGTATTCGGCTTGTCCGCTGT-TTTT

>AP019048 Human gammaherpesvirus 4 UPN114_PBMC DNA, nearly complete genome

AGGACAGCCGTTGCCCTAGTGGTTTCGGACACACCGCCAACGCTCAGTGCGGTGCTACCGACCCGAGGTCAAGTCCCGGGGGAGGAGAAGAGAGGCTTCCCG-CCTAGAGCATTTGCAAGTCAGGATTCTCTAATCCCTCTGGGAGAAGGGTATTCGGCTTGTCCGCTGT-TTTT

>AP019049 Human gammaherpesvirus 4 UPN114_tumor DNA, nearly complete genome

AGGACAGCCGTTGCCCTAGTGGTTTCGGACACACCGCCAACGCTCAGTGCGGTGCTACCGACCCGAGGTCAAGTCCCGGGGGAGGAGAAGAGAGGCTTCCCG-CCTAGAGCATTTGCAAGTCAGGATTCTCTAATCCCTCTGGGAGAAGGGTATTCGGCTTGTCCGCTGT-TTTT

>AP019051 Human gammaherpesvirus 4 UPN1201_PBMC DNA, nearly complete genome

AGGACAGCCGTTGCCCTAGTGGTTTCGGACACACCGCCAACGCTCAGTGCGGTGCTACCGACCCGAGGTCAAGTCCCGGGGGAGGAGAAGAGAGGCTTCCCG-CCTAGAGCATTTGCAAGTCAGGATTCTCTAATCCCTCTGGGAGAAGGGTATTCGGCTTGTCCGCTGT-TTTT

>AP019053 Human gammaherpesvirus 4 UPN123_tumor DNA, nearly complete genome

AGGACAGCCGTTGCCCTAGTGGTTTCGGACACACCGCCAACGCTCAGTGCGGTGCTACCGACCCGAGGTCAAGTCCCGGGGGAGGAGAAGAGAGGCTTCCCG-CCTAGAGCATTTGCAAGTCAGGATTCTCTAATCCCTCTGGGAGAAGGGTATTCGGCTTGTCCGCTGT-TTTT

>AP019054 Human gammaherpesvirus 4 UPN125_PBMC DNA, nearly complete genome

AGGACAGCCGTTGCCCTAGTGGTTTCGGACACACCGCCAACGCTCAGTGCGGTGCTACCGACCCGAGGTCAAGTCCCGGGGGAGGAGAAGAGAGGCTTCCCG-CCTAGAGCATTTGCAAGTCAGGATTCTCTAATCCCTCTGGGAGAAGGGTATTCGGCTTGTCCGCTGT-TTTT

>AP019055 Human gammaherpesvirus 4 UPN128_PBMC DNA, nearly complete genome

AGGACAGCCGTTGCCCTAGTGGTTTCGGACACACCGCCAACGCTCAGTGCGGTGCTACCGACCCGAGGTCAAGTCCCGGGGGAGGAGAAGAGAGGCTTCCCG-CCTAGAGCATTTGCAAGTCAGGATTCTCTAATCCCTCTGGGAGAAGGGTATTCGGCTTGTCCGCTGT-TTTT

>AP019056 Human gammaherpesvirus 4 UPN128_tumor DNA, nearly complete genome

AGGACAGCCGTTGCCCTAGTGGTTTCGGACACACCGCCAACGCTCAGTGCGGTGCTACCGACCCGAGGTCAAGTCCCGGGGGAGGAGAAGAGAGGCTTCCCG-CCTAGAGCATTTGCAAGTCAGGATTCTCTAATCCCTCTGGGAGAAGGGTATTCGGCTTGTCCGCTGT-TTTT

>AP019057 Human gammaherpesvirus 4 UPN13_PBMC DNA, nearly complete genome

AGGACAGCCGTTGCCCTAGTGGTTTCGGACACACCGCCAACGCTCAGTGCGGTGCTACCGACCCGAGGTCAAGTCCCGGGGGAGGAGAAGAGAGGCTTCCCG-CCTAGAGCATTTGCAAGTCAGGATTCTCTAATCCCTCTGGGAGAAGGGTATTCGGCTTGTCCGCTGT-TTTT

>AP019058 Human gammaherpesvirus 4 UPN130_PBMC DNA, nearly complete genome

AGGACAGCCGTTGCCCTAGTGGTTTCGGACACACCGCCAACGCTCAGTGCGGTGCTACCGACCCGAGGTCAAGTCCCGGGGGAGGAGAAGAGAGGCTTCCCG-CCTAGAGCATTTGCAAGTCAGGATTCTCTAATCCCTCTGGGAGAAGGGTATTCGGCTTGTCCGCTGT-TTTT

>AP019059 Human gammaherpesvirus 4 UPN130_tumor DNA, nearly complete genome

AGGACAGCCGTTGCCCTAGTGGTTTCGGACACACCGCCAACGCTCAGTGCGGTGCTACCGACCCGAGGTCAAGTCCCGGGGGAGGAGAAGAGAGGCTTCCCG-CCTAGAGCATTTGCAAGTCAGGATTCTCTAATCCCTCTGGGAGAAGGGTATTCGGCTTGTCCGCTGT-TTTT

>AP019060 Human gammaherpesvirus 4 UPN132_PBMC DNA, nearly complete genome

AGGACAGCCGTTGCCCTAGTGGTTTCGGACACACCGCCAACGCTCAGTGCGGTGCTACCGACCCGAGGTCAAGTCCCGGGGGAGGAGAAGAGAGGCTTCCCG-CCTAGAGCATTTGCAAGTCAGGATTCTCTAATCCCTCTGGGAGAAGGGTATTCGGCTTGTCCGCTGT-TTTT

>AP019061 Human gammaherpesvirus 4 UPN132_tumor DNA, complete genome

AGGACAGCCGTTGCCCTAGTGGTTTCGGACACACCGCCAACGCTCAGTGCGGTGCTACCGACCCGAGGTCAAGTCCCGGGGGAGGAGAAGAGAGGCTTCCCG-CCTAGAGCATTTGCAAGTCAGGATTCTCTAATCCCTCTGGGAGAAGGGTATTCGGCTTGTCCGCTGT-TTTT

>AP019062 Human gammaherpesvirus 4 UPN134_PBMC DNA, nearly complete genome

AGGACAGCCGTTGCCCTAGTGGTTTCGGACACACCGCCAACGCTCAGTGCGGTGCTACCGACCCGAGGTCAAGTCCCGGGGGAGGAGAAGAGAGGCTTCCCG-CCTAGAGCATTTGCAAGTCAGGATTCTCTAATCCCTCTGGGAGAAGGGTATTCGGCTTGTCCGCTGT-TTTT

>AP019063 Human gammaherpesvirus 4 UPN134_tumor DNA, nearly complete genome

AGGACAGCCGTTGCCCTAGTGGTTTCGGACACACCGCCAACGCTCAGTGCGGTGCTACCGACCCGAGGTCAAGTCCCGGGGGAGGAGAAGAGAGGCTTCCCG-CCTAGAGCATTTGCAAGTCAGGATTCTCTAATCCCTCTGGGAGAAGGGTATTCGGCTTGTCCGCTGT-TTTT

>AP019064 Human gammaherpesvirus 4 UPN136_PBMC DNA, nearly complete genome

AGGACAGCCGTTGCCCTAGTGGTTTCGGACACACCGCCAACGCTCAGTGCGGTGCTACCGACCCGAGGTCAAGTCCCGGGGGAGGAGAAGAGAGGCTTCCCG-CCTAGAGCATTTGCAAGTCAGGATTCTCTAATCCCTCTGGGAGAAGGGTATTCGGCTTGTCCGCTGT-TTTT

>AP019065 Human gammaherpesvirus 4 UPN136_tumor DNA, nearly complete genome

AGGACAGCCGTTGCCCTAGTGGTTTCGGACACACCGCCAACGCTCAGTGCGGTGCTACCGACCCGAGGTCAAGTCCCGGGGGAGGAGAAGAGAGGCTTCCCG-CCTAGAGCATTTGCAAGTCAGGATTCTCTAATCCCTCTGGGAGAAGGGTATTCGGCTTGTCCGCTGT-TTTT

>AP019066 Human gammaherpesvirus 4 UPN138_PBMC DNA, nearly complete genome

AGGACAGCCGTTGCCCTAGTGGTTTCGGACACACCGCCAACGCTCAGTGCGGTGCTACCGACCCGAGGTCAAGTCCCGGGGGAGGAGAAGAGAGGCTTCCCG-CCTAGAGCATTTGCAAGTCAGGATTCTCTAATCCCTCTGGGAGAAGGGTATTCGGCTTGTCCGCTGT-TTTT

>AP019067 Human gammaherpesvirus 4 UPN138_tumor DNA, nearly complete genome

AGGACAGCCGTTGCCCTAGTGGTTTCGGACACACCGCCAACGCTCAGTGCGGTGCTACCGACCCGAGGTCAAGTCCCGGGGGAGGAGAAGAGAGGCTTCCCG-CCTAGAGCATTTGCAAGTCAGGATTCTCTAATCCCTCTGGGAGAAGGGTATTCGGCTTGTCCGCTGT-TTTT

>AP019068 Human gammaherpesvirus 4 UPN14_PBMC DNA, nearly complete genome

AGGACAGCCGTTGCCCTAGTGGTTTCGGACACACCGCCAACGCTCAGTGCGGTGCTACCGACCCGAGGTCAAGTCCCGGGGGAGGAGAAGAGAGGCTTCCCG-CCTAGAGCATTTGCAAGTCAGGATTCTCTAATCCCTCTGGGAGAAGGGTATTCGGCTTGTCCGCTGT-TTTT

>AP019069 Human gammaherpesvirus 4 UPN140_PBMC DNA, nearly complete genome

AGGACAGCCGTTGCCCTAGTGGTTTCGGACACACCGCCAACGCTCAGTGCGGTGCTACCGACCCGAGGTCAAGTCCCGGGGGAGGAGAAGAGAGGCTTCCCG-CCTAGAGCATTTGCAAGTCAGGATTCTCTAATCCCTCTGGGAGAAGGGTATTCGGCTTGTCCGCTGT-TTTT

>AP019070 Human gammaherpesvirus 4 UPN140_tumor DNA, nearly complete genome

AGGACAGCCGTTGCCCTAGTGGTTTCGGACACACCGCCAACGCTCAGTGCGGTGCTACCGACCCGAGGTCAAGTCCCGGGGGAGGAGAAGAGAGGCTTCCCG-CCTAGAGCATTTGCAAGTCAGGATTCTCTAATCCCTCTGGGAGAAGGGTATTCGGCTTGTCCGCTGT-TTTT

>AP019071 Human gammaherpesvirus 4 UPN142_gdT-1 DNA, nearly complete genome

AGGACAGCCGTTGCCCTAGTGGTTTCGGACACACCGCCAACGCTCAGTGCGGTGCTACCGACCCGAGGTCAAGTCCCGGGGGAGGAGAAGAGAGGCTTCCCG-CCTAGAGCATTTGCAAGTCAGGATTCTCTAATCCCTCTGGGAGAAGGGTATTCGGCTTGTCCGCTGT-TTTT

>AP019072 Human gammaherpesvirus 4 UPN142_gdT-2 DNA, nearly complete genome

AGGACAGCCGTTGCCCTAGTGGTTTCGGACACACCGCCAACGCTCAGTGCGGTGCTACCGACCCGAGGTCAAGTCCCGGGGGAGGAGAAGAGAGGCTTCCCG-CCTAGAGCATTTGCAAGTCAGGATTCTCTAATCCCTCTGGGAGAAGGGTATTCGGCTTGTCCGCTGT-TTTT

>AP019073 Human gammaherpesvirus 4 UPN142_PBMC DNA, nearly complete genome

AGGACAGCCGTTGCCCTAGTGGTTTCGGACACACCGCCAACGCTCAGTGCGGTGCTACCGACCCGAGGTCAAGTCCCGGGGGAGGAGAAGAGAGGCTTCCCG-CCTAGAGCATTTGCAAGTCAGGATTCTCTAATCCCTCTGGGAGAAGGGTATTCGGCTTGTCCGCTGT-TTTT

>AP019074 Human gammaherpesvirus 4 UPN15_PBMC DNA, nearly complete genome

AGGACAGCCGTTGCCCTAGTGGTTTCGGACACACCGCCAACGCTCAGTGCGGTGCTACCGACCCGAGGTCAAGTCCCGGGGGAGGAGAAGAGAGGCTTCCCG-CCTAGAGCATTTGCAAGTCAGGATTCTCTAATCCCTCTGGGAGAAGGGTATTCGGCTTGTCCGCTGT-TTTT

>AP019075 Human gammaherpesvirus 4 UPN17_PBMC DNA, nearly complete genome

AGGACAGCCGTTGCCCTAGTGGTTTCGGACACACCGCCAACGCTCAGTGCGGTGCTACCGACCCGAGGTCAAGTCCCGGGGGAGGAGAAGAGAGGCTTCCCG-CCTAGAGCATTTGCAAGTCAGGATTCTCTAATCCCTCTGGGAGAAGGGTATTCGGCTTGTCCGCTGT-TTTT

>AP019076 Human gammaherpesvirus 4 UPN1756_PBMC DNA, complete genome

AGGACAGCCGTTGCCCTAGTGGTTTCGGACACACCGCCAACGCTCAGTGCGGTGCTACCGACCCGAGGTCAAGTCCCGGGGGAGGAGAAGAGAGGCTTCCCG-CCTAGAGCATTTGCAAGTCAGGATTCTCTAATCCCTCTGGGAGAAGGGTATTCGGCTTGTCCGCTGT-TTTT

>AP019077 Human gammaherpesvirus 4 UPN1757_PBMC DNA, nearly complete genome

AGGACAGCCGTTGCCCTAGTGGTTTCGGACACACCGCCAACGCTCAGTGCGGTGCTACCGACCCGAGGTCAAGTCCCGGGGGAGGAGAAGAGAGGCTTCCCG-CCTAGAGCATTTGCAAGTCAGGATTCTCTAATCCCTCTGGGAGAAGGGTATTCGGCTTGTCCGCTGT-TTTT

>AP019079 Human gammaherpesvirus 4 UPN1789_PBMC DNA, nearly complete genome

AGGACAGCCGTTGCCCTAGTGGTTTCGGACACACCGCCAACGCTCAGTGCGGTGCTACCGACCCGAGGTCAAGTCCCGGGGGAGGAGAAGAGAGGCTTCCCG-CCTAGAGCATTTGCAAGTCAGGATTCTCTAATCCCTCTGGGAGAAGGGTATTCGGCTTGTCCGCTGT-TTTT

>AP019080 Human gammaherpesvirus 4 UPN18_PBMC DNA, nearly complete genome

AGGACAGCCGTTGCCCTAGTGGTTTCGGACACACCGCCAACGCTCAGTGCGGTGCTACCGACCCGAGGTCAAGTCCCGGGGGAGGAGAAGAGAGGCTTCCCG-CCTAGAGCATTTGCAAGTCAGGATTCTCTAATCCCTCTGGGAGAAGGGTATTCGGCTTGTCCGCTGT-TTTT

>AP019081 Human gammaherpesvirus 4 UPN1802_PBMC DNA, nearly complete genome

AGGACAGCCGTTGCCCTAGTGGTTTCGGACACACCGCCAACGCTCAGTGCGGTGCTACCGACCCGAGGTCAAGTCCCGGGGGAGGAGAAGAGAGGCTTCCCG-CCTAGAGCATTTGCAAGTCAGGATTCTCTAATCCCTCTGGGAGAAGGGTATTCGGCTTGTCCGCTGT-TTTT

>AP019082 Human gammaherpesvirus 4 UPN1813_PBMC DNA, nearly complete genome

AGGACAGCCGTTGCCCTAGTGGTTTCGGACACACCGCCAACGCTCAGTGCGGTGCTACCGACCCGAGGTCAAGTCCCGGGGGAGGAGAAGAGAGGCTTCCCG-CCTAGAGCATTTGCAAGTCAGGATTCTCTAATCCCTCTGGGAGAAGGGTATTCGGCTTGTCCGCTGT-TTTT

>AP019083 Human gammaherpesvirus 4 UPN1833_tissue DNA, complete genome

AGGACAGCCGTTGCCCTAGTGGTTTCGGACACACCGCCAACGCTCAGTGCGGTGCTACCGACCCGAGGTCAAGTCCCGGGGGAGGAGAAGAGAGGCTTCCCG-CCTAGAGCATTTGCAAGTCAGGATTCTCTAATCCCTCTGGGAGAAGGGTATTCGGCTTGTCCGCTGT-TTTT

>AP019084 Human gammaherpesvirus 4 UPN185_PBMC DNA, nearly complete genome

AGGACAGCCGTTGCCCTAGTGGTTTCGGACACACCGCCAACGCTCAGTGCGGTGCTACCGACCCGAGGTCAAGTCCCGGGGGAGGAGAAGAGAGGCTTCCCG-CCTAGAGCATTTGCAAGTCAGGATTCTCTAATCCCTCTGGGAGAAGGGTATTCGGCTTGTCCGCTGT-TTTT

>AP019085 Human gammaherpesvirus 4 UPN19_PBMC DNA, nearly complete genome

AGGACAGCCGTTGCCCTAGTGGTTTCGGACACACCGCCAACGCTCAGTGCGGTGCTACCGACCCGAGGTCAAGTCCCGGGGGAGGAGAAGAGAGGCTTCCCG-CCTAGAGCATTTGCAAGTCAGGATTCTCTAATCCCTCTGGGAGAAGGGTATTCGGCTTGTCCGCTGT-TTTT

>AP019086 Human gammaherpesvirus 4 UPN1901_tissue DNA, nearly complete genome

AGGACAGCCGTTGCCCTAGTGGTTTCGGACACACCGCCAACGCTCAGTGCGGTGCTACCGACCCGAGGTCAAGTCCCGGGGGAGGAGAAGAGAGGCTTCCCG-CCTAGAGCATTTGCAAGTCAGGATTCTCTAATCCCTCTGGGAGAAGGGTATTCGGCTTGTCCGCTGT-TTTT

>AP019088 Human gammaherpesvirus 4 UPN2_PBMC DNA, nearly complete genome

AGGACAGCCGTTGCCCTAGTGGTTTCGGACACACCGCCAACGCTCAGTGCGGTGCTACCGACCCGAGGTCAAGTCCCGGGGGAGGAGAAGAGAGGCTTCCCG-CCTAGAGCATTTGCAAGTCAGGATTCTCTAATCCCTCTGGGAGAAGGGTATTCGGCTTGTCCGCTGT-TTTT

>AP019089 Human gammaherpesvirus 4 UPN20_PBMC DNA, nearly complete genome

AGGACAGCCGTTGCCCTAGTGGTTTCGGACACACCGCCAACGCTCAGTGCGGTGCTACCGACCCGAGGTCAAGTCCCGGGGGAGGAGAAGAGAGGCTTCCCG-CCTAGAGCATTTGCAAGTCAGGATTCTCTAATCCCTCTGGGAGAAGGGTATTCGGCTTGTCCGCTGT-TTTT

>AP019090 Human gammaherpesvirus 4 UPN21_PBMC DNA, nearly complete genome

AGGACAGCCGTTGCCCTAGTGGTTTCGGACACACCGCCAACGCTCAGTGCGGTGCTACCGACCCGAGGTCAAGTCCCGGGGGAGGAGAAGAGAGGCTTCCCG-CCTAGAGCATTTGCAAGTCAGGATTCTCTAATCCCTCTGGGAGAAGGGTATTCGGCTTGTCCGCTGT-TTTT

>AP019091 Human gammaherpesvirus 4 UPN22_PBMC DNA, nearly complete genome

AGGACAGCCGTTGCCCTAGTGGTTTCGGACACACCGCCAACGCTCAGTGCGGTGCTACCGACCCGAGGTCAAGTCCCGGGGGAGGAGAAGAGAGGCTTCCCG-CCTAGAGCATTTGCAAGTCAGGATTCTCTAATCCCTCTGGGAGAAGGGTATTCGGCTTGTCCGCTGT-TTTT

>AP019092 Human gammaherpesvirus 4 UPN23_PBMC DNA, nearly complete genome

AGGACAGCCGTTGCCCTAGTGGTTTCGGACACACCGCCAACGCTCAGTGCGGTGCTACCGACCCGAGGTCAAGTCCCGGGGGAGGAGAAGAGAGGCTTCCCG-CCTAGAGCATTTGCAAGTCAGGATTCTCTAATCCCTCTGGGAGAAGGGTATTCGGCTTGTCCGCTGT-TTTT

>AP019093 Human gammaherpesvirus 4 UPN2329_PBMC DNA, nearly complete genome

AGGACAGCCGTTGCCCTAGTGGTTTCGGACACACCGCCAACGCTCAGTGCGGTGCTACCGACCCGAGGTCAAGTCCCGGGGGAGGAGAAGAGAGGCTTCCCG-CCTAGAGCATTTGCAAGTCAGGATTCTCTAATCCCTCTGGGAGAAGGGTATTCGGCTTGTCCGCTGT-TTTT

>AP019094 Human gammaherpesvirus 4 UPN24_PBMC DNA, nearly complete genome

AGGACAGCCGTTGCCCTAGTGGTTTCGGACACACCGCCAACGCTCAGTGCGGTGCTACCGACCCGAGGTCAAGTCCCGGGGGAGGAGAAGAGAGGCTTCCCG-CCTAGAGCATTTGCAAGTCAGGATTCTCTAATCCCTCTGGGAGAAGGGTATTCGGCTTGTCCGCTGT-TTTT

>AP019095 Human gammaherpesvirus 4 UPN25_PBMC DNA, nearly complete genome

AGGACAGCCGTTGCCCTAGTGGTTTCGGACACACCGCCAACGCTCAGTGCGGTGCTACCGACCCGAGGTCAAGTCCCGGGGGAGGAGAAGAGAGGCTTCCCG-CCTAGAGCATTTGCAAGTCAGGATTCTCTAATCCCTCTGGGAGAAGGGTATTCGGCTTGTCCGCTGT-TTTT

>AP019096 Human gammaherpesvirus 4 UPN251_PBMC DNA, complete genome

AGGACAGCCGTTGCCCTAGTGGTTTCGGACACACCGCCAACGCTCAGTGCGGTGCTACCGACCCGAGGTCAAGTCCCGGGGGAGGAGAAGAGAGGCTTCCCG-CCTAGAGCATTTGCAAGTCAGGATTCTCTAATCCCTCTGGGAGAAGGGTATTCGGCTTGTCCGCTGT-TTTT

>AP019097 Human gammaherpesvirus 4 UPN252_PBMC DNA, nearly complete genome

AGGACAGCCGTTGCCCTAGTGGTTTCGGACACACCGCCAACGCTCAGTGCGGTGCTACCGACCCGAGGTCAAGTCCCGGGGGAGGAGAAGAGAGGCTTCCCG-CCTAGAGCATTTGCAAGTCAGGATTCTCTAATCCCTCTGGGAGAAGGGTATTCGGCTTGTCCGCTGT-TTTT

>AP019098 Human gammaherpesvirus 4 UPN253_PBMC DNA, nearly complete genome

AGGACAGCCGTTGCCCTAGTGGTTTCGGACACACCGCCAACGCTCAGTGCGGTGCTACCGACCCGAGGTCAAGTCCCGGGGGAGGAGAAGAGAGGCTTCCCG-CCTAGAGCATTTGCAAGTCAGGATTCTCTAATCCCTCTGGGAGAAGGGTATTCGGCTTGTCCGCTGT-TTTT

>AP019099 Human gammaherpesvirus 4 UPN254_PBMC DNA, nearly complete genome

AGGACAGCCGTTGCCCTAGTGGTTTCGGACACACCGCCAACGCTCAGTGCGGTGCTACCGACCCGAGGTCAAGTCCCGGGGGAGGAGAAGAGAGGCTTCCCG-CCTAGAGCATTTGCAAGTCAGGATTCTCTAATCCCTCTGGGAGAAGGGTATTCGGCTTGTCCGCTGT-TTTT

>AP019100 Human gammaherpesvirus 4 UPN255_PBMC DNA, nearly complete genome

AGGACAGCCGTTGCCCTAGTGGTTTCGGACACACCGCCAACGCTCAGTGCGGTGCTACCGACCCGAGGTCAAGTCCCGGGGGAGGAGAAGAGAGGCTTCCCG-CCTAGAGCATTTGCAAGTCAGGATTCTCTAATCCCTCTGGGAGAAGGGTATTCGGCTTGTCCGCTGT-TTTT

>AP019102 Human gammaherpesvirus 4 UPN257_PBMC DNA, complete genome

AGGACAGCCGTTGCCCTAGTGGTTTCGGACACACCGCCAACGCTCAGTGCGGTGCTACCGACCCGAGGTCAAGTCCCGGGGGAGGAGAAGAGAGGCTTCCCG-CCTAGAGCATTTGCAAGTCAGGATTCTCTAATCCCTCTGGGAGAAGGGTATTCGGCTTGTCCGCTGT-TTTT

>AP019104 Human gammaherpesvirus 4 UPN259_PBMC DNA, nearly complete genome

AGGACAGCCGTTGCCCTAGTGGTTTCGGACACACCGCCAACGCTCAGTGCGGTGCTACCGACCCGAGGTCAAGTCCCGGGGGAGGAGAAGAGAGGCTTCCCG-CCTAGAGCATTTGCAAGTCAGGATTCTCTAATCCCTCTGGGAGAAGGGTATTCGGCTTGTCCGCTGT-TTTT

>AP019105 Human gammaherpesvirus 4 UPN26_PBMC DNA, nearly complete genome

AGGACAGCCGTTGCCCTAGTGGTTTCGGACACACCGCCAACGCTCAGTGCGGTGCTACCGACCCGAGGTCAAGTCCCGGGGGAGGAGAAGAGAGGCTTCCCG-CCTAGAGCATTTGCAAGTCAGGATTCTCTAATCCCTCTGGGAGAAGGGTATTCGGCTTGTCCGCTGT-TTTT

>AP019107 Human gammaherpesvirus 4 UPN261_PBMC DNA, nearly complete genome

AGGACAGCCGTTGCCCTAGTGGTTTCGGACACACCGCCAACGCTCAGTGCGGTGCTACCGACCCGAGGTCAAGTCCCGGGGGAGGAGAAGAGAGGCTTCCCG-CCTAGAGCATTTGCAAGTCAGGATTCTCTAATCCCTCTGGGAGAAGGGTATTCGGCTTGTCCGCTGT-TTTT

>AP019108 Human gammaherpesvirus 4 UPN262_PBMC DNA, nearly complete genome

AGGACAGCCGTTGCCCTAGTGGTTTCGGACACACCGCCAACGCTCAGTGCGGTGCTACCGACCCGAGGTCAAGTCCCGGGGGAGGAGAAGAGAGGCTTCCCG-CCTAGAGCATTTGCAAGTCAGGATTCTCTAATCCCTCTGGGAGAAGGGTATTCGGCTTGTCCGCTGT-TTTT

>AP019109 Human gammaherpesvirus 4 UPN263_PBMC DNA, nearly complete genome

AGGACAGCCGTTGCCCTAGTGGTTTCGGACACACCGCCAACGCTCAGTGCGGTGCTACCGACCCGAGGTCAAGTCCCGGGGGAGGAGAAGAGAGGCTTCCCG-CCTAGAGCATTTGCAAGTCAGGATTCTCTAATCCCTCTGGGAGAAGGGTATTCGGCTTGTCCGCTGT-TTTT

>AP019110 Human gammaherpesvirus 4 UPN264_PBMC DNA, nearly complete genome

AGGACAGCCGTTGCCCTAGTGGTTTCGGACACACCGCCAACGCTCAGTGCGGTGCTACCGACCCGAGGTCAAGTCCCGGGGGAGGAGAAGAGAGGCTTCCCG-CCTAGAGCATTTGCAAGTCAGGATTCTCTAATCCCTCTGGGAGAAGGGTATTCGGCTTGTCCGCTGT-TTTT

>AP019111 Human gammaherpesvirus 4 UPN265_PBMC DNA, nearly complete genome

AGGACAGCCGTTGCCCTAGTGGTTTCGGACACACCGCCAACGCTCAGTGCGGTGCTACCGACCCGAGGTCAAGTCCCGGGGGAGGAGAAGAGAGGCTTCCCG-CCTAGAGCATTTGCAAGTCAGGATTCTCTAATCCCTCTGGGAGAAGGGTATTCGGCTTGTCCGCTGT-TTTT

>AP019112 Human gammaherpesvirus 4 UPN2654_PBMC DNA, complete genome

AGGACAGCCGTTGCCCTAGTGGTTTCGGACACACCGCCAACGCTCAGTGCGGTGCTACCGACCCGAGGTCAAGTCCCGGGGGAGGAGAAGAGAGGCTTCCCG-CCTAGAGCATTTGCAAGTCAGGATTCTCTAATCCCTCTGGGAGAAGGGTATTCGGCTTGTCCGCTGT-TTTT

>AP019113 Human gammaherpesvirus 4 UPN266_PBMC DNA, nearly complete genome

AGGACAGCCGTTGCCCTAGTGGTTTCGGACACACCGCCAACGCTCAGTGCGGTGCTACCGACCCGAGGTCAAGTCCCGGGGGAGGAGAAGAGAGGCTTCCCG-CCTAGAGCATTTGCAAGTCAGGATTCTCTAATCCCTCTGGGAGAAGGGTATTCGGCTTGTCCGCTGT-TTTT

>AP019115 Human gammaherpesvirus 4 UPN27_PBMC DNA, nearly complete genome

AGGACAGCCGTTGCCCTAGTGGTTTCGGACACACCGCCAACGCTCAGTGCGGTGCTACCGACCCGAGGTCAAGTCCCGGGGGAGGAGAAGAGAGGCTTCCCG-CCTAGAGCATTTGCAAGTCAGGATTCTCTAATCCCTCTGGGAGAAGGGTATTCGGCTTGTCCGCTGT-TTTT

>AP019117 Human gammaherpesvirus 4 UPN28_PBMC DNA, nearly complete genome

AGGACAGCCGTTGCCCTAGTGGTTTCGGACACACCGCCAACGCTCAGTGCGGTGCTACCGACCCGAGGTCAAGTCCCGGGGGAGGAGAAGAGAGGCTTCCCG-CCTAGAGCATTTGCAAGTCAGGATTCTCTAATCCCTCTGGGAGAAGGGTATTCGGCTTGTCCGCTGT-TTTT

>AP019118 Human gammaherpesvirus 4 UPN301_CD56+ DNA, nearly complete genome

AGGACAGCCGTTGCCCTAGTGGTTTCGGACACACCGCCAACGCTCAGTGCGGTGCTACCGACCCGAGGTCAAGTCCCGGGGGAGGAGAAGAGAGGCTTCCCG-CCTAGAGCATTTGCAAGTCAGGATTCTCTAATCCCTCTGGGAGAAGGGTATTCGGCTTGTCCGCTGT-TTTT

>AP019119 Human gammaherpesvirus 4 UPN303_CD56+ DNA, nearly complete genome

AGGACAGCCGTTGCCCTAGTGGTTTCGGACACACCGCCAACGCTCAGTGCGGTGCTACCGACCCGAGGTCAAGTCCCGGGGGAGGAGAAGAGAGGCTTCCCG-CCTAGAGCATTTGCAAGTCAGGATTCTCTAATCCCTCTGGGAGAAGGGTATTCGGCTTGTCCGCTGT-TTTT

>AP019120 Human gammaherpesvirus 4 UPN306_CD56+ DNA, nearly complete genome

AGGACAGCCGTTGCCCTAGTGGTTTCGGACACACCGCCAACGCTCAGTGCGGTGCTACCGACCCGAGGTCAAGTCCCGGGGGAGGAGAAGAGAGGCTTCCCG-CCTAGAGCATTTGCAAGTCAGGATTCTCTAATCCCTCTGGGAGAAGGGTATTCGGCTTGTCCGCTGT-TTTT

>AP019121 Human gammaherpesvirus 4 UPN3065_PBMC DNA, nearly complete genome

AGGACAGCCGTTGCCCTAGTGGTTTCGGACACACCGCCAACGCTCAGTGCGGTGCTACCGACCCGAGGTCAAGTCCCGGGGGAGGAGAAGAGAGGCTTCCCG-CCTAGAGCATTTGCAAGTCAGGATTCTCTAATCCCTCTGGGAGAAGGGTATTCGGCTTGTCCGCTGT-TTTT

>AP019122 Human gammaherpesvirus 4 UPN307_CD56+ DNA, nearly complete genome

AGGACAGCCGTTGCCCTAGTGGTTTCGGACACACCGCCAACGCTCAGTGCGGTGCTACCGACCCGAGGTCAAGTCCCGGGGGAGGAGAAGAGAGGCTTCCCG-CCTAGAGCATTTGCAAGTCAGGATTCTCTAATCCCTCTGGGAGAAGGGTATTCGGCTTGTCCGCTGT-TTTT

>AP019123 Human gammaherpesvirus 4 UPN310_CD56+ DNA, nearly complete genome

AGGACAGCCGTTGCCCTAGTGGTTTCGGACACACCGCCAACGCTCAGTGCGGTGCTACCGACCCGAGGTCAAGTCCCGGGGGAGGAGAAGAGAGGCTTCCCG-CCTAGAGCATTTGCAAGTCAGGATTCTCTAATCCCTCTGGGAGAAGGGTATTCGGCTTGTCCGCTGT-TTTT

>AP019124 Human gammaherpesvirus 4 UPN311_CD56+ DNA, nearly complete genome

AGGACAGCCGTTGCCCTAGTGGTTTCGGACACACCGCCAACGCTCAGTGCGGTGCTACCGACCCGAGGTCAAGTCCCGGGGGAGGAGAAGAGAGGCTTCCCG-CCTAGAGCATTTGCAAGTCAGGATTCTCTAATCCCTCTGGGAGAAGGGTATTCGGCTTGTCCGCTGT-TTTT

>AP019125 Human gammaherpesvirus 4 UPN3198_PBMC DNA, nearly complete genome

AGGACAGCCGTTGCCCTAGTGGTTTCGGACACACCGCCAACGCTCAGTGCGGTGCTACCGACCCGAGGTCAAGTCCCGGGGGAGGAGAAGAGAGGCTTCCCG-CCTAGAGCATTTGCAAGTCAGGATTCTCTAATCCCTCTGGGAGAAGGGTATTCGGCTTGTCCGCTGT-TTTT

>AP019126 Human gammaherpesvirus 4 UPN322_CD3+ DNA, nearly complete genome

AGGACAGCCGTTGCCCTAGTGGTTTCGGACACACCGCCAACGCTCAGTGCGGTGCTACCGACCCGAGGTCAAGTCCCGGGGGAGGAGAAGAGAGGCTTCCCG-CCTAGAGCATTTGCAAGTCAGGATTCTCTAATCCCTCTGGGAGAAGGGTATTCGGCTTGTCCGCTGT-TTTT

>AP019127 Human gammaherpesvirus 4 UPN322_CD56+ DNA, nearly complete genome

AGGACAGCCGTTGCCCTAGTGGTTTCGGACACACCGCCAACGCTCAGTGCGGTGCTACCGACCCGAGGTCAAGTCCCGGGGGAGGAGAAGAGAGGCTTCCCG-CCTAGAGCATTTGCAAGTCAGGATTCTCTAATCCCTCTGGGAGAAGGGTATTCGGCTTGTCCGCTGT-TTTT

>AP019130 Human gammaherpesvirus 4 UPN3448_PBMC DNA, nearly complete genome

AGGACAGCCGTTGCCCTAGTGGTTTCGGACACACCGCCAACGCTCAGTGCGGTGCTACCGACCCGAGGTCAAGTCCCGGGGGAGGAGAAGAGAGGCTTCCCG-CCTAGAGCATTTGCAAGTCAGGATTCTCTAATCCCTCTGGGAGAAGGGTATTCGGCTTGTCCGCTGT-TTTT

>AP019131 Human gammaherpesvirus 4 UPN3621_PBMC DNA, nearly complete genome

AGGACAGCCGTTGCCCTAGTGGTTTCGGACACACCGCCAACGCTCAGTGCGGTGCTACCGACCCGAGGTCAAGTCCCGGGGGAGGAGAAGAGAGGCTTCCCG-CCTAGAGCATTTGCAAGTCAGGATTCTCTAATCCCTCTGGGAGAAGGGTATTCGGCTTGTCCGCTGT-TTTT

>AP019132 Human gammaherpesvirus 4 UPN365_CD3+ DNA, nearly complete genome

AGGACAGCCGTTGCCCTAGTGGTTTCGGACACACCGCCAACGCTCAGTGCGGTGCTACCGACCCGAGGTCAAGTCCCGGGGGAGGAGAAGAGAGGCTTCCCG-CCTAGAGCATTTGCAAGTCAGGATTCTCTAATCCCTCTGGGAGAAGGGTATTCGGCTTGTCCGCTGT-TTTT

>AP019133 Human gammaherpesvirus 4 UPN365_CD56+ DNA, nearly complete genome

AGGACAGCCGTTGCCCTAGTGGTTTCGGACACACCGCCAACGCTCAGTGCGGTGCTACCGACCCGAGGTCAAGTCCCGGGGGAGGAGAAGAGAGGCTTCCCG-CCTAGAGCATTTGCAAGTCAGGATTCTCTAATCCCTCTGGGAGAAGGGTATTCGGCTTGTCCGCTGT-TTTT

>AP019134 Human gammaherpesvirus 4 UPN366_CD3+ DNA, nearly complete genome

AGGACAGCCGTTGCCCTAGTGGTTTCGGACACACCGCCAACGCTCAGTGCGGTGCTACCGACCCGAGGTCAAGTCCCGGGGGAGGAGAAGAGAGGCTTCCCG-CCTAGAGCATTTGCAAGTCAGGATTCTCTAATCCCTCTGGGAGAAGGGTATTCGGCTTGTCCGCTGT-TTTT

>AP019135 Human gammaherpesvirus 4 UPN366_CD56+ DNA, nearly complete genome

AGGACAGCCGTTGCCCTAGTGGTTTCGGACACACCGCCAACGCTCAGTGCGGTGCTACCGACCCGAGGTCAAGTCCCGGGGGAGGAGAAGAGAGGCTTCCCG-CCTAGAGCATTTGCAAGTCAGGATTCTCTAATCCCTCTGGGAGAAGGGTATTCGGCTTGTCCGCTGT-TTTT

>AP019136 Human gammaherpesvirus 4 UPN3707_PBMC DNA, nearly complete genome

AGGACAGCCGTTGCCCTAGTGGTTTCGGACACACCGCCAACGCTCAGTGCGGTGCTACCGACCCGAGGTCAAGTCCCGGGGGAGGAGAAGAGAGGCTTCCCG-CCTAGAGCATTTGCAAGTCAGGATTCTCTAATCCCTCTGGGAGAAGGGTATTCGGCTTGTCCGCTGT-TTTT

>AP019137 Human gammaherpesvirus 4 UPN375_CD3+ DNA, nearly complete genome

AGGACAGCCGTTGCCCTAGTGGTTTCGGACACACCGCCAACGCTCAGTGCGGTGCTACCGACCCGAGGTCAAGTCCCGGGGGAGGAGAAGAGAGGCTTCCCG-CCTAGAGCATTTGCAAGTCAGGATTCTCTAATCCCTCTGGGAGAAGGGTATTCGGCTTGTCCGCTGT-TTTT

>AP019138 Human gammaherpesvirus 4 UPN375_CD56+ DNA, nearly complete genome

AGGACAGCCGTTGCCCTAGTGGTTTCGGACACACCGCCAACGCTCAGTGCGGTGCTACCGACCCGAGGTCAAGTCCCGGGGGAGGAGAAGAGAGGCTTCCCG-CCTAGAGCATTTGCAAGTCAGGATTCTCTAATCCCTCTGGGAGAAGGGTATTCGGCTTGTCCGCTGT-TTTT

>AP019139 Human gammaherpesvirus 4 UPN3755_tissue DNA, nearly complete genome

AGGACAGCCGTTGCCCTAGTGGTTTCGGACACACCGCCAACGCTCAGTGCGGTGCTACCGACCCGAGGTCAAGTCCCGGGGGAGGAGAAGAGAGGCTTCCCG-CCTAGAGCATTTGCAAGTCAGGATTCTCTAATCCCTCTGGGAGAAGGGTATTCGGCTTGTCCGCTGT-TTTT

>AP019140 Human gammaherpesvirus 4 UPN404_CD3+ DNA, nearly complete genome

AGGACAGCCGTTGCCCTAGTGGTTTCGGACACACCGCCAACGCTCAGTGCGGTGCTACCGACCCGAGGTCAAGTCCCGGGGGAGGAGAAGAGAGGCTTCCCG-CCTAGAGCATTTGCAAGTCAGGATTCTCTAATCCCTCTGGGAGAAGGGTATTCGGCTTGTCCGCTGT-TTTT

>AP019141 Human gammaherpesvirus 4 UPN404_CD56+ DNA, nearly complete genome

AGGACAGCCGTTGCCCTAGTGGTTTCGGACACACCGCCAACGCTCAGTGCGGTGCTACCGACCCGAGGTCAAGTCCCGGGGGAGGAGAAGAGAGGCTTCCCG-CCTAGAGCATTTGCAAGTCAGGATTCTCTAATCCCTCTGGGAGAAGGGTATTCGGCTTGTCCGCTGT-TTTT

>AP019142 Human gammaherpesvirus 4 UPN404_PBMC DNA, nearly complete genome

AGGACAGCCGTTGCCCTAGTGGTTTCGGACACACCGCCAACGCTCAGTGCGGTGCTACCGACCCGAGGTCAAGTCCCGGGGGAGGAGAAGAGAGGCTTCCCG-CCTAGAGCATTTGCAAGTCAGGATTCTCTAATCCCTCTGGGAGAAGGGTATTCGGCTTGTCCGCTGT-TTTT

>AP019143 Human gammaherpesvirus 4 UPN405_CD3+ DNA, nearly complete genome

AGGACAGCCGTTGCCCTAGTGGTTTCGGACACACCGCCAACGCTCAGTGCGGTGCTACCGACCCGAGGTCAAGTCCCGGGGGAGGAGAAGAGAGGCTTCCCG-CCTAGAGCATTTGCAAGTCAGGATTCTCTAATCCCTCTGGGAGAAGGGTATTCGGCTTGTCCGCTGT-TTTT

>AP019144 Human gammaherpesvirus 4 UPN405_CD56+ DNA, nearly complete genome

AGGACAGCCGTTGCCCTAGTGGTTTCGGACACACCGCCAACGCTCAGTGCGGTGCTACCGACCCGAGGTCAAGTCCCGGGGGAGGAGAAGAGAGGCTTCCCG-CCTAGAGCATTTGCAAGTCAGGATTCTCTAATCCCTCTGGGAGAAGGGTATTCGGCTTGTCCGCTGT-TTTT

>AP019145 Human gammaherpesvirus 4 UPN412_CD3+ DNA, nearly complete genome

AGGACAGCCGTTGCCCTAGTGGTTTCGGACACACCGCCAACGCTCAGTGCGGTGCTACCGACCCGAGGTCAAGTCCCGGGGGAGGAGAAGAGAGGCTTCCCG-CCTAGAGCATTTGCAAGTCAGGATTCTCTAATCCCTCTGGGAGAAGGGTATTCGGCTTGTCCGCTGT-TTTT

>AP019146 Human gammaherpesvirus 4 UPN412_CD56+ DNA, nearly complete genome

AGGACAGCCGTTGCCCTAGTGGTTTCGGACACACCGCCAACGCTCAGTGCGGTGCTACCGACCCGAGGTCAAGTCCCGGGGGAGGAGAAGAGAGGCTTCCCG-CCTAGAGCATTTGCAAGTCAGGATTCTCTAATCCCTCTGGGAGAAGGGTATTCGGCTTGTCCGCTGT-TTTT

>AP019147 Human gammaherpesvirus 4 UPN412_PBMC DNA, nearly complete genome

AGGACAGCCGTTGCCCTAGTGGTTTCGGACACACCGCCAACGCTCAGTGCGGTGCTACCGACCCGAGGTCAAGTCCCGGGGGAGGAGAAGAGAGGCTTCCCG-CCTAGAGCATTTGCAAGTCAGGATTCTCTAATCCCTCTGGGAGAAGGGTATTCGGCTTGTCCGCTGT-TTTT

>AP019148 Human gammaherpesvirus 4 UPN4137_tissue DNA, complete genome

AGGACAGCCGTTGCCCTAGTGGTTTCGGACACACCGCCAACGCTCAGTGCGGTGCTACCGACCCGAGGTCAAGTCCCGGGGGAGGAGAAGAGAGGCTTCCCG-CCTAGAGCATTTGCAAGTCAGGATTCTCTAATCCCTCTGGGAGAAGGGTATTCGGCTTGTCCGCTGT-TTTT

>AP019149 Human gammaherpesvirus 4 UPN417_CD3+ DNA, nearly complete genome

AGGACAGCCGTTGCCCTAGTGGTTTCGGACACACCGCCAACGCTCAGTGCGGTGCTACCGACCCGAGGTCAAGTCCCGGGGGAGGAGAAGAGAGGCTTCCCG-CCTAGAGCATTTGCAAGTCAGGATTCTCTAATCCCTCTGGGAGAAGGGTATTCGGCTTGTCCGCTGT-TTTT

>AP019150 Human gammaherpesvirus 4 UPN417_CD56+ DNA, nearly complete genome

AGGACAGCCGTTGCCCTAGTGGTTTCGGACACACCGCCAACGCTCAGTGCGGTGCTACCGACCCGAGGTCAAGTCCCGGGGGAGGAGAAGAGAGGCTTCCCG-CCTAGAGCATTTGCAAGTCAGGATTCTCTAATCCCTCTGGGAGAAGGGTATTCGGCTTGTCCGCTGT-TTTT

>AP019151 Human gammaherpesvirus 4 UPN417_PBMC DNA, nearly complete genome

AGGACAGCCGTTGCCCTAGTGGTTTCGGACACACCGCCAACGCTCAGTGCGGTGCTACCGACCCGAGGTCAAGTCCCGGGGGAGGAGAAGAGAGGCTTCCCG-CCTAGAGCATTTGCAAGTCAGGATTCTCTAATCCCTCTGGGAGAAGGGTATTCGGCTTGTCCGCTGT-TTTT

>AP019152 Human gammaherpesvirus 4 UPN431_CD19+ DNA, nearly complete genome

AGGACAGCCGTTGCCCTAGTGGTTTCGGACACACCGCCAACGCTCAGTGCGGTGCTACCGACCCGAGGTCAAGTCCCGGGGGAGGAGAAGAGAGGCTTCCCG-CCTAGAGCATTTGCAAGTCAGGATTCTCTAATCCCTCTGGGAGAAGGGTATTCGGCTTGTCCGCTGT-TTTT

>AP019153 Human gammaherpesvirus 4 UPN431_CD3+ DNA, nearly complete genome

AGGACAGCCGTTGCCCTAGTGGTTTCGGACACACCGCCAACGCTCAGTGCGGTGCTACCGACCCGAGGTCAAGTCCCGGGGGAGGAGAAGAGAGGCTTCCCG-CCTAGAGCATTTGCAAGTCAGGATTCTCTAATCCCTCTGGGAGAAGGGTATTCGGCTTGTCCGCTGT-TTTT

>AP019154 Human gammaherpesvirus 4 UPN431_CD56+ DNA, nearly complete genome

AGGACAGCCGTTGCCCTAGTGGTTTCGGACACACCGCCAACGCTCAGTGCGGTGCTACCGACCCGAGGTCAAGTCCCGGGGGAGGAGAAGAGAGGCTTCCCG-CCTAGAGCATTTGCAAGTCAGGATTCTCTAATCCCTCTGGGAGAAGGGTATTCGGCTTGTCCGCTGT-TTTT

>AP019155 Human gammaherpesvirus 4 UPN4314_PBMC DNA, complete genome

AGGACAGCCGTTGCCCTAGTGGTTTCGGACACACCGCCAACGCTCAGTGCGGTGCTACCGACCCGAGGTCAAGTCCCGGGGGAGGAGAAGAGAGGCTTCCCG-CCTAGAGCATTTGCAAGTCAGGATTCTCTAATCCCTCTGGGAGAAGGGTATTCGGCTTGTCCGCTGT-TTTT

>AP019156 Human gammaherpesvirus 4 UPN463_PBMC DNA, nearly complete genome

AGGACAGCCGTTGCCCTAGTGGTTTCGGACACACCGCCAACGCTCAGTGCGGTGCTACCGACCCGAGGTCAAGTCCCGGGGGAGGAGAAGAGAGGCTTCCCG-CCTAGAGCATTTGCAAGTCAGGATTCTCTAATCCCTCTGGGAGAAGGGTATTCGGCTTGTCCGCTGT-TTTT

>AP019157 Human gammaherpesvirus 4 UPN470_PBMC DNA, nearly complete genome

AGGACAGCCGTTGCCCTAGTGGTTTCGGACACACCGCCAACGCTCAGTGCGGTGCTACCGACCCGAGGTCAAGTCCCGGGGGAGGAGAAGAGAGGCTTCCCG-CCTAGAGCATTTGCAAGTCAGGATTCTCTAATCCCTCTGGGAGAAGGGTATTCGGCTTGTCCGCTGT-TTTT

>AP019158 Human gammaherpesvirus 4 UPN484_PBMC DNA, nearly complete genome

AGGACAGCCGTTGCCCTAGTGGTTTCGGACACACCGCCAACGCTCAGTGCGGTGCTACCGACCCGAGGTCAAGTCCCGGGGGAGGAGAAGAGAGGCTTCCCG-CCTAGAGCATTTGCAAGTCAGGATTCTCTAATCCCTCTGGGAGAAGGGTATTCGGCTTGTCCGCTGT-TTTT

>AP019160 Human gammaherpesvirus 4 UPN494_CD3+ DNA, nearly complete genome

AGGACAGCCGTTGCCCTAGTGGTTTCGGACACACCGCCAACGCTCAGTGCGGTGCTACCGACCCGAGGTCAAGTCCCGGGGGAGGAGAAGAGAGGCTTCCCG-CCTAGAGCATTTGCAAGTCAGGATTCTCTAATCCCTCTGGGAGAAGGGTATTCGGCTTGTCCGCTGT-TTTT

>AP019161 Human gammaherpesvirus 4 UPN494_CD56+ DNA, nearly complete genome

AGGACAGCCGTTGCCCTAGTGGTTTCGGACACACCGCCAACGCTCAGTGCGGTGCTACCGACCCGAGGTCAAGTCCCGGGGGAGGAGAAGAGAGGCTTCCCG-CCTAGAGCATTTGCAAGTCAGGATTCTCTAATCCCTCTGGGAGAAGGGTATTCGGCTTGTCCGCTGT-TTTT

>AP019162 Human gammaherpesvirus 4 UPN495_PBMC DNA, complete genome

AGGACAGCCGTTGCCCTAGTGGTTTCGGACACACCGCCAACGCTCAGTGCGGTGCTACCGACCCGAGGTCAAGTCCCGGGGGAGGAGAAGAGAGGCTTCCCG-CCTAGAGCATTTGCAAGTCAGGATTCTCTAATCCCTCTGGGAGAAGGGTATTCGGCTTGTCCGCTGT-TTTT

>AP019163 Human gammaherpesvirus 4 UPN497_PBMC DNA, nearly complete genome

AGGACAGCCGTTGCCCTAGTGGTTTCGGACACACCGCCAACGCTCAGTGCGGTGCTACCGACCCGAGGTCAAGTCCCGGGGGAGGAGAAGAGAGGCTTCCCG-CCTAGAGCATTTGCAAGTCAGGATTCTCTAATCCCTCTGGGAGAAGGGTATTCGGCTTGTCCGCTGT-TTTT

>AP019164 Human gammaherpesvirus 4 UPN498_PBMC DNA, nearly complete genome

AGGACAGCCGTTGCCCTAGTGGTTTCGGACACACCGCCAACGCTCAGTGCGGTGCTACCGACCCGAGGTCAAGTCCCGGGGGAGGAGAAGAGAGGCTTCCCG-CCTAGAGCATTTGCAAGTCAGGATTCTCTAATCCCTCTGGGAGAAGGGTATTCGGCTTGTCCGCTGT-TTTT

>AP019165 Human gammaherpesvirus 4 UPN5_PBMC DNA, nearly complete genome

AGGACAGCCGTTGCCCTAGTGGTTTCGGACACACCGCCAACGCTCAGTGCGGTGCTACCGACCCGAGGTCAAGTCCCGGGGGAGGAGAAGAGAGGCTTCCCG-CCTAGAGCATTTGCAAGTCAGGATTCTCTAATCCCTCTGGGAGAAGGGTATTCGGCTTGTCCGCTGT-TTTT

>AP019166 Human gammaherpesvirus 4 UPN5087_PBMC DNA, complete genome

AGGACAGCCGTTGCCCTAGTGGTTTCGGACACACCGCCAACGCTCAGTGCGGTGCTACCGACCCGAGGTCAAGTCCCGGGGGAGGAGAAGAGAGGCTTCCCG-CCTAGAGCATTTGCAAGTCAGGATTCTCTAATCCCTCTGGGAGAAGGGTATTCGGCTTGTCCGCTGT-TTTT

>AP019167 Human gammaherpesvirus 4 UPN5173_PBMC DNA, nearly complete genome

AGGACAGCCGTTGCCCTAGTGGTTTCGGACACACCGCCAACGCTCAGTGCGGTGCTACCGACCCGAGGTCAAGTCCCGGGGGAGGAGAAGAGAGGCTTCCCG-CCTAGAGCATTTGCAAGTCAGGATTCTCTAATCCCTCTGGGAGAAGGGTATTCGGCTTGTCCGCTGT-TTTT

>AP019168 Human gammaherpesvirus 4 UPN519_PBMC DNA, nearly complete genome

AGGACAGCCGTTGCCCTAGTGGTTTCGGACACACCGCCAACGCTCAGTGCGGTGCTACCGACCCGAGGTCAAGTCCCGGGGGAGGAGAAGAGAGGCTTCCCG-CCTAGAGCATTTGCAAGTCAGGATTCTCTAATCCCTCTGGGAGAAGGGTATTCGGCTTGTCCGCTGT-TTTT

>AP019169 Human gammaherpesvirus 4 UPN520_PBMC DNA, nearly complete genome

AGGACAGCCGTTGCCCTAGTGGTTTCGGACACACCGCCAACGCTCAGTGCGGTGCTACCGACCCGAGGTCAAGTCCCGGGGGAGGAGAAGAGAGGCTTCCCG-CCTAGAGCATTTGCAAGTCAGGATTCTCTAATCCCTCTGGGAGAAGGGTATTCGGCTTGTCCGCTGT-TTTT

>AP019170 Human gammaherpesvirus 4 UPN539_PBMC DNA, nearly complete genome

AGGACAGCCGTTGCCCTAGTGGTTTCGGACACACCGCCAACGCTCAGTGCGGTGCTACCGACCCGAGGTCAAGTCCCGGGGGAGGAGAAGAGAGGCTTCCCG-CCTAGAGCATTTGCAAGTCAGGATTCTCTAATCCCTCTGGGAGAAGGGTATTCGGCTTGTCCGCTGT-TTTT

>AP019171 Human gammaherpesvirus 4 UPN554_PBMC DNA, nearly complete genome

AGGACAGCCGTTGCCCTAGTGGTTTCGGACACACCGCCAACGCTCAGTGCGGTGCTACCGACCCGAGGTCAAGTCCCGGGGGAGGAGAAGAGAGGCTTCCCG-CCTAGAGCATTTGCAAGTCAGGATTCTCTAATCCCTCTGGGAGAAGGGTATTCGGCTTGTCCGCTGT-TTTT

>AP019173 Human gammaherpesvirus 4 UPN563_CD19+ DNA, nearly complete genome

AGGACAGCCGTTGCCCTAGTGGTTTCGGACACACCGCCAACGCTCAGTGCGGTGCTACCGACCCGAGGTCAAGTCCCGGGGGAGGAGAAGAGAGGCTTCCCG-CCTAGAGCATTTGCAAGTCAGGATTCTCTAATCCCTCTGGGAGAAGGGTATTCGGCTTGTCCGCTGT-TTTT

>AP019174 Human gammaherpesvirus 4 UPN563_CD3+ DNA, nearly complete genome

AGGACAGCCGTTGCCCTAGTGGTTTCGGACACACCGCCAACGCTCAGTGCGGTGCTACCGACCCGAGGTCAAGTCCCGGGGGAGGAGAAGAGAGGCTTCCCG-CCTAGAGCATTTGCAAGTCAGGATTCTCTAATCCCTCTGGGAGAAGGGTATTCGGCTTGTCCGCTGT-TTTT

>AP019175 Human gammaherpesvirus 4 UPN563_CD56+ DNA, nearly complete genome

AGGACAGCCGTTGCCCTAGTGGTTTCGGACACACCGCCAACGCTCAGTGCGGTGCTACCGACCCGAGGTCAAGTCCCGGGGGAGGAGAAGAGAGGCTTCCCG-CCTAGAGCATTTGCAAGTCAGGATTCTCTAATCCCTCTGGGAGAAGGGTATTCGGCTTGTCCGCTGT-TTTT

>AP019176 Human gammaherpesvirus 4 UPN571_CD3+ DNA, nearly complete genome

AGGACAGCCGTTGCCCTAGTGGTTTCGGACACACCGCCAACGCTCAGTGCGGTGCTACCGACCCGAGGTCAAGTCCCGGGGGAGGAGAAGAGAGGCTTCCCG-CCTAGAGCATTTGCAAGTCAGGATTCTCTAATCCCTCTGGGAGAAGGGTATTCGGCTTGTCCGCTGT-TTTT

>AP019177 Human gammaherpesvirus 4 UPN571_CD56+ DNA, nearly complete genome

AGGACAGCCGTTGCCCTAGTGGTTTCGGACACACCGCCAACGCTCAGTGCGGTGCTACCGACCCGAGGTCAAGTCCCGGGGGAGGAGAAGAGAGGCTTCCCG-CCTAGAGCATTTGCAAGTCAGGATTCTCTAATCCCTCTGGGAGAAGGGTATTCGGCTTGTCCGCTGT-TTTT

>AP019179 Human gammaherpesvirus 4 UPN582_PBMC DNA, complete genome

AGGACAGCCGTTGCCCTAGTGGTTTCGGACACACCGCCAACGCTCAGTGCGGTGCTACCGACCCGAGGTCAAGTCCCGGGGGAGGAGAAGAGAGGCTTCCCG-CCTAGAGCATTTGCAAGTCAGGATTCTCTAATCCCTCTGGGAGAAGGGTATTCGGCTTGTCCGCTGT-TTTT

>AP019180 Human gammaherpesvirus 4 UPN584_PBMC DNA, complete genome

AGGACAGCCGTTGCCCTAGTGGTTTCGGACACACCGCCAACGCTCAGTGCGGTGCTACCGACCCGAGGTCAAGTCCCGGGGGAGGAGAAGAGAGGCTTCCCG-CCTAGAGCATTTGCAAGTCAGGATTCTCTAATCCCTCTGGGAGAAGGGTATTCGGCTTGTCCGCTGT-TTTT

>AP019181 Human gammaherpesvirus 4 UPN874_PBMC DNA, nearly complete genome

AGGACAGCCGTTGCCCTAGTGGTTTCGGACACACCGCCAACGCTCAGTGCGGTGCTACCGACCCGAGGTCAAGTCCCGGGGGAGGAGAAGAGAGGCTTCCCG-CCTAGAGCATTTGCAAGTCAGGATTCTCTAATCCCTCTGGGAGAAGGGTATTCGGCTTGTCCGCTGT-TTTT

>AP019184 Human gammaherpesvirus 4 UPN93_gdT-2 DNA, nearly complete genome

AGGACAGCCGTTGCCCTAGTGGTTTCGGACACACCGCCAACGCTCAGTGCGGTGCTACCGACCCGAGGTCAAGTCCCGGGGGAGGAGAAGAGAGGCTTCCCG-CCTAGAGCATTTGCAAGTCAGGATTCTCTAATCCCTCTGGGAGAAGGGTATTCGGCTTGTCCGCTGT-TTTT

>AP019185 Human gammaherpesvirus 4 UPN97_PBMC DNA, nearly complete genome

AGGACAGCCGTTGCCCTAGTGGTTTCGGACACACCGCCAACGCTCAGTGCGGTGCTACCGACCCGAGGTCAAGTCCCGGGGGAGGAGAAGAGAGGCTTCCCG-CCTAGAGCATTTGCAAGTCAGGATTCTCTAATCCCTCTGGGAGAAGGGTATTCGGCTTGTCCGCTGT-TTTT

>AP019186 Human gammaherpesvirus 4 UPN97_tumor DNA, nearly complete genome

AGGACAGCCGTTGCCCTAGTGGTTTCGGACACACCGCCAACGCTCAGTGCGGTGCTACCGACCCGAGGTCAAGTCCCGGGGGAGGAGAAGAGAGGCTTCCCG-CCTAGAGCATTTGCAAGTCAGGATTCTCTAATCCCTCTGGGAGAAGGGTATTCGGCTTGTCCGCTGT-TTTT

>AP019187 Human gammaherpesvirus 4 UPN98_PBMC DNA, nearly complete genome

AGGACAGCCGTTGCCCTAGTGGTTTCGGACACACCGCCAACGCTCAGTGCGGTGCTACCGACCCGAGGTCAAGTCCCGGGGGAGGAGAAGAGAGGCTTCCCG-CCTAGAGCATTTGCAAGTCAGGATTCTCTAATCCCTCTGGGAGAAGGGTATTCGGCTTGTCCGCTGT-TTTT

>AP019188 Human gammaherpesvirus 4 UPN99_PBMC DNA, nearly complete genome

AGGACAGCCGTTGCCCTAGTGGTTTCGGACACACCGCCAACGCTCAGTGCGGTGCTACCGACCCGAGGTCAAGTCCCGGGGGAGGAGAAGAGAGGCTTCCCG-CCTAGAGCATTTGCAAGTCAGGATTCTCTAATCCCTCTGGGAGAAGGGTATTCGGCTTGTCCGCTGT-TTTT

>AY961628 Human herpesvirus 4 strain GD1, complete genome

AGGACAGCCGTTGCCCTAGTGGTTTCGGACACACCGCCAACGCTCAGTGCGGTGCTACCGACCCGAGGTCAAGTCCCGGGGGAGGAGAAGAGAGGCTTCCCG-CCTAGAGCATTTGCAAGTCAGGATTCTCTAATCCCTCTGGGAGAAGGGTATTCGGCTTGTCCGCTGT-TTTT

>DQ279927 Human herpesvirus 4 strain AG876, complete genome

AGGACAGCCGTTGCCCTAGTGGTTTCGGACACACCGCCAACGCTCAGTGCGGTGCTACCGACCCGAGGTCAAGTCCCGGGGGAGGAGAAGAGAGGCTTCCCG-CCTAGAGCATTTGCAAGTCAGGATTCTCTAATCCCTCTGGGAGAAGGGTATTCGGCTTGTCCGCTGT-TTTT

>EF187849 Human herpesvirus 4 isolate SNU-299 EBER-1 and EBER-2 genes, complete sequence

AGGACAGCCGTTGCCCTAGTGGTTTCGGACACACCGCCAACGCTCAGTGCGGTGCTACCGACCCGAGGTCAAGTCCCGGGGGAGGAGAAGAGAGGCTTCCCG-CCTAGAGCATTTGCAAGTCAGGATTCTCTAATCCCTCTGGGAGAAGGGTATTCGGCTTGTCCGCTGT-TTTT

>EF187850 Human herpesvirus 4 isolate MJC-26 EBER-1 and EBER-2 genes, complete sequence

AGGACAGCCGTTGCCCTAGTGGTTTCGGACACACCGCCAACGCTCAGTGCGGTGCTACCGACCCGAGGTCAAGTCCCGGGGGAGGAGAAGAGAGGCTTCCCG-CCTAGAGCATTTGCAAGTCAGGATTCTCTAATCCCTCTGGGAGAAGGGTATTCGGCTTGTCCGCTGT-TTTT

>EF187851 Human herpesvirus 4 isolate SNU-99 EBER-1 and EBER-2 genes, complete sequence

AGGACAGCCGTTGCCCTAGTGGTTTCGGACACACCGCCAACGCTCAGTGCGGTGCTACCGACCCGAGGTCAAGTCCCGGGGGAGGAGAAGAGAGGCTTCCCG-CCTAGAGCATTTGCAAGTCAGGATTCTCTAATCCCTCTGGGAGAAGGGTATTCGGCTTGTCCGCTGT-TTTT

>EF187852 Human herpesvirus 4 isolate SNU-445 EBER-1 and EBER-2 genes, complete sequence

AGGACAGCCGTTGCCCTAGTGGTTTCGGACACACCGCCAACGCTCAGTGCGGTGCTACCGACCCGAGGTCAAGTCCCGGGGGAGGAGAAGAGAGGCTTCCCG-CCTAGAGCATTTGCAAGTCAGGATTCTCTAATCCCTCTGGGAGAAGGGTATTCGGCTTGTCCGCTGT-TTTT

>EF187853 Human herpesvirus 4 isolate SNU-1103 EBER-1 and EBER-2 genes, complete sequence

AGGACAGCCGTTGCCCTAGTGGTTTCGGACACACCGCCAACGCTCAGTGCGGTGCTACCGACCCGAGGTCAAGTCCCGGGGGAGGAGAAGAGAGGCTTCCCG-CCTAGAGCATTTGCAAGTCAGGATTCTCTAATCCCTCTGGGAGAAGGGTATTCGGCTTGTCCGCTGT-TTTT

>J02077 epstein-barr virus small rna genes(j rnai & j rnaii)

AGGACAGCCGTTGCCCTAGTGGTTTCGGACACACCGCCAACGCTCAGTGCGGTGCTACCGACCCGAGGTCAAGTCCCGGGGAAGGAGAAGAGAGGCTTCCCG-CCTAGAGCATTTGCAAGTCAGGATTCTCTAATCCCTCTGGGAGAAGGGTATTCGGCTTGTCCGCTAT-TTTT

>KC207813 Human herpesvirus 4 strain Akata, complete genome

AGGACAGCCGTTGCCCTAGTGGTTTCGGACACACCGCCAACGCTCAGTGCGGTGCTACCGACCCGAGGTCAAGTCCCGGGGGAGGAGAAGAGAGGCTTCCCG-CCTAGAGCATTTGCAAGTCAGGATTCTCTAATCCCTCTGGGAGAAGGGTATTCGGCTTGTCCGCTGT-TTTT

>KC207814 Human herpesvirus 4 strain Mutu, complete genome

AGGACAGCCGTTGCCCTAGTGGTTTCGGACACACCGCCAACGCTCAGTGCGGTGCTACCGACCCGAGGTCAAGTCCCGGGGGAGGAGAAGAGAGGCTTCCCG-CCTAGAGCATTTGCAAGTCAGGATTCTCTAATCCCTCTGGGAGAAGGGTATTCGGCTTGTCCGCTGT-TTTT

>KC440851 Human herpesvirus 4 strain K4123-Mi, partial genome

AGGACAGCCGTTGCCCTAGTGGTTTCGGACACACCGCCAACGCTCAGTGCGGTGCTACCGACCCGAGGTCAAGTCCCGGGGGAGGAGAAGAGAGGCTTCCCG-CCTAGAGCATTTGCAAGTCAGGATTCTCTAATCCCTCTGGGAGAAGGGTATTCGGCTTGTCCGCTGT-TTTT

>KC440852 Human herpesvirus 4 strain K4123-MiEBV, partial genome

AGGACAGCCGTTGCCCTAGTGGTTTCGGACACACCGCCAACGCTCAGTGCGGTGCTACCGACCCGAGGTCAAGTCCCGGGGGAGGAGAAGAGAGGCTTCCCG-CCTAGAGCATTTGCAAGTCAGGATTCTCTAATCCCTCTGGGAGAAGGGTATTCGGCTTGTCCGCTGT-TTTT

>KF717093 Human herpesvirus 4 strain Raji, complete genome

AGGACAGCCGTTGCCCTAGTGGTTTCGGACACACCGCCAACGCTCAGTGCGGTGCTACCGACCCGAGGTCAAGTCCCGGGGGAGGAGAAGAGAGGCTTCCCG-CCTAGAGCATTTGCAAGTCAGGATTCTCTAATCCCTCTGGGAGAAGGGTATTCGGCTTGTCCGCTGT-TTTT

>KP195386 Human herpesvirus 4 isolate GDNPC3 EBER1 and EBER2 genes, complete sequence

AGGACAGCCGTTGCCCTAGTGGTTTCGGACACACCGCCAACGCTCAGTGCGGTGCTACCGACCCGAGGTCAAGTCCCGGGGGAGGAGAAGAGAGGCTTCCCG-CCTAGAGCATTTGCAAGTCAGGATTCTCTAATCCCTCTGGGAGAAGGGTATTCGGCTTGTCCGCTGT-TTTT

>KP195388 Human herpesvirus 4 isolate GDNPC5 EBER1 and EBER2 genes, complete sequence

AGGACAGCCGTTGCCCTAGTGGTTTCGGACACACCGCCAACGCTCAGTGCGGTGCTACCGACCCGAGGTCAAGTCCCGGGGGAGGAGAAGAGAGGCTTCCCG-CCTAGAGCATTTGCAAGTCAGGATTCTCTAATCCCTCTGGGAGAAGGGTATTCGGCTTGTCCGCTGT-TTTT

>KP195408 Human herpesvirus 4 isolate GDNPC28 EBER1 and EBER2 genes, complete sequence

AGGACAGCCGTTGCCCTAGTGGTTTCGGACACACCGCCAACGCTCAGTGCGGTGCTACCGACCCGAGGTCAAGTCCCGGGGGAGGAGAAGAGAGGCTTCCCG-CCTAGAGCATTTGCAAGTCAGGATTCTCTAATCCCTCTGGGAGAAGGGTATTCGGCTTGTCCGCTGT-TTTT

>KP195439 Human herpesvirus 4 isolate SDNPC9 EBER1 and EBER2 genes, complete sequence

AGGACAGCCGTTGCCCTAGTGGTTTCGGACACACCGCCAACGCTCAGTGCGGTGCTACCGACCCGAGGTCAAGTCCCGGGGGAGGAGAAGAGAGGCTTCCCG-CCTAGAGCATTTGCAAGTCAGGATTCTCTAATCCCTCTGGGAGAAGGGTATTCGGCTTGTCCGCTTT-TTTT

>KP195442 Human herpesvirus 4 isolate SDNPC13 EBER1 and EBER2 genes, complete sequence

AGGACAGCCGTTGCCCTAGTGGTTTCGGACACACCGCCAACGCTCAGTGCGGTGCTACCGACCCGAGGTCAAGTCCCGGGGGAGGAGAAGAGAGGCTTCCCG-CCTAGAGCATTTGCAAGTCAGGATTCTCTAATCCCTCTGGGAGAAGGGTATTCGGCTTGTCCGCTTT-TTTT

>KP195452 Human herpesvirus 4 isolate SDNPC24 EBER1 and EBER2 genes, complete sequence

AGGACAGCCGTTGCCCTAGTGGTTTCGGACACACCGCCAACGCTCAGTGCGGTGCTACCGACCCGAGGTCAAGTCCCGGGGGAGGAGAAGAGAGGCTTCCCG-CCTAGAGCATTTGCAAGTCAGGATTCTCTAATCCCTCTGGGAGAAGGGTATTCGGCTTGTCCGCTGT-TTTT

>KP195453 Human herpesvirus 4 isolate SDNPC25 EBER1 and EBER2 genes, complete sequence

AGGACAGCCGTTGCCCTAGTGGTTTCGGACACACCGCCAACGCTCAGTGCGGTGCTACCGACCCGAGGTCAAGTCCCGGGGGAGGAGAAGAGAGGCTTCCCG-CCTAGAGCATTTGCAAGTCAGGATTCTCTAATCCCTCTGGGAGAAGGGTATTCGGCTTGTCCGCTGT-TTTT

>KP195456 Human herpesvirus 4 isolate SDNPC32 EBER1 and EBER2 genes, complete sequence

AGGACAGCCGTTGCCCTAGTGGTTTCGGACACACCGCCAACGCTCAGTGCGGTGCTACCGACCCGAGGTCAAGTCCCGGGGGAGGAGAAGAGAGGCTTCCCG-CCTAGAGCATTTGCAAGTCAGGATTCTCTAATCCCTCTGGGAGAAGGGTATTCGGCTTGTCCGCTGT-TTTT

>KP195464 Human herpesvirus 4 isolate SDNPC40 EBER1 and EBER2 genes, complete sequence

AGGACAGCCGTTGCCCTAGTGGTTTCGGACACACCGCCAACGCTCAGTGCGGTGCTACCGACCCGAGGTCAAGTCCCGGGGGAGGAGAAGAGAGGCTTCCCG-CCTAGAGCATTTGCAAGTCAGGATTCTCTAATCCCTCTGGGAGAAGGGTATTCGGCTTGTCCGCTGT-TTTT

>KP195472 Human herpesvirus 4 isolate SDNPC49 EBER1 and EBER2 genes, complete sequence

AGGACAGCCGTTGCCCTAGTGGTTTCGGACACACCGCCAACGCTCAGTGCGGTGCTACCGACCCGAGGTCAAGTCCCGGGGGAGGAGAAGAGAGGCTTCCCG-CCTAGAGCATTTGCAAGTCAGGATTCTCTAATCCCTCTGGGAGAAGGGTATTCGGCTTGTCCGCTGT-TTTT

>KP195475 Human herpesvirus 4 isolate SDNPC52 EBER1 and EBER2 genes, complete sequence

AGGACAGCCGTTGCCCTAGTGGTTTCGGACACACCGCCAACGCTCAGTGCGGTGCTACCGACCCGAGGTCAAGTCCCGGGGGAGGAGAAGAGAGGCTTCCCG-CCTAGAGCATTTGCAAGTCAGGATTCTCTAATCCCTCTGGGAGAAGGGTATTCGGCTTGTCCGCTGT-TTTT

>KP195477 Human herpesvirus 4 isolate SDNPC54 EBER1 and EBER2 genes, complete sequence

AGGACAGCCGTTGCCCTAGTGGTTTCGGACACACCGCCAACGCTCAGTGCGGTGCTACCGACCCGAGGTCAAGTCCCGGGGGAGGAGAAGAGAGGCTTCCCG-CCTAGAGCATTTGCAAGTCAGGATTCTCTAATCCCTCTGGGAGAAGGGTATTCGGCTTGTCCGCTGT-TTTT

>KP195478 Human herpesvirus 4 isolate SDNPC55 EBER1 and EBER2 genes, complete sequence

AGGACAGCCGTTGCCCTAGTGGTTTCGGACACACCGCCAACGCTCAGTGCGGTGCTACCGACCCGAGGTCAAGTCCCGGGGGAGGAGAAGAGAGGCTTCCCG-CCTAGAGCATTTGCAAGTCAGGATTCTCTAATCCCTCTGGGAGAAGGGTATTCGGCTTGTCCGCTGT-TTTT

>KP195480 Human herpesvirus 4 isolate SDNPC57 EBER1 and EBER2 genes, complete sequence

AGGACAGCCGTTGCCCTAGTGGTTTCGGACACACCGCCAACGCTCAGTGCGGTGCTACCGACCCGAGGTCAAGTCCCGGGGGAGGAGAAGAGAGGCTTCCCG-CCTAGAGCATTTGCAAGTCAGGATTCTCTAATCCCTCTGGGAGAAGGGTATTCGGCTTGTCCGCTGT-TTTT

>KP195481 Human herpesvirus 4 isolate SDNPC58 EBER1 and EBER2 genes, complete sequence

AGGACAGCCGTTGCCCTAGTGGTTTCGGACACACCGCCAACGCTCAGTGCGGTGCTACCGACCCGAGGTCAAGTCCCGGGGGAGGAGAAGAGAGGCTTCCCG-CCTAGAGCATTTGCAAGTCAGGATTCTCTAATCCCTCTGGGAGAAGGGTATTCGGCTTGTCCGCTGT-TTTT

>KP195494 Human herpesvirus 4 isolate SDNPC101 EBER1 and EBER2 genes, complete sequence

AGGACAGCCGTTGCCCTAGTGGTTTCGGACACACCGCCAACGCTCAGTGCGGTGCTACCGACCCGAGGTCAAGTCCCGGGGGAGGAGAAGAGAGGCTTCCCG-CCTAGAGCATTTGCAAGTCAGGATTCTCTAATCCCTCTGGGAGAAGGGTATTCGGCTTGTCCGCTGT-TTTT

>KP195530 Human herpesvirus 4 isolate GDTW1 EBER1 and EBER2 genes, complete sequence

AGGACAGCCGTTGCCCTAGTGGTTTCGGACACACCGCCAACGCTCAGTGCGGTGCTACCGACCCGAGGTCAAGTCCCGGGGGAGGAGAAGAGAGGCTTCCCG-CCTAGAGCATTTGCAAGTCAGGATTCTCTAATCCCTCTGGGAGAAGGGTATTCGGCTTGTCCGCTGT-TTTT

>KP195604 Human herpesvirus 4 isolate GDTW138 EBER1 and EBER2 genes, complete sequence

AGGACAGCCGTTGCCCTAGTGGTTTCGGACACACCGCCAACGCTCAGTGCGGTGCTACCGACCCGAGGTCAAGTCCCGGGGGAGGAGAAGAGAGGCTTCCCG-CCTAGAGCATTTGCAAGTCAGGATTCTCTAATCCCTCTGGGAGAAGGGTATTCGGCTTGTCCGCTGT-TTTT

>KP195607 Human herpesvirus 4 isolate GDTW151 EBER1 and EBER2 genes, complete sequence

AGGACAGCCGTTGCCCTAGTGGTTTCGGACACACCGCCAACGCTCAGTGCGGTGCTACCGACCCGAGGTCAAGTCCCGGGGGAGGAGAAGAGAGGCTTCCCG-CCTAGAGCATTTGCAAGTCAGGATTCTCTAATCCCTCTGGGAGAAGGGTATTCGGCTTGTCCGCTGT-TTTT

>KP195624 Human herpesvirus 4 isolate SDTW86 EBER1 and EBER2 genes, complete sequence

AGGACAGCCGTTGCCCTAGTGGTTTCGGACACACCGCCAACGCTCAGTGCGGTGCTACCGACCCGAGGTCAAGTCCCGGGGGAGGAGAAGAGAGGCTTCCCG-CCTAGAGCATTTGCAAGTCAGGATTCTCTAATCCCTCTGGGAGAAGGGTATTCGGCTTGTCCGCTGT-TTTT

>KP195630 Human herpesvirus 4 isolate SDTW106 EBER1 and EBER2 genes, complete sequence

AGGACAGCCGTTGCCCTAGTGGTTTCGGACACACCGCCAACGCTCAGTGCGGTGCTACCGACCCGAGGTCAAGTCCCGGGGGAGGAGAAGAGAGGCTTCCCG-CCTAGAGCATTTGCAAGTCAGGATTCTCTAATCCCTCTGGGAGAAGGGTATTCGGCTTGTCCGCTGT-TTTT

>KP195632 Human herpesvirus 4 isolate SDTW111 EBER1 and EBER2 genes, complete sequence

AGGACAGCCGTTGCCCTAGTGGTTTCGGACACACCGCCAACGCTCAGTGCGGTGCTACCGACCCGAGGTCAAGTCCCGGGGGAGGAGAAGAGAGGCTTCCCG-CCTAGAGCATTTGCAAGTCAGGATTCTCTAATCCCTCTGGGAGAAGGGTATTCGGCTTGTCCGCTGT-TTTT

>KP195651 Human herpesvirus 4 isolate SDTW143 EBER1 and EBER2 genes, complete sequence

AGGACAGCCGTTGCCCTAGTGGTTTCGGACACACCGCCAACGCTCAGTGCGGTGCTACCGACCCGAGGTCAAGTCCCGGGGGAGGAGAAGAGAGGCTTCCCG-CCTAGAGCATTTGCAAGTCAGGATTCTCTAATCCCTCTGGGAGAAGGGTATTCGGCTTGTCCGCTGT-TTTT

>KP195665 Human herpesvirus 4 isolate SDTW187 EBER1 and EBER2 genes, complete sequence

AGGACAGCCGTTGCCCTAGTGGTTTCGGACACACCGCCAACGCTCAGTGCGGTGCTACCGACCCGAGGTCAAGTCCCGGGGGAGGAGAAGAGAGGCTTCCCG-CCTAGAGCATTTGCAAGTCAGGATTCTCTAATCCCTCTGGGAGAAGGGTATTCGGCTTGTCCGCTGT-TTTT

>KP195667 Human herpesvirus 4 isolate SDTW229 EBER1 and EBER2 genes, complete sequence

AGGACAGCCGTTGCCCTAGTGGTTTCGGACACACCGCCAACGCTCAGTGCGGTGCTACCGACCCGAGGTCAAGTCCCGGGGGAGGAGAAGAGAGGCTTCCCG-CCTAGAGCATTTGCAAGTCAGGATTCTCTAATCCCTCTGGGAGAAGGGTATTCGGCTTGTCCGCTGT-TTTT

>KP195673 Human herpesvirus 4 isolate SDTW252 EBER1 and EBER2 genes, complete sequence

AGGACAGCCGTTGCCCTAGTGGTTTCGGACACACCGCCAACGCTCAGTGCGGTGCTACCGACCCGAGGTCAAGTCCCGGGGGAGGAGAAGAGAGGCTTCCCG-CCTAGAGCATTTGCAAGTCAGGATTCTCTAATCCCTCTGGGAGAAGGGTATTCGGCTTGTCCGCTGT-TTTT

>KP735248 Human herpesvirus 4 strain GC1, complete genome

AGGACAGCCGTTGCCCTAGTGGTTTCGGACACACCGCCAACGCTCAGTGCGGTGCTACCGACCCGAGGTCAAGTCCCGGGGGAGGAGAAGAGAGGCTTCCCG-CCTAGAGCATTTGCAAGTCAGGATTCTCTAATCCCTCTGGGAGAAGGGTATTCGGCTTGTCCGCTGT-TTTT

>KP968257 Human herpesvirus 4 strain CCH, partial genome

AGGACAGCCGTTGCCCTAGTGGTTTCGGACACACCGCCAACGCTCAGTGCGGTGCTACCGACCCGAGGTCAAGTCCCGGGGGAGGAGAAGAGAGGCTTCCCG-CCTAGAGCATTTGCAAGTCAGGATTCTCTAATCCCTCTGGGAGAAGGGTATTCGGCTTGTCCGCTGT-TTTT

>KP968258 Human herpesvirus 4 strain MP, partial genome

AGGACAGCCGTTGCCCTAGTGGTTTCGGACACACCGCCAACGCTCAGTGCGGTGCTACCGACCCGAGGTCAAGTCCCGGGGGAGGAGAAGAGAGGCTTCCCG-CCTAGAGCATTTGCAAGTCAGGATTCTCTAATCCCTCTGGGAGAAGGGTATTCGGCTTGTCCGCTGT-TTTT

>KP968259 Human herpesvirus 4 strain SCL, partial genome

AGGACAGCCGTTGCCCTAGTGGTTTCGGACACACCGCCAACGCTCAGTGCGGTGCTACCGACCCGAGGTCAAGTCCCGGGGGAGGAGAAGAGAGGCTTCCCG-CCTAGAGCATTTGCAAGTCAGGATTCTCTAATCCCTCTGGGAGAAGGGTATTCGGCTTGTCCGCTGT-TTTT

>KP968261 Human herpesvirus 4 strain HU11393, partial genome

AGGACAGCCGTTGCCCTAGTGGTTTCGGACACACCGCCAACGCTCAGTGCGGTGCTACCGACCCGAGGTCAAGTCCCGGGGGAGGAGAAGAGAGGCTTCCCG-CCTAGAGCATTTGCAAGTCAGGATTCTCTAATCCCTCTGGGAGAAGGGTATTCGGCTTGTCCGCTGT-TTTT

>KP968262 Human herpesvirus 4 strain H018436D, partial genome

AGGACAGCCGTTGCCCTAGTGGTTTCGGACACACCGCCAACGCTCAGTGCGGTGCTACCGACCCGAGGTCAAGTCCCGGGGGAGGAGAAGAGAGGCTTCCCG-CCTAGAGCATTTGCAAGTCAGGATTCTCTAATCCCTCTGGGAGAAGGGTATTCGGCTTGTCCGCTGT-TTTT

>KP968263 Human herpesvirus 4 strain H058015C, partial genome

AGGACAGCCGTTGCCCTAGTGGTTTCGGACACACCGCCAACGCTCAGTGCGGTGCTACCGACCCGAGGTCAAGTCCCGGGGGAGGAGAAGAGAGGCTTCCCG-CCTAGAGCATTTGCAAGTCAGGATTCTCTAATCCCTCTGGGAGAAGGGTATTCGGCTTGTCCGCTGT-TTTT

>KP968264 Human herpesvirus 4 strain H002213, partial genome

AGGACAGCCGTTGCCCTAGTGGTTTCGGACACACCGCCAACGCTCAGTGCGGTGCTACCGACCCGAGGTCAAGTCCCGGGGGAGGAGAAGAGAGGCTTCCCG-CCTAGAGCATTTGCAAGTCAGGATTCTCTAATCCCTCTGGGAGAAGGGTATTCGGCTTGTCCGCTGT-TTTT

>KR063342 Human gammaherpesvirus 4 strain H03753A

AGGACAGCCGTTGCCCTAGTGGTTTCGGACACACCGCCAACGCTCAGTGCGGTGCTACCGACCCGAGGTCAAGTCCCGGGGGAGGAGAAGAGAGGCTTCCCG-CCTAGAGCATTTGCAAGTCAGGATTCTCTAATCCCTCTGGGAGAAGGGTATTCGGCTTGTCCGCTGT-TTTT

>KR063345 Human gammaherpesvirus 4 strain FNR

AGGACAGCCGTTGCCCTAGTGGTTTCGGACACACCGCCAACGCTCAGTGCGGTGCTACCGACCCGAGGTCAAGTCCCGGGGGAGGAGAAGAGAGGCTTCCCG-CCTAGAGCATTTGCAAGTCAGGATTCTCTAATCCCTCTGGGAGAAGGGTATTCGGCTTGTCCGCTGT-TTTT

>KT001102 Human gammaherpesvirus 4 strain VA

AGGACAGCCGTTGCCCTAGTGGTTTCGGACACACCGCCAACGCTCAGTGCGGTGCTACCGACCCGAGGTCAAGTCCCGGGGGAGGAGAAGAGAGGCTTCCCG-CCTAGAGCATTTGCAAGTCAGGATTCTCTAATCCCTCTGGGAGAAGGGTATTCGGCTTGTCCGCTGT-TTTT

>KT001103 Human gammaherpesvirus 4 strain SG

AGGACAGCCGTTGCCCTAGTGGTTTCGGACACACCGCCAACGCTCAGTGCGGTGCTACCGACCCGAGGTCAAGTCCCGGGGGAGGAGAAGAGAGGCTTCCCG-CCTAGAGCATTTGCAAGTCAGGATTCTCTAATCCCTCTGGGAGAAGGGTATTCGGCTTGTCCGCTGT-TTTT

>KT254013 Human gammaherpesvirus 4 isolate EBVaGC3

AGGACAGCCGTTGCCCTAGTGGTTTCGGACACACCGCCAACGCTCAGTGCGGTGCTACCGACCCGAGGTCAAGTCCCGGGGGAGGAGAAGAGAGGCTTCCCG-CCTAGAGCATTTGCAAGTCAGGATTCTCTAATCCCTCTGGGAGAAGGGTATTCGGCTTGTCCGCTGT-TTTT

>KT273942 Human gammaherpesvirus 4 isolate EBVaGC1

AGGACAGCCGTTGCCCTAGTGGTTTCGGACACACCGCCAACGCTCAGTGCGGTGCTACCGACCCGAGGTCAAGTCCCGGGGGAGGAGAAGAGAGGCTTCCCG-CCTAGAGCATTTGCAAGTCAGGATTCTCTAATCCCTCTGGGAGAAGGGTATTCGGCTTGTCCGCTGT-TTTT

>KT273943 Human gammaherpesvirus 4 isolate EBVaGC2

AGGACAGCCGTTGCCCTAGTGGTTTCGGACACACCGCCAACGCTCAGTGCGGTGCTACCGACCCGAGGTCAAGTCCCGGGGGAGGAGAAGAGAGGCTTCCCG-CCTAGAGCATTTGCAAGTCAGGATTCTCTAATCCCTCTGGGAGAAGGGTATTCGGCTTGTCCGCTGT-TTTT

>KT273944 Human gammaherpesvirus 4 isolate EBVaGC4

AGGACAGCCGTTGCCCTAGTGGTTTCGGACACACCGCCAACGCTCAGTGCGGTGCTACCGACCCGAGGTCAAGTCCCGGGGGAGGAGAAGAGAGGCTTCCCG-CCTAGAGCATTTGCAAGTCAGGATTCTCTAATCCCTCTGGGAGAAGGGTATTCGGCTTGTCCGCTGT-TTTT

>KT273945 Human gammaherpesvirus 4 isolate EBVaGC5

AGGACAGCCGTTGCCCTAGTGGTTTCGGACACACCGCCAACGCTCAGTGCGGTGCTACCGACCCGAGGTCAAGTCCCGGGGGAGGAGAAGAGAGGCTTCCCG-CCTAGAGCATTTGCAAGTCAGGATTCTCTAATCCCTCTGGGAGAAGGGTATTCGGCTTGTCCGCTGT-TTTT

>KT273946 Human gammaherpesvirus 4 isolate EBVaGC6

AGGACAGCCGTTGCCCTAGTGGTTTCGGACACACCGCCAACGCTCAGTGCGGTGCTACCGACCCGAGGTCAAGTCCCGGGGGAGGAGAAGAGAGGCTTCCCG-CCTAGAGCATTTGCAAGTCAGGATTCTCTAATCCCTCTGGGAGAAGGGTATTCGGCTTGTCCGCTGT-TTTT

>KT273947 Human gammaherpesvirus 4 isolate EBVaGC7

AGGACAGCCGTTGCCCTAGTGGTTTCGGACACACCGCCAACGCTCAGTGCGGTGCTACCGACCCGAGGTCAAGTCCCGGGGGAGGAGAAGAGAGGCTTCCCG-CCTAGAGCATTTGCAAGTCAGGATTCTCTAATCCCTCTGGGAGAAGGGTATTCGGCTTGTCCGCTGT-TTTT

>KT273948 Human gammaherpesvirus 4 isolate EBVaGC8

AGGACAGCCGTTGCCCTAGTGGTTTCGGACACACCGCCAACGCTCAGTGCGGTGCTACCGACCCGAGGTCAAGTCCCGGGGGAGGAGAAGAGAGGCTTCCCG-CCTAGAGCATTTGCAAGTCAGGATTCTCTAATCCCTCTGGGAGAAGGGTATTCGGCTTGTCCGCTGT-TTTT

>KT823506 Human gammaherpesvirus 4 isolate LC1

AGGACAGCCGTTGCCCTAGTGGTTTCGGACACACCGCCAACGCTCAGTGCGGTGCTACCGACCCGAGGTCAAGTCCCGGGGGAGGAGAAGAGAGGCTTCCCG-CCTAGAGCATTTGCAAGTCAGGATTCTCTAATCCCTCTGGGAGAAGGGTATTCGGCTTGTCCGCTGT-TTTT

>KT823508 Human gammaherpesvirus 4 isolate LC3

AGGACAGCCGTTGCCCTAGTGGTTTCGGACACACCGCCAACGCTCAGTGCGGTGCTACCGACCCGAGGTCAAGTCCCGGGGGAGGAGAAGAGAGGCTTCCCG-CCTAGAGCATTTGCAAGTCAGGATTCTCTAATCCCTCTGGGAGAAGGGTATTCGGCTTGTCCGCTGT-TTTT

>KT823509 Human gammaherpesvirus 4 isolate LC4

AGGACAGCCGTTGCCCTAGTGGTTTCGGACACACCGCCAACGCTCAGTGCGGTGCTACCGACCCGAGGTCAAGTCCCGGGGGAGGAGAAGAGAGGCTTCCCG-CCTAGAGCATTTGCAAGTCAGGATTCTCTAATCCCTCTGGGAGAAGGGTATTCGGCTTGTCCGCTGT-TTTT

>KX125051 Human gammaherpesvirus 4 isolate GC-EBV2

AGGACAGCCGTTGCCCTAGTGGTTTCGGACACACCGCCAACGCTCAGTGCGGTGCTACCGACCCGAGGTCAAGTCCCGGGGGAGGAGAAGAGAGGCTTCCCG-CCTAGAGCATTTGCAAGTCAGGATTCTCTAATCCCTCTGGGAGAAGGGTATTCGGCTTGTCCGCTGT-TTTT

>KX125052 Human gammaherpesvirus 4 isolate SNU-719

AGGACAGCCGTTGCCCTAGTGGTTTCGGACACACCGCCAACGCTCAGTGCGGTGCTACCGACCCGAGGTCAAGTCCCGGGGGAGGAGAAGAGAGGCTTCCCG-CCTAGAGCATTTGCAAGTCAGGATTCTCTAATCCCTCTGGGAGAAGGGTATTCGGCTTGTCCGCTGT-TTTT

>KX674065 Human gammaherpesvirus 4 isolate GDGC2

AGGACAGCCGTTGCCCTAGTGGTTTCGGACACACCGCCAACGCTCAGTGCGGTGCTACCGACCCGAGGTCAAGTCCCGGGGGAGGAGAAGAGAGGCTTCCCG-CCTAGAGCATTTGCAAGTCAGGATTCTCTAATCCCTCTGGGAGAAGGGTATTCGGCTTGTCCGCTGT-TTTT

>LC573550 Human gammaherpesvirus 4 sLCL-T2.27 DNA, complete genome

AGGACAGCCGTTGCCCTAGTGGTTTCGGACACACCGCCAACGCTCAGTGCGGTGCTACCGACCCGAGGTCAAGTCCCGGGGGAGGAGAAGAGAGGCTTCCCG-CCTAGAGCATTTGCAAGTCAGGATTCTCTAATCCCTCTGGGAGAAGGGTATTCGGCTTGTCCGCTGT-TTTT

>LC573551 Human gammaherpesvirus 4 sLCL-T3.27 DNA, complete genome

AGGACAGCCGTTGCCCTAGTGGTTTCGGACACACCGCCAACGCTCAGTGCGGTGCTACCGACCCGAGGTCAAGTCCCGGGGGAGGAGAAGAGAGGCTTCCCG-CCTAGAGCATTTGCAAGTCAGGATTCTCTAATCCCTCTGGGAGAAGGGTATTCGGCTTGTCCGCTGT-TTTT

>LC573552 Human gammaherpesvirus 4 sLCL-T12.18 DNA, complete genome

AGGACAGCCGTTGCCCTAGTGGTTTCGGACACACCGCCAACGCTCAGTGCGGTGCTACCGACCCGAGGTCAAGTCCCGGGGGAGGAGAAGAGAGGCTTCCCG-CCTAGAGCATTTGCAAGTCAGGATTCTCTAATCCCTCTGGGAGAAGGGTATTCGGCTTGTCCGCTGT-TTTT

>LC573553 Human gammaherpesvirus 4 sLCL-T12.20 DNA, complete genome

AGGACAGCCGTTGCCCTAGTGGTTTCGGACACACCGCCAACGCTCAGTGCGGTGCTACCGACCCGAGGTCAAGTCCCGGGGGAGGAGAAGAGAGGCTTCCCG-CCTAGAGCATTTGCAAGTCAGGATTCTCTAATCCCTCTGGGAGAAGGGTATTCGGCTTGTCCGCTGT-TTTT

>LC573554 Human gammaherpesvirus 4 sLCL-T12.22 DNA, complete genome

AGGACAGCCGTTGCCCTAGTGGTTTCGGACACACCGCCAACGCTCAGTGCGGTGCTACCGACCCGAGGTCAAGTCCCGGGGGAGGAGAAGAGAGGCTTCCCG-CCTAGAGCATTTGCAAGTCAGGATTCTCTAATCCCTCTGGGAGAAGGGTATTCGGCTTGTCCGCTGT-TTTT

>LC573555 Human gammaherpesvirus 4 sLCL-T12.11 DNA, complete genome

AGGACAGCCGTTGCCCTAGTGGTTTCGGACACACCGCCAACGCTCAGTGCGGTGCTACCGACCCGAGGTCAAGTCCCGGGGGAGGAGAAGAGAGGCTTCCCG-CCTAGAGCATTTGCAAGTCAGGATTCTCTAATCCCTCTGGGAGAAGGGTATTCGGCTTGTCCGCTGT-TTTT

>LC573556 Human gammaherpesvirus 4 sLCL-T1.12 DNA, complete genome

AGGACAGCCGTTGCCCTAGTGGTTTCGGACACACCGCCAACGCTCAGTGCGGTGCTACCGACCCGAGGTCAAGTCCCGGGGGAGGAGAAGAGAGGCTTCCCG-CCTAGAGCATTTGCAAGTCAGGATTCTCTAATCCCTCTGGGAGAAGGGTATTCGGCTTGTCCGCTGT-TTTT

>LN824142 Human herpesvirus 4 genome assembly Saliva1_assembly, segment : I

AGGACAGCCGTTGCCCTAGTGGTTTCGGACACACCGCCAACGCTCAGTGCGGTGCTACCGACCCGAGGTCAAGTCCCGGGGGAGGAGAAGAGAGGCTTCCCG-CCTAGAGCATTTGCAAGTCAGGATTCTCTAATCCCTCTGGGAGAAGGGTATTCGGCTTGTCCGCTGT-TTTT

>LN824203 Human herpesvirus 4 genome assembly Mak_1, segment : I

AGGACAGCCGTTGCCCTAGTGGTTTCGGACACACCGCCAACGCTCAGTGCGGTGCTACCGACCCGAGGTCAAGTCCCGGGGGAGGAGAAGAGAGGCTTCCCG-CCTAGAGCATTTGCAAGTCAGGATTCTCTAATCCCTCTGGGAGAAGGGTATTCGGCTTGTCCGCTGT-TTTT

>LN824204 Human herpesvirus 4 genome assembly HL05, segment : I

AGGACAGCCGTTGCCCTAGTGGTTTCGGACACACCGCCAACGCTCAGTGCGGTGCTACCGACCCGAGGTCAAGTCCCGGGGGAGGAGAAGAGAGGCTTCCCG-CCTAGAGCATTTGCAAGTCAGGATTCTCTAATCCCTCTGGGAGAAGGGTATTCGGCTTGTCCGCTGT-TTTT

>LN824205 Human herpesvirus 4 genome assembly sLCL-1.12, segment : I

AGGACAGCCGTTGCCCTAGTGGTTTCGGACACACCGCCAACGCTCAGTGCGGTGCTACCGACCCGAGGTCAAGTCCCGGGGGAGGAGAAGAGAGGCTTCCCG-CCTAGAGCATTTGCAAGTCAGGATTCTCTAATCCCTCTGGGAGAAGGGTATTCGGCTTGTCCGCTGT-TTTT

>LN824206 Human herpesvirus 4 genome assembly pLCL-TRL1-post

AGGACAGCCGTTGCCCTAGTGGTTTCGGACACACCGCCAACGCTCAGTGCGGTGCTACCGACCCGAGGTCAAGTCCCGGGGGAGGAGAAGAGAGGCTTCCCG-CCTAGAGCATTTGCAAGTCAGGATTCTCTAATCCCTCTGGGAGAAGGGTATTCGGCTTGTCCGCTGT-TTTT

>LN824207 Human herpesvirus 4 genome assembly pLCL-TRL1-pre, segment : I

AGGACAGCCGTTGCCCTAGTGGTTTCGGACACACCGCCAACGCTCAGTGCGGTGCTACCGACCCGAGGTCAAGTCCCGGGGGAGGAGAAGAGAGGCTTCCCG-CCTAGAGCATTTGCAAGTCAGGATTCTCTAATCCCTCTGGGAGAAGGGTATTCGGCTTGTCCGCTGT-TTTT

>LN824208 Human herpesvirus 4 genome assembly Akata, segment : I

AGGACAGCCGTTGCCCTAGTGGTTTCGGACACACCGCCAACGCTCAGTGCGGTGCTACCGACCCGAGGTCAAGTCCCGGGGGAGGAGAAGAGAGGCTTCCCG-CCTAGAGCATTTGCAAGTCAGGATTCTCTAATCCCTCTGGGAGAAGGGTATTCGGCTTGTCCGCTGT-TTTT

>LN824209 Human herpesvirus 4 genome assembly HKN14, segment : I

AGGACAGCCGTTGCCCTAGTGGTTTCGGACACACCGCCAACGCTCAGTGCGGTGCTACCGACCCGAGGTCAAGTCCCGGGGGAGGAGAAGAGAGGCTTCCCG-CCTAGAGCATTTGCAAGTCAGGATTCTCTAATCCCTCTGGGAGAAGGGTATTCGGCTTGTCCGCTGT-TTTT

>LN824225 Human herpesvirus 4 genome assembly HL08, segment : I

AGGACAGCCGTTGCCCTAGTGGTTTCGGACACACCGCCAACGCTCAGTGCGGTGCTACCGACCCGAGGTCAAGTCCCGGGGGAGGAGAAGAGAGGCTTCCCG-CCTAGAGCATTTGCAAGTCAGGATTCTCTAATCCCTCTGGGAGAAGGGTATTCGGCTTGTCCGCTGT-TTTT

>LN824226 Human herpesvirus 4 genome assembly HL01, segment : I

AGGACAGCCGTTGCCCTAGTGGTTTCGGACACACCGCCAACGCTCAGTGCGGTGCTACCGACCCGAGGTCAAGTCCCGGGGGAGGAGAAGAGAGGCTTCCCG-CCTAGAGCATTTGCAAGTCAGGATTCTCTAATCCCTCTGGGAGAAGGGTATTCGGCTTGTCCGCTGT-TTTT

>LN827522 Human herpesvirus 4 genome assembly HL09, segment : I

AGGACAGCCGTTGCCCTAGTGGTTTCGGACACACCGCCAACGCTCAGTGCGGTGCTACCGACCCGAGGTCAAGTCCCGGGGGAGGAGAAGAGAGGCTTCCCG-CCTAGAGCATTTGCAAGTCAGGATTCTCTAATCCCTCTGGGAGAAGGGTATTCGGCTTGTCCGCTGT-TTTT

>LN827523 Human herpesvirus 4 genome assembly L591, segment : I

AGGACAGCCGTTGCCCTAGTGGTTTCGGACACACCGCCAACGCTCAGTGCGGTGCTACCGACCCGAGGTCAAGTCCCGGGGGAGGAGAAGAGAGGCTTCCCG-CCTAGAGCATTTGCAAGTCAGGATTCTCTAATCCCTCTGGGAGAAGGGTATTCGGCTTGTCCGCTGT-TTTT

>LN827524 Human herpesvirus 4 genome assembly HL11, segment : I

AGGACAGCCGTTGCCCTAGTGGTTTCGGACACACCGCCAACGCTCAGTGCGGTGCTACCGACCCGAGGTCAAGTCCCGGGGGAGGAGAAGAGAGGCTTCCCG-CCTAGAGCATTTGCAAGTCAGGATTCTCTAATCCCTCTGGGAGAAGGGTATTCGGCTTGTCCGCTGT-TTTT

>LN827544 Human herpesvirus 4 strain Wewak_2 genome assembly, segment: I

AGGACAGCCGTTGCCCTAGTGGTTTCGGACACACCGCCAACGCTCAGTGCGGTGCTACCGACCCGAGGTCAAGTCCCGGGGGAGGAGAAGAGAGGCTTCCCG-CCTAGAGCATTTGCAAGTCAGGATTCTCTAATCCCTCTGGGAGAAGGGTATTCGGCTTGTCCGCTGT-TTTT

>LN827545 Human herpesvirus 4 genome assembly Daudi, segment : I

AGGACAGCCGTTGCCCTAGTGGTTTCGGACACACCGCCAACGCTCAGTGCGGTGCTACCGACCCGAGGTCAAGTCCCGGGGGAGGAGAAGAGAGGCTTCCCG-CCTAGAGCATTTGCAAGTCAGGATTCTCTAATCCCTCTGGGAGAAGGGTATTCGGCTTGTCCGCTGT-TTTT

>LN827546 Human herpesvirus 4 genome assembly HL02, segment : I

AGGACAGCCGTTGCCCTAGTGGTTTCGGACACACCGCCAACGCTCAGTGCGGTGCTACCGACCCGAGGTCAAGTCCCGGGGGAGGAGAAGAGAGGCTTCCCG-CCTAGAGCATTTGCAAGTCAGGATTCTCTAATCCCTCTGGGAGAAGGGTATTCGGCTTGTCCGCTGT-TTTT

>LN827548 Human herpesvirus 4 genome assembly P3HR1_c16 Assembly, segment : I

AGGACAGCCGTTGCCCTAGTGGTTTCGGACACACCGCCAACGCTCAGTGCGGTGCTACCGACCCGAGGTCAAGTCCCGGGGGAGGAGAAGAGAGGCTTCCCG-CCTAGAGCATTTGCAAGTCAGGATTCTCTAATCCCTCTGGGAGAAGGGTATTCGGCTTGTCCGCTGT-TTTT

>LN827551 Human herpesvirus 4 genome assembly Makau, segment : I

AGGACAGCCGTTGCCCTAGTGGTTTCGGACACACCGCCAACGCTCAGTGCGGTGCTACCGACCCGAGGTCAAGTCCCGGGGGAGGAGAAGAGAGGCTTCCCG-CCTAGAGCATTTGCAAGTCAGGATTCTCTAATCCCTCTGGGAGAAGGGTATTCGGCTTGTCCGCTGT-TTTT

>LN827552 Human herpesvirus 4 genome assembly sLCL-1.08, segment : I

AGGACAGCCGTTGCCCTAGTGGTTTCGGACACACCGCCAACGCTCAGTGCGGTGCTACCGACCCGAGGTCAAGTCCCGGGGGAGGAGAAGAGAGGCTTCCCG-CCTAGAGCATTTGCAAGTCAGGATTCTCTAATCCCTCTGGGAGAAGGGTATTCGGCTTGTCCGCTGT-TTTT

>LN827553 Human herpesvirus 4 genome assembly sLCL-IS1.08, segment : I

AGGACAGCCGTTGCCCTAGTGGTTTCGGACACACCGCCAACGCTCAGTGCGGTGCTACCGACCCGAGGTCAAGTCCCGGGGGAGGAGAAGAGAGGCTTCCCG-CCTAGAGCATTTGCAAGTCAGGATTCTCTAATCCCTCTGGGAGAAGGGTATTCGGCTTGTCCGCTGT-TTTT

>LN827554 Human herpesvirus 4 genome assembly AFB1b, segment : I

AGGACAGCCGTTGCCCTAGTGGTTTCGGACACACCGCCAACGCTCAGTGCGGTGCTACCGACCCGAGGTCAAGTCCCGGGGGAGGAGAAGAGAGGCTTCCCG-CCTAGAGCATTTGCAAGTCAGGATTCTCTAATCCCTCTGGGAGAAGGGTATTCGGCTTGTCCGCTGT-TTTT

>LN827559 Human herpesvirus 4 genome assembly pLCL-TRL595, segment : I

AGGACAGCCGTTGCCCTAGTGGTTTCGGACACACCGCCAACGCTCAGTGCGGTGCTACCGACCCGAGGTCAAGTCCCGGGGGAGGAGAAGAGAGGCTTCCCG-CCTAGAGCATTTGCAAGTCAGGATTCTCTAATCCCTCTGGGAGAAGGGTATTCGGCTTGTCCGCTGT-TTTT

>LN827560 Human herpesvirus 4 genome assembly sLCL-2.14, segment : I

AGGACAGCCGTTGCCCTAGTGGTTTCGGACACACCGCCAACGCTCAGTGCGGTGCTACCGACCCGAGGTCAAGTCCCGGGGGAGGAGAAGAGAGGCTTCCCG-CCTAGAGCATTTGCAAGTCAGGATTCTCTAATCCCTCTGGGAGAAGGGTATTCGGCTTGTCCGCTGT-TTTT

>LN827562 Human herpesvirus 4 genome assembly sLCL-1.19, segment : I

AGGACAGCCGTTGCCCTAGTGGTTTCGGACACACCGCCAACGCTCAGTGCGGTGCTACCGACCCGAGGTCAAGTCCCGGGGGAGGAGAAGAGAGGCTTCCCG-CCTAGAGCATTTGCAAGTCAGGATTCTCTAATCCCTCTGGGAGAAGGGTATTCGGCTTGTCCGCTGT-TTTT

>LN827563 Human herpesvirus 4 genome assembly sLCL-1.18, segment : I

AGGACAGCCGTTGCCCTAGTGGTTTCGGACACACCGCCAACGCTCAGTGCGGTGCTACCGACCCGAGGTCAAGTCCCGGGGGAGGAGAAGAGAGGCTTCCCG-CCTAGAGCATTTGCAAGTCAGGATTCTCTAATCCCTCTGGGAGAAGGGTATTCGGCTTGTCCGCTGT-TTTT

>LN827564 Human herpesvirus 4 genome assembly HL04, segment : I

AGGACAGCCGTTGCCCTAGTGGTTTCGGACACACCGCCAACGCTCAGTGCGGTGCTACCGACCCGAGGTCAAGTCCCGGGGGAGGAGAAGAGAGGCTTCCCG-CCTAGAGCATTTGCAAGTCAGGATTCTCTAATCCCTCTGGGAGAAGGGTATTCGGCTTGTCCGCTGT-TTTT

>LN827565 Human herpesvirus 4 genome assembly sLCL-1.07, segment : I

AGGACAGCCGTTGCCCTAGTGGTTTCGGACACACCGCCAACGCTCAGTGCGGTGCTACCGACCCGAGGTCAAGTCCCGGGGGAGGAGAAGAGAGGCTTCCCG-CCTAGAGCATTTGCAAGTCAGGATTCTCTAATCCCTCTGGGAGAAGGGTATTCGGCTTGTCCGCTGT-TTTT

>LN827566 Human herpesvirus 4 genome assembly sLCL-1.06, segment : I

AGGACAGCCGTTGCCCTAGTGGTTTCGGACACACCGCCAACGCTCAGTGCGGTGCTACCGACCCGAGGTCAAGTCCCGGGGGAGGAGAAGAGAGGCTTCCCG-CCTAGAGCATTTGCAAGTCAGGATTCTCTAATCCCTCTGGGAGAAGGGTATTCGGCTTGTCCGCTGT-TTTT

>LN827567 Human herpesvirus 4 genome assembly sLCL-IM1.09, segment : I

AGGACAGCCGTTGCCCTAGTGGTTTCGGACACACCGCCAACGCTCAGTGCGGTGCTACCGACCCGAGGTCAAGTCCCGGGGGAGGAGAAGAGAGGCTTCCCG-CCTAGAGCATTTGCAAGTCAGGATTCTCTAATCCCTCTGGGAGAAGGGTATTCGGCTTGTCCGCTGT-TTTT

>LN827568 Human herpesvirus 4 genome assembly sLCL-1.24, segment : I

AGGACAGCCGTTGCCCTAGTGGTTTCGGACACACCGCCAACGCTCAGTGCGGTGCTACCGACCCGAGGTCAAGTCCCGGGGGAGGAGAAGAGAGGCTTCCCG-CCTAGAGCATTTGCAAGTCAGGATTCTCTAATCCCTCTGGGAGAAGGGTATTCGGCTTGTCCGCTGT-TTTT

>LN827569 Human herpesvirus 4 genome assembly sLCL-IS1.11, segment : I

AGGACAGCCGTTGCCCTAGTGGTTTCGGACACACCGCCAACGCTCAGTGCGGTGCTACCGACCCGAGGTCAAGTCCCGGGGGAGGAGAAGAGAGGCTTCCCG-CCTAGAGCATTTGCAAGTCAGGATTCTCTAATCCCTCTGGGAGAAGGGTATTCGGCTTGTCCGCTGT-TTTT

>LN827570 Human herpesvirus 4 genome assembly sLCL-IS1.01, segment : I

AGGACAGCCGTTGCCCTAGTGGTTTCGGACACACCGCCAACGCTCAGTGCGGTGCTACCGACCCGAGGTCAAGTCCCGGGGGAGGAGAAGAGAGGCTTCCCG-CCTAGAGCATTTGCAAGTCAGGATTCTCTAATCCCTCTGGGAGAAGGGTATTCGGCTTGTCCGCTGT-TTTT

>LN827571 Human herpesvirus 4 genome assembly sLCL-BL1.20, segment : I

AGGACAGCCGTTGCCCTAGTGGTTTCGGACACACCGCCAACGCTCAGTGCGGTGCTACCGACCCGAGGTCAAGTCCCGGGGGAGGAGAAGAGAGGCTTCCCG-CCTAGAGCATTTGCAAGTCAGGATTCTCTAATCCCTCTGGGAGAAGGGTATTCGGCTTGTCCGCTGT-TTTT

>LN827573 Human herpesvirus 4 genome assembly sLCL-1.10, segment : I

AGGACAGCCGTTGCCCTAGTGGTTTCGGACACACCGCCAACGCTCAGTGCGGTGCTACCGACCCGAGGTCAAGTCCCGGGGGAGGAGAAGAGAGGCTTCCCG-CCTAGAGCATTTGCAAGTCAGGATTCTCTAATCCCTCTGGGAGAAGGGTATTCGGCTTGTCCGCTGT-TTTT

>LN827574 Human herpesvirus 4 genome assembly sLCL-1.09, segment : I

AGGACAGCCGTTGCCCTAGTGGTTTCGGACACACCGCCAACGCTCAGTGCGGTGCTACCGACCCGAGGTCAAGTCCCGGGGGAGGAGAAGAGAGGCTTCCCG-CCTAGAGCATTTGCAAGTCAGGATTCTCTAATCCCTCTGGGAGAAGGGTATTCGGCTTGTCCGCTGT-TTTT

>LN827575 Human herpesvirus 4 genome assembly sLCL-IS1.14, segment : I

AGGACAGCCGTTGCCCTAGTGGTTTCGGACACACCGCCAACGCTCAGTGCGGTGCTACCGACCCGAGGTCAAGTCCCGGGGGAGGAGAAGAGAGGCTTCCCG-CCTAGAGCATTTGCAAGTCAGGATTCTCTAATCCCTCTGGGAGAAGGGTATTCGGCTTGTCCGCTGT-TTTT

>LN827576 Human herpesvirus 4 genome assembly sLCL-IS1.20, segment : I

AGGACAGCCGTTGCCCTAGTGGTTTCGGACACACCGCCAACGCTCAGTGCGGTGCTACCGACCCGAGGTCAAGTCCCGGGGGAGGAGAAGAGAGGCTTCCCG-CCTAGAGCATTTGCAAGTCAGGATTCTCTAATCCCTCTGGGAGAAGGGTATTCGGCTTGTCCGCTGT-TTTT

>LN827577 Human herpesvirus 4 genome assembly sLCL-1.17, segment : I

AGGACAGCCGTTGCCCTAGTGGTTTCGGACACACCGCCAACGCTCAGTGCGGTGCTACCGACCCGAGGTCAAGTCCCGGGGGAGGAGAAGAGAGGCTTCCCG-CCTAGAGCATTTGCAAGTCAGGATTCTCTAATCCCTCTGGGAGAAGGGTATTCGGCTTGTCCGCTGT-TTTT

>LN827578 Human herpesvirus 4 genome assembly sLCL-IS1.13, chromosome : I

AGGACAGCCGTTGCCCTAGTGGTTTCGGACACACCGCCAACGCTCAGTGCGGTGCTACCGACCCGAGGTCAAGTCCCGGGGGAGGAGAAGAGAGGCTTCCCG-CCTAGAGCATTTGCAAGTCAGGATTCTCTAATCCCTCTGGGAGAAGGGTATTCGGCTTGTCCGCTGT-TTTT

>LN827579 Human herpesvirus 4 genome assembly sLCL-1.13, segment : I

AGGACAGCCGTTGCCCTAGTGGTTTCGGACACACCGCCAACGCTCAGTGCGGTGCTACCGACCCGAGGTCAAGTCCCGGGGGAGGAGAAGAGAGGCTTCCCG-CCTAGAGCATTTGCAAGTCAGGATTCTCTAATCCCTCTGGGAGAAGGGTATTCGGCTTGTCCGCTGT-TTTT

>LN827580 Human herpesvirus 4 genome assembly sLCL-2.16, segment : I

AGGACAGCCGTTGCCCTAGTGGTTTCGGACACACCGCCAACGCTCAGTGCGGTGCTACCGACCCGAGGTCAAGTCCCGGGGGAGGAGAAGAGAGGCTTCCCG-CCTAGAGCATTTGCAAGTCAGGATTCTCTAATCCCTCTGGGAGAAGGGTATTCGGCTTGTCCGCTGT-TTTT

>LN827581 Human herpesvirus 4 genome assembly sLCL-1.05, segment : I

AGGACAGCCGTTGCCCTAGTGGTTTCGGACACACCGCCAACGCTCAGTGCGGTGCTACCGACCCGAGGTCAAGTCCCGGGGGAGGAGAAGAGAGGCTTCCCG-CCTAGAGCATTTGCAAGTCAGGATTCTCTAATCCCTCTGGGAGAAGGGTATTCGGCTTGTCCGCTGT-TTTT

>LN827584 Human herpesvirus 4 genome assembly sLCL-IS1.06, segment : I

AGGACAGCCGTTGCCCTAGTGGTTTCGGACACACCGCCAACGCTCAGTGCGGTGCTACCGACCCGAGGTCAAGTCCCGGGGGAGGAGAAGAGAGGCTTCCCG-CCTAGAGCATTTGCAAGTCAGGATTCTCTAATCCCTCTGGGAGAAGGGTATTCGGCTTGTCCGCTGT-TTTT

>LN827585 Human herpesvirus 4 genome assembly sLCL-1.04, segment : I

AGGACAGCCGTTGCCCTAGTGGTTTCGGACACACCGCCAACGCTCAGTGCGGTGCTACCGACCCGAGGTCAAGTCCCGGGGGAGGAGAAGAGAGGCTTCCCG-CCTAGAGCATTTGCAAGTCAGGATTCTCTAATCCCTCTGGGAGAAGGGTATTCGGCTTGTCCGCTGT-TTTT

>LN827586 Human herpesvirus 4 genome assembly sLCL-IS1.15, segment : I

AGGACAGCCGTTGCCCTAGTGGTTTCGGACACACCGCCAACGCTCAGTGCGGTGCTACCGACCCGAGGTCAAGTCCCGGGGGAGGAGAAGAGAGGCTTCCCG-CCTAGAGCATTTGCAAGTCAGGATTCTCTAATCCCTCTGGGAGAAGGGTATTCGGCTTGTCCGCTGT-TTTT

>LN827588 Human herpesvirus 4 genome assembly sLCL-IS1.19, segment : I

AGGACAGCCGTTGCCCTAGTGGTTTCGGACACACCGCCAACGCTCAGTGCGGTGCTACCGACCCGAGGTCAAGTCCCGGGGGAGGAGAAGAGAGGCTTCCCG-CCTAGAGCATTTGCAAGTCAGGATTCTCTAATCCCTCTGGGAGAAGGGTATTCGGCTTGTCCGCTGT-TTTT

>LN827589 Human herpesvirus 4 genome assembly sLCL-IS2.01, segment : I

AGGACAGCCGTTGCCCTAGTGGTTTCGGACACACCGCCAACGCTCAGTGCGGTGCTACCGACCCGAGGTCAAGTCCCGGGGGAGGAGAAGAGAGGCTTCCCG-CCTAGAGCATTTGCAAGTCAGGATTCTCTAATCCCTCTGGGAGAAGGGTATTCGGCTTGTCCGCTGT-TTTT

>LN827590 Human herpesvirus 4 genome assembly sLCL-IM1.05, segment : I

AGGACAGCCGTTGCCCTAGTGGTTTCGGACACACCGCCAACGCTCAGTGCGGTGCTACCGACCCGAGGTCAAGTCCCGGGGGAGGAGAAGAGAGGCTTCCCG-CCTAGAGCATTTGCAAGTCAGGATTCTCTAATCCCTCTGGGAGAAGGGTATTCGGCTTGTCCGCTGT-TTTT

>LN827591 Human herpesvirus 4 genome assembly sLCL-2.15, segment : I

AGGACAGCCGTTGCCCTAGTGGTTTCGGACACACCGCCAACGCTCAGTGCGGTGCTACCGACCCGAGGTCAAGTCCCGGGGGAGGAGAAGAGAGGCTTCCCG-CCTAGAGCATTTGCAAGTCAGGATTCTCTAATCCCTCTGGGAGAAGGGTATTCGGCTTGTCCGCTGT-TTTT

>LN827592 Human herpesvirus 4 genome assembly sLCL-IS1.10, segment : I

AGGACAGCCGTTGCCCTAGTGGTTTCGGACACACCGCCAACGCTCAGTGCGGTGCTACCGACCCGAGGTCAAGTCCCGGGGGAGGAGAAGAGAGGCTTCCCG-CCTAGAGCATTTGCAAGTCAGGATTCTCTAATCCCTCTGGGAGAAGGGTATTCGGCTTGTCCGCTGT-TTTT

>LN827593 Human herpesvirus 4 genome assembly sLCL-IS1.12, segment : I

AGGACAGCCGTTGCCCTAGTGGTTTCGGACACACCGCCAACGCTCAGTGCGGTGCTACCGACCCGAGGTCAAGTCCCGGGGGAGGAGAAGAGAGGCTTCCCG-CCTAGAGCATTTGCAAGTCAGGATTCTCTAATCCCTCTGGGAGAAGGGTATTCGGCTTGTCCGCTGT-TTTT

>LN827594 Human herpesvirus 4 genome assembly sLCL-IS1.07, segment : I

AGGACAGCCGTTGCCCTAGTGGTTTCGGACACACCGCCAACGCTCAGTGCGGTGCTACCGACCCGAGGTCAAGTCCCGGGGGAGGAGAAGAGAGGCTTCCCG-CCTAGAGCATTTGCAAGTCAGGATTCTCTAATCCCTCTGGGAGAAGGGTATTCGGCTTGTCCGCTGT-TTTT

>LN827595 Human herpesvirus 4 genome assembly sLCL-IS1.03, segment : I

AGGACAGCCGTTGCCCTAGTGGTTTCGGACACACCGCCAACGCTCAGTGCGGTGCTACCGACCCGAGGTCAAGTCCCGGGGGAGGAGAAGAGAGGCTTCCCG-CCTAGAGCATTTGCAAGTCAGGATTCTCTAATCCCTCTGGGAGAAGGGTATTCGGCTTGTCCGCTGT-TTTT

>LN827596 Human herpesvirus 4 genome assembly sLCL-IM1.02, segment : I

AGGACAGCCGTTGCCCTAGTGGTTTCGGACACACCGCCAACGCTCAGTGCGGTGCTACCGACCCGAGGTCAAGTCCCGGGGGAGGAGAAGAGAGGCTTCCCG-CCTAGAGCATTTGCAAGTCAGGATTCTCTAATCCCTCTGGGAGAAGGGTATTCGGCTTGTCCGCTGT-TTTT

>LN827799 Human herpesvirus 4 genome assembly sLCL-IM1.16, segment : I

AGGACAGCCGTTGCCCTAGTGGTTTCGGACACACCGCCAACGCTCAGTGCGGTGCTACCGACCCGAGGTCAAGTCCCGGGGGAGGAGAAGAGAGGCTTCCCG-CCTAGAGCATTTGCAAGTCAGGATTCTCTAATCCCTCTGGGAGAAGGGTATTCGGCTTGTCCGCTGT-TTTT

>LN827800 Human herpesvirus 4 genome assembly Jijoye, segment : I

AGGACAGCCGTTGCCCTAGTGGTTTCGGACACACCGCCAACGCTCAGTGCGGTGCTACCGACCCGAGGTCAAGTCCCGGGGGAGGAGAAGAGAGGCTTCCCG-CCTAGAGCATTTGCAAGTCAGGATTCTCTAATCCCTCTGGGAGAAGGGTATTCGGCTTGTCCGCTGT-TTTT

>LN831023 Human herpesvirus 4 genome assembly sLCL-2.22, segment : I

AGGACAGCCGTTGCCCTAGTGGTTTCGGACACACCGCCAACGCTCAGTGCGGTGCTACCGACCCGAGGTCAAGTCCCGGGGGAGGAGAAGAGAGGCTTCCCG-CCTAGAGCATTTGCAAGTCAGGATTCTCTAATCCCTCTGGGAGAAGGGTATTCGGCTTGTCCGCTGT-TTTT

>LR812977 Human gammaherpesvirus 4 isolate eBL_CL-03 genome assembly, chromosome: EBV

AGGACAGCCGTTGCCCTAGTGGTTTCGGACACACCGCCAACGCTCAGTGCGGTGCTACCGACCCGAGGTCAAGTCCCGGGGGAGGAGAAGAGAGGCTTCCCG-CCTAGAGCATTTGCAAGTCAGGATTCTCTAATCCCTCTGGGAGAAGGGTATTCGGCTTGTCCGCTGT-TTTT

>LR812978 Human gammaherpesvirus 4 isolate eBL_CL-02 genome assembly, chromosome: EBV

AGGACAGCCGTTGCCCTAGTGGTTTCGGACACACCGCCAACGCTCAGTGCGGTGCTACCGACCCGAGGTCAAGTCCCGGGGGAGGAGAAGAGAGGCTTCCCG-CCTAGAGCATTTGCAAGTCAGGATTCTCTAATCCCTCTGGGAGAAGGGTATTCGGCTTGTCCGCTGT-TTTT

>LR812979 Human gammaherpesvirus 4 isolate HC-0004 genome assembly, chromosome: EBV

AGGACAGCCGTTGCCCTAGTGGTTTCGGACACACCGCCAACGCTCAGTGCGGTGCTACCGACCCGAGGTCAAGTCCCGGGGGAGGAGAAGAGAGGCTTCCCG-CCTAGAGCATTTGCAAGTCAGGATTCTCTAATCCCTCTGGGAGAAGGGTATTCGGCTTGTCCGCTGT-TTTT

>LR812980 Human gammaherpesvirus 4 isolate HC-0001 genome assembly, chromosome: EBV

AGGACAGCCGTTGCCCTAGTGGTTTCGGACACACCGCCAACGCTCAGTGCGGTGCTACCGACCCGAGGTCAAGTCCCGGGGGAGGAGAAGAGAGGCTTCCCG-CCTAGAGCATTTGCAAGTCAGGATTCTCTAATCCCTCTGGGAGAAGGGTATTCGGCTTGTCCGCTGT-TTTT

>LR812981 Human gammaherpesvirus 4 isolate HC-0002 genome assembly, chromosome: EBV

AGGACAGCCGTTGCCCTAGTGGTTTCGGACACACCGCCAACGCTCAGTGCGGTGCTACCGACCCGAGGTCAAGTCCCGGGGGAGGAGAAGAGAGGCTTCCCG-CCTAGAGCATTTGCAAGTCAGGATTCTCTAATCCCTCTGGGAGAAGGGTATTCGGCTTGTCCGCTGT-TTTT

>LR812982 Human gammaherpesvirus 4 isolate HC-0003 genome assembly, chromosome: EBV

AGGACAGCCGTTGCCCTAGTGGTTTCGGACACACCGCCAACGCTCAGTGCGGTGCTACCGACCCGAGGTCAAGTCCCGGGGGAGGAGAAGAGAGGCTTCCCG-CCTAGAGCATTTGCAAGTCAGGATTCTCTAATCCCTCTGGGAGAAGGGTATTCGGCTTGTCCGCTGT-TTTT

>LR812983 Human gammaherpesvirus 4 isolate HC-0005 genome assembly, chromosome: EBV

AGGACAGCCGTTGCCCTAGTGGTTTCGGACACACCGCCAACGCTCAGTGCGGTGCTACCGACCCGAGGTCAAGTCCCGGGGGAGGAGAAGAGAGGCTTCCCG-CCTAGAGCATTTGCAAGTCAGGATTCTCTAATCCCTCTGGGAGAAGGGTATTCGGCTTGTCCGCTGT-TTTT

>LR812984 Human gammaherpesvirus 4 isolate HC-0007 genome assembly, chromosome: EBV

AGGACAGCCGTTGCCCTAGTGGTTTCGGACACACCGCCAACGCTCAGTGCGGTGCTACCGACCCGAGGTCAAGTCCCGGGGGAGGAGAAGAGAGGCTTCCCG-CCTAGAGCATTTGCAAGTCAGGATTCTCTAATCCCTCTGGGAGAAGGGTATTCGGCTTGTCCGCTGT-TTTT

>LR812987 Human gammaherpesvirus 4 isolate HC-0009 genome assembly, chromosome: EBV

AGGACAGCCGTTGCCCTAGTGGTTTCGGACACACCGCCAACGCTCAGTGCGGTGCTACCGACCCGAGGTCAAGTCCCGGGGGAGGAGAAGAGAGGCTTCCCG-CCTAGAGCATTTGCAAGTCAGGATTCTCTAATCCCTCTGGGAGAAGGGTATTCGGCTTGTCCGCTGT-TTTT

>LR812995 Human gammaherpesvirus 4 isolate HC-0015 genome assembly, chromosome: EBV

AGGACAGCCGTTGCCCTAGTGGTTTCGGACACACCGCCAACGCTCAGTGCGGTGCTACCGACCCGAGGTCAAGTCCCGGGGGAGGAGAAGAGAGGCTTCCCG-CCTAGAGCATTTGCAAGTCAGGATTCTCTAATCCCTCTGGGAGAAGGGTATTCGGCTTGTCCGCTGT-TTTT

>LR812997 Human gammaherpesvirus 4 isolate HC-0019 genome assembly, chromosome: EBV

AGGACAGCCGTTGCCCTAGTGGTTTCGGACACACCGCCAACGCTCAGTGCGGTGCTACCGACCCGAGGTCAAGTCCCGGGGGAGGAGAAGAGAGGCTTCCCG-CCTAGAGCATTTGCAAGTCAGGATTCTCTAATCCCTCTGGGAGAAGGGTATTCGGCTTGTCCGCTGT-TTTT

>LR812998 Human gammaherpesvirus 4 isolate HC-0028 genome assembly, chromosome: EBV

AGGACAGCCGTTGCCCTAGTGGTTTCGGACACACCGCCAACGCTCAGTGCGGTGCTACCGACCCGAGGTCAAGTCCCGGGGGAGGAGAAGAGAGGCTTCCCG-CCTAGAGCATTTGCAAGTCAGGATTCTCTAATCCCTCTGGGAGAAGGGTATTCGGCTTGTCCGCTGT-TTTT

>LR812999 Human gammaherpesvirus 4 isolate HC-0023 genome assembly, chromosome: EBV

AGGACAGCCGTTGCCCTAGTGGTTTCGGACACACCGCCAACGCTCAGTGCGGTGCTACCGACCCGAGGTCAAGTCCCGGGGGAGGAGAAGAGAGGCTTCCCG-CCTAGAGCATTTGCAAGTCAGGATTCTCTAATCCCTCTGGGAGAAGGGTATTCGGCTTGTCCGCTGT-TTTT

>LR813000 Human gammaherpesvirus 4 isolate HC-0026 genome assembly, chromosome: EBV

AGGACAGCCGTTGCCCTAGTGGTTTCGGACACACCGCCAACGCTCAGTGCGGTGCTACCGACCCGAGGTCAAGTCCCGGGGGAGGAGAAGAGAGGCTTCCCG-CCTAGAGCATTTGCAAGTCAGGATTCTCTAATCCCTCTGGGAGAAGGGTATTCGGCTTGTCCGCTGT-TTTT

>LR813001 Human gammaherpesvirus 4 isolate HC-0027 genome assembly, chromosome: EBV

AGGACAGCCGTTGCCCTAGTGGTTTCGGACACACCGCCAACGCTCAGTGCGGTGCTACCGACCCGAGGTCAAGTCCCGGGGGAGGAGAAGAGAGGCTTCCCG-CCTAGAGCATTTGCAAGTCAGGATTCTCTAATCCCTCTGGGAGAAGGGTATTCGGCTTGTCCGCTGT-TTTT

>LR813002 Human gammaherpesvirus 4 isolate HC-0029 genome assembly, chromosome: EBV

AGGACAGCCGTTGCCCTAGTGGTTTCGGACACACCGCCAACGCTCAGTGCGGTGCTACCGACCCGAGGTCAAGTCCCGGGGGAGGAGAAGAGAGGCTTCCCG-CCTAGAGCATTTGCAAGTCAGGATTCTCTAATCCCTCTGGGAGAAGGGTATTCGGCTTGTCCGCTGT-TTTT

>LR813003 Human gammaherpesvirus 4 isolate HC-0024 genome assembly, chromosome: EBV

AGGACAGCCGTTGCCCTAGTGGTTTCGGACACACCGCCAACGCTCAGTGCGGTGCTACCGACCCGAGGTCAAGTCCCGGGGGAGGAGAAGAGAGGCTTCCCG-CCTAGAGCATTTGCAAGTCAGGATTCTCTAATCCCTCTGGGAGAAGGGTATTCGGCTTGTCCGCTGT-TTTT

>LR813007 Human gammaherpesvirus 4 isolate HC-0025 genome assembly, chromosome: EBV

AGGACAGCCGTTGCCCTAGTGGTTTCGGACACACCGCCAACGCTCAGTGCGGTGCTACCGACCCGAGGTCAAGTCCCGGGGGAGGAGAAGAGAGGCTTCCCG-CCTAGAGCATTTGCAAGTCAGGATTCTCTAATCCCTCTGGGAGAAGGGTATTCGGCTTGTCCGCTGT-TTTT

>LR813018 Human gammaherpesvirus 4 isolate eBL-Tumor-0011 genome assembly, chromosome: EBV

AGGACAGCCGTTGCCCTAGTGGTTTCGGACACACCGCCAACGCTCAGTGCGGTGCTACCGACCCGAGGTCAAGTCCCGGGGGAGGAGAAGAGAGGCTTCCCG-CCTAGAGCATTTGCAAGTCAGGATTCTCTAATCCCTCTGGGAGAAGGGTATTCGGCTTGTCCGCTGT-TTTT

>LR813019 Human gammaherpesvirus 4 isolate eBL-Tumor-0012 genome assembly, chromosome: EBV

AGGACAGCCGTTGCCCTAGTGGTTTCGGACACACCGCCAACGCTCAGTGCGGTGCTACCGACCCGAGGTCAAGTCCCGGGGGAGGAGAAGAGAGGCTTCCCG-CCTAGAGCATTTGCAAGTCAGGATTCTCTAATCCCTCTGGGAGAAGGGTATTCGGCTTGTCCGCTGT-TTTT

>LR813020 Human gammaherpesvirus 4 isolate eBL-Tumor-0002 genome assembly, chromosome: EBV

AGGACAGCCGTTGCCCTAGTGGTTTCGGACACACCGCCAACGCTCAGTGCGGTGCTACCGACCCGAGGTCAAGTCCCGGGGGAGGAGAAGAGAGGCTTCCCG-CCTAGAGCATTTGCAAGTCAGGATTCTCTAATCCCTCTGGGAGAAGGGTATTCGGCTTGTCCGCTGT-TTTT

>LR813021 Human gammaherpesvirus 4 isolate eBL-Tumor-0008 genome assembly, chromosome: EBV

AGGACAGCCGTTGCCCTAGTGGTTTCGGACACACCGCCAACGCTCAGTGCGGTGCTACCGACCCGAGGTCAAGTCCCGGGGGAGGAGAAGAGAGGCTTCCCG-CCTAGAGCATTTGCAAGTCAGGATTCTCTAATCCCTCTGGGAGAAGGGTATTCGGCTTGTCCGCTGT-TTTT

>LR813022 Human gammaherpesvirus 4 isolate eBL-Tumor-0010 genome assembly, chromosome: EBV

AGGACAGCCGTTGCCCTAGTGGTTTCGGACACACCGCCAACGCTCAGTGCGGTGCTACCGACCCGAGGTCAAGTCCCGGGGGAGGAGAAGAGAGGCTTCCCG-CCTAGAGCATTTGCAAGTCAGGATTCTCTAATCCCTCTGGGAGAAGGGTATTCGGCTTGTCCGCTGT-TTTT

>LR813023 Human gammaherpesvirus 4 isolate eBL-Tumor-0001 genome assembly, chromosome: EBV

AGGACAGCCGTTGCCCTAGTGGTTTCGGACACACCGCCAACGCTCAGTGCGGTGCTACCGACCCGAGGTCAAGTCCCGGGGGAGGAGAAGAGAGGCTTCCCG-CCTAGAGCATTTGCAAGTCAGGATTCTCTAATCCCTCTGGGAGAAGGGTATTCGGCTTGTCCGCTGT-TTTT

>LR813024 Human gammaherpesvirus 4 isolate eBL-Tumor-0007 genome assembly, chromosome: EBV

AGGACAGCCGTTGCCCTAGTGGTTTCGGACACACCGCCAACGCTCAGTGCGGTGCTACCGACCCGAGGTCAAGTCCCGGGGGAGGAGAAGAGAGGCTTCCCG-CCTAGAGCATTTGCAAGTCAGGATTCTCTAATCCCTCTGGGAGAAGGGTATTCGGCTTGTCCGCTGT-TTTT

>LR813025 Human gammaherpesvirus 4 isolate eBL-Tumor-0003 genome assembly, chromosome: EBV

AGGACAGCCGTTGCCCTAGTGGTTTCGGACACACCGCCAACGCTCAGTGCGGTGCTACCGACCCGAGGTCAAGTCCCGGGGGAGGAGAAGAGAGGCTTCCCG-CCTAGAGCATTTGCAAGTCAGGATTCTCTAATCCCTCTGGGAGAAGGGTATTCGGCTTGTCCGCTGT-TTTT

>LR813026 Human gammaherpesvirus 4 isolate eBL-Tumor-0006 genome assembly, chromosome: EBV

AGGACAGCCGTTGCCCTAGTGGTTTCGGACACACCGCCAACGCTCAGTGCGGTGCTACCGACCCGAGGTCAAGTCCCGGGGGAGGAGAAGAGAGGCTTCCCG-CCTAGAGCATTTGCAAGTCAGGATTCTCTAATCCCTCTGGGAGAAGGGTATTCGGCTTGTCCGCTGT-TTTT

>LR813028 Human gammaherpesvirus 4 isolate eBL-Tumor-0005 genome assembly, chromosome: EBV

AGGACAGCCGTTGCCCTAGTGGTTTCGGACACACCGCCAACGCTCAGTGCGGTGCTACCGACCCGAGGTCAAGTCCCGGGGGAGGAGAAGAGAGGCTTCCCG-CCTAGAGCATTTGCAAGTCAGGATTCTCTAATCCCTCTGGGAGAAGGGTATTCGGCTTGTCCGCTGT-TTTT

>LR813029 Human gammaherpesvirus 4 isolate eBL-Tumor-0004 genome assembly, chromosome: EBV

AGGACAGCCGTTGCCCTAGTGGTTTCGGACACACCGCCAACGCTCAGTGCGGTGCTACCGACCCGAGGTCAAGTCCCGGGGGAGGAGAAGAGAGGCTTCCCG-CCTAGAGCATTTGCAAGTCAGGATTCTCTAATCCCTCTGGGAGAAGGGTATTCGGCTTGTCCGCTGT-TTTT

>LR813030 Human gammaherpesvirus 4 isolate eBL-Tumor-0009 genome assembly, chromosome: EBV

AGGACAGCCGTTGCCCTAGTGGTTTCGGACACACCGCCAACGCTCAGTGCGGTGCTACCGACCCGAGGTCAAGTCCCGGGGGAGGAGAAGAGAGGCTTCCCG-CCTAGAGCATTTGCAAGTCAGGATTCTCTAATCCCTCTGGGAGAAGGGTATTCGGCTTGTCCGCTGT-TTTT

>LR813031 Human gammaherpesvirus 4 isolate eBL-Tumor-0015 genome assembly, chromosome: EBV

AGGACAGCCGTTGCCCTAGTGGTTTCGGACACACCGCCAACGCTCAGTGCGGTGCTACCGACCCGAGGTCAAGTCCCGGGGGAGGAGAAGAGAGGCTTCCCG-CCTAGAGCATTTGCAAGTCAGGATTCTCTAATCCCTCTGGGAGAAGGGTATTCGGCTTGTCCGCTGT-TTTT

>LR813033 Human gammaherpesvirus 4 isolate eBL-Tumor-0013 genome assembly, chromosome: EBV

AGGACAGCCGTTGCCCTAGTGGTTTCGGACACACCGCCAACGCTCAGTGCGGTGCTACCGACCCGAGGTCAAGTCCCGGGGGAGGAGAAGAGAGGCTTCCCG-CCTAGAGCATTTGCAAGTCAGGATTCTCTAATCCCTCTGGGAGAAGGGTATTCGGCTTGTCCGCTGT-TTTT

>LR813034 Human gammaherpesvirus 4 isolate eBL-Tumor-0020 genome assembly, chromosome: EBV

AGGACAGCCGTTGCCCTAGTGGTTTCGGACACACCGCCAACGCTCAGTGCGGTGCTACCGACCCGAGGTCAAGTCCCGGGGGAGGAGAAGAGAGGCTTCCCG-CCTAGAGCATTTGCAAGTCAGGATTCTCTAATCCCTCTGGGAGAAGGGTATTCGGCTTGTCCGCTGT-TTTT

>LR813035 Human gammaherpesvirus 4 isolate eBL-Tumor-0018 genome assembly, chromosome: EBV

AGGACAGCCGTTGCCCTAGTGGTTTCGGACACACCGCCAACGCTCAGTGCGGTGCTACCGACCCGAGGTCAAGTCCCGGGGGAGGAGAAGAGAGGCTTCCCG-CCTAGAGCATTTGCAAGTCAGGATTCTCTAATCCCTCTGGGAGAAGGGTATTCGGCTTGTCCGCTGT-TTTT

>LR813036 Human gammaherpesvirus 4 isolate eBL-Tumor-0023 genome assembly, chromosome: EBV

AGGACAGCCGTTGCCCTAGTGGTTTCGGACACACCGCCAACGCTCAGTGCGGTGCTACCGACCCGAGGTCAAGTCCCGGGGGAGGAGAAGAGAGGCTTCCCG-CCTAGAGCATTTGCAAGTCAGGATTCTCTAATCCCTCTGGGAGAAGGGTATTCGGCTTGTCCGCTGT-TTTT

>LR813037 Human gammaherpesvirus 4 isolate eBL-Tumor-0019 genome assembly, chromosome: EBV

AGGACAGCCGTTGCCCTAGTGGTTTCGGACACACCGCCAACGCTCAGTGCGGTGCTACCGACCCGAGGTCAAGTCCCGGGGGAGGAGAAGAGAGGCTTCCCG-CCTAGAGCATTTGCAAGTCAGGATTCTCTAATCCCTCTGGGAGAAGGGTATTCGGCTTGTCCGCTGT-TTTT

>LR813038 Human gammaherpesvirus 4 isolate eBL-Tumor-0022 genome assembly, chromosome: EBV

AGGACAGCCGTTGCCCTAGTGGTTTCGGACACACCGCCAACGCTCAGTGCGGTGCTACCGACCCGAGGTCAAGTCCCGGGGGAGGAGAAGAGAGGCTTCCCG-CCTAGAGCATTTGCAAGTCAGGATTCTCTAATCCCTCTGGGAGAAGGGTATTCGGCTTGTCCGCTGT-TTTT

>LR813039 Human gammaherpesvirus 4 isolate eBL-Tumor-0016 genome assembly, chromosome: EBV

AGGACAGCCGTTGCCCTAGTGGTTTCGGACACACCGCCAACGCTCAGTGCGGTGCTACCGACCCGAGGTCAAGTCCCGGGGGAGGAGAAGAGAGGCTTCCCG-CCTAGAGCATTTGCAAGTCAGGATTCTCTAATCCCTCTGGGAGAAGGGTATTCGGCTTGTCCGCTGT-TTTT

>LR813040 Human gammaherpesvirus 4 isolate eBL-Tumor-0024 genome assembly, chromosome: EBV

AGGACAGCCGTTGCCCTAGTGGTTTCGGACACACCGCCAACGCTCAGTGCGGTGCTACCGACCCGAGGTCAAGTCCCGGGGGAGGAGAAGAGAGGCTTCCCG-CCTAGAGCATTTGCAAGTCAGGATTCTCTAATCCCTCTGGGAGAAGGGTATTCGGCTTGTCCGCTGT-TTTT

>LR813041 Human gammaherpesvirus 4 isolate eBL-Tumor-0021 genome assembly, chromosome: EBV

AGGACAGCCGTTGCCCTAGTGGTTTCGGACACACCGCCAACGCTCAGTGCGGTGCTACCGACCCGAGGTCAAGTCCCGGGGGAGGAGAAGAGAGGCTTCCCG-CCTAGAGCATTTGCAAGTCAGGATTCTCTAATCCCTCTGGGAGAAGGGTATTCGGCTTGTCCGCTGT-TTTT

>LR813042 Human gammaherpesvirus 4 isolate eBL-Tumor-0017 genome assembly, chromosome: EBV

AGGACAGCCGTTGCCCTAGTGGTTTCGGACACACCGCCAACGCTCAGTGCGGTGCTACCGACCCGAGGTCAAGTCCCGGGGGAGGAGAAGAGAGGCTTCCCG-CCTAGAGCATTTGCAAGTCAGGATTCTCTAATCCCTCTGGGAGAAGGGTATTCGGCTTGTCCGCTGT-TTTT

>LR813044 Human gammaherpesvirus 4 isolate eBL-Tumor-0031 genome assembly, chromosome: EBV

AGGACAGCCGTTGCCCTAGTGGTTTCGGACACACCGCCAACGCTCAGTGCGGTGCTACCGACCCGAGGTCAAGTCCCGGGGGAGGAGAAGAGAGGCTTCCCG-CCTAGAGCATTTGCAAGTCAGGATTCTCTAATCCCTCTGGGAGAAGGGTATTCGGCTTGTCCGCTGT-TTTT

>LR813045 Human gammaherpesvirus 4 isolate eBL-Tumor-0028 genome assembly, chromosome: EBV

AGGACAGCCGTTGCCCTAGTGGTTTCGGACACACCGCCAACGCTCAGTGCGGTGCTACCGACCCGAGGTCAAGTCCCGGGGGAGGAGAAGAGAGGCTTCCCG-CCTAGAGCATTTGCAAGTCAGGATTCTCTAATCCCTCTGGGAGAAGGGTATTCGGCTTGTCCGCTGT-TTTT

>LR813046 Human gammaherpesvirus 4 isolate eBL-Tumor-0025 genome assembly, chromosome: EBV

AGGACAGCCGTTGCCCTAGTGGTTTCGGACACACCGCCAACGCTCAGTGCGGTGCTACCGACCCGAGGTCAAGTCCCGGGGGAGGAGAAGAGAGGCTTCCCG-CCTAGAGCATTTGCAAGTCAGGATTCTCTAATCCCTCTGGGAGAAGGGTATTCGGCTTGTCCGCTGT-TTTT

>LR813047 Human gammaherpesvirus 4 isolate eBL-Tumor-0026 genome assembly, chromosome: EBV

AGGACAGCCGTTGCCCTAGTGGTTTCGGACACACCGCCAACGCTCAGTGCGGTGCTACCGACCCGAGGTCAAGTCCCGGGGGAGGAGAAGAGAGGCTTCCCG-CCTAGAGCATTTGCAAGTCAGGATTCTCTAATCCCTCTGGGAGAAGGGTATTCGGCTTGTCCGCTGT-TTTT

>LR813048 Human gammaherpesvirus 4 isolate eBL-Tumor-0030 genome assembly, chromosome: EBV

AGGACAGCCGTTGCCCTAGTGGTTTCGGACACACCGCCAACGCTCAGTGCGGTGCTACCGACCCGAGGTCAAGTCCCGGGGGAGGAGAAGAGAGGCTTCCCG-CCTAGAGCATTTGCAAGTCAGGATTCTCTAATCCCTCTGGGAGAAGGGTATTCGGCTTGTCCGCTGT-TTTT

>LR813049 Human gammaherpesvirus 4 isolate eBL-Tumor-0027 genome assembly, chromosome: EBV

AGGACAGCCGTTGCCCTAGTGGTTTCGGACACACCGCCAACGCTCAGTGCGGTGCTACCGACCCGAGGTCAAGTCCCGGGGGAGGAGAAGAGAGGCTTCCCG-CCTAGAGCATTTGCAAGTCAGGATTCTCTAATCCCTCTGGGAGAAGGGTATTCGGCTTGTCCGCTGT-TTTT

>LR813050 Human gammaherpesvirus 4 isolate eBL-Tumor-0032 genome assembly, chromosome: EBV

AGGACAGCCGTTGCCCTAGTGGTTTCGGACACACCGCCAACGCTCAGTGCGGTGCTACCGACCCGAGGTCAAGTCCCGGGGGAGGAGAAGAGAGGCTTCCCG-CCTAGAGCATTTGCAAGTCAGGATTCTCTAATCCCTCTGGGAGAAGGGTATTCGGCTTGTCCGCTGT-TTTT

>LR813051 Human gammaherpesvirus 4 isolate eBL-Tumor-0036 genome assembly, chromosome: EBV

AGGACAGCCGTTGCCCTAGTGGTTTCGGACACACCGCCAACGCTCAGTGCGGTGCTACCGACCCGAGGTCAAGTCCCGGGGGAGGAGAAGAGAGGCTTCCCG-CCTAGAGCATTTGCAAGTCAGGATTCTCTAATCCCTCTGGGAGAAGGGTATTCGGCTTGTCCGCTGT-TTTT

>LR813052 Human gammaherpesvirus 4 isolate eBL-Tumor-0033 genome assembly, chromosome: EBV

AGGACAGCCGTTGCCCTAGTGGTTTCGGACACACCGCCAACGCTCAGTGCGGTGCTACCGACCCGAGGTCAAGTCCCGGGGGAGGAGAAGAGAGGCTTCCCG-CCTAGAGCATTTGCAAGTCAGGATTCTCTAATCCCTCTGGGAGAAGGGTATTCGGCTTGTCCGCTGT-TTTT

>LR813053 Human gammaherpesvirus 4 isolate eBL-Tumor-0035 genome assembly, chromosome: EBV

AGGACAGCCGTTGCCCTAGTGGTTTCGGACACACCGCCAACGCTCAGTGCGGTGCTACCGACCCGAGGTCAAGTCCCGGGGGAGGAGAAGAGAGGCTTCCCG-CCTAGAGCATTTGCAAGTCAGGATTCTCTAATCCCTCTGGGAGAAGGGTATTCGGCTTGTCCGCTGT-TTTT

>LR813054 Human gammaherpesvirus 4 isolate eBL-Tumor-0037 genome assembly, chromosome: EBV

AGGACAGCCGTTGCCCTAGTGGTTTCGGACACACCGCCAACGCTCAGTGCGGTGCTACCGACCCGAGGTCAAGTCCCGGGGGAGGAGAAGAGAGGCTTCCCG-CCTAGAGCATTTGCAAGTCAGGATTCTCTAATCCCTCTGGGAGAAGGGTATTCGGCTTGTCCGCTGT-TTTT

>LR813055 Human gammaherpesvirus 4 isolate eBL-Tumor-0034 genome assembly, chromosome: EBV

AGGACAGCCGTTGCCCTAGTGGTTTCGGACACACCGCCAACGCTCAGTGCGGTGCTACCGACCCGAGGTCAAGTCCCGGGGGAGGAGAAGAGAGGCTTCCCG-CCTAGAGCATTTGCAAGTCAGGATTCTCTAATCCCTCTGGGAGAAGGGTATTCGGCTTGTCCGCTGT-TTTT

>LR813056 Human gammaherpesvirus 4 isolate eBL-Tumor-0039 genome assembly, chromosome: EBV

AGGACAGCCGTTGCCCTAGTGGTTTCGGACACACCGCCAACGCTCAGTGCGGTGCTACCGACCCGAGGTCAAGTCCCGGGGGAGGAGAAGAGAGGCTTCCCG-CCTAGAGCATTTGCAAGTCAGGATTCTCTAATCCCTCTGGGAGAAGGGTATTCGGCTTGTCCGCTGT-TTTT

>LR813057 Human gammaherpesvirus 4 isolate eBL-Plasma-0047 genome assembly, chromosome: EBV

AGGACAGCCGTTGCCCTAGTGGTTTCGGACACACCGCCAACGCTCAGTGCGGTGCTACCGACCCGAGGTCAAGTCCCGGGGGAGGAGAAGAGAGGCTTCCCG-CCTAGAGCATTTGCAAGTCAGGATTCTCTAATCCCTCTGGGAGAAGGGTATTCGGCTTGTCCGCTGT-TTTT

>LR813060 Human gammaherpesvirus 4 isolate eBL-Plasma-0042 genome assembly, chromosome: EBV

AGGACAGCCGTTGCCCTAGTGGTTTCGGACACACCGCCAACGCTCAGTGCGGTGCTACCGACCCGAGGTCAAGTCCCGGGGGAGGAGAAGAGAGGCTTCCCG-CCTAGAGCATTTGCAAGTCAGGATTCTCTAATCCCTCTGGGAGAAGGGTATTCGGCTTGTCCGCTGT-TTTT

>LR813061 Human gammaherpesvirus 4 isolate eBL-Plasma-0043 genome assembly, chromosome: EBV

AGGACAGCCGTTGCCCTAGTGGTTTCGGACACACCGCCAACGCTCAGTGCGGTGCTACCGACCCGAGGTCAAGTCCCGGGGGAGGAGAAGAGAGGCTTCCCG-CCTAGAGCATTTGCAAGTCAGGATTCTCTAATCCCTCTGGGAGAAGGGTATTCGGCTTGTCCGCTGT-TTTT

>LR813062 Human gammaherpesvirus 4 isolate eBL-Tumor-0038 genome assembly, chromosome: EBV

AGGACAGCCGTTGCCCTAGTGGTTTCGGACACACCGCCAACGCTCAGTGCGGTGCTACCGACCCGAGGTCAAGTCCCGGGGGAGGAGAAGAGAGGCTTCCCG-CCTAGAGCATTTGCAAGTCAGGATTCTCTAATCCCTCTGGGAGAAGGGTATTCGGCTTGTCCGCTGT-TTTT

>LR813063 Human gammaherpesvirus 4 isolate eBL-Tumor-0040 genome assembly, chromosome: EBV

AGGACAGCCGTTGCCCTAGTGGTTTCGGACACACCGCCAACGCTCAGTGCGGTGCTACCGACCCGAGGTCAAGTCCCGGGGGAGGAGAAGAGAGGCTTCCCG-CCTAGAGCATTTGCAAGTCAGGATTCTCTAATCCCTCTGGGAGAAGGGTATTCGGCTTGTCCGCTGT-TTTT

>LR813064 Human gammaherpesvirus 4 isolate eBL-Tumor-0041 genome assembly, chromosome: EBV

AGGACAGCCGTTGCCCTAGTGGTTTCGGACACACCGCCAACGCTCAGTGCGGTGCTACCGACCCGAGGTCAAGTCCCGGGGGAGGAGAAGAGAGGCTTCCCG-CCTAGAGCATTTGCAAGTCAGGATTCTCTAATCCCTCTGGGAGAAGGGTATTCGGCTTGTCCGCTGT-TTTT

>LR813065 Human gammaherpesvirus 4 isolate eBL-Plasma-0044 genome assembly, chromosome: EBV

AGGACAGCCGTTGCCCTAGTGGTTTCGGACACACCGCCAACGCTCAGTGCGGTGCTACCGACCCGAGGTCAAGTCCCGGGGGAGGAGAAGAGAGGCTTCCCG-CCTAGAGCATTTGCAAGTCAGGATTCTCTAATCCCTCTGGGAGAAGGGTATTCGGCTTGTCCGCTGT-TTTT

>LR813068 Human gammaherpesvirus 4 isolate eBL-Plasma-0050 genome assembly, chromosome: EBV

AGGACAGCCGTTGCCCTAGTGGTTTCGGACACACCGCCAACGCTCAGTGCGGTGCTACCGACCCGAGGTCAAGTCCCGGGGGAGGAGAAGAGAGGCTTCCCG-CCTAGAGCATTTGCAAGTCAGGATTCTCTAATCCCTCTGGGAGAAGGGTATTCGGCTTGTCCGCTGT-TTTT

>LR813069 Human gammaherpesvirus 4 isolate eBL-Plasma-0054 genome assembly, chromosome: EBV

AGGACAGCCGTTGCCCTAGTGGTTTCGGACACACCGCCAACGCTCAGTGCGGTGCTACCGACCCGAGGTCAAGTCCCGGGGGAGGAGAAGAGAGGCTTCCCG-CCTAGAGCATTTGCAAGTCAGGATTCTCTAATCCCTCTGGGAGAAGGGTATTCGGCTTGTCCGCTGT-TTTT

>LR813071 Human gammaherpesvirus 4 isolate eBL-Plasma-0052 genome assembly, chromosome: EBV

AGGACAGCCGTTGCCCTAGTGGTTTCGGACACACCGCCAACGCTCAGTGCGGTGCTACCGACCCGAGGTCAAGTCCCGGGGGAGGAGAAGAGAGGCTTCCCG-CCTAGAGCATTTGCAAGTCAGGATTCTCTAATCCCTCTGGGAGAAGGGTATTCGGCTTGTCCGCTGT-TTTT

>LR813073 Human gammaherpesvirus 4 isolate eBL-Plasma-0035 genome assembly, chromosome: EBV

AGGACAGCCGTTGCCCTAGTGGTTTCGGACACACCGCCAACGCTCAGTGCGGTGCTACCGACCCGAGGTCAAGTCCCGGGGGAGGAGAAGAGAGGCTTCCCG-CCTAGAGCATTTGCAAGTCAGGATTCTCTAATCCCTCTGGGAGAAGGGTATTCGGCTTGTCCGCTGT-TTTT

>LR813075 Human gammaherpesvirus 4 isolate eBL-Plasma-0055 genome assembly, chromosome: EBV

AGGACAGCCGTTGCCCTAGTGGTTTCGGACACACCGCCAACGCTCAGTGCGGTGCTACCGACCCGAGGTCAAGTCCCGGGGGAGGAGAAGAGAGGCTTCCCG-CCTAGAGCATTTGCAAGTCAGGATTCTCTAATCCCTCTGGGAGAAGGGTATTCGGCTTGTCCGCTGT-TTTT

>LR813076 Human gammaherpesvirus 4 isolate Jijoye genome assembly, chromosome: EBV

AGGACAGCCGTTGCCCTAGTGGTTTCGGACACACCGCCAACGCTCAGTGCGGTGCTACCGACCCGAGGTCAAGTCCCGGGGGAGGAGAAGAGAGGCTTCCCG-CCTAGAGCATTTGCAAGTCAGGATTCTCTAATCCCTCTGGGAGAAGGGTATTCGGCTTGTCCGCTGT-TTTT

>LR813077 Human gammaherpesvirus 4 isolate Daudi genome assembly, chromosome: EBV

AGGACAGCCGTTGCCCTAGTGGTTTCGGACACACCGCCAACGCTCAGTGCGGTGCTACCGACCCGAGGTCAAGTCCCGGGGGAGGAGAAGAGAGGCTTCCCG-CCTAGAGCATTTGCAAGTCAGGATTCTCTAATCCCTCTGGGAGAAGGGTATTCGGCTTGTCCGCTGT-TTTT

>LR813078 Human gammaherpesvirus 4 isolate Raji genome assembly, chromosome: EBV

AGGACAGCCGTTGCCCTAGTGGTTTCGGACACACCGCCAACGCTCAGTGCGGTGCTACCGACCCGAGGTCAAGTCCCGGGGGAGGAGAAGAGAGGCTTCCCG-CCTAGAGCATTTGCAAGTCAGGATTCTCTAATCCCTCTGGGAGAAGGGTATTCGGCTTGTCCGCTGT-TTTT

>LR813079 Human gammaherpesvirus 4 isolate eBL-Plasma-0040 genome assembly, chromosome: EBV

AGGACAGCCGTTGCCCTAGTGGTTTCGGACACACCGCCAACGCTCAGTGCGGTGCTACCGACCCGAGGTCAAGTCCCGGGGGAGGAGAAGAGAGGCTTCCCG-CCTAGAGCATTTGCAAGTCAGGATTCTCTAATCCCTCTGGGAGAAGGGTATTCGGCTTGTCCGCTGT-TTTT

>LR813080 Human gammaherpesvirus 4 isolate eBL-Plasma-0038 genome assembly, chromosome: EBV

AGGACAGCCGTTGCCCTAGTGGTTTCGGACACACCGCCAACGCTCAGTGCGGTGCTACCGACCCGAGGTCAAGTCCCGGGGGAGGAGAAGAGAGGCTTCCCG-CCTAGAGCATTTGCAAGTCAGGATTCTCTAATCCCTCTGGGAGAAGGGTATTCGGCTTGTCCGCTGT-TTTT

>LR813081 Human gammaherpesvirus 4 isolate eBL-Plasma-0039 genome assembly, chromosome: EBV

AGGACAGCCGTTGCCCTAGTGGTTTCGGACACACCGCCAACGCTCAGTGCGGTGCTACCGACCCGAGGTCAAGTCCCGGGGGAGGAGAAGAGAGGCTTCCCG-CCTAGAGCATTTGCAAGTCAGGATTCTCTAATCCCTCTGGGAGAAGGGTATTCGGCTTGTCCGCTGT-TTTT

>LR813082 Human gammaherpesvirus 4 isolate Namalwa genome assembly, chromosome: EBV

AGGACAGCCGTTGCCCTAGTGGTTTCGGACACACCGCCAACGCTCAGTGCGGTGCTACCGACCCGAGGTCAAGTCCCGGGGGAGGAGAAGAGAGGCTTCCCG-CCTAGAGCATTTGCAAGTCAGGATTCTCTAATCCCTCTGGGAGAAGGGTATTCGGCTTGTCCGCTGT-TTTT

>LR813143 Human gammaherpesvirus 4 isolate eBL-Plasma-0036 genome assembly, chromosome: EBV

AGGACAGCCGTTGCCCTAGTGGTTTCGGACACACCGCCAACGCTCAGTGCGGTGCTACCGACCCGAGGTCAAGTCCCGGGGGAGGAGAAGAGAGGCTTCCCG-CCTAGAGCATTTGCAAGTCAGGATTCTCTAATCCCTCTGGGAGAAGGGTATTCGGCTTGTCCGCTGT-TTTT

>LS992239 Human gammaherpesvirus 4 isolate Human herpesvirus 4 genome assembly, chromosome: I

AGGACAGCCGTTGCCCTAGTGGTTTCGGACACACCGCCAACGCTCAGTGCGGTGCTACCGACCCGAGGTCAAGTCCCGGGGGAGGAGAAGAGAGGCTTCCCG-CCTAGAGCATTTGCAAGTCAGGATTCTCTAATCCCTCTGGGAGAAGGGTATTCGGCTTGTCCGCTGT-TTTT

>LS992240 Human gammaherpesvirus 4 isolate Human herpesvirus 4 genome assembly, chromosome: I

AGGACAGCCGTTGCCCTAGTGGTTTCGGACACACCGCCAACGCTCAGTGCGGTGCTACCGACCCGAGGTCAAGTCCCGGGGGAGGAGAAGAGAGGCTTCCCG-CCTAGAGCATTTGCAAGTCAGGATTCTCTAATCCCTCTGGGAGAAGGGTATTCGGCTTGTCCGCTGT-TTTT

>LS992242 Human gammaherpesvirus 4 isolate Human herpesvirus 4 genome assembly, chromosome: I

AGGACAGCCGTTGCCCTAGTGGTTTCGGACACACCGCCAACGCTCAGTGCGGTGCTACCGACCCGAGGTCAAGTCCCGGGGGAGGAGAAGAGAGGCTTCCCG-CCTAGAGCATTTGCAAGTCAGGATTCTCTAATCCCTCTGGGAGAAGGGTATTCGGCTTGTCCGCTGT-TTTT

>LS992243 Human gammaherpesvirus 4 isolate Human herpesvirus 4 genome assembly, chromosome: I

AGGACAGCCGTTGCCCTAGTGGTTTCGGACACACCGCCAACGCTCAGTGCGGTGCTACCGACCCGAGGTCAAGTCCCGGGGGAGGAGAAGAGAGGCTTCCCG-CCTAGAGCATTTGCAAGTCAGGATTCTCTAATCCCTCTGGGAGAAGGGTATTCGGCTTGTCCGCTGT-TTTT

>LS992244 Human gammaherpesvirus 4 isolate Human herpesvirus 4 genome assembly, chromosome: I

AGGACAGCCGTTGCCCTAGTGGTTTCGGACACACCGCCAACGCTCAGTGCGGTGCTACCGACCCGAGGTCAAGTCCCGGGGGAGGAGAAGAGAGGCTTCCCG-CCTAGAGCATTTGCAAGTCAGGATTCTCTAATCCCTCTGGGAGAAGGGTATTCGGCTTGTCCGCTGT-TTTT

>LS992245 Human gammaherpesvirus 4 isolate Human herpesvirus 4 genome assembly, chromosome: I

AGGACAGCCGTTGCCCTAGTGGTTTCGGACACACCGCCAACGCTCAGTGCGGTGCTACCGACCCGAGGTCAAGTCCCGGGGGAGGAGAAGAGAGGCTTCCCG-CCTAGAGCATTTGCAAGTCAGGATTCTCTAATCCCTCTGGGAGAAGGGTATTCGGCTTGTCCGCTGT-TTTT

>LS992248 Human gammaherpesvirus 4 isolate Human herpesvirus 4 genome assembly, chromosome: I

AGGACAGCCGTTGCCCTAGTGGTTTCGGACACACCGCCAACGCTCAGTGCGGTGCTACCGACCCGAGGTCAAGTCCCGGGGGAGGAGAAGAGAGGCTTCCCG-CCTAGAGCATTTGCAAGTCAGGATTCTCTAATCCCTCTGGGAGAAGGGTATTCGGCTTGTCCGCTGT-TTTT

>LS992249 Human gammaherpesvirus 4 isolate Human herpesvirus 4 genome assembly, chromosome: I

AGGACAGCCGTTGCCCTAGTGGTTTCGGACACACCGCCAACGCTCAGTGCGGTGCTACCGACCCGAGGTCAAGTCCCGGGGGAGGAGAAGAGAGGCTTCCCG-CCTAGAGCATTTGCAAGTCAGGATTCTCTAATCCCTCTGGGAGAAGGGTATTCGGCTTGTCCGCTGT-TTTT

>LS992250 Human gammaherpesvirus 4 isolate Human herpesvirus 4 genome assembly, chromosome: I

AGGACAGCCGTTGCCCTAGTGGTTTCGGACACACCGCCAACGCTCAGTGCGGTGCTACCGACCCGAGGTCAAGTCCCGGGGGAGGAGAAGAGAGGCTTCCCG-CCTAGAGCATTTGCAAGTCAGGATTCTCTAATCCCTCTGGGAGAAGGGTATTCGGCTTGTCCGCTGT-TTTT

>LS992251 Human gammaherpesvirus 4 isolate Human herpesvirus 4 genome assembly, chromosome: I

AGGACAGCCGTTGCCCTAGTGGTTTCGGACACACCGCCAACGCTCAGTGCGGTGCTACCGACCCGAGGTCAAGTCCCGGGGGAGGAGAAGAGAGGCTTCCCG-CCTAGAGCATTTGCAAGTCAGGATTCTCTAATCCCTCTGGGAGAAGGGTATTCGGCTTGTCCGCTGT-TTTT

>LS992252 Human gammaherpesvirus 4 isolate Human herpesvirus 4 genome assembly, chromosome: I

AGGACAGCCGTTGCCCTAGTGGTTTCGGACACACCGCCAACGCTCAGTGCGGTGCTACCGACCCGAGGTCAAGTCCCGGGGGAGGAGAAGAGAGGCTTCCCG-CCTAGAGCATTTGCAAGTCAGGATTCTCTAATCCCTCTGGGAGAAGGGTATTCGGCTTGTCCGCTGT-TTTT

>LS992254 Human gammaherpesvirus 4 isolate Human herpesvirus 4 genome assembly, chromosome: I

AGGACAGCCGTTGCCCTAGTGGTTTCGGACACACCGCCAACGCTCAGTGCGGTGCTACCGACCCGAGGTCAAGTCCCGGGGGAGGAGAAGAGAGGCTTCCCG-CCTAGAGCATTTGCAAGTCAGGATTCTCTAATCCCTCTGGGAGAAGGGTATTCGGCTTGTCCGCTGT-TTTT

>LS992255 Human gammaherpesvirus 4 isolate Human herpesvirus 4 genome assembly, chromosome: I

AGGACAGCCGTTGCCCTAGTGGTTTCGGACACACCGCCAACGCTCAGTGCGGTGCTACCGACCCGAGGTCAAGTCCCGGGGGAGGAGAAGAGAGGCTTCCCG-CCTAGAGCATTTGCAAGTCAGGATTCTCTAATCCCTCTGGGAGAAGGGTATTCGGCTTGTCCGCTGT-TTTT

>LS992256 Human gammaherpesvirus 4 isolate Human herpesvirus 4 genome assembly, chromosome: I

AGGACAGCCGTTGCCCTAGTGGTTTCGGACACACCGCCAACGCTCAGTGCGGTGCTACCGACCCGAGGTCAAGTCCCGGGGGAGGAGAAGAGAGGCTTCCCG-CCTAGAGCATTTGCAAGTCAGGATTCTCTAATCCCTCTGGGAGAAGGGTATTCGGCTTGTCCGCTGT-TTTT

>LS992258 Human gammaherpesvirus 4 isolate Human herpesvirus 4 genome assembly, chromosome: I

AGGACAGCCGTTGCCCTAGTGGTTTCGGACACACCGCCAACGCTCAGTGCGGTGCTACCGACCCGAGGTCAAGTCCCGGGGGAGGAGAAGAGAGGCTTCCCG-CCTAGAGCATTTGCAAGTCAGGATTCTCTAATCCCTCTGGGAGAAGGGTATTCGGCTTGTCCGCTGT-TTTT

>LS992259 Human gammaherpesvirus 4 isolate Human herpesvirus 4 genome assembly, chromosome: I

AGGACAGCCGTTGCCCTAGTGGTTTCGGACACACCGCCAACGCTCAGTGCGGTGCTACCGACCCGAGGTCAAGTCCCGGGGGAGGAGAAGAGAGGCTTCCCG-CCTAGAGCATTTGCAAGTCAGGATTCTCTAATCCCTCTGGGAGAAGGGTATTCGGCTTGTCCGCTGT-TTTT

>LS992260 Human gammaherpesvirus 4 isolate Human herpesvirus 4 genome assembly, chromosome: I

AGGACAGCCGTTGCCCTAGTGGTTTCGGACACACCGCCAACGCTCAGTGCGGTGCTACCGACCCGAGGTCAAGTCCCGGGGGAGGAGAAGAGAGGCTTCCCG-CCTAGAGCATTTGCAAGTCAGGATTCTCTAATCCCTCTGGGAGAAGGGTATTCGGCTTGTCCGCTGT-TTTT

>LS992261 Human gammaherpesvirus 4 isolate Human herpesvirus 4 genome assembly, chromosome: I

AGGACAGCCGTTGCCCTAGTGGTTTCGGACACACCGCCAACGCTCAGTGCGGTGCTACCGACCCGAGGTCAAGTCCCGGGGGAGGAGAAGAGAGGCTTCCCG-CCTAGAGCATTTGCAAGTCAGGATTCTCTAATCCCTCTGGGAGAAGGGTATTCGGCTTGTCCGCTGT-TTTT

>LS992263 Human gammaherpesvirus 4 isolate Human herpesvirus 4 genome assembly, chromosome: I

AGGACAGCCGTTGCCCTAGTGGTTTCGGACACACCGCCAACGCTCAGTGCGGTGCTACCGACCCGAGGTCAAGTCCCGGGGGAGGAGAAGAGAGGCTTCCCG-CCTAGAGCATTTGCAAGTCAGGATTCTCTAATCCCTCTGGGAGAAGGGTATTCGGCTTGTCCGCTGT-TTTT

>LS992265 Human gammaherpesvirus 4 isolate Human herpesvirus 4 genome assembly, chromosome: I

AGGACAGCCGTTGCCCTAGTGGTTTCGGACACACCGCCAACGCTCAGTGCGGTGCTACCGACCCGAGGTCAAGTCCCGGGGGAGGAGAAGAGAGGCTTCCCG-CCTAGAGCATTTGCAAGTCAGGATTCTCTAATCCCTCTGGGAGAAGGGTATTCGGCTTGTCCGCTGT-TTTT

>LS992266 Human gammaherpesvirus 4 isolate Human herpesvirus 4 genome assembly, chromosome: I

AGGACAGCCGTTGCCCTAGTGGTTTCGGACACACCGCCAACGCTCAGTGCGGTGCTACCGACCCGAGGTCAAGTCCCGGGGGAGGAGAAGAGAGGCTTCCCG-CCTAGAGCATTTGCAAGTCAGGATTCTCTAATCCCTCTGGGAGAAGGGTATTCGGCTTGTCCGCTGT-TTTT

>LS992267 Human gammaherpesvirus 4 isolate Human herpesvirus 4 genome assembly, chromosome: I

AGGACAGCCGTTGCCCTAGTGGTTTCGGACACACCGCCAACGCTCAGTGCGGTGCTACCGACCCGAGGTCAAGTCCCGGGGGAGGAGAAGAGAGGCTTCCCG-CCTAGAGCATTTGCAAGTCAGGATTCTCTAATCCCTCTGGGAGAAGGGTATTCGGCTTGTCCGCTGT-TTTT

>LS992268 Human gammaherpesvirus 4 isolate Human herpesvirus 4 genome assembly, chromosome: I

AGGACAGCCGTTGCCCTAGTGGTTTCGGACACACCGCCAACGCTCAGTGCGGTGCTACCGACCCGAGGTCAAGTCCCGGGGGAGGAGAAGAGAGGCTTCCCG-CCTAGAGCATTTGCAAGTCAGGATTCTCTAATCCCTCTGGGAGAAGGGTATTCGGCTTGTCCGCTGT-TTTT

>LS992276 Human gammaherpesvirus 4 isolate Human herpesvirus 4 genome assembly, chromosome: I

AGGACAGCCGTTGCCCTAGTGGTTTCGGACACACCGCCAACGCTCAGTGCGGTGCTACCGACCCGAGGTCAAGTCCCGGGGGAGGAGAAGAGAGGCTTCCCG-CCTAGAGCATTTGCAAGTCAGGATTCTCTAATCCCTCTGGGAGAAGGGTATTCGGCTTGTCCGCTGT-TTTT

>LS992277 Human gammaherpesvirus 4 isolate Human herpesvirus 4 genome assembly, chromosome: I

AGGACAGCCGTTGCCCTAGTGGTTTCGGACACACCGCCAACGCTCAGTGCGGTGCTACCGACCCGAGGTCAAGTCCCGGGGGAGGAGAAGAGAGGCTTCCCG-CCTAGAGCATTTGCAAGTCAGGATTCTCTAATCCCTCTGGGAGAAGGGTATTCGGCTTGTCCGCTGT-TTTT

>MG021305 Human gammaherpesvirus 4 isolate YCCEL1-GC1

AGGACAGCCGTTGCCCTAGTGGTTTCGGACACACCGCCAACGCTCAGTGCGGTGCTACCGACCCGAGGTCAAGTCCCGGGGGAGGAGAAGAGAGGCTTCCCG-CCTAGAGCATTTGCAAGTCAGGATTCTCTAATCCCTCTGGGAGAAGGGTATTCGGCTTGTCCGCTGT-TTTT

>MG021307 Human gammaherpesvirus 4 isolate Akata-GC1

AGGACAGCCGTTGCCCTAGTGGTTTCGGACACACCGCCAACGCTCAGTGCGGTGCTACCGACCCGAGGTCAAGTCCCGGGGGAGGAGAAGAGAGGCTTCCCG-CCTAGAGCATTTGCAAGTCAGGATTCTCTAATCCCTCTGGGAGAAGGGTATTCGGCTTGTCCGCTGT-TTTT

>MG021308 Human gammaherpesvirus 4 isolate Mutu-GC1

AGGACAGCCGTTGCCCTAGTGGTTTCGGACACACCGCCAACGCTCAGTGCGGTGCTACCGACCCGAGGTCAAGTCCCGGGGGAGGAGAAGAGAGGCTTCCCG-CCTAGAGCATTTGCAAGTCAGGATTCTCTAATCCCTCTGGGAGAAGGGTATTCGGCTTGTCCGCTGT-TTTT

>MG021309 Human gammaherpesvirus 4 isolate Mutu-GC2

AGGACAGCCGTTGCCCTAGTGGTTTCGGACACACCGCCAACGCTCAGTGCGGTGCTACCGACCCGAGGTCAAGTCCCGGGGGAGGAGAAGAGAGGCTTCCCG-CCTAGAGCATTTGCAAGTCAGGATTCTCTAATCCCTCTGGGAGAAGGGTATTCGGCTTGTCCGCTGT-TTTT

>MG021310 Human gammaherpesvirus 4 isolate Mutu-GC3

AGGACAGCCGTTGCCCTAGTGGTTTCGGACACACCGCCAACGCTCAGTGCGGTGCTACCGACCCGAGGTCAAGTCCCGGGGGAGGAGAAGAGAGGCTTCCCG-CCTAGAGCATTTGCAAGTCAGGATTCTCTAATCCCTCTGGGAGAAGGGTATTCGGCTTGTCCGCTGT-TTTT

>MG021311 Human gammaherpesvirus 4 isolate Mutu-GC4

AGGACAGCCGTTGCCCTAGTGGTTTCGGACACACCGCCAACGCTCAGTGCGGTGCTACCGACCCGAGGTCAAGTCCCGGGGGAGGAGAAGAGAGGCTTCCCG-CCTAGAGCATTTGCAAGTCAGGATTCTCTAATCCCTCTGGGAGAAGGGTATTCGGCTTGTCCGCTGT-TTTT

>MG021312 Human gammaherpesvirus 4 isolate AG876-GC1

AGGACAGCCGTTGCCCTAGTGGTTTCGGACACACCGCCAACGCTCAGTGCGGTGCTACCGACCCGAGGTCAAGTCCCGGGGGAGGAGAAGAGAGGCTTCCCG-CCTAGAGCATTTGCAAGTCAGGATTCTCTAATCCCTCTGGGAGAAGGGTATTCGGCTTGTCCGCTGT-TTTT

>MG021313 Human gammaherpesvirus 4 isolate EBVaGC8-1

AGGACAGCCGTTGCCCTAGTGGTTTCGGACACACCGCCAACGCTCAGTGCGGTGCTACCGACCCGAGGTCAAGTCCCGGGGGAGGAGAAGAGAGGCTTCCCG-CCTAGAGCATTTGCAAGTCAGGATTCTCTAATCCCTCTGGGAGAAGGGTATTCGGCTTGTCCGCTGT-TTTT

>MG021314 Human gammaherpesvirus 4 isolate EBVaGC8-2

AGGACAGCCGTTGCCCTAGTGGTTTCGGACACACCGCCAACGCTCAGTGCGGTGCTACCGACCCGAGGTCAAGTCCCGGGGGAGGAGAAGAGAGGCTTCCCG-CCTAGAGCATTTGCAAGTCAGGATTCTCTAATCCCTCTGGGAGAAGGGTATTCGGCTTGTCCGCTGT-TTTT

>MG021315 Human gammaherpesvirus 4 isolate EBVaGC8-3

AGGACAGCCGTTGCCCTAGTGGTTTCGGACACACCGCCAACGCTCAGTGCGGTGCTACCGACCCGAGGTCAAGTCCCGGGGGAGGAGAAGAGAGGCTTCCCG-CCTAGAGCATTTGCAAGTCAGGATTCTCTAATCCCTCTGGGAGAAGGGTATTCGGCTTGTCCGCTGT-TTTT

>MG021316 Human gammaherpesvirus 4 isolate HKNPC6-GC1

AGGACAGCCGTTGCCCTAGTGGTTTCGGACACACCGCCAACGCTCAGTGCGGTGCTACCGACCCGAGGTCAAGTCCCGGGGGAGGAGAAGAGAGGCTTCCCG-CCTAGAGCATTTGCAAGTCAGGATTCTCTAATCCCTCTGGGAGAAGGGTATTCGGCTTGTCCGCTGT-TTTT

>MG021317 Human gammaherpesvirus 4 isolate EBVaGC5-1

AGGACAGCCGTTGCCCTAGTGGTTTCGGACACACCGCCAACGCTCAGTGCGGTGCTACCGACCCGAGGTCAAGTCCCGGGGGAGGAGAAGAGAGGCTTCCCG-CCTAGAGCATTTGCAAGTCAGGATTCTCTAATCCCTCTGGGAGAAGGGTATTCGGCTTGTCCGCTGT-TTTT

>MG298825 Human gammaherpesvirus 4 isolate AH_Saliva_8489

AGGACAGCCGTTGCCCTAGTGGTTTCGGACACACCGCCAACGCTCAGTGCGGTGCTACCGACCCGAGGTCAAGTCCCGGGGGAGGAGAAGAGAGGCTTCCCG-CCTAGAGCATTTGCAAGTCAGGATTCTCTAATCCCTCTGGGAGAAGGGTATTCGGCTTGTCCGCTGT-TTTT

>MG298826 Human gammaherpesvirus 4 isolate AH_Saliva_9077

AGGACAGCCGTTGCCCTAGTGGTTTCGGACACACCGCCAACGCTCAGTGCGGTGCTACCGACCCGAGGTCAAGTCCCGGGGGAGGAGAAGAGAGGCTTCCCG-CCTAGAGCATTTGCAAGTCAGGATTCTCTAATCCCTCTGGGAGAAGGGTATTCGGCTTGTCCGCTGT-TTTT

>MG298827 Human gammaherpesvirus 4 isolate AH_Saliva_9316

AGGACAGCCGTTGCCCTAGTGGTTTCGGACACACCGCCAACGCTCAGTGCGGTGCTACCGACCCGAGGTCAAGTCCCGGGGGAGGAGAAGAGAGGCTTCCCG-CCTAGAGCATTTGCAAGTCAGGATTCTCTAATCCCTCTGGGAGAAGGGTATTCGGCTTGTCCGCTGT-TTTT

>MG298830 Human gammaherpesvirus 4 isolate GK_Akuba

AGGACAGCCGTTGCCCTAGTGGTTTCGGACACACCGCCAACGCTCAGTGCGGTGCTACCGACCCGAGGTCAAGTCCCGGGGGAGGAGAAGAGAGGCTTCCCG-CCTAGAGCATTTGCAAGTCAGGATTCTCTAATCCCTCTGGGAGAAGGGTATTCGGCTTGTCCGCTGT-TTTT

>MG298831 Human gammaherpesvirus 4 isolate GK_BL16

AGGACAGCCGTTGCCCTAGTGGTTTCGGACACACCGCCAACGCTCAGTGCGGTGCTACCGACCCGAGGTCAAGTCCCGGGGGAGGAGAAGAGAGGCTTCCCG-CCTAGAGCATTTGCAAGTCAGGATTCTCTAATCCCTCTGGGAGAAGGGTATTCGGCTTGTCCGCTGT-TTTT

>MG298837 Human gammaherpesvirus 4 isolate GK_BL67

AGGACAGCCGTTGCCCTAGTGGTTTCGGACACACCGCCAACGCTCAGTGCGGTGCTACCGACCCGAGGTCAAGTCCCGGGGGAGGAGAAGAGAGGCTTCCCG-CCTAGAGCATTTGCAAGTCAGGATTCTCTAATCCCTCTGGGAGAAGGGTATTCGGCTTGTCCGCTGT-TTTT

>MG298838 Human gammaherpesvirus 4 isolate GK_BL72

AGGACAGCCGTTGCCCTAGTGGTTTCGGACACACCGCCAACGCTCAGTGCGGTGCTACCGACCCGAGGTCAAGTCCCGGGGGAGGAGAAGAGAGGCTTCCCG-CCTAGAGCATTTGCAAGTCAGGATTCTCTAATCCCTCTGGGAGAAGGGTATTCGGCTTGTCCGCTGT-TTTT

>MG298839 Human gammaherpesvirus 4 isolate GK_Farage

AGGACAGCCGTTGCCCTAGTGGTTTCGGACACACCGCCAACGCTCAGTGCGGTGCTACCGACCCGAGGTCAAGTCCCGGGGGAGGAGAAGAGAGGCTTCCCG-CCTAGAGCATTTGCAAGTCAGGATTCTCTAATCCCTCTGGGAGAAGGGTATTCGGCTTGTCCGCTGT-TTTT

>MG298840 Human gammaherpesvirus 4 isolate GK_LY47

AGGACAGCCGTTGCCCTAGTGGTTTCGGACACACCGCCAACGCTCAGTGCGGTGCTACCGACCCGAGGTCAAGTCCCGGGGGAGGAGAAGAGAGGCTTCCCG-CCTAGAGCATTTGCAAGTCAGGATTCTCTAATCCCTCTGGGAGAAGGGTATTCGGCTTGTCCGCTGT-TTTT

>MG298842 Human gammaherpesvirus 4 isolate GK_PUT

AGGACAGCCGTTGCCCTAGTGGTTTCGGACACACCGCCAACGCTCAGTGCGGTGCTACCGACCCGAGGTCAAGTCCCGGGGGAGGAGAAGAGAGGCTTCCCG-CCTAGAGCATTTGCAAGTCAGGATTCTCTAATCCCTCTGGGAGAAGGGTATTCGGCTTGTCCGCTGT-TTTT

>MG298843 Human gammaherpesvirus 4 isolate GK_RUDU

AGGACAGCCGTTGCCCTAGTGGTTTCGGACACACCGCCAACGCTCAGTGCGGTGCTACCGACCCGAGGTCAAGTCCCGGGGGAGGAGAAGAGAGGCTTCCCG-CCTAGAGCATTTGCAAGTCAGGATTCTCTAATCCCTCTGGGAGAAGGGTATTCGGCTTGTCCGCTGT-TTTT

>MG298845 Human gammaherpesvirus 4 isolate IMS_Saliva_12

AGGACAGCCGTTGCCCTAGTGGTTTCGGACACACCGCCAACGCTCAGTGCGGTGCTACCGACCCGAGGTCAAGTCCCGGGGGAGGAGAAGAGAGGCTTCCCG-CCTAGAGCATTTGCAAGTCAGGATTCTCTAATCCCTCTGGGAGAAGGGTATTCGGCTTGTCCGCTGT-TTTT

>MG298846 Human gammaherpesvirus 4 isolate IMS_Saliva_120

AGGACAGCCGTTGCCCTAGTGGTTTCGGACACACCGCCAACGCTCAGTGCGGTGCTACCGACCCGAGGTCAAGTCCCGGGGGAGGAGAAGAGAGGCTTCCCG-CCTAGAGCATTTGCAAGTCAGGATTCTCTAATCCCTCTGGGAGAAGGGTATTCGGCTTGTCCGCTGT-TTTT

>MG298848 Human gammaherpesvirus 4 isolate IMS_Saliva_170

AGGACAGCCGTTGCCCTAGTGGTTTCGGACACACCGCCAACGCTCAGTGCGGTGCTACCGACCCGAGGTCAAGTCCCGGGGGAGGAGAAGAGAGGCTTCCCG-CCTAGAGCATTTGCAAGTCAGGATTCTCTAATCCCTCTGGGAGAAGGGTATTCGGCTTGTCCGCTGT-TTTT

>MG298849 Human gammaherpesvirus 4 isolate IMS_Saliva_177

AGGACAGCCGTTGCCCTAGTGGTTTCGGACACACCGCCAACGCTCAGTGCGGTGCTACCGACCCGAGGTCAAGTCCCGGGGGAGGAGAAGAGAGGCTTCCCG-CCTAGAGCATTTGCAAGTCAGGATTCTCTAATCCCTCTGGGAGAAGGGTATTCGGCTTGTCCGCTGT-TTTT

>MG298851 Human gammaherpesvirus 4 isolate IMS_Saliva_204

AGGACAGCCGTTGCCCTAGTGGTTTCGGACACACCGCCAACGCTCAGTGCGGTGCTACCGACCCGAGGTCAAGTCCCGGGGGAGGAGAAGAGAGGCTTCCCG-CCTAGAGCATTTGCAAGTCAGGATTCTCTAATCCCTCTGGGAGAAGGGTATTCGGCTTGTCCGCTGT-TTTT

>MG298852 Human gammaherpesvirus 4 isolate IMS_Saliva_220

AGGACAGCCGTTGCCCTAGTGGTTTCGGACACACCGCCAACGCTCAGTGCGGTGCTACCGACCCGAGGTCAAGTCCCGGGGGAGGAGAAGAGAGGCTTCCCG-CCTAGAGCATTTGCAAGTCAGGATTCTCTAATCCCTCTGGGAGAAGGGTATTCGGCTTGTCCGCTGT-TTTT

>MG298853 Human gammaherpesvirus 4 isolate IMS_Saliva_231

AGGACAGCCGTTGCCCTAGTGGTTTCGGACACACCGCCAACGCTCAGTGCGGTGCTACCGACCCGAGGTCAAGTCCCGGGGGAGGAGAAGAGAGGCTTCCCG-CCTAGAGCATTTGCAAGTCAGGATTCTCTAATCCCTCTGGGAGAAGGGTATTCGGCTTGTCCGCTGT-TTTT

>MG298854 Human gammaherpesvirus 4 isolate IMS_Saliva_243

AGGACAGCCGTTGCCCTAGTGGTTTCGGACACACCGCCAACGCTCAGTGCGGTGCTACCGACCCGAGGTCAAGTCCCGGGGGAGGAGAAGAGAGGCTTCCCG-CCTAGAGCATTTGCAAGTCAGGATTCTCTAATCCCTCTGGGAGAAGGGTATTCGGCTTGTCCGCTGT-TTTT

>MG298855 Human gammaherpesvirus 4 isolate IMS_Saliva_248

AGGACAGCCGTTGCCCTAGTGGTTTCGGACACACCGCCAACGCTCAGTGCGGTGCTACCGACCCGAGGTCAAGTCCCGGGGGAGGAGAAGAGAGGCTTCCCG-CCTAGAGCATTTGCAAGTCAGGATTCTCTAATCCCTCTGGGAGAAGGGTATTCGGCTTGTCCGCTGT-TTTT

>MG298857 Human gammaherpesvirus 4 isolate IMS_Saliva_31

AGGACAGCCGTTGCCCTAGTGGTTTCGGACACACCGCCAACGCTCAGTGCGGTGCTACCGACCCGAGGTCAAGTCCCGGGGGAGGAGAAGAGAGGCTTCCCG-CCTAGAGCATTTGCAAGTCAGGATTCTCTAATCCCTCTGGGAGAAGGGTATTCGGCTTGTCCGCTGT-TTTT

>MG298858 Human gammaherpesvirus 4 isolate IMS_Saliva_49

AGGACAGCCGTTGCCCTAGTGGTTTCGGACACACCGCCAACGCTCAGTGCGGTGCTACCGACCCGAGGTCAAGTCCCGGGGGAGGAGAAGAGAGGCTTCCCG-CCTAGAGCATTTGCAAGTCAGGATTCTCTAATCCCTCTGGGAGAAGGGTATTCGGCTTGTCCGCTGT-TTTT

>MG298859 Human gammaherpesvirus 4 isolate IMS_Saliva_52

AGGACAGCCGTTGCCCTAGTGGTTTCGGACACACCGCCAACGCTCAGTGCGGTGCTACCGACCCGAGGTCAAGTCCCGGGGGAGGAGAAGAGAGGCTTCCCG-CCTAGAGCATTTGCAAGTCAGGATTCTCTAATCCCTCTGGGAGAAGGGTATTCGGCTTGTCCGCTGT-TTTT

>MG298860 Human gammaherpesvirus 4 isolate IMS_Saliva_6

AGGACAGCCGTTGCCCTAGTGGTTTCGGACACACCGCCAACGCTCAGTGCGGTGCTACCGACCCGAGGTCAAGTCCCGGGGGAGGAGAAGAGAGGCTTCCCG-CCTAGAGCATTTGCAAGTCAGGATTCTCTAATCCCTCTGGGAGAAGGGTATTCGGCTTGTCCGCTGT-TTTT

>MG298861 Human gammaherpesvirus 4 isolate IMS_Saliva_70

AGGACAGCCGTTGCCCTAGTGGTTTCGGACACACCGCCAACGCTCAGTGCGGTGCTACCGACCCGAGGTCAAGTCCCGGGGGAGGAGAAGAGAGGCTTCCCG-CCTAGAGCATTTGCAAGTCAGGATTCTCTAATCCCTCTGGGAGAAGGGTATTCGGCTTGTCCGCTGT-TTTT

>MG298862 Human gammaherpesvirus 4 isolate IMS_Saliva_71

AGGACAGCCGTTGCCCTAGTGGTTTCGGACACACCGCCAACGCTCAGTGCGGTGCTACCGACCCGAGGTCAAGTCCCGGGGGAGGAGAAGAGAGGCTTCCCG-CCTAGAGCATTTGCAAGTCAGGATTCTCTAATCCCTCTGGGAGAAGGGTATTCGGCTTGTCCGCTGT-TTTT

>MG298863 Human gammaherpesvirus 4 isolate IMS_Saliva_9

AGGACAGCCGTTGCCCTAGTGGTTTCGGACACACCGCCAACGCTCAGTGCGGTGCTACCGACCCGAGGTCAAGTCCCGGGGGAGGAGAAGAGAGGCTTCCCG-CCTAGAGCATTTGCAAGTCAGGATTCTCTAATCCCTCTGGGAGAAGGGTATTCGGCTTGTCCGCTGT-TTTT

>MG298864 Human gammaherpesvirus 4 isolate JC_002

AGGACAGCCGTTGCCCTAGTGGTTTCGGACACACCGCCAACGCTCAGTGCGGTGCTACCGACCCGAGGTCAAGTCCCGGGGGAGGAGAAGAGAGGCTTCCCG-CCTAGAGCATTTGCAAGTCAGGATTCTCTAATCCCTCTGGGAGAAGGGTATTCGGCTTGTCCGCTGT-TTTT

>MG298865 Human gammaherpesvirus 4 isolate JC_023

AGGACAGCCGTTGCCCTAGTGGTTTCGGACACACCGCCAACGCTCAGTGCGGTGCTACCGACCCGAGGTCAAGTCCCGGGGGAGGAGAAGAGAGGCTTCCCG-CCTAGAGCATTTGCAAGTCAGGATTCTCTAATCCCTCTGGGAGAAGGGTATTCGGCTTGTCCGCTGT-TTTT

>MG298866 Human gammaherpesvirus 4 isolate JC_030_18

AGGACAGCCGTTGCCCTAGTGGTTTCGGACACACCGCCAACGCTCAGTGCGGTGCTACCGACCCGAGGTCAAGTCCCGGGGGAGGAGAAGAGAGGCTTCCCG-CCTAGAGCATTTGCAAGTCAGGATTCTCTAATCCCTCTGGGAGAAGGGTATTCGGCTTGTCCGCTGT-TTTT

>MG298867 Human gammaherpesvirus 4 isolate JC_030_29

AGGACAGCCGTTGCCCTAGTGGTTTCGGACACACCGCCAACGCTCAGTGCGGTGCTACCGACCCGAGGTCAAGTCCCGGGGGAGGAGAAGAGAGGCTTCCCG-CCTAGAGCATTTGCAAGTCAGGATTCTCTAATCCCTCTGGGAGAAGGGTATTCGGCTTGTCCGCTGT-TTTT

>MG298868 Human gammaherpesvirus 4 isolate JC_037

AGGACAGCCGTTGCCCTAGTGGTTTCGGACACACCGCCAACGCTCAGTGCGGTGCTACCGACCCGAGGTCAAGTCCCGGGGGAGGAGAAGAGAGGCTTCCCG-CCTAGAGCATTTGCAAGTCAGGATTCTCTAATCCCTCTGGGAGAAGGGTATTCGGCTTGTCCGCTGT-TTTT

>MG298869 Human gammaherpesvirus 4 isolate JC_040

AGGACAGCCGTTGCCCTAGTGGTTTCGGACACACCGCCAACGCTCAGTGCGGTGCTACCGACCCGAGGTCAAGTCCCGGGGGAGGAGAAGAGAGGCTTCCCG-CCTAGAGCATTTGCAAGTCAGGATTCTCTAATCCCTCTGGGAGAAGGGTATTCGGCTTGTCCGCTGT-TTTT

>MG298870 Human gammaherpesvirus 4 isolate JC_V005

AGGACAGCCGTTGCCCTAGTGGTTTCGGACACACCGCCAACGCTCAGTGCGGTGCTACCGACCCGAGGTCAAGTCCCGGGGGAGGAGAAGAGAGGCTTCCCG-CCTAGAGCATTTGCAAGTCAGGATTCTCTAATCCCTCTGGGAGAAGGGTATTCGGCTTGTCCGCTGT-TTTT

>MG298871 Human gammaherpesvirus 4 isolate JC_V006

AGGACAGCCGTTGCCCTAGTGGTTTCGGACACACCGCCAACGCTCAGTGCGGTGCTACCGACCCGAGGTCAAGTCCCGGGGGAGGAGAAGAGAGGCTTCCCG-CCTAGAGCATTTGCAAGTCAGGATTCTCTAATCCCTCTGGGAGAAGGGTATTCGGCTTGTCCGCTGT-TTTT

>MG298872 Human gammaherpesvirus 4 isolate JM_LCL_IK

AGGACAGCCGTTGCCCTAGTGGTTTCGGACACACCGCCAACGCTCAGTGCGGTGCTACCGACCCGAGGTCAAGTCCCGGGGGAGGAGAAGAGAGGCTTCCCG-CCTAGAGCATTTGCAAGTCAGGATTCTCTAATCCCTCTGGGAGAAGGGTATTCGGCTTGTCCGCTGT-TTTT

>MG298873 Human gammaherpesvirus 4 isolate JM_LCL_IN

AGGACAGCCGTTGCCCTAGTGGTTTCGGACACACCGCCAACGCTCAGTGCGGTGCTACCGACCCGAGGTCAAGTCCCGGGGGAGGAGAAGAGAGGCTTCCCG-CCTAGAGCATTTGCAAGTCAGGATTCTCTAATCCCTCTGGGAGAAGGGTATTCGGCTTGTCCGCTGT-TTTT

>MG298874 Human gammaherpesvirus 4 isolate JM_LCL_MU

AGGACAGCCGTTGCCCTAGTGGTTTCGGACACACCGCCAACGCTCAGTGCGGTGCTACCGACCCGAGGTCAAGTCCCGGGGGAGGAGAAGAGAGGCTTCCCG-CCTAGAGCATTTGCAAGTCAGGATTCTCTAATCCCTCTGGGAGAAGGGTATTCGGCTTGTCCGCTGT-TTTT

>MG298875 Human gammaherpesvirus 4 isolate JM_LCL_SU

AGGACAGCCGTTGCCCTAGTGGTTTCGGACACACCGCCAACGCTCAGTGCGGTGCTACCGACCCGAGGTCAAGTCCCGGGGGAGGAGAAGAGAGGCTTCCCG-CCTAGAGCATTTGCAAGTCAGGATTCTCTAATCCCTCTGGGAGAAGGGTATTCGGCTTGTCCGCTGT-TTTT

>MG298876 Human gammaherpesvirus 4 isolate JM_NKTLY_218.1

AGGACAGCCGTTGCCCTAGTGGTTTCGGACACACCGCCAACGCTCAGTGCGGTGCTACCGACCCGAGGTCAAGTCCCGGGGGAGGAGAAGAGAGGCTTCCCG-CCTAGAGCATTTGCAAGTCAGGATTCTCTAATCCCTCTGGGAGAAGGGTATTCGGCTTGTCCGCTGT-TTTT

>MG298877 Human gammaherpesvirus 4 isolate JM_NKTLY_96.1

AGGACAGCCGTTGCCCTAGTGGTTTCGGACACACCGCCAACGCTCAGTGCGGTGCTACCGACCCGAGGTCAAGTCCCGGGGGAGGAGAAGAGAGGCTTCCCG-CCTAGAGCATTTGCAAGTCAGGATTCTCTAATCCCTCTGGGAGAAGGGTATTCGGCTTGTCCGCTGT-TTTT

>MG298878 Human gammaherpesvirus 4 isolate JM_NKTLY_98.1

AGGACAGCCGTTGCCCTAGTGGTTTCGGACACACCGCCAACGCTCAGTGCGGTGCTACCGACCCGAGGTCAAGTCCCGGGGGAGGAGAAGAGAGGCTTCCCG-CCTAGAGCATTTGCAAGTCAGGATTCTCTAATCCCTCTGGGAGAAGGGTATTCGGCTTGTCCGCTGT-TTTT

>MG298879 Human gammaherpesvirus 4 isolate JM_NPC_biop_162

AGGACAGCCGTTGCCCTAGTGGTTTCGGACACACCGCCAACGCTCAGTGCGGTGCTACCGACCCGAGGTCAAGTCCCGGGGGAGGAGAAGAGAGGCTTCCCG-CCTAGAGCATTTGCAAGTCAGGATTCTCTAATCCCTCTGGGAGAAGGGTATTCGGCTTGTCCGCTGT-TTTT

>MG298880 Human gammaherpesvirus 4 isolate JM_NPC_biop_238

AGGACAGCCGTTGCCCTAGTGGTTTCGGACACACCGCCAACGCTCAGTGCGGTGCTACCGACCCGAGGTCAAGTCCCGGGGGAGGAGAAGAGAGGCTTCCCG-CCTAGAGCATTTGCAAGTCAGGATTCTCTAATCCCTCTGGGAGAAGGGTATTCGGCTTGTCCGCTGT-TTTT

>MG298881 Human gammaherpesvirus 4 isolate JM_NPC_biop_27

AGGACAGCCGTTGCCCTAGTGGTTTCGGACACACCGCCAACGCTCAGTGCGGTGCTACCGACCCGAGGTCAAGTCCCGGGGGAGGAGAAGAGAGGCTTCCCG-CCTAGAGCATTTGCAAGTCAGGATTCTCTAATCCCTCTGGGAGAAGGGTATTCGGCTTGTCCGCTGT-TTTT

>MG298882 Human gammaherpesvirus 4 isolate JM_NPC_biop_51

AGGACAGCCGTTGCCCTAGTGGTTTCGGACACACCGCCAACGCTCAGTGCGGTGCTACCGACCCGAGGTCAAGTCCCGGGGGAGGAGAAGAGAGGCTTCCCG-CCTAGAGCATTTGCAAGTCAGGATTCTCTAATCCCTCTGGGAGAAGGGTATTCGGCTTGTCCGCTGT-TTTT

>MG298884 Human gammaherpesvirus 4 isolate JM_NPC_bru_238

AGGACAGCCGTTGCCCTAGTGGTTTCGGACACACCGCCAACGCTCAGTGCGGTGCTACCGACCCGAGGTCAAGTCCCGGGGGAGGAGAAGAGAGGCTTCCCG-CCTAGAGCATTTGCAAGTCAGGATTCTCTAATCCCTCTGGGAGAAGGGTATTCGGCTTGTCCGCTGT-TTTT

>MG298885 Human gammaherpesvirus 4 isolate JM_NPC_bru_377

AGGACAGCCGTTGCCCTAGTGGTTTCGGACACACCGCCAACGCTCAGTGCGGTGCTACCGACCCGAGGTCAAGTCCCGGGGGAGGAGAAGAGAGGCTTCCCG-CCTAGAGCATTTGCAAGTCAGGATTCTCTAATCCCTCTGGGAGAAGGGTATTCGGCTTGTCCGCTGT-TTTT

>MG298886 Human gammaherpesvirus 4 isolate JM_NPC_bru_38

AGGACAGCCGTTGCCCTAGTGGTTTCGGACACACCGCCAACGCTCAGTGCGGTGCTACCGACCCGAGGTCAAGTCCCGGGGGAGGAGAAGAGAGGCTTCCCG-CCTAGAGCATTTGCAAGTCAGGATTCTCTAATCCCTCTGGGAGAAGGGTATTCGGCTTGTCCGCTGT-TTTT

>MG298887 Human gammaherpesvirus 4 isolate JM_NPC_bru_51

AGGACAGCCGTTGCCCTAGTGGTTTCGGACACACCGCCAACGCTCAGTGCGGTGCTACCGACCCGAGGTCAAGTCCCGGGGGAGGAGAAGAGAGGCTTCCCG-CCTAGAGCATTTGCAAGTCAGGATTCTCTAATCCCTCTGGGAGAAGGGTATTCGGCTTGTCCGCTGT-TTTT

>MG298889 Human gammaherpesvirus 4 isolate JM_NPC_bru_L2

AGGACAGCCGTTGCCCTAGTGGTTTCGGACACACCGCCAACGCTCAGTGCGGTGCTACCGACCCGAGGTCAAGTCCCGGGGGAGGAGAAGAGAGGCTTCCCG-CCTAGAGCATTTGCAAGTCAGGATTCTCTAATCCCTCTGGGAGAAGGGTATTCGGCTTGTCCGCTGT-TTTT

>MG298890 Human gammaherpesvirus 4 isolate JM_NPC_bru_L29

AGGACAGCCGTTGCCCTAGTGGTTTCGGACACACCGCCAACGCTCAGTGCGGTGCTACCGACCCGAGGTCAAGTCCCGGGGGAGGAGAAGAGAGGCTTCCCG-CCTAGAGCATTTGCAAGTCAGGATTCTCTAATCCCTCTGGGAGAAGGGTATTCGGCTTGTCCGCTGT-TTTT

>MG298891 Human gammaherpesvirus 4 isolate JM_NPC_bru_L3

AGGACAGCCGTTGCCCTAGTGGTTTCGGACACACCGCCAACGCTCAGTGCGGTGCTACCGACCCGAGGTCAAGTCCCGGGGGAGGAGAAGAGAGGCTTCCCG-CCTAGAGCATTTGCAAGTCAGGATTCTCTAATCCCTCTGGGAGAAGGGTATTCGGCTTGTCCGCTGT-TTTT

>MG298892 Human gammaherpesvirus 4 isolate JM_NPC_bru_L37

AGGACAGCCGTTGCCCTAGTGGTTTCGGACACACCGCCAACGCTCAGTGCGGTGCTACCGACCCGAGGTCAAGTCCCGGGGGAGGAGAAGAGAGGCTTCCCG-CCTAGAGCATTTGCAAGTCAGGATTCTCTAATCCCTCTGGGAGAAGGGTATTCGGCTTGTCCGCTGT-TTTT

>MG298893 Human gammaherpesvirus 4 isolate JM_NPC_bru_L4

AGGACAGCCGTTGCCCTAGTGGTTTCGGACACACCGCCAACGCTCAGTGCGGTGCTACCGACCCGAGGTCAAGTCCCGGGGGAGGAGAAGAGAGGCTTCCCG-CCTAGAGCATTTGCAAGTCAGGATTCTCTAATCCCTCTGGGAGAAGGGTATTCGGCTTGTCCGCTGT-TTTT

>MG298894 Human gammaherpesvirus 4 isolate JM_NPC_bru_L41

AGGACAGCCGTTGCCCTAGTGGTTTCGGACACACCGCCAACGCTCAGTGCGGTGCTACCGACCCGAGGTCAAGTCCCGGGGGAGGAGAAGAGAGGCTTCCCG-CCTAGAGCATTTGCAAGTCAGGATTCTCTAATCCCTCTGGGAGAAGGGTATTCGGCTTGTCCGCTGT-TTTT

>MG298895 Human gammaherpesvirus 4 isolate JM_NPC_bru_L42

AGGACAGCCGTTGCCCTAGTGGTTTCGGACACACCGCCAACGCTCAGTGCGGTGCTACCGACCCGAGGTCAAGTCCCGGGGGAGGAGAAGAGAGGCTTCCCG-CCTAGAGCATTTGCAAGTCAGGATTCTCTAATCCCTCTGGGAGAAGGGTATTCGGCTTGTCCGCTGT-TTTT

>MG298898 Human gammaherpesvirus 4 isolate JM_NPC_bru_L7

AGGACAGCCGTTGCCCTAGTGGTTTCGGACACACCGCCAACGCTCAGTGCGGTGCTACCGACCCGAGGTCAAGTCCCGGGGGAGGAGAAGAGAGGCTTCCCG-CCTAGAGCATTTGCAAGTCAGGATTCTCTAATCCCTCTGGGAGAAGGGTATTCGGCTTGTCCGCTGT-TTTT

>MG298899 Human gammaherpesvirus 4 isolate JM_Saliva_18

AGGACAGCCGTTGCCCTAGTGGTTTCGGACACACCGCCAACGCTCAGTGCGGTGCTACCGACCCGAGGTCAAGTCCCGGGGGAGGAGAAGAGAGGCTTCCCG-CCTAGAGCATTTGCAAGTCAGGATTCTCTAATCCCTCTGGGAGAAGGGTATTCGGCTTGTCCGCTGT-TTTT

>MG298900 Human gammaherpesvirus 4 isolate JM_Saliva_20

AGGACAGCCGTTGCCCTAGTGGTTTCGGACACACCGCCAACGCTCAGTGCGGTGCTACCGACCCGAGGTCAAGTCCCGGGGGAGGAGAAGAGAGGCTTCCCG-CCTAGAGCATTTGCAAGTCAGGATTCTCTAATCCCTCTGGGAGAAGGGTATTCGGCTTGTCCGCTGT-TTTT

>MG298901 Human gammaherpesvirus 4 isolate JM_Saliva_33

AGGACAGCCGTTGCCCTAGTGGTTTCGGACACACCGCCAACGCTCAGTGCGGTGCTACCGACCCGAGGTCAAGTCCCGGGGGAGGAGAAGAGAGGCTTCCCG-CCTAGAGCATTTGCAAGTCAGGATTCTCTAATCCCTCTGGGAGAAGGGTATTCGGCTTGTCCGCTGT-TTTT

>MG298902 Human gammaherpesvirus 4 isolate JM_Saliva_5

AGGACAGCCGTTGCCCTAGTGGTTTCGGACACACCGCCAACGCTCAGTGCGGTGCTACCGACCCGAGGTCAAGTCCCGGGGGAGGAGAAGAGAGGCTTCCCG-CCTAGAGCATTTGCAAGTCAGGATTCTCTAATCCCTCTGGGAGAAGGGTATTCGGCTTGTCCGCTGT-TTTT

>MG298903 Human gammaherpesvirus 4 isolate JWBL121B

AGGACAGCCGTTGCCCTAGTGGTTTCGGACACACCGCCAACGCTCAGTGCGGTGCTACCGACCCGAGGTCAAGTCCCGGGGGAGGAGAAGAGAGGCTTCCCG-CCTAGAGCATTTGCAAGTCAGGATTCTCTAATCCCTCTGGGAGAAGGGTATTCGGCTTGTCCGCTGT-TTTT

>MG298908 Human gammaherpesvirus 4 isolate RK_LCL_H16

AGGACAGCCGTTGCCCTAGTGGTTTCGGACACACCGCCAACGCTCAGTGCGGTGCTACCGACCCGAGGTCAAGTCCCGGGGGAGGAGAAGAGAGGCTTCCCG-CCTAGAGCATTTGCAAGTCAGGATTCTCTAATCCCTCTGGGAGAAGGGTATTCGGCTTGTCCGCTGT-TTTT

>MG298909 Human gammaherpesvirus 4 isolate RK_LCL_H35

AGGACAGCCGTTGCCCTAGTGGTTTCGGACACACCGCCAACGCTCAGTGCGGTGCTACCGACCCGAGGTCAAGTCCCGGGGGAGGAGAAGAGAGGCTTCCCG-CCTAGAGCATTTGCAAGTCAGGATTCTCTAATCCCTCTGGGAGAAGGGTATTCGGCTTGTCCGCTGT-TTTT

>MG298910 Human gammaherpesvirus 4 isolate RK_LCL_L12

AGGACAGCCGTTGCCCTAGTGGTTTCGGACACACCGCCAACGCTCAGTGCGGTGCTACCGACCCGAGGTCAAGTCCCGGGGGAGGAGAAGAGAGGCTTCCCG-CCTAGAGCATTTGCAAGTCAGGATTCTCTAATCCCTCTGGGAGAAGGGTATTCGGCTTGTCCGCTGT-TTTT

>MG298911 Human gammaherpesvirus 4 isolate RK_LCL_L19

AGGACAGCCGTTGCCCTAGTGGTTTCGGACACACCGCCAACGCTCAGTGCGGTGCTACCGACCCGAGGTCAAGTCCCGGGGGAGGAGAAGAGAGGCTTCCCG-CCTAGAGCATTTGCAAGTCAGGATTCTCTAATCCCTCTGGGAGAAGGGTATTCGGCTTGTCCGCTGT-TTTT

>MG298917 Human gammaherpesvirus 4 strain Epstein-Barr virus isolate DF_Tonsil_T156_BWA

AGGACAGCCGTTGCCCTAGTGGTTTCGGACACACCGCCAACGCTCAGTGCGGTGCTACCGACCCGAGGTCAAGTCCCGGGGGAGGAGAAGAGAGGCTTCCCG-CCTAGAGCATTTGCAAGTCAGGATTCTCTAATCCCTCTGGGAGAAGGGTATTCGGCTTGTCCGCTGT-TTTT

>MG298918 Human gammaherpesvirus 4 strain Epstein-Barr virus isolate GK_LY91_BWA

AGGACAGCCGTTGCCCTAGTGGTTTCGGACACACCGCCAACGCTCAGTGCGGTGCTACCGACCCGAGGTCAAGTCCCGGGGGAGGAGAAGAGAGGCTTCCCG-CCTAGAGCATTTGCAAGTCAGGATTCTCTAATCCCTCTGGGAGAAGGGTATTCGGCTTGTCCGCTGT-TTTT

>MG298919 Human gammaherpesvirus 4 strain Epstein-Barr virus isolate IMS_Saliva_155_BWA

AGGACAGCCGTTGCCCTAGTGGTTTCGGACACACCGCCAACGCTCAGTGCGGTGCTACCGACCCGAGGTCAAGTCCCGGGGGAGGAGAAGAGAGGCTTCCCG-CCTAGAGCATTTGCAAGTCAGGATTCTCTAATCCCTCTGGGAGAAGGGTATTCGGCTTGTCCGCTGT-TTTT

>MG298920 Human gammaherpesvirus 4 strain Epstein-Barr virus isolate IMS_Saliva_187_BWA

AGGACAGCCGTTGCCCTAGTGGTTTCGGACACACCGCCAACGCTCAGTGCGGTGCTACCGACCCGAGGTCAAGTCCCGGGGGAGGAGAAGAGAGGCTTCCCG-CCTAGAGCATTTGCAAGTCAGGATTCTCTAATCCCTCTGGGAGAAGGGTATTCGGCTTGTCCGCTGT-TTTT

>MG298921 Human gammaherpesvirus 4 strain Epstein-Barr virus isolate IMS_Saliva_216_BWA

AGGACAGCCGTTGCCCTAGTGGTTTCGGACACACCGCCAACGCTCAGTGCGGTGCTACCGACCCGAGGTCAAGTCCCGGGGGAGGAGAAGAGAGGCTTCCCG-CCTAGAGCATTTGCAAGTCAGGATTCTCTAATCCCTCTGGGAGAAGGGTATTCGGCTTGTCCGCTGT-TTTT

>MG298922 Human gammaherpesvirus 4 strain Epstein-Barr virus isolate IMS_Saliva_5_BWA

AGGACAGCCGTTGCCCTAGTGGTTTCGGACACACCGCCAACGCTCAGTGCGGTGCTACCGACCCGAGGTCAAGTCCCGGGGGAGGAGAAGAGAGGCTTCCCG-CCTAGAGCATTTGCAAGTCAGGATTCTCTAATCCCTCTGGGAGAAGGGTATTCGGCTTGTCCGCTGT-TTTT

>MG298923 Human gammaherpesvirus 4 strain Epstein-Barr virus isolate IMS_Saliva_81_BWA

AGGACAGCCGTTGCCCTAGTGGTTTCGGACACACCGCCAACGCTCAGTGCGGTGCTACCGACCCGAGGTCAAGTCCCGGGGGAGGAGAAGAGAGGCTTCCCG-CCTAGAGCATTTGCAAGTCAGGATTCTCTAATCCCTCTGGGAGAAGGGTATTCGGCTTGTCCGCTGT-TTTT

>MG298924 Human gammaherpesvirus 4 strain Epstein-Barr virus isolate IMS_Saliva_90_BWA

AGGACAGCCGTTGCCCTAGTGGTTTCGGACACACCGCCAACGCTCAGTGCGGTGCTACCGACCCGAGGTCAAGTCCCGGGGGAGGAGAAGAGAGGCTTCCCG-CCTAGAGCATTTGCAAGTCAGGATTCTCTAATCCCTCTGGGAGAAGGGTATTCGGCTTGTCCGCTGT-TTTT

>MG298926 Human gammaherpesvirus 4 strain Epstein-Barr virus isolate JC_039_BWA

AGGACAGCCGTTGCCCTAGTGGTTTCGGACACACCGCCAACGCTCAGTGCGGTGCTACCGACCCGAGGTCAAGTCCCGGGGGAGGAGAAGAGAGGCTTCCCG-CCTAGAGCATTTGCAAGTCAGGATTCTCTAATCCCTCTGGGAGAAGGGTATTCGGCTTGTCCGCTGT-TTTT

>MG298927 Human gammaherpesvirus 4 strain Epstein-Barr virus isolate JC_VID14_BWA

AGGACAGCCGTTGCCCTAGTGGTTTCGGACACACCGCCAACGCTCAGTGCGGTGCTACCGACCCGAGGTCAAGTCCCGGGGGAGGAGAAGAGAGGCTTCCCG-CCTAGAGCATTTGCAAGTCAGGATTCTCTAATCCCTCTGGGAGAAGGGTATTCGGCTTGTCCGCTGT-TTTT

>MG298928 Human gammaherpesvirus 4 strain Epstein-Barr virus isolate JC_VID41_BWA

AGGACAGCCGTTGCCCTAGTGGTTTCGGACACACCGCCAACGCTCAGTGCGGTGCTACCGACCCGAGGTCAAGTCCCGGGGGAGGAGAAGAGAGGCTTCCCG-CCTAGAGCATTTGCAAGTCAGGATTCTCTAATCCCTCTGGGAGAAGGGTATTCGGCTTGTCCGCTGT-TTTT

>MH144213 Human gammaherpesvirus 4 isolate NKTCL-SG02

AGGACAGCCGTTGCCCTAGTGGTTTCGGACACACCGCCAACGCTCAGTGCGGTGCTACCGACCCGAGGTCAAGTCCCGGGGGAGGAGAAGAGAGGCTTCCCG-CCTAGAGCATTTGCAAGTCAGGATTCTCTAATCCCTCTGGGAGAAGGGTATTCGGCTTGTCCGCTGT-TTTT

>MH144214 Human gammaherpesvirus 4 isolate NKTCL-SG03

AGGACAGCCGTTGCCCTAGTGGTTTCGGACACACCGCCAACGCTCAGTGCGGTGCTACCGACCCGAGGTCAAGTCCCGGGGGAGGAGAAGAGAGGCTTCCCG-CCTAGAGCATTTGCAAGTCAGGATTCTCTAATCCCTCTGGGAGAAGGGTATTCGGCTTGTCCGCTGT-TTTT

>MH144215 Human gammaherpesvirus 4 isolate NKTCL-SG04

AGGACAGCCGTTGCCCTAGTGGTTTCGGACACACCGCCAACGCTCAGTGCGGTGCTACCGACCCGAGGTCAAGTCCCGGGGGAGGAGAAGAGAGGCTTCCCG-CCTAGAGCATTTGCAAGTCAGGATTCTCTAATCCCTCTGGGAGAAGGGTATTCGGCTTGTCCGCTGT-TTTT

>MH144216 Human gammaherpesvirus 4 isolate NKTCL-SG05

AGGACAGCCGTTGCCCTAGTGGTTTCGGACACACCGCCAACGCTCAGTGCGGTGCTACCGACCCGAGGTCAAGTCCCGGGGGAGGAGAAGAGAGGCTTCCCG-CCTAGAGCATTTGCAAGTCAGGATTCTCTAATCCCTCTGGGAGAAGGGTATTCGGCTTGTCCGCTTT-TTTT

>MH144217 Human gammaherpesvirus 4 isolate NKTCL-SG06

AGGACAGCCGTTGCCCTAGTGGTTTCGGACACACCGCCAACGCTCAGTGCGGTGCTACCGACCCGAGGTCAAGTCCCGGGGGAGGAGAAGAGAGGCTTCCCG-CCTAGAGCATTTGCAAGTCAGGATTCTCTAATCCCTCTGGGAGAAGGGTATTCGGCTTGTCCGCTGT-TTTT

>MH144218 Human gammaherpesvirus 4 isolate NKTCL-SG07

AGGACAGCCGTTGCCCTAGTGGTTTCGGACACACCGCCAACGCTCAGTGCGGTGCTACCGACCCGAGGTCAAGTCCCGGGGGAGGAGAAGAGAGGCTTCCCG-CCTAGAGCATTTGCAAGTCAGGATTCTCTAATCCCTCTGGGAGAAGGGTATTCGGCTTGTCCGCTGT-TTTT

>MH144219 Human gammaherpesvirus 4 isolate NKTCL-SG08

AGGACAGCCGTTGCCCTAGTGGTTTCGGACACACCGCCAACGCTCAGTGCGGTGCTACCGACCCGAGGTCAAGTCCCGGGGGAGGAGAAGAGAGGCTTCCCG-CCTAGAGCATTTGCAAGTCAGGATTCTCTAATCCCTCTGGGAGAAGGGTATTCGGCTTGTCCGCTGT-TTTT

>MH144220 Human gammaherpesvirus 4 isolate NKTCL-SG09

AGGACAGCCGTTGCCCTAGTGGTTTCGGACACACCGCCAACGCTCAGTGCGGTGCTACCGACCCGAGGTCAAGTCCCGGGGGAGGAGAAGAGAGGCTTCCCG-CCTAGAGCATTTGCAAGTCAGGATTCTCTAATCCCTCTGGGAGAAGGGTATTCGGCTTGTCCGCTGT-TTTT

>MH144221 Human gammaherpesvirus 4 isolate NKTCL-SG10

AGGACAGCCGTTGCCCTAGTGGTTTCGGACACACCGCCAACGCTCAGTGCGGTGCTACCGACCCGAGGTCAAGTCCCGGGGGAGGAGAAGAGAGGCTTCCCG-CCTAGAGCATTTGCAAGTCAGGATTCTCTAATCCCTCTGGGAGAAGGGTATTCGGCTTGTCCGCTGT-TTTT

>MH144222 Human gammaherpesvirus 4 isolate NKTCL-SG11

AGGACAGCCGTTGCCCTAGTGGTTTCGGACACACCGCCAACGCTCAGTGCGGTGCTACCGACCCGAGGTCAAGTCCCGGGGGAGGAGAAGAGAGGCTTCCCG-CCTAGAGCATTTGCAAGTCAGGATTCTCTAATCCCTCTGGGAGAAGGGTATTCGGCTTGTCCGCTGT-TTTT

>MH590373 Human gammaherpesvirus 4 isolate HKHD4

AGGACAGCCGTTGCCCTAGTGGTTTCGGACACACCGCCAACGCTCAGTGCGGTGCTACCGACCCGAGGTCAAGTCCCGGGGGAGGAGAAGAGAGGCTTCCCG-CCTAGAGCATTTGCAAGTCAGGATTCTCTAATCCCTCTGGGAGAAGGGTATTCGGCTTGTCCGCTGT-TTTT

>MH590374 Human gammaherpesvirus 4 isolate HKHD5

AGGACAGCCGTTGCCCTAGTGGTTTCGGACACACCGCCAACGCTCAGTGCGGTGCTACCGACCCGAGGTCAAGTCCCGGGGGAGGAGAAGAGAGGCTTCCCG-CCTAGAGCATTTGCAAGTCAGGATTCTCTAATCCCTCTGGGAGAAGGGTATTCGGCTTGTCCGCTGT-TTTT

>MH590376 Human gammaherpesvirus 4 isolate HKHD7

AGGACAGCCGTTGCCCTAGTGGTTTCGGACACACCGCCAACGCTCAGTGCGGTGCTACCGACCCGAGGTCAAGTCCCGGGGGAGGAGAAGAGAGGCTTCCCG-CCTAGAGCATTTGCAAGTCAGGATTCTCTAATCCCTCTGGGAGAAGGGTATTCGGCTTGTCCGCTGT-TTTT

>MH590378 Human gammaherpesvirus 4 isolate HKHD9

AGGACAGCCGTTGCCCTAGTGGTTTCGGACACACCGCCAACGCTCAGTGCGGTGCTACCGACCCGAGGTCAAGTCCCGGGGGAGGAGAAGAGAGGCTTCCCG-CCTAGAGCATTTGCAAGTCAGGATTCTCTAATCCCTCTGGGAGAAGGGTATTCGGCTTGTCCGCTGT-TTTT

>MH590380 Human gammaherpesvirus 4 isolate HKHD11

AGGACAGCCGTTGCCCTAGTGGTTTCGGACACACCGCCAACGCTCAGTGCGGTGCTACCGACCCGAGGTCAAGTCCCGGGGGAGGAGAAGAGAGGCTTCCCG-CCTAGAGCATTTGCAAGTCAGGATTCTCTAATCCCTCTGGGAGAAGGGTATTCGGCTTGTCCGCTGT-TTTT

>MH590381 Human gammaherpesvirus 4 isolate HKHD12

AGGACAGCCGTTGCCCTAGTGGTTTCGGACACACCGCCAACGCTCAGTGCGGTGCTACCGACCCGAGGTCAAGTCCCGGGGGAGGAGAAGAGAGGCTTCCCG-CCTAGAGCATTTGCAAGTCAGGATTCTCTAATCCCTCTGGGAGAAGGGTATTCGGCTTGTCCGCTTT-TTTT

>MH590382 Human gammaherpesvirus 4 isolate HKHD13

AGGACAGCCGTTGCCCTAGTGGTTTCGGACACACCGCCAACGCTCAGTGCGGTGCTACCGACCCGAGGTCAAGTCCCGGGGGAGGAGAAGAGAGGCTTCCCG-CCTAGAGCATTTGCAAGTCAGGATTCTCTAATCCCTCTGGGAGAAGGGTATTCGGCTTGTCCGCTGT-TTTT

>MH590384 Human gammaherpesvirus 4 isolate HKHD15

AGGACAGCCGTTGCCCTAGTGGTTTCGGACACACCGCCAACGCTCAGTGCGGTGCTACCGACCCGAGGTCAAGTCCCGGGGGAGGAGAAGAGAGGCTTCCCG-CCTAGAGCATTTGCAAGTCAGGATTCTCTAATCCCTCTGGGAGAAGGGTATTCGGCTTGTCCGCTGT-TTTT

>MH590391 Human gammaherpesvirus 4 isolate HKHD22

AGGACAGCCGTTGCCCTAGTGGTTTCGGACACACCGCCAACGCTCAGTGCGGTGCTACCGACCCGAGGTCAAGTCCCGGGGGAGGAGAAGAGAGGCTTCCCG-CCTAGAGCATTTGCAAGTCAGGATTCTCTAATCCCTCTGGGAGAAGGGTATTCGGCTTGTCCGCTTT-TTTT

>MH590393 Human gammaherpesvirus 4 isolate HKHD24

AGGACAGCCGTTGCCCTAGTGGTTTCGGACACACCGCCAACGCTCAGTGCGGTGCTACCGACCCGAGGTCAAGTCCCGGGGGAGGAGAAGAGAGGCTTCCCG-CCTAGAGCATTTGCAAGTCAGGATTCTCTAATCCCTCTGGGAGAAGGGTATTCGGCTTGTCCGCTGT-TTTT

>MH590394 Human gammaherpesvirus 4 isolate HKHD25

AGGACAGCCGTTGCCCTAGTGGTTTCGGACACACCGCCAACGCTCAGTGCGGTGCTACCGACCCGAGGTCAAGTCCCGGGGGAGGAGAAGAGAGGCTTCCCG-CCTAGAGCATTTGCAAGTCAGGATTCTCTAATCCCTCTGGGAGAAGGGTATTCGGCTTGTCCGCTGT-TTTT

>MH590396 Human gammaherpesvirus 4 isolate HKHD27

AGGACAGCCGTTGCCCTAGTGGTTTCGGACACACCGCCAACGCTCAGTGCGGTGCTACCGACCCGAGGTCAAGTCCCGGGGGAGGAGAAGAGAGGCTTCCCG-CCTAGAGCATTTGCAAGTCAGGATTCTCTAATCCCTCTGGGAGAAGGGTATTCGGCTTGTCCGCTGT-TTTT

>MH590398 Human gammaherpesvirus 4 isolate HKHD29

AGGACAGCCGTTGCCCTAGTGGTTTCGGACACACCGCCAACGCTCAGTGCGGTGCTACCGACCCGAGGTCAAGTCCCGGGGGAGGAGAAGAGAGGCTTCCCG-CCTAGAGCATTTGCAAGTCAGGATTCTCTAATCCCTCTGGGAGAAGGGTATTCGGCTTGTCCGCTGT-TTTT

>MH590399 Human gammaherpesvirus 4 isolate HKHD30

AGGACAGCCGTTGCCCTAGTGGTTTCGGACACACCGCCAACGCTCAGTGCGGTGCTACCGACCCGAGGTCAAGTCCCGGGGGAGGAGAAGAGAGGCTTCCCG-CCTAGAGCATTTGCAAGTCAGGATTCTCTAATCCCTCTGGGAGAAGGGTATTCGGCTTGTCCGCTTT-TTTT

>MH590400 Human gammaherpesvirus 4 isolate HKHD31

AGGACAGCCGTTGCCCTAGTGGTTTCGGACACACCGCCAACGCTCAGTGCGGTGCTACCGACCCGAGGTCAAGTCCCGGGGGAGGAGAAGAGAGGCTTCCCG-CCTAGAGCATTTGCAAGTCAGGATTCTCTAATCCCTCTGGGAGAAGGGTATTCGGCTTGTCCGCTGT-TTTT

>MH590401 Human gammaherpesvirus 4 isolate HKHD32

AGGACAGCCGTTGCCCTAGTGGTTTCGGACACACCGCCAACGCTCAGTGCGGTGCTACCGACCCGAGGTCAAGTCCCGGGGGAGGAGAAGAGAGGCTTCCCG-CCTAGAGCATTTGCAAGTCAGGATTCTCTAATCCCTCTGGGAGAAGGGTATTCGGCTTGTCCGCTGT-TTTT

>MH590405 Human gammaherpesvirus 4 isolate HKHD36

AGGACAGCCGTTGCCCTAGTGGTTTCGGACACACCGCCAACGCTCAGTGCGGTGCTACCGACCCGAGGTCAAGTCCCGGGGGAGGAGAAGAGAGGCTTCCCG-CCTAGAGCATTTGCAAGTCAGGATTCTCTAATCCCTCTGGGAGAAGGGTATTCGGCTTGTCCGCTGT-TTTT

>MH590407 Human gammaherpesvirus 4 isolate HKHD38

AGGACAGCCGTTGCCCTAGTGGTTTCGGACACACCGCCAACGCTCAGTGCGGTGCTACCGACCCGAGGTCAAGTCCCGGGGGAGGAGAAGAGAGGCTTCCCG-CCTAGAGCATTTGCAAGTCAGGATTCTCTAATCCCTCTGGGAGAAGGGTATTCGGCTTGTCCGCTGT-TTTT

>MH590410 Human gammaherpesvirus 4 isolate HKHD41

AGGACAGCCGTTGCCCTAGTGGTTTCGGACACACCGCCAACGCTCAGTGCGGTGCTACCGACCCGAGGTCAAGTCCCGGGGGAGGAGAAGAGAGGCTTCCCG-CCTAGAGCATTTGCAAGTCAGGATTCTCTAATCCCTCTGGGAGAAGGGTATTCGGCTTGTCCGCTGT-TTTT

>MH590412 Human gammaherpesvirus 4 isolate HKHD43

AGGACAGCCGTTGCCCTAGTGGTTTCGGACACACCGCCAACGCTCAGTGCGGTGCTACCGACCCGAGGTCAAGTCCCGGGGGAGGAGAAGAGAGGCTTCCCG-CCTAGAGCATTTGCAAGTCAGGATTCTCTAATCCCTCTGGGAGAAGGGTATTCGGCTTGTCCGCTTT-TTTT

>MH590413 Human gammaherpesvirus 4 isolate HKHD44

AGGACAGCCGTTGCCCTAGTGGTTTCGGACACACCGCCAACGCTCAGTGCGGTGCTACCGACCCGAGGTCAAGTCCCGGGGGAGGAGAAGAGAGGCTTCCCG-CCTAGAGCATTTGCAAGTCAGGATTCTCTAATCCCTCTGGGAGAAGGGTATTCGGCTTGTCCGCTGT-TTTT

>MH590415 Human gammaherpesvirus 4 isolate HKHD46

AGGACAGCCGTTGCCCTAGTGGTTTCGGACACACCGCCAACGCTCAGTGCGGTGCTACCGACCCGAGGTCAAGTCCCGGGGGAGGAGAAGAGAGGCTTCCCG-CCTAGAGCATTTGCAAGTCAGGATTCTCTAATCCCTCTGGGAGAAGGGTATTCGGCTTGTCCGCTTT-TTTT

>MH590419 Human gammaherpesvirus 4 isolate HKHD50

AGGACAGCCGTTGCCCTAGTGGTTTCGGACACACCGCCAACGCTCAGTGCGGTGCTACCGACCCGAGGTCAAGTCCCGGGGGAGGAGAAGAGAGGCTTCCCG-CCTAGAGCATTTGCAAGTCAGGATTCTCTAATCCCTCTGGGAGAAGGGTATTCGGCTTGTCCGCTGT-TTTT

>MH590420 Human gammaherpesvirus 4 isolate HKHD51

AGGACAGCCGTTGCCCTAGTGGTTTCGGACACACCGCCAACGCTCAGTGCGGTGCTACCGACCCGAGGTCAAGTCCCGGGGGAGGAGAAGAGAGGCTTCCCG-CCTAGAGCATTTGCAAGTCAGGATTCTCTAATCCCTCTGGGAGAAGGGTATTCGGCTTGTCCGCTGT-TTTT

>MH590421 Human gammaherpesvirus 4 isolate HKHD52

AGGACAGCCGTTGCCCTAGTGGTTTCGGACACACCGCCAACGCTCAGTGCGGTGCTACCGACCCGAGGTCAAGTCCCGGGGGAGGAGAAGAGAGGCTTCCCG-CCTAGAGCATTTGCAAGTCAGGATTCTCTAATCCCTCTGGGAGAAGGGTATTCGGCTTGTCCGCTGT-TTTT

>MH590422 Human gammaherpesvirus 4 isolate HKHD53

AGGACAGCCGTTGCCCTAGTGGTTTCGGACACACCGCCAACGCTCAGTGCGGTGCTACCGACCCGAGGTCAAGTCCCGGGGGAGGAGAAGAGAGGCTTCCCG-CCTAGAGCATTTGCAAGTCAGGATTCTCTAATCCCTCTGGGAGAAGGGTATTCGGCTTGTCCGCTGT-TTTT

>MH590424 Human gammaherpesvirus 4 isolate HKHD55

AGGACAGCCGTTGCCCTAGTGGTTTCGGACACACCGCCAACGCTCAGTGCGGTGCTACCGACCCGAGGTCAAGTCCCGGGGGAGGAGAAGAGAGGCTTCCCG-CCTAGAGCATTTGCAAGTCAGGATTCTCTAATCCCTCTGGGAGAAGGGTATTCGGCTTGTCCGCTGT-TTTT

>MH590425 Human gammaherpesvirus 4 isolate HKHD56

AGGACAGCCGTTGCCCTAGTGGTTTCGGACACACCGCCAACGCTCAGTGCGGTGCTACCGACCCGAGGTCAAGTCCCGGGGGAGGAGAAGAGAGGCTTCCCG-CCTAGAGCATTTGCAAGTCAGGATTCTCTAATCCCTCTGGGAGAAGGGTATTCGGCTTGTCCGCTGT-TTTT

>MH590427 Human gammaherpesvirus 4 isolate HKHD58

AGGACAGCCGTTGCCCTAGTGGTTTCGGACACACCGCCAACGCTCAGTGCGGTGCTACCGACCCGAGGTCAAGTCCCGGGGGAGGAGAAGAGAGGCTTCCCG-CCTAGAGCATTTGCAAGTCAGGATTCTCTAATCCCTCTGGGAGAAGGGTATTCGGCTTGTCCGCTGT-TTTT

>MH590428 Human gammaherpesvirus 4 isolate HKHD59

AGGACAGCCGTTGCCCTAGTGGTTTCGGACACACCGCCAACGCTCAGTGCGGTGCTACCGACCCGAGGTCAAGTCCCGGGGGAGGAGAAGAGAGGCTTCCCG-CCTAGAGCATTTGCAAGTCAGGATTCTCTAATCCCTCTGGGAGAAGGGTATTCGGCTTGTCCGCTGT-TTTT

>MH590429 Human gammaherpesvirus 4 isolate HKHD60

AGGACAGCCGTTGCCCTAGTGGTTTCGGACACACCGCCAACGCTCAGTGCGGTGCTACCGACCCGAGGTCAAGTCCCGGGGGAGGAGAAGAGAGGCTTCCCG-CCTAGAGCATTTGCAAGTCAGGATTCTCTAATCCCTCTGGGAGAAGGGTATTCGGCTTGTCCGCTGT-TTTT

>MH590430 Human gammaherpesvirus 4 isolate HKHD61

AGGACAGCCGTTGCCCTAGTGGTTTCGGACACACCGCCAACGCTCAGTGCGGTGCTACCGACCCGAGGTCAAGTCCCGGGGGAGGAGAAGAGAGGCTTCCCG-CCTAGAGCATTTGCAAGTCAGGATTCTCTAATCCCTCTGGGAGAAGGGTATTCGGCTTGTCCGCTGT-TTTT

>MH590431 Human gammaherpesvirus 4 isolate HKHD62

AGGACAGCCGTTGCCCTAGTGGTTTCGGACACACCGCCAACGCTCAGTGCGGTGCTACCGACCCGAGGTCAAGTCCCGGGGGAGGAGAAGAGAGGCTTCCCG-CCTAGAGCATTTGCAAGTCAGGATTCTCTAATCCCTCTGGGAGAAGGGTATTCGGCTTGTCCGCTGT-TTTT

>MH590435 Human gammaherpesvirus 4 isolate HKHD66

AGGACAGCCGTTGCCCTAGTGGTTTCGGACACACCGCCAACGCTCAGTGCGGTGCTACCGACCCGAGGTCAAGTCCCGGGGGAGGAGAAGAGAGGCTTCCCG-CCTAGAGCATTTGCAAGTCAGGATTCTCTAATCCCTCTGGGAGAAGGGTATTCGGCTTGTCCGCTGT-TTTT

>MH590436 Human gammaherpesvirus 4 isolate HKHD67

AGGACAGCCGTTGCCCTAGTGGTTTCGGACACACCGCCAACGCTCAGTGCGGTGCTACCGACCCGAGGTCAAGTCCCGGGGGAGGAGAAGAGAGGCTTCCCG-CCTAGAGCATTTGCAAGTCAGGATTCTCTAATCCCTCTGGGAGAAGGGTATTCGGCTTGTCCGCTGT-TTTT

>MH590437 Human gammaherpesvirus 4 isolate HKHD68

AGGACAGCCGTTGCCCTAGTGGTTTCGGACACACCGCCAACGCTCAGTGCGGTGCTACCGACCCGAGGTCAAGTCCCGGGGGAGGAGAAGAGAGGCTTCCCG-CCTAGAGCATTTGCAAGTCAGGATTCTCTAATCCCTCTGGGAGAAGGGTATTCGGCTTGTCCGCTGT-TTTT

>MH590438 Human gammaherpesvirus 4 isolate HKHD69

AGGACAGCCGTTGCCCTAGTGGTTTCGGACACACCGCCAACGCTCAGTGCGGTGCTACCGACCCGAGGTCAAGTCCCGGGGGAGGAGAAGAGAGGCTTCCCG-CCTAGAGCATTTGCAAGTCAGGATTCTCTAATCCCTCTGGGAGAAGGGTATTCGGCTTGTCCGCTGT-TTTT

>MH590439 Human gammaherpesvirus 4 isolate HKHD70

AGGACAGCCGTTGCCCTAGTGGTTTCGGACACACCGCCAACGCTCAGTGCGGTGCTACCGACCCGAGGTCAAGTCCCGGGGGAGGAGAAGAGAGGCTTCCCG-CCTAGAGCATTTGCAAGTCAGGATTCTCTAATCCCTCTGGGAGAAGGGTATTCGGCTTGTCCGCTGT-TTTT

>MH590441 Human gammaherpesvirus 4 isolate HKHD72

AGGACAGCCGTTGCCCTAGTGGTTTCGGACACACCGCCAACGCTCAGTGCGGTGCTACCGACCCGAGGTCAAGTCCCGGGGGAGGAGAAGAGAGGCTTCCCG-CCTAGAGCATTTGCAAGTCAGGATTCTCTAATCCCTCTGGGAGAAGGGTATTCGGCTTGTCCGCTGT-TTTT

>MH590443 Human gammaherpesvirus 4 isolate HKHD74

AGGACAGCCGTTGCCCTAGTGGTTTCGGACACACCGCCAACGCTCAGTGCGGTGCTACCGACCCGAGGTCAAGTCCCGGGGGAGGAGAAGAGAGGCTTCCCG-CCTAGAGCATTTGCAAGTCAGGATTCTCTAATCCCTCTGGGAGAAGGGTATTCGGCTTGTCCGCTGT-TTTT

>MH590444 Human gammaherpesvirus 4 isolate HKHD75

AGGACAGCCGTTGCCCTAGTGGTTTCGGACACACCGCCAACGCTCAGTGCGGTGCTACCGACCCGAGGTCAAGTCCCGGGGGAGGAGAAGAGAGGCTTCCCG-CCTAGAGCATTTGCAAGTCAGGATTCTCTAATCCCTCTGGGAGAAGGGTATTCGGCTTGTCCGCTGT-TTTT

>MH590445 Human gammaherpesvirus 4 isolate HKHD76

AGGACAGCCGTTGCCCTAGTGGTTTCGGACACACCGCCAACGCTCAGTGCGGTGCTACCGACCCGAGGTCAAGTCCCGGGGGAGGAGAAGAGAGGCTTCCCG-CCTAGAGCATTTGCAAGTCAGGATTCTCTAATCCCTCTGGGAGAAGGGTATTCGGCTTGTCCGCTGT-TTTT

>MH590446 Human gammaherpesvirus 4 isolate HKHD77

AGGACAGCCGTTGCCCTAGTGGTTTCGGACACACCGCCAACGCTCAGTGCGGTGCTACCGACCCGAGGTCAAGTCCCGGGGGAGGAGAAGAGAGGCTTCCCG-CCTAGAGCATTTGCAAGTCAGGATTCTCTAATCCCTCTGGGAGAAGGGTATTCGGCTTGTCCGCTGT-TTTT

>MH590448 Human gammaherpesvirus 4 isolate HKHD79

AGGACAGCCGTTGCCCTAGTGGTTTCGGACACACCGCCAACGCTCAGTGCGGTGCTACCGACCCGAGGTCAAGTCCCGGGGGAGGAGAAGAGAGGCTTCCCG-CCTAGAGCATTTGCAAGTCAGGATTCTCTAATCCCTCTGGGAGAAGGGTATTCGGCTTGTCCGCTGT-TTTT

>MH590449 Human gammaherpesvirus 4 isolate HKHD80

AGGACAGCCGTTGCCCTAGTGGTTTCGGACACACCGCCAACGCTCAGTGCGGTGCTACCGACCCGAGGTCAAGTCCCGGGGGAGGAGAAGAGAGGCTTCCCG-CCTAGAGCATTTGCAAGTCAGGATTCTCTAATCCCTCTGGGAGAAGGGTATTCGGCTTGTCCGCTGT-TTTT

>MH590452 Human gammaherpesvirus 4 isolate HKHD83

AGGACAGCCGTTGCCCTAGTGGTTTCGGACACACCGCCAACGCTCAGTGCGGTGCTACCGACCCGAGGTCAAGTCCCGGGGGAGGAGAAGAGAGGCTTCCCG-CCTAGAGCATTTGCAAGTCAGGATTCTCTAATCCCTCTGGGAGAAGGGTATTCGGCTTGTCCGCTGT-TTTT

>MH590453 Human gammaherpesvirus 4 isolate HKHD84

AGGACAGCCGTTGCCCTAGTGGTTTCGGACACACCGCCAACGCTCAGTGCGGTGCTACCGACCCGAGGTCAAGTCCCGGGGGAGGAGAAGAGAGGCTTCCCG-CCTAGAGCATTTGCAAGTCAGGATTCTCTAATCCCTCTGGGAGAAGGGTATTCGGCTTGTCCGCTGT-TTTT

>MH590458 Human gammaherpesvirus 4 isolate HKHD89

AGGACAGCCGTTGCCCTAGTGGTTTCGGACACACCGCCAACGCTCAGTGCGGTGCTACCGACCCGAGGTCAAGTCCCGGGGGAGGAGAAGAGAGGCTTCCCG-CCTAGAGCATTTGCAAGTCAGGATTCTCTAATCCCTCTGGGAGAAGGGTATTCGGCTTGTCCGCTGT-TTTT

>MH590459 Human gammaherpesvirus 4 isolate HKHD90

AGGACAGCCGTTGCCCTAGTGGTTTCGGACACACCGCCAACGCTCAGTGCGGTGCTACCGACCCGAGGTCAAGTCCCGGGGGAGGAGAAGAGAGGCTTCCCG-CCTAGAGCATTTGCAAGTCAGGATTCTCTAATCCCTCTGGGAGAAGGGTATTCGGCTTGTCCGCTGT-TTTT

>MH590464 Human gammaherpesvirus 4 isolate HKHD95

AGGACAGCCGTTGCCCTAGTGGTTTCGGACACACCGCCAACGCTCAGTGCGGTGCTACCGACCCGAGGTCAAGTCCCGGGGGAGGAGAAGAGAGGCTTCCCG-CCTAGAGCATTTGCAAGTCAGGATTCTCTAATCCCTCTGGGAGAAGGGTATTCGGCTTGTCCGCTGT-TTTT

>MH590465 Human gammaherpesvirus 4 isolate HKHD96

AGGACAGCCGTTGCCCTAGTGGTTTCGGACACACCGCCAACGCTCAGTGCGGTGCTACCGACCCGAGGTCAAGTCCCGGGGGAGGAGAAGAGAGGCTTCCCG-CCTAGAGCATTTGCAAGTCAGGATTCTCTAATCCCTCTGGGAGAAGGGTATTCGGCTTGTCCGCTGT-TTTT

>MH590466 Human gammaherpesvirus 4 isolate HKHD97

AGGACAGCCGTTGCCCTAGTGGTTTCGGACACACCGCCAACGCTCAGTGCGGTGCTACCGACCCGAGGTCAAGTCCCGGGGGAGGAGAAGAGAGGCTTCCCG-CCTAGAGCATTTGCAAGTCAGGATTCTCTAATCCCTCTGGGAGAAGGGTATTCGGCTTGTCCGCTGT-TTTT

>MH590467 Human gammaherpesvirus 4 isolate HKHD98

AGGACAGCCGTTGCCCTAGTGGTTTCGGACACACCGCCAACGCTCAGTGCGGTGCTACCGACCCGAGGTCAAGTCCCGGGGGAGGAGAAGAGAGGCTTCCCG-CCTAGAGCATTTGCAAGTCAGGATTCTCTAATCCCTCTGGGAGAAGGGTATTCGGCTTGTCCGCTGT-TTTT

>MH590468 Human gammaherpesvirus 4 isolate HKHD99

AGGACAGCCGTTGCCCTAGTGGTTTCGGACACACCGCCAACGCTCAGTGCGGTGCTACCGACCCGAGGTCAAGTCCCGGGGGAGGAGAAGAGAGGCTTCCCG-CCTAGAGCATTTGCAAGTCAGGATTCTCTAATCCCTCTGGGAGAAGGGTATTCGGCTTGTCCGCTGT-TTTT

>MH590470 Human gammaherpesvirus 4 isolate HKHD101

AGGACAGCCGTTGCCCTAGTGGTTTCGGACACACCGCCAACGCTCAGTGCGGTGCTACCGACCCGAGGTCAAGTCCCGGGGGAGGAGAAGAGAGGCTTCCCG-CCTAGAGCATTTGCAAGTCAGGATTCTCTAATCCCTCTGGGAGAAGGGTATTCGGCTTGTCCGCTGT-TTTT

>MH590472 Human gammaherpesvirus 4 isolate HKHD103

AGGACAGCCGTTGCCCTAGTGGTTTCGGACACACCGCCAACGCTCAGTGCGGTGCTACCGACCCGAGGTCAAGTCCCGGGGGAGGAGAAGAGAGGCTTCCCG-CCTAGAGCATTTGCAAGTCAGGATTCTCTAATCCCTCTGGGAGAAGGGTATTCGGCTTGTCCGCTGT-TTTT

>MH590473 Human gammaherpesvirus 4 isolate HKHD104

AGGACAGCCGTTGCCCTAGTGGTTTCGGACACACCGCCAACGCTCAGTGCGGTGCTACCGACCCGAGGTCAAGTCCCGGGGGAGGAGAAGAGAGGCTTCCCG-CCTAGAGCATTTGCAAGTCAGGATTCTCTAATCCCTCTGGGAGAAGGGTATTCGGCTTGTCCGCTGT-TTTT

>MH590475 Human gammaherpesvirus 4 isolate HKHD106

AGGACAGCCGTTGCCCTAGTGGTTTCGGACACACCGCCAACGCTCAGTGCGGTGCTACCGACCCGAGGTCAAGTCCCGGGGGAGGAGAAGAGAGGCTTCCCG-CCTAGAGCATTTGCAAGTCAGGATTCTCTAATCCCTCTGGGAGAAGGGTATTCGGCTTGTCCGCTGT-TTTT

>MH590476 Human gammaherpesvirus 4 isolate HKHD107

AGGACAGCCGTTGCCCTAGTGGTTTCGGACACACCGCCAACGCTCAGTGCGGTGCTACCGACCCGAGGTCAAGTCCCGGGGGAGGAGAAGAGAGGCTTCCCG-CCTAGAGCATTTGCAAGTCAGGATTCTCTAATCCCTCTGGGAGAAGGGTATTCGGCTTGTCCGCTGT-TTTT

>MH590478 Human gammaherpesvirus 4 isolate HKHD109

AGGACAGCCGTTGCCCTAGTGGTTTCGGACACACCGCCAACGCTCAGTGCGGTGCTACCGACCCGAGGTCAAGTCCCGGGGGAGGAGAAGAGAGGCTTCCCG-CCTAGAGCATTTGCAAGTCAGGATTCTCTAATCCCTCTGGGAGAAGGGTATTCGGCTTGTCCGCTGT-TTTT

>MH590481 Human gammaherpesvirus 4 isolate HKHD112

AGGACAGCCGTTGCCCTAGTGGTTTCGGACACACCGCCAACGCTCAGTGCGGTGCTACCGACCCGAGGTCAAGTCCCGGGGGAGGAGAAGAGAGGCTTCCCG-CCTAGAGCATTTGCAAGTCAGGATTCTCTAATCCCTCTGGGAGAAGGGTATTCGGCTTGTCCGCTGT-TTTT

>MH590482 Human gammaherpesvirus 4 isolate HKHD113

AGGACAGCCGTTGCCCTAGTGGTTTCGGACACACCGCCAACGCTCAGTGCGGTGCTACCGACCCGAGGTCAAGTCCCGGGGGAGGAGAAGAGAGGCTTCCCG-CCTAGAGCATTTGCAAGTCAGGATTCTCTAATCCCTCTGGGAGAAGGGTATTCGGCTTGTCCGCTGT-TTTT

>MH590483 Human gammaherpesvirus 4 isolate HKHD114

AGGACAGCCGTTGCCCTAGTGGTTTCGGACACACCGCCAACGCTCAGTGCGGTGCTACCGACCCGAGGTCAAGTCCCGGGGGAGGAGAAGAGAGGCTTCCCG-CCTAGAGCATTTGCAAGTCAGGATTCTCTAATCCCTCTGGGAGAAGGGTATTCGGCTTGTCCGCTGT-TTTT

>MH590484 Human gammaherpesvirus 4 isolate HKHD115

AGGACAGCCGTTGCCCTAGTGGTTTCGGACACACCGCCAACGCTCAGTGCGGTGCTACCGACCCGAGGTCAAGTCCCGGGGGAGGAGAAGAGAGGCTTCCCG-CCTAGAGCATTTGCAAGTCAGGATTCTCTAATCCCTCTGGGAGAAGGGTATTCGGCTTGTCCGCTGT-TTTT

>MH590485 Human gammaherpesvirus 4 isolate HKHD116

AGGACAGCCGTTGCCCTAGTGGTTTCGGACACACCGCCAACGCTCAGTGCGGTGCTACCGACCCGAGGTCAAGTCCCGGGGGAGGAGAAGAGAGGCTTCCCG-CCTAGAGCATTTGCAAGTCAGGATTCTCTAATCCCTCTGGGAGAAGGGTATTCGGCTTGTCCGCTGT-TTTT

>MH590486 Human gammaherpesvirus 4 isolate HKHD117

AGGACAGCCGTTGCCCTAGTGGTTTCGGACACACCGCCAACGCTCAGTGCGGTGCTACCGACCCGAGGTCAAGTCCCGGGGGAGGAGAAGAGAGGCTTCCCG-CCTAGAGCATTTGCAAGTCAGGATTCTCTAATCCCTCTGGGAGAAGGGTATTCGGCTTGTCCGCTGT-TTTT

>MH590487 Human gammaherpesvirus 4 isolate HKHD118

AGGACAGCCGTTGCCCTAGTGGTTTCGGACACACCGCCAACGCTCAGTGCGGTGCTACCGACCCGAGGTCAAGTCCCGGGGGAGGAGAAGAGAGGCTTCCCG-CCTAGAGCATTTGCAAGTCAGGATTCTCTAATCCCTCTGGGAGAAGGGTATTCGGCTTGTCCGCTGT-TTTT

>MH590488 Human gammaherpesvirus 4 isolate HKHD119

AGGACAGCCGTTGCCCTAGTGGTTTCGGACACACCGCCAACGCTCAGTGCGGTGCTACCGACCCGAGGTCAAGTCCCGGGGGAGGAGAAGAGAGGCTTCCCG-CCTAGAGCATTTGCAAGTCAGGATTCTCTAATCCCTCTGGGAGAAGGGTATTCGGCTTGTCCGCTGT-TTTT

>MH590489 Human gammaherpesvirus 4 isolate HKHD120

AGGACAGCCGTTGCCCTAGTGGTTTCGGACACACCGCCAACGCTCAGTGCGGTGCTACCGACCCGAGGTCAAGTCCCGGGGGAGGAGAAGAGAGGCTTCCCG-CCTAGAGCATTTGCAAGTCAGGATTCTCTAATCCCTCTGGGAGAAGGGTATTCGGCTTGTCCGCTGT-TTTT

>MH590490 Human gammaherpesvirus 4 isolate HKHD121

AGGACAGCCGTTGCCCTAGTGGTTTCGGACACACCGCCAACGCTCAGTGCGGTGCTACCGACCCGAGGTCAAGTCCCGGGGGAGGAGAAGAGAGGCTTCCCG-CCTAGAGCATTTGCAAGTCAGGATTCTCTAATCCCTCTGGGAGAAGGGTATTCGGCTTGTCCGCTGT-TTTT

>MH590491 Human gammaherpesvirus 4 isolate HKHD122

AGGACAGCCGTTGCCCTAGTGGTTTCGGACACACCGCCAACGCTCAGTGCGGTGCTACCGACCCGAGGTCAAGTCCCGGGGGAGGAGAAGAGAGGCTTCCCG-CCTAGAGCATTTGCAAGTCAGGATTCTCTAATCCCTCTGGGAGAAGGGTATTCGGCTTGTCCGCTGT-TTTT

>MH590492 Human gammaherpesvirus 4 isolate HKHD123

AGGACAGCCGTTGCCCTAGTGGTTTCGGACACACCGCCAACGCTCAGTGCGGTGCTACCGACCCGAGGTCAAGTCCCGGGGGAGGAGAAGAGAGGCTTCCCG-CCTAGAGCATTTGCAAGTCAGGATTCTCTAATCCCTCTGGGAGAAGGGTATTCGGCTTGTCCGCTGT-TTTT

>MH590493 Human gammaherpesvirus 4 isolate HKHD124

AGGACAGCCGTTGCCCTAGTGGTTTCGGACACACCGCCAACGCTCAGTGCGGTGCTACCGACCCGAGGTCAAGTCCCGGGGGAGGAGAAGAGAGGCTTCCCG-CCTAGAGCATTTGCAAGTCAGGATTCTCTAATCCCTCTGGGAGAAGGGTATTCGGCTTGTCCGCTGT-TTTT

>MH590494 Human gammaherpesvirus 4 isolate HKHD125

AGGACAGCCGTTGCCCTAGTGGTTTCGGACACACCGCCAACGCTCAGTGCGGTGCTACCGACCCGAGGTCAAGTCCCGGGGGAGGAGAAGAGAGGCTTCCCG-CCTAGAGCATTTGCAAGTCAGGATTCTCTAATCCCTCTGGGAGAAGGGTATTCGGCTTGTCCGCTGT-TTTT

>MH590495 Human gammaherpesvirus 4 isolate HKHD126

AGGACAGCCGTTGCCCTAGTGGTTTCGGACACACCGCCAACGCTCAGTGCGGTGCTACCGACCCGAGGTCAAGTCCCGGGGGAGGAGAAGAGAGGCTTCCCG-CCTAGAGCATTTGCAAGTCAGGATTCTCTAATCCCTCTGGGAGAAGGGTATTCGGCTTGTCCGCTGT-TTTT

>MH590496 Human gammaherpesvirus 4 isolate HKHD127

AGGACAGCCGTTGCCCTAGTGGTTTCGGACACACCGCCAACGCTCAGTGCGGTGCTACCGACCCGAGGTCAAGTCCCGGGGGAGGAGAAGAGAGGCTTCCCG-CCTAGAGCATTTGCAAGTCAGGATTCTCTAATCCCTCTGGGAGAAGGGTATTCGGCTTGTCCGCTGT-TTTT

>MH590503 Human gammaherpesvirus 4 isolate HKHD134

AGGACAGCCGTTGCCCTAGTGGTTTCGGACACACCGCCAACGCTCAGTGCGGTGCTACCGACCCGAGGTCAAGTCCCGGGGGAGGAGAAGAGAGGCTTCCCG-CCTAGAGCATTTGCAAGTCAGGATTCTCTAATCCCTCTGGGAGAAGGGTATTCGGCTTGTCCGCTGT-TTTT

>MH590505 Human gammaherpesvirus 4 isolate HKHD136

AGGACAGCCGTTGCCCTAGTGGTTTCGGACACACCGCCAACGCTCAGTGCGGTGCTACCGACCCGAGGTCAAGTCCCGGGGGAGGAGAAGAGAGGCTTCCCG-CCTAGAGCATTTGCAAGTCAGGATTCTCTAATCCCTCTGGGAGAAGGGTATTCGGCTTGTCCGCTTT-TTTT

>MH590506 Human gammaherpesvirus 4 isolate HKHD137

AGGACAGCCGTTGCCCTAGTGGTTTCGGACACACCGCCAACGCTCAGTGCGGTGCTACCGACCCGAGGTCAAGTCCCGGGGGAGGAGAAGAGAGGCTTCCCG-CCTAGAGCATTTGCAAGTCAGGATTCTCTAATCCCTCTGGGAGAAGGGTATTCGGCTTGTCCGCTGT-TTTT

>MH590507 Human gammaherpesvirus 4 isolate HKHD138

AGGACAGCCGTTGCCCTAGTGGTTTCGGACACACCGCCAACGCTCAGTGCGGTGCTACCGACCCGAGGTCAAGTCCCGGGGGAGGAGAAGAGAGGCTTCCCG-CCTAGAGCATTTGCAAGTCAGGATTCTCTAATCCCTCTGGGAGAAGGGTATTCGGCTTGTCCGCTTT-TTTT

>MH590508 Human gammaherpesvirus 4 isolate HKHD139

AGGACAGCCGTTGCCCTAGTGGTTTCGGACACACCGCCAACGCTCAGTGCGGTGCTACCGACCCGAGGTCAAGTCCCGGGGGAGGAGAAGAGAGGCTTCCCG-CCTAGAGCATTTGCAAGTCAGGATTCTCTAATCCCTCTGGGAGAAGGGTATTCGGCTTGTCCGCTTT-TTTT

>MH590510 Human gammaherpesvirus 4 isolate HKHD141

AGGACAGCCGTTGCCCTAGTGGTTTCGGACACACCGCCAACGCTCAGTGCGGTGCTACCGACCCGAGGTCAAGTCCCGGGGGAGGAGAAGAGAGGCTTCCCG-CCTAGAGCATTTGCAAGTCAGGATTCTCTAATCCCTCTGGGAGAAGGGTATTCGGCTTGTCCGCTGT-TTTT

>MH590511 Human gammaherpesvirus 4 isolate HKHD142

AGGACAGCCGTTGCCCTAGTGGTTTCGGACACACCGCCAACGCTCAGTGCGGTGCTACCGACCCGAGGTCAAGTCCCGGGGGAGGAGAAGAGAGGCTTCCCG-CCTAGAGCATTTGCAAGTCAGGATTCTCTAATCCCTCTGGGAGAAGGGTATTCGGCTTGTCCGCTGT-TTTT

>MH883755 Human gammaherpesvirus 4 isolate ebv6, partial genome

AGGACAGCCGTTGCCCTAGTGGTTTCGGACACACCGCCAACGCTCAGTGCGGTGCTACCGACCCGAGGTCAAGTCCCGGGGGAGGAGAAGAGAGGCTTCCCG-CCTAGAGCATTTGCAAGTCAGGATTCTCTAATCCCTCTGGGAGAAGGGTATTCGGCTTGTCCGCTGT-TTTT

>MH883756 Human gammaherpesvirus 4 isolate ebv7

AGGACAGCCGTTGCCCTAGTGGTTTCGGACACACCGCCAACGCTCAGTGCGGTGCTACCGACCCGAGGTCAAGTCCCGGGGGAGGAGAAGAGAGGCTTCCCG-CCTAGAGCATTTGCAAGTCAGGATTCTCTAATCCCTCTGGGAGAAGGGTATTCGGCTTGTCCGCTGT-TTTT

>MH883757 Human gammaherpesvirus 4 isolate ebv8

AGGACAGCCGTTGCCCTAGTGGTTTCGGACACACCGCCAACGCTCAGTGCGGTGCTACCGACCCGAGGTCAAGTCCCGGGGGAGGAGAAGAGAGGCTTCCCG-CCTAGAGCATTTGCAAGTCAGGATTCTCTAATCCCTCTGGGAGAAGGGTATTCGGCTTGTCCGCTGT-TTTT

>MH883758 Human gammaherpesvirus 4 isolate ebv9

AGGACAGCCGTTGCCCTAGTGGTTTCGGACACACCGCCAACGCTCAGTGCGGTGCTACCGACCCGAGGTCAAGTCCCGGGGGAGGAGAAGAGAGGCTTCCCG-CCTAGAGCATTTGCAAGTCAGGATTCTCTAATCCCTCTGGGAGAAGGGTATTCGGCTTGTCCGCTGT-TTTT

>MH883759 Human gammaherpesvirus 4 isolate ebv13

AGGACAGCCGTTGCCCTAGTGGTTTCGGACACACCGCCAACGCTCAGTGCGGTGCTACCGACCCGAGGTCAAGTCCCGGGGGAGGAGAAGAGAGGCTTCCCG-CCTAGAGCATTTGCAAGTCAGGATTCTCTAATCCCTCTGGGAGAAGGGTATTCGGCTTGTCCGCTGT-TTTT

>MH883761 Human gammaherpesvirus 4 isolate ebv15

AGGACAGCCGTTGCCCTAGTGGTTTCGGACACACCGCCAACGCTCAGTGCGGTGCTACCGACCCGAGGTCAAGTCCCGGGGGAGGAGAAGAGAGGCTTCCCG-CCTAGAGCATTTGCAAGTCAGGATTCTCTAATCCCTCTGGGAGAAGGGTATTCGGCTTGTCCGCTGT-TTTT

>MH883763 Human gammaherpesvirus 4 isolate ebv17

AGGACAGCCGTTGCCCTAGTGGTTTCGGACACACCGCCAACGCTCAGTGCGGTGCTACCGACCCGAGGTCAAGTCCCGGGGGAGGAGAAGAGAGGCTTCCCG-CCTAGAGCATTTGCAAGTCAGGATTCTCTAATCCCTCTGGGAGAAGGGTATTCGGCTTGTCCGCTGT-TTTT

>MH883764 Human gammaherpesvirus 4 isolate ebv19, partial genome

AGGACAGCCGTTGCCCTAGTGGTTTCGGACACACCGCCAACGCTCAGTGCGGTGCTACCGACCCGAGGTCAAGTCCCGGGGGAGGAGAAGAGAGGCTTCCCG-CCTAGAGCATTTGCAAGTCAGGATTCTCTAATCCCTCTGGGAGAAGGGTATTCGGCTTGTCCGCTGT-TTTT

>MH883765 Human gammaherpesvirus 4 isolate ebv21

AGGACAGCCGTTGCCCTAGTGGTTTCGGACACACCGCCAACGCTCAGTGCGGTGCTACCGACCCGAGGTCAAGTCCCGGGGGAGGAGAAGAGAGGCTTCCCG-CCTAGAGCATTTGCAAGTCAGGATTCTCTAATCCCTCTGGGAGAAGGGTATTCGGCTTGTCCGCTGT-TTTT

>MH883766 Human gammaherpesvirus 4 isolate ebv22

AGGACAGCCGTTGCCCTAGTGGTTTCGGACACACCGCCAACGCTCAGTGCGGTGCTACCGACCCGAGGTCAAGTCCCGGGGGAGGAGAAGAGAGGCTTCCCG-CCTAGAGCATTTGCAAGTCAGGATTCTCTAATCCCTCTGGGAGAAGGGTATTCGGCTTGTCCGCTGT-TTTT

>MH883768 Human gammaherpesvirus 4 isolate ebv25

AGGACAGCCGTTGCCCTAGTGGTTTCGGACACACCGCCAACGCTCAGTGCGGTGCTACCGACCCGAGGTCAAGTCCCGGGGGAGGAGAAGAGAGGCTTCCCG-CCTAGAGCATTTGCAAGTCAGGATTCTCTAATCCCTCTGGGAGAAGGGTATTCGGCTTGTCCGCTGT-TTTT

>MH883769 Human gammaherpesvirus 4 isolate ebv27

AGGACAGCCGTTGCCCTAGTGGTTTCGGACACACCGCCAACGCTCAGTGCGGTGCTACCGACCCGAGGTCAAGTCCCGGGGGAGGAGAAGAGAGGCTTCCCG-CCTAGAGCATTTGCAAGTCAGGATTCTCTAATCCCTCTGGGAGAAGGGTATTCGGCTTGTCCGCTGT-TTTT

>MH883770 Human gammaherpesvirus 4 isolate ebv30, partial genome

AGGACAGCCGTTGCCCTAGTGGTTTCGGACACACCGCCAACGCTCAGTGCGGTGCTACCGACCCGAGGTCAAGTCCCGGGGGAGGAGAAGAGAGGCTTCCCG-CCTAGAGCATTTGCAAGTCAGGATTCTCTAATCCCTCTGGGAGAAGGGTATTCGGCTTGTCCGCTGT-TTTT

>MH883771 Human gammaherpesvirus 4 isolate ebv31, partial genome

AGGACAGCCGTTGCCCTAGTGGTTTCGGACACACCGCCAACGCTCAGTGCGGTGCTACCGACCCGAGGTCAAGTCCCGGGGGAGGAGAAGAGAGGCTTCCCG-CCTAGAGCATTTGCAAGTCAGGATTCTCTAATCCCTCTGGGAGAAGGGTATTCGGCTTGTCCGCTGT-TTTT

>MH883772 Human gammaherpesvirus 4 isolate P1-812

AGGACAGCCGTTGCCCTAGTGGTTTCGGACACACCGCCAACGCTCAGTGCGGTGCTACCGACCCGAGGTCAAGTCCCGGGGGAGGAGAAGAGAGGCTTCCCG-CCTAGAGCATTTGCAAGTCAGGATTCTCTAATCCCTCTGGGAGAAGGGTATTCGGCTTGTCCGCTGT-TTTT

>MH883773 Human gammaherpesvirus 4 isolate P1-T1

AGGACAGCCGTTGCCCTAGTGGTTTCGGACACACCGCCAACGCTCAGTGCGGTGCTACCGACCCGAGGTCAAGTCCCGGGGGAGGAGAAGAGAGGCTTCCCG-CCTAGAGCATTTGCAAGTCAGGATTCTCTAATCCCTCTGGGAGAAGGGTATTCGGCTTGTCCGCTGT-TTTT

>MH883774 Human gammaherpesvirus 4 isolate P2-1213

AGGACAGCCGTTGCCCTAGTGGTTTCGGACACACCGCCAACGCTCAGTGCGGTGCTACCGACCCGAGGTCAAGTCCCGGGGGAGGAGAAGAGAGGCTTCCCG-CCTAGAGCATTTGCAAGTCAGGATTCTCTAATCCCTCTGGGAGAAGGGTATTCGGCTTGTCCGCTGT-TTTT

>MH883776 Human gammaherpesvirus 4 isolate P3-2670

AGGACAGCCGTTGCCCTAGTGGTTTCGGACACACCGCCAACGCTCAGTGCGGTGCTACCGACCCGAGGTCAAGTCCCGGGGGAGGAGAAGAGAGGCTTCCCG-CCTAGAGCATTTGCAAGTCAGGATTCTCTAATCCCTCTGGGAGAAGGGTATTCGGCTTGTCCGCTGT-TTTT

>MH883777 Human gammaherpesvirus 4 isolate P3-T1

AGGACAGCCGTTGCCCTAGTGGTTTCGGACACACCGCCAACGCTCAGTGCGGTGCTACCGACCCGAGGTCAAGTCCCGGGGGAGGAGAAGAGAGGCTTCCCG-CCTAGAGCATTTGCAAGTCAGGATTCTCTAATCCCTCTGGGAGAAGGGTATTCGGCTTGTCCGCTGT-TTTT

>MH883779 Human gammaherpesvirus 4 isolate P4-T1

AGGACAGCCGTTGCCCTAGTGGTTTCGGACACACCGCCAACGCTCAGTGCGGTGCTACCGACCCGAGGTCAAGTCCCGGGGGAGGAGAAGAGAGGCTTCCCG-CCTAGAGCATTTGCAAGTCAGGATTCTCTAATCCCTCTGGGAGAAGGGTATTCGGCTTGTCCGCTGT-TTTT

>MH883780 Human gammaherpesvirus 4 isolate P5-1294

AGGACAGCCGTTGCCCTAGTGGTTTCGGACACACCGCCAACGCTCAGTGCGGTGCTACCGACCCGAGGTCAAGTCCCGGGGGAGGAGAAGAGAGGCTTCCCG-CCTAGAGCATTTGCAAGTCAGGATTCTCTAATCCCTCTGGGAGAAGGGTATTCGGCTTGTCCGCTGT-TTTT

>MH883781 Human gammaherpesvirus 4 isolate P6-1751

AGGACAGCCGTTGCCCTAGTGGTTTCGGACACACCGCCAACGCTCAGTGCGGTGCTACCGACCCGAGGTCAAGTCCCGGGGGAGGAGAAGAGAGGCTTCCCG-CCTAGAGCATTTGCAAGTCAGGATTCTCTAATCCCTCTGGGAGAAGGGTATTCGGCTTGTCCGCTGT-TTTT

>MH883782 Human gammaherpesvirus 4 isolate P7-2634, partial genome

AGGACAGCCGTTGCCCTAGTGGTTTCGGACACACCGCCAACGCTCAGTGCGGTGCTACCGACCCGAGGTCAAGTCCCGGGGGAGGAGAAGAGAGGCTTCCCG-CCTAGAGCATTTGCAAGTCAGGATTCTCTAATCCCTCTGGGAGAAGGGTATTCGGCTTGTCCGCTGT-TTTT

>MH883783 Human gammaherpesvirus 4 isolate P8-414

AGGACAGCCGTTGCCCTAGTGGTTTCGGACACACCGCCAACGCTCAGTGCGGTGCTACCGACCCGAGGTCAAGTCCCGGGGGAGGAGAAGAGAGGCTTCCCG-CCTAGAGCATTTGCAAGTCAGGATTCTCTAATCCCTCTGGGAGAAGGGTATTCGGCTTGTCCGCTGT-TTTT

>MH883784 Human gammaherpesvirus 4 isolate P9-2631

AGGACAGCCGTTGCCCTAGTGGTTTCGGACACACCGCCAACGCTCAGTGCGGTGCTACCGACCCGAGGTCAAGTCCCGGGGGAGGAGAAGAGAGGCTTCCCG-CCTAGAGCATTTGCAAGTCAGGATTCTCTAATCCCTCTGGGAGAAGGGTATTCGGCTTGTCCGCTGT-TTTT

>MK540275 Human gammaherpesvirus 4 isolate HS018

AGGACAGCCGTTGCCCTAGTGGTTTCGGACACACCGCCAACGCTCAGTGCGGTGCTACCGACCCGAGGTCGAGTCCCGGGGGAGGAGAAGAGAGGCTTCCCG-CCTAGAGCATTTGCAAGTCAGGATTCTCTAATCCCTCTGGGAGAAGGGTATTCGGCTTGTCCGCTAT-TTTT

>MK540309 Human gammaherpesvirus 4 isolate NNPCT002

AGGACAGCCGTTGCCCTAGTGGTTTCGGACACACCGCCAACGCTCAGTGCGGTGCTACCGACCCGAGATCAAGTCCCGGGGGAGGAGAAGAGAGGCTTCCCG-CCTAGAGCATTTGCAAGTCAGGATTCTCTAATCCCTCTGGGAGAAGGGTATTCGGCTTGTCCGCTAT-TTTT

>MK540419 Human gammaherpesvirus 4 isolate NPCT062

AGGACAGCCGTTGCCCTAGTGGTTTCGGACACACCGCCAACGCTCAGTGCGGTGCTACCGACCCGAGTTCAAGTCCCGGGGGAGGAGAAGAGAGGCTTCCCG-CCTAGAGCATTTGCAAGTCAGGATTCTCTAATCCCTCTGGGAGAAGGGTATTCGGCTTGTCCGCTAT-TTTT

>MK973062 Human gammaherpesvirus 4 strain rMSHJ

AGGACAGCCGTTGCCCTAGTGGTTTCGGACACACCGCCAACGCTCAGTGCGGTGCTACCGACCCGAGGTCAAGTCCCGGGGGAGGAGAAGAGAGGCTTCCCG-CCTAGAGCATTTGCAAGTCAGGATTCTCTAATCCCTCTGGGAGAAGGGTATTCGGCTTGTCCGCTGT-TTTT

>MN921212 Human gammaherpesvirus 4 isolate OIF-75 EBER snRNA gene, complete sequence

AGGACAGCCGTTGCCCTAGTGGTTTCGGACACACCGCCAACGCTCAGTGCGGTGCTACCGACCCGAGGTCAAGTCCCGGGGGAGGAGAAGAGAGGCTTCCCG-CCTAGAGCATTTGCAAGTCAGGATTCTGTAATCCCTCTGGGAGAAGGGTATTCGGCTTGTCCGCTAT-TTTT

>MN921214 Human gammaherpesvirus 4 isolate OSCC-84 EBER snRNA gene, complete sequence

AGGACAGCCGTTGCCCTAGTGGTTTCGGACACACCGCCAACGCTCAGTGCGGTGCTACCGACCCGAGGTCAAGTCCCGGGGGAGGAGAAGAGAGGCTTCCCG-CCTAGAGCATTTGCAAGTCAGGATTCTGTAATCCCTCTGGGAGAAGGGTATTCGGCTTGTCCGCTAT-TTTT

>MN921221 Human gammaherpesvirus 4 isolate OLP-35 EBER snRNA gene, complete sequence

AGGACAGCCGTTGCCCTAGTGGTTTCGGACACACCGCCAACGCTCAGTGCGGTGCTACCGACCCGAGGTCAAGTCCCGGGGGAGGAGAAGAGAGGCTTCCCG-CCTAGAGCATTTGCAAGTCAGGATTCTGTAATCCCTCTGGGAGAAGGGTATTCGGCTTGTCCGCTAT-TTTT

>MT648642 Human gammaherpesvirus 4 isolate Necker-1, partial genome

AGGACAGCCGTTGCCCTAGTGGTTTCGGACACACCGCCAACGCTCAGTGCGGTGCTACCGACCCGAGGTCAAGTCCCGGGGGAGGAGAAGAGAGGCTTCCCG-CCTAGAGCATTTGCAAGTCAGGATTCTCTAATCCCTCTGGGAGAAGGGTATTCGGCTTGTCCGCTGT-TTTT

>MT648643 Human gammaherpesvirus 4 isolate Necker-2, partial genome

AGGACAGCCGTTGCCCTAGTGGTTTCGGACACACCGCCAACGCTCAGTGCGGTGCTACCGACCCGAGGTCAAGTCCCGGGGGAGGAGAAGAGAGGCTTCCCG-CCTAGAGCATTTGCAAGTCAGGATTCTCTAATCCCTCTGGGAGAAGGGTATTCGGCTTGTCCGCTGT-TTTT

>MT648644 Human gammaherpesvirus 4 isolate Necker-3, partial genome

AGGACAGCCGTTGCCCTAGTGGTTTCGGACACACCGCCAACGCTCAGTGCGGTGCTACCGACCCGAGGTCAAGTCCCGGGGGAGGAGAAGAGAGGCTTCCCG-CCTAGAGCATTTGCAAGTCAGGATTCTCTAATCCCTCTGGGAGAAGGGTATTCGGCTTGTCCGCTGT-TTTT

>MT648645 Human gammaherpesvirus 4 isolate Necker-4, partial genome

AGGACAGCCGTTGCCCTAGTGGTTTCGGACACACCGCCAACGCTCAGTGCGGTGCTACCGACCCGAGGTCAAGTCCCGGGGGAGGAGAAGAGAGGCTTCCCG-CCTAGAGCATTTGCAAGTCAGGATTCTCTAATCCCTCTGGGAGAAGGGTATTCGGCTTGTCCGCTGT-TTTT

>MT648646 Human gammaherpesvirus 4 isolate Necker-5, partial genome

AGGACAGCCGTTGCCCTAGTGGTTTCGGACACACCGCCAACGCTCAGTGCGGTGCTACCGACCCGAGGTCAAGTCCCGGGGGAGGAGAAGAGAGGCTTCCCG-CCTAGAGCATTTGCAAGTCAGGATTCTCTAATCCCTCTGGGAGAAGGGTATTCGGCTTGTCCGCTGT-TTTT

>MT648647 Human gammaherpesvirus 4 isolate Necker-6, partial genome

AGGACAGCCGTTGCCCTAGTGGTTTCGGACACACCGCCAACGCTCAGTGCGGTGCTACCGACCCGAGGTCAAGTCCCGGGGGAGGAGAAGAGAGGCTTCCCG-CCTAGAGCATTTGCAAGTCAGGATTCTCTAATCCCTCTGGGAGAAGGGTATTCGGCTTGTCCGCTGT-TTTT

>MT648648 Human gammaherpesvirus 4 isolate Necker-7, partial genome

AGGACAGCCGTTGCCCTAGTGGTTTCGGACACACCGCCAACGCTCAGTGCGGTGCTACCGACCCGAGGTCAAGTCCCGGGGGAGGAGAAGAGAGGCTTCCCG-CCTAGAGCATTTGCAAGTCAGGATTCTCTAATCCCTCTGGGAGAAGGGTATTCGGCTTGTCCGCTGT-TTTT

>MT648649 Human gammaherpesvirus 4 isolate Necker-8, partial genome

AGGACAGCCGTTGCCCTAGTGGTTTCGGACACACCGCCAACGCTCAGTGCGGTGCTACCGACCCGAGGTCAAGTCCCGGGGGAGGAGAAGAGAGGCTTCCCG-CCTAGAGCATTTGCAAGTCAGGATTCTCTAATCCCTCTGGGAGAAGGGTATTCGGCTTGTCCGCTGT-TTTT

>MT648650 Human gammaherpesvirus 4 isolate Necker-9, partial genome

AGGACAGCCGTTGCCCTAGTGGTTTCGGACACACCGCCAACGCTCAGTGCGGTGCTACCGACCCGAGGTCAAGTCCCGGGGGAGGAGAAGAGAGGCTTCCCG-CCTAGAGCATTTGCAAGTCAGGATTCTCTAATCCCTCTGGGAGAAGGGTATTCGGCTTGTCCGCTGT-TTTT

>MT648651 Human gammaherpesvirus 4 isolate Necker-10, partial genome

AGGACAGCCGTTGCCCTAGTGGTTTCGGACACACCGCCAACGCTCAGTGCGGTGCTACCGACCCGAGGTCAAGTCCCGGGGGAGGAGAAGAGAGGCTTCCCG-CCTAGAGCATTTGCAAGTCAGGATTCTCTAATCCCTCTGGGAGAAGGGTATTCGGCTTGTCCGCTGT-TTTT

>MT648652 Human gammaherpesvirus 4 isolate Necker-11, partial genome

AGGACAGCCGTTGCCCTAGTGGTTTCGGACACACCGCCAACGCTCAGTGCGGTGCTACCGACCCGAGGTCAAGTCCCGGGGGAGGAGAAGAGAGGCTTCCCG-CCTAGAGCATTTGCAAGTCAGGATTCTCTAATCCCTCTGGGAGAAGGGTATTCGGCTTGTCCGCTGT-TTTT

>MT648653 Human gammaherpesvirus 4 isolate Necker-12, partial genome

AGGACAGCCGTTGCCCTAGTGGTTTCGGACACACCGCCAACGCTCAGTGCGGTGCTACCGACCCGAGGTCAAGTCCCGGGGGAGGAGAAGAGAGGCTTCCCG-CCTAGAGCATTTGCAAGTCAGGATTCTCTAATCCCTCTGGGAGAAGGGTATTCGGCTTGTCCGCTGT-TTTT

>MT648654 Human gammaherpesvirus 4 isolate Necker-13, partial genome

AGGACAGCCGTTGCCCTAGTGGTTTCGGACACACCGCCAACGCTCAGTGCGGTGCTACCGACCCGAGGTCAAGTCCCGGGGGAGGAGAAGAGAGGCTTCCCG-CCTAGAGCATTTGCAAGTCAGGATTCTCTAATCCCTCTGGGAGAAGGGTATTCGGCTTGTCCGCTGT-TTTT

>MT648655 Human gammaherpesvirus 4 isolate Necker-14, partial genome

AGGACAGCCGTTGCCCTAGTGGTTTCGGACACACCGCCAACGCTCAGTGCGGTGCTACCGACCCGAGGTCAAGTCCCGGGGGAGGAGAAGAGAGGCTTCCCG-CCTAGAGCATTTGCAAGTCAGGATTCTCTAATCCCTCTGGGAGAAGGGTATTCGGCTTGTCCGCTGT-TTTT

>MT648656 Human gammaherpesvirus 4 isolate Necker-15, partial genome

AGGACAGCCGTTGCCCTAGTGGTTTCGGACACACCGCCAACGCTCAGTGCGGTGCTACCGACCCGAGGTCAAGTCCCGGGGGAGGAGAAGAGAGGCTTCCCG-CCTAGAGCATTTGCAAGTCAGGATTCTCTAATCCCTCTGGGAGAAGGGTATTCGGCTTGTCCGCTGT-TTTT

>MT648657 Human gammaherpesvirus 4 isolate Necker-16, partial genome

AGGACAGCCGTTGCCCTAGTGGTTTCGGACACACCGCCAACGCTCAGTGCGGTGCTACCGACCCGAGGTCAAGTCCCGGGGGAGGAGAAGAGAGGCTTCCCG-CCTAGAGCATTTGCAAGTCAGGATTCTCTAATCCCTCTGGGAGAAGGGTATTCGGCTTGTCCGCTGT-TTTT

>MT648659 Human gammaherpesvirus 4 isolate Necker-18, partial genome

AGGACAGCCGTTGCCCTAGTGGTTTCGGACACACCGCCAACGCTCAGTGCGGTGCTACCGACCCGAGGTCAAGTCCCGGGGGAGGAGAAGAGAGGCTTCCCG-CCTAGAGCATTTGCAAGTCAGGATTCTCTAATCCCTCTGGGAGAAGGGTATTCGGCTTGTCCGCTGT-TTTT

>MT648660 Human gammaherpesvirus 4 isolate Necker-19, partial genome

AGGACAGCCGTTGCCCTAGTGGTTTCGGACACACCGCCAACGCTCAGTGCGGTGCTACCGACCCGAGGTCAAGTCCCGGGGGAGGAGAAGAGAGGCTTCCCG-CCTAGAGCATTTGCAAGTCAGGATTCTCTAATCCCTCTGGGAGAAGGGTATTCGGCTTGTCCGCTGT-TTTT

>MT648661 Human gammaherpesvirus 4 isolate Necker-20, partial genome

AGGACAGCCGTTGCCCTAGTGGTTTCGGACACACCGCCAACGCTCAGTGCGGTGCTACCGACCCGAGGTCAAGTCCCGGGGGAGGAGAAGAGAGGCTTCCCG-CCTAGAGCATTTGCAAGTCAGGATTCTCTAATCCCTCTGGGAGAAGGGTATTCGGCTTGTCCGCTGT-TTTT

>GU205107 Human herpesvirus 4 EBER-2, partial sequence

AGGACAGCCGTTGCCCTAGTGGTTTCGGACACACCGCCAACGCTCAGTGCGGTGCTACCGACCCGAGGTCAAGTCCCGGGGGAGGAGAAGAGAGGCTTCCCG-CCTAGAGCATTTGCAAGTCAGGATTCTCTAATCCCTCTGGGAGAAGGGTATTCGGCTTGTCCGCTA------

>AP015016 Human herpesvirus 4 DNA, complete genome, strain: YCCEL1

AGGACAGCCGTTGCCCTAGTGGTTTAGGACACACCGCCAACGCTCAGTGCGGTGCTACCGACCCGAGGTCAAGTCCCGGGGGAGGAGAAGAGAGGCTTCCCG-CCTAGAGCATTTGCAAGTCAGGATTCTCTAATCCCTCTGGGAGAAGGGTATTCGGCTTGTCCGCTGT-TTTT

>AP019016 Human gammaherpesvirus 4 SNT15_cell DNA, nearly complete genome

AGGACAGCCGTTGCCCTAGTGGTTTCGGACACACCGCCAACGCTCAGTGCGGTGCTACCGACCCGAGGTCGAGTCCCGGGGGAGGAGAAGAGAGGCTTCCCG-CCTAGAGCATTTGCAAGTCAGGATTCTCTAATCCCTCTGGGAGAAGGGTATTCGGCTTGTCCGCTGT-TTTT

>AP019044 Human gammaherpesvirus 4 UPN112_PBMC DNA, nearly complete genome

AGGACAGCCGTTGCCCTAGTGGTTTCGCACACACCGCCAACGCTCAGTGCGGTGCTACCGACCCGAGGTCAAGTCCCGGGGGAGGAGAAGAGAGGCTTCCCG-CCTAGAGCATTTGCAAGTCAGGATTCTCTAATCCCTCTGGGAGAAGGGTATTCGGCTTGTCCGCTGT-TTTT

>AP019050 Human gammaherpesvirus 4 UPN12_PBMC DNA, nearly complete genome

AGGACAGCCGTTGCCCTAGTGGTTTCCGACACACCGCCAACGCTCAGTGCGGTGCTACCGACCCGAGGTCAAGTCCCGGGGGAGGAGAAGAGAGGCTTCCCG-CCTAGAGCATTTGCAAGTCAGGATTCTCTAATCCCTCTGGGAGAAGGGTATTCGGCTTGTCCGCTGT-TTTT

>AP019052 Human gammaherpesvirus 4 UPN1202_PBMC DNA, nearly complete genome

AGGACAGCCGTTGCCCTAGTGGTTTCGGACACACCGCCAAAGCTCAGTGCGGTGCTACCGACCCGAGGTCAAGTCCCGGGGGAGGAGAAGAGAGGCTTCCCG-CCTAGAGCATTTGCAAGTCAGGATTCTCTAATCCCTCTGGGAGAAGGGTATTCGGCTTGTCCGCTGT-TTTT

>AP019078 Human gammaherpesvirus 4 UPN1758_PBMC DNA, nearly complete genome

AGGACAGCCGTTGCCCTAGTGGTTTCGGACACACCGCCAACGCTCAGTGCGGTGCTACCGACCCGAGGTCAAGTCCCGGGGGAGGAGAAGAGAGGCTTCCCG-CCTAGAGCATTTGCAAGTCAGGATTCTCTAATCCCTCTGGGAAAAGGGTATTCGGCTTGTCCGCTGT-TTTT

>AP019103 Human gammaherpesvirus 4 UPN258_PBMC DNA, nearly complete genome

AGGACAGCCGTTGCCCTAGTGGTTTCGGACACACCGCCAACGCTCAGTGCGGTGCTAGCGACCCGAGGTCAAGTCCCGGGGGAGGAGAAGAGAGGCTTCCCG-CCTAGAGCATTTGCAAGTCAGGATTCTCTAATCCCTCTGGGAGAAGGGTATTCGGCTTGTCCGCTGT-TTTT

>AP019106 Human gammaherpesvirus 4 UPN260_PBMC DNA, nearly complete genome

AGGACAGCCGTTGCCCTAGTGGTTTCGGACACACCGTCAACGCTCAGTGCGGTGCTACCGACCCGAGGTCAAGTCCCGGGGGAGGAGAAGAGAGGCTTCCCG-CCTAGAGCATTTGCAAGTCAGGATTCTCTAATCCCTCTGGGAGAAGGGTATTCGGCTTGTCCGCTGT-TTTT

>AP019114 Human gammaherpesvirus 4 UPN267_PBMC DNA, complete genome

AGGACAGCCGTTGCCCTAGTGGTTTCGGACACACCGCCAACGCTCAGTGCGGTGCTACCGACCCGAGTTCAAGTCCCGGGGGAGGAGAAGAGAGGCTTCCCG-CCTAGAGCATTTGCAAGTCAGGATTCTCTAATCCCTCTGGGAGAAGGGTATTCGGCTTGTCCGCTGT-TTTT

>AP019128 Human gammaherpesvirus 4 UPN326_PBMC DNA, nearly complete genome

AGGACAGCCGTTGCCCTAGTGGTTTCGCACACACCGCCAACGCTCAGTGCGGTGCTACCGACCCGAGGTCAAGTCCCGGGGGAGGAGAAGAGAGGCTTCCCG-CCTAGAGCATTTGCAAGTCAGGATTCTCTAATCCCTCTGGGAGAAGGGTATTCGGCTTGTCCGCTGT-TTTT

>AP019129 Human gammaherpesvirus 4 UPN3300_PBMC DNA, nearly complete genome

AGGACAGCCGTTGCCCTAGTGGTTTCGGACACACCGCCAACGCTCAGTGCGGTGCTACCGACCCGAGTTCAAGTCCCGGGGGAGGAGAAGAGAGGCTTCCCG-CCTAGAGCATTTGCAAGTCAGGATTCTCTAATCCCTCTGGGAGAAGGGTATTCGGCTTGTCCGCTGT-TTTT

>AP019159 Human gammaherpesvirus 4 UPN486_PBMC DNA, nearly complete genome

AGGACAGCCGTTGCCCTAGTGGTTTCGTACACACCGCCAACGCTCAGTGCGGTGCTACCGACCCGAGGTCAAGTCCCGGGGGAGGAGAAGAGAGGCTTCCCG-CCTAGAGCATTTGCAAGTCAGGATTCTCTAATCCCTCTGGGAGAAGGGTATTCGGCTTGTCCGCTGT-TTTT

>AP019172 Human gammaherpesvirus 4 UPN5575_PBMC DNA, nearly complete genome

AGGACAGCCGTTGCCCTAGTGGTTTCGGACACACCGCCAACGCTCAGTGCGGTGCTACCGACCCGAGATCAAGTCCCGGGGGAGGAGAAGAGAGGCTTCCCG-CCTAGAGCATTTGCAAGTCAGGATTCTCTAATCCCTCTGGGAGAAGGGTATTCGGCTTGTCCGCTGT-TTTT

>AP019178 Human gammaherpesvirus 4 UPN573_PBMC DNA, nearly complete genome

AGGACAGCCGTTGCCCTAGTGGTTTCGGACACACCGCCAACGCTCAGTGCGGTGCTAACGACCCGAGGTCAAGTCCCGGGGGAGGAGAAGAGAGGCTTCCCG-CCTAGAGCATTTGCAAGTCAGGATTCTCTAATCCCTCTGGGAGAAGGGTATTCGGCTTGTCCGCTGT-TTTT

>AP019182 Human gammaherpesvirus 4 UPN88_CD56+-1 DNA, nearly complete genome

AGGACAGCCGTTGCCCTAGTGGTTTCGCACACACCGCCAACGCTCAGTGCGGTGCTACCGACCCGAGGTCAAGTCCCGGGGGAGGAGAAGAGAGGCTTCCCG-CCTAGAGCATTTGCAAGTCAGGATTCTCTAATCCCTCTGGGAGAAGGGTATTCGGCTTGTCCGCTGT-TTTT

>AP019183 Human gammaherpesvirus 4 UPN88_CD56+-3 DNA, nearly complete genome

AGGACAGCCGTTGCCCTAGTGGTTTCGCACACACCGCCAACGCTCAGTGCGGTGCTACCGACCCGAGGTCAAGTCCCGGGGGAGGAGAAGAGAGGCTTCCCG-CCTAGAGCATTTGCAAGTCAGGATTCTCTAATCCCTCTGGGAGAAGGGTATTCGGCTTGTCCGCTGT-TTTT

>EF187848 Human herpesvirus 4 isolate SNU-538 EBER-1 and EBER-2 genes, complete sequence

AGGACAGCCGTTGCCCTAGTGGTTTCGGACACACCGCCAACGCTCAGTGCGGTGCTACCGACCCGAGATCAAGTCCCGGGGGAGGAGAAGAGAGGCTTCCCG-CCTAGAGCATTTGCAAGTCAGGATTCTCTAATCCCTCTGGGAGAAGGGTATTCGGCTTGTCCGCTGT-TTTT

>KP195435 Human herpesvirus 4 isolate SDNPC1 EBER1 and EBER2 genes, complete sequence

AGGACAGCCGTTGCCCTAGTGGTTTCGGACACACCGCCAACGCTCAGTGCGGTGCTACCGACCCGAGGTCGAGTCCCGGGGGAGGAGAAGAGAGGCTTCCCG-CCTAGAGCATTTGCAAGTCAGGATTCTCTAATCCCTCTGGGAGAAGGGTATTCGGCTTGTCCGCTGT-TTTT

>KP195446 Human herpesvirus 4 isolate SDNPC17 EBER1 and EBER2 genes, complete sequence

AGGACAGCCGTTGCCCTAGTGGTTTCGGACACACCGCCAACGCTCAGTGCGGTGCTACCGACCCGAGTTCAAGTCCCGGGGGAGGAGAAGAGAGGCTTCCCG-CCTAGAGCATTTGCAAGTCAGGATTCTCTAATCCCTCTGGGAGAAGGGTATTCGGCTTGTCCGCTGT-TTTT

>KP195458 Human herpesvirus 4 isolate SDNPC34 EBER1 and EBER2 genes, complete sequence

AGGACAGCCGTTGCCCTAGTGGTTTCGGACACACCGCCAACGCTCAGTGCGGTGCTACCGACCCGAGGTCAAGTCCCGGGGGAGGAGAAGAGAGGCTTCCCG-CCTAGAGCATTTGCAAGTCAGAATTCTCTAATCCCTCTGGGAGAAGGGTATTCGGCTTGTCCGCTGT-TTTT

>KP195461 Human herpesvirus 4 isolate SDNPC37 EBER1 and EBER2 genes, complete sequence

AGGACAGCCGTTGCCCTAGTGGTTTCGGACACACCGCCAACGCTCAGTGCGGTGCTACCGACCCGAGATCAAGTCCCGGGGGAGGAGAAGAGAGGCTTCCCG-CCTAGAGCATTTGCAAGTCAGGATTCTCTAATCCCTCTGGGAGAAGGGTATTCGGCTTGTCCGCTGT-TTTT

>KP195570 Human herpesvirus 4 isolate GDTW64 EBER1 and EBER2 genes, complete sequence

AGGACAGCCGTTGTCCTAGTGGTTTCGGACACACCGCCAACGCTCAGTGCGGTGCTACCGACCCGAGGTCAAGTCCCGGGGGAGGAGAAGAGAGGCTTCCCG-CCTAGAGCATTTGCAAGTCAGGATTCTCTAATCCCTCTGGGAGAAGGGTATTCGGCTTGTCCGCTGT-TTTT

>KP195599 Human herpesvirus 4 isolate GDTW127 EBER1 and EBER2 genes, complete sequence

AGGACAGCCGTTGCCCTAGTGGTTTCGGACACACCGCCAACGCTCAGTGCGGTGCTACCGACCCGAGGTCAAGTCCCAGGGGAGGAGAAGAGAGGCTTCCCG-CCTAGAGCATTTGCAAGTCAGGATTCTCTAATCCCTCTGGGAGAAGGGTATTCGGCTTGTCCGCTGT-TTTT

>KP195618 Human herpesvirus 4 isolate SDTW63 EBER1 and EBER2 genes, complete sequence

AGGACAGCCGTTGGCCTAGTGGTTTCGGACACACCGCCAACGCTCAGTGCGGTGCTACCGACCCGAGGTCAAGTCCCGGGGGAGGAGAAGAGAGGCTTCCCG-CCTAGAGCATTTGCAAGTCAGGATTCTCTAATCCCTCTGGGAGAAGGGTATTCGGCTTGTCCGCTGT-TTTT

>KP195666 Human herpesvirus 4 isolate SDTW188 EBER1 and EBER2 genes, complete sequence

AGGACAGCCGTTGCCCTAGTGGTTTCGGACACACCGCCAACTCTCAGTGCGGTGCTACCGACCCGAGGTCAAGTCCCGGGGGAGGAGAAGAGAGGCTTCCCG-CCTAGAGCATTTGCAAGTCAGGATTCTCTAATCCCTCTGGGAGAAGGGTATTCGGCTTGTCCGCTGT-TTTT

>KP195670 Human herpesvirus 4 isolate SDTW241 EBER1 and EBER2 genes, complete sequence

AGGACAGCCGTTGGCCTAGTGGTTTCGGACACACCGCCAACGCTCAGTGCGGTGCTACCGACCCGAGGTCAAGTCCCGGGGGAGGAGAAGAGAGGCTTCCCG-CCTAGAGCATTTGCAAGTCAGGATTCTCTAATCCCTCTGGGAGAAGGGTATTCGGCTTGTCCGCTGT-TTTT

>KT273949 Human gammaherpesvirus 4 isolate EBVaGC9

AGGACAGCCGTTGCCCTAGTGGTTTCGGACACACCGCCAACGCTCAGTGCGGTGCTACCGACCCGAGTTCAAGTCCCGGGGGAGGAGAAGAGAGGCTTCCCG-CCTAGAGCATTTGCAAGTCAGGATTCTCTAATCCCTCTGGGAGAAGGGTATTCGGCTTGTCCGCTGT-TTTT

>KX125053 Human gammaherpesvirus 4 isolate YCCEL1

AGGACAGCCGTTGCCCTAGTGGTTTAGGACACACCGCCAACGCTCAGTGCGGTGCTACCGACCCGAGGTCAAGTCCCGGGGGAGGAGAAGAGAGGCTTCCCG-CCTAGAGCATTTGCAAGTCAGGATTCTCTAATCCCTCTGGGAGAAGGGTATTCGGCTTGTCCGCTGT-TTTT

>LN827526 Human herpesvirus 4 genome assembly BL37, segment : I

AGGACAGCCGTTGCCCTAGTGGTTTCGGACACACCGCCAACGCTCAGTGCGGTGCTACCGACCCGAGATCAAGTCCCGGGGGAGGAGAAGAGAGGCTTCCCG-CCTAGAGCATTTGCAAGTCAGGATTCTCTAATCCCTCTGGGAGAAGGGTATTCGGCTTGTCCGCTGT-TTTT

>LN827527 Human herpesvirus 4 genome assembly M-ABA, segment : I

AGGACAGCCGTTGCCCTAGTGGTTTCGGACACACCGCCAACGCTCAGTGCGGTGCTACCGACCCGAGATCAAGTCCCGGGGGAGGAGAAGAGAGGCTTCCCG-CCTAGAGCATTTGCAAGTCAGGATTCTCTAATCCCTCTGGGAGAAGGGTATTCGGCTTGTCCGCTGT-TTTT

>LN827561 Human herpesvirus 4 genome assembly YCCEL1, segment : I

AGGACAGCCGTTGCCCTAGTGGTTTAGGACACACCGCCAACGCTCAGTGCGGTGCTACCGACCCGAGGTCAAGTCCCGGGGGAGGAGAAGAGAGGCTTCCCG-CCTAGAGCATTTGCAAGTCAGGATTCTCTAATCCCTCTGGGAGAAGGGTATTCGGCTTGTCCGCTGT-TTTT

>LN827583 Human herpesvirus 4 genome assembly sLCL-IM1.17, segment : I

AGGACAGCCGTTGCCCTAGTGGTTTCGGACACACCGCCAACGCTCAGTGCGGTGCTACCGACTCGAGGTCAAGTCCCGGGGGAGGAGAAGAGAGGCTTCCCG-CCTAGAGCATTTGCAAGTCAGGATTCTCTAATCCCTCTGGGAGAAGGGTATTCGGCTTGTCCGCTGT-TTTT

>LR812996 Human gammaherpesvirus 4 isolate HC-0016 genome assembly, chromosome: EBV

AGGACAGCCGTTGCCCTAGTGGTTTCGGACACACCGCCAACGCTCAGTGCGGTGCTACCGACCCGAGGTCAAGTCCCGGGGGAGGAGAAGAGGGGCTTCCCG-CCTAGAGCATTTGCAAGTCAGGATTCTCTAATCCCTCTGGGAGAAGGGTATTCGGCTTGTCCGCTGT-TTTT

>LR813043 Human gammaherpesvirus 4 isolate eBL-Tumor-0029 genome assembly, chromosome: EBV

AGGACAGCCGTTGCCCTAGTGGTTTCGGACACACCGCCAACGCTCAGTGCGGTGCTACCGACCCGAGATCAAGTCCCGGGGGAGGAGAAGAGAGGCTTCCCG-CCTAGAGCATTTGCAAGTCAGGATTCTCTAATCCCTCTGGGAGAAGGGTATTCGGCTTGTCCGCTGT-TTTT

>LS992247 Human gammaherpesvirus 4 isolate Human herpesvirus 4 genome assembly, chromosome: I

AGGACAGCCGTTGCCCTAGTGGTTTCGGACACACCGCCAACGCTCAGTGCGGTGCTACCGACCCGAGATCAAGTCCCGGGGGAGGAGAAGAGAGGCTTCCCG-CCTAGAGCATTTGCAAGTCAGGATTCTCTAATCCCTCTGGGAGAAGGGTATTCGGCTTGTCCGCTGT-TTTT

>LS992264 Human gammaherpesvirus 4 isolate Human herpesvirus 4 genome assembly, chromosome: I

AGGACAGCCGTTGCCCTAGTGGTTTCGGACACACCGCCAACGCTCAGTGCGGTGCTACCGACCCGAGATCAAGTCCCGGGGGAGGAGAAGAGAGGCTTCCCG-CCTAGAGCATTTGCAAGTCAGGATTCTCTAATCCCTCTGGGAGAAGGGTATTCGGCTTGTCCGCTGT-TTTT

>MG021306 Human gammaherpesvirus 4 isolate YCCEL1-GC2

AGGACAGCCGTTGCCCTAGTGGTTTAGGACACACCGCCAACGCTCAGTGCGGTGCTACCGACCCGAGGTCAAGTCCCGGGGGAGGAGAAGAGAGGCTTCCCG-CCTAGAGCATTTGCAAGTCAGGATTCTCTAATCCCTCTGGGAGAAGGGTATTCGGCTTGTCCGCTGT-TTTT

>MG298824 Human gammaherpesvirus 4 isolate AH_Saliva_8471

AGGACAGCCGTTGCCCTAGTGGTTTCGGACACACCGCCAACGCTCAGTGCGGTGCTACCGACCCGAGGTCAAGTCCCGGGGGAGGAGAAGAGAGGCTTCCCG-CCTAGAGCATTTGCAAGTCAGGATTCTCTAATCCCTCTGGGAGAAGGGTATTAGGCTTGTCCGCTGT-TTTT

>MG298832 Human gammaherpesvirus 4 isolate GK_BL18

AGGACAGCCGTTGCCCTAGTGGTTTCGGACACACCGCCAACGCTCAGTGCGGTGCTACCGACCCGAGATCAAGTCCCGGGGGAGGAGAAGAGAGGCTTCCCG-CCTAGAGCATTTGCAAGTCAGGATTCTCTAATCCCTCTGGGAGAAGGGTATTCGGCTTGTCCGCTGT-TTTT

>MG298850 Human gammaherpesvirus 4 isolate IMS_Saliva_193

AGGACAGCCGTTGCCCTAGTGGTTTCGGACACACAGCCAACGCTCAGTGCGGTGCTACCGACCCGAGGTCAAGTCCCGGGGGAGGAGAAGAGAGGCTTCCCG-CCTAGAGCATTTGCAAGTCAGGATTCTCTAATCCCTCTGGGAGAAGGGTATTCGGCTTGTCCGCTGT-TTTT

>MG298904 Human gammaherpesvirus 4 isolate JWBL17A

AGGACAGCCGTTGCCCTAGTGGTTTCGGACACACCGCCAACGCTCAGTGCGGTGCTACCGACCCGAGATCAAGTCCCGGGGGAGGAGAAGAGAGGCTTCCCG-CCTAGAGCATTTGCAAGTCAGGATTCTCTAATCCCTCTGGGAGAAGGGTATTCGGCTTGTCCGCTGT-TTTT

>MG298914 Human gammaherpesvirus 4 isolate RK_LCL_L3

AGGACAGCCGTTGCCCTAGTGGTTTCGGACACACCGCCAACGCTCAGTGCGGTGCTACCGACCCGAGGTCAAGTCCCGGGGGAGGAGAAGAGAGGCTTCCCG-CCTAGAGCATTTGCAAGTCAGGATTCTCTAATCCCTCTGGGAGAAGGGTATTCGGCTTGTTCGCTGT-TTTT

>MG298915 Human gammaherpesvirus 4 isolate RK_LCL_L4

AGGACAGCCGTTGCCCTAGTGGTTTCGGACACACCGCCAACGCTCAGTGCGGTGCTACCGACCCGAGGTCAAGTCCCGGGGGAGGAGAAGAGAGGCTTCCCG-CCTAGAGCATTTGCAAGTCAGGATTCTCTAATCCCTCTGGGTGAAGGGTATTCGGCTTGTCCGCTTT-TTTT

>MG298925 Human gammaherpesvirus 4 strain Epstein-Barr virus isolate JC_027_BWA

AGGACAGCCGTTGCCCTAGTGGTTTCGGACACACCGCCAACGCTCAGTGCGGTGCTACCGACCCGAGGTCGAGTCCCGGGGGAGGAGAAGAGAGGCTTCCCG-CCTAGAGCATTTGCAAGTCAGGATTCTCTAATCCCTCTGGGAGAAGGGTATTCGGCTTGTCCGCTGT-TTTT

>MH590387 Human gammaherpesvirus 4 isolate HKHD18

AGGACAGCCGTTGCCCTAGTGGTTTCGGACACACCGCCAACGCTCAGTGCGGTGCTGCCGACCCGAGGTCAAGTCCCGGGGGAGGAGAAGAGAGGCTTCCCG-CCTAGAGCATTTGCAAGTCAGGATTCTCTAATCCCTCTGGGAGAAGGGTATTCGGCTTGTCCGCTGT-TTTT

>MH590539 Human gammaherpesvirus 4 isolate HKNPC28

AGGACAGCCGTTGCCCTAGTGGTTTCGGACACACCGCCAACGCTCAGTGCGGTGCTACCGACCCGAGGTCGAGTCCCGGGGGAGGAGAAGAGAGGCTTCCCG-CCTAGAGCATTTGCAAGTCAGGATTCTCTAATCCCTCTGGGAGAAGGGTATTCGGCTTGTCCGCTGT-TTTT

>MH590554 Human gammaherpesvirus 4 isolate HKNPC43

AGGACAGCCGTTGCCCTAGTGGTTTCGGACACACCGCCAACGCTCAGTGCGGTGCTACCGACCCGAGGTCAAGTCCCGGGGGAGGAGAAGAGAGGCTTCCCGCCCTAGAGCATTTGCAAGTCAGGATTCTCTAATCCCTCTGGGAGAAGGGTATTCGGCTTGTCCGCTTT-TTTT

>MH883760 Human gammaherpesvirus 4 isolate ebv14

AGGACAGCCGTTGCCCTAGTGGTTTCGGACACACCGCCAACGCTCAGTGCGGTGCTACCGACCCGAGATCAAGTCCCGGGGGAGGAGAAGAGAGGCTTCCCG-CCTAGAGCATTTGCAAGTCAGGATTCTCTAATCCCTCTGGGAGAAGGGTATTCGGCTTGTCCGCTGT-TTTT

>MH883785 Human gammaherpesvirus 4 isolate P11-871

AGGACAGCCGTTGCCCTAGTGGTTTCGGACACACCGCCAACGCTCAGTGCGGTGCTACCGACCCGAGATCAAGTCCCGGGGGAGGAGAAGAGAGGCTTCCCG-CCTAGAGCATTTGCAAGTCAGGATTCTCTAATCCCTCTGGGAGAAGGGTATTCGGCTTGTCCGCTGT-TTTT

>MN921213 Human gammaherpesvirus 4 isolate OIF-82 EBER snRNA gene, complete sequence

AGGACAGCCGTTGCCCTAGTGGTTTCGGACACACCGCCAACGCTCAGTGCGGTGCTACCGACCCGAGGTCAAGTCCCGGGGGAGGAGAAGAGAGGCTTCCCG-CCTAGAGCATTTGCAAGTCAGGATTCTTTAATCCCTTTGGGAGAAGGGTATTCGGCTTGTCCGCTAT-TTTT

>MT648658 Human gammaherpesvirus 4 isolate Necker-17, partial genome

AGGACAGCCGTTGCCCTAGTGGTTTCGGACACACCGCCAACGCTCAGTGCGGTGCTACCGACCCGAGATCAAGTCCCGGGGGAGGAGAAGAGAGGCTTCCCG-CCTAGAGCATTTGCAAGTCAGGATTCTCTAATCCCTCTGGGAGAAGGGTATTCGGCTTGTCCGCTGT-TTTT

>MT648662 Human gammaherpesvirus 4 isolate Necker-21, partial genome

AGGACAGCCGTTGCCCTAGTGGTTTCGGACACACCGCCAACGCTCAGTGCGGTGCTACCGACCCGAGATCAAGTCCCGGGGGAGGAGAAGAGAGGCTTCCCG-CCTAGAGCATTTGCAAGTCAGGATTCTCTAATCCCTCTGGGAGAAGGGTATTCGGCTTGTCCGCTGT-TTTT

>J02068 epstein-barr virus b95-8 major rna gene region

AGGACAGCCGTTGCCCTAGTGGTTTCGGACACACCGCCAACGCTCAGTGCGGTGCTACCGACCCGAGGTCAAGTCCCGGGGAAGGAGAAGAGAGGCTTCCCG-CCTAGAGCATTTGCAAGTCAGGATTCTCTAAT-CCTCTGGGAGAAGGGTATTCGGCTTGTCCGCTAT-TTTT

>LS992262 Human gammaherpesvirus 4 isolate Human herpesvirus 4 genome assembly, chromosome: I

AGGACAGCCGTTGCCCTAGTGGTTTCGGACACACCGCCAACGCTCAGTGCGGTGCTANNGACCCGAGGTCAAGTCCCGGGGGAGGAGAAGAGAGGCTTCCCG-CCTAGAGCATTTGCAAGTCAGGATTCTCTAATCCCTCTGGGAGAAGGGTATTCGGCTTGTCCGCTGT-TTTT

>LS992269 Human gammaherpesvirus 4 isolate Human herpesvirus 4 genome assembly, chromosome: I

AGNACAGCCGTNGCCCTAGTGGTTTCGGACACACCGCCAACGCTCAGTGCGGTGCTACCGACCCGAGGTCAAGTCCCGGGGGAGGAGAAGAGAGGCTTCCCG-CCTAGAGCATTTGCAAGTCAGGATTCTCTAATCCCTCTGGGAGAAGGGTATTCGGCTTGTCCGCTGT-TTTT

>MH883778 Human gammaherpesvirus 4 isolate P4-2274, partial genome

AGGACAGCCGTTGCCCTAGTGGTTTCGGACACACCGCCAACGCTCAGTGCGGTGCTANNGACCCGAGGTCAAGTCCCGGGGGAGGAGAAGAGAGGCTTCCCG-CCTAGAGCATTTGCAAGTCAGGATTCTCTAATCCCTCTGGGAGAAGGGTATTCGGCTTGTCCGCTGT-TTTT

>MH883786 Human gammaherpesvirus 4 isolate P12-1026, partial genome

AGNACAGCCGTNGCCCTAGTGGTTTCGGACACACCGCCAACGCTCAGTGCGGTGCTACCGACCCGAGGTCAAGTCCCGGGGGAGGAGAAGAGAGGCTTCCCG-CCTAGAGCATTTGCAAGTCAGGATTCTCTAATCCCTCTGGGAGAAGGGTATTCGGCTTGTCCGCTGT-TTTT

>FN545286 Human herpesvirus 4 proviral DNA for partial EBER-1 and EBER-2 promoter, cell line NC37

AGGACAGCCGTTGCCCTAGTGGTTTCGGACACACCGCCAACGCTCAGTGCGGTGCTACCGACCCGAGGTCAAGTCCCGGGGGAGGAGAAGAGAGGCTTCCCG-CCTAGAGCATTTGCAAGTCAGGATTCTCTAATCCCTCTGGGAGAAGGGTATTCGGCTTGTCCG---------

>AP019027 Human gammaherpesvirus 4 UPN105_PBMC DNA, nearly complete genome

AGGACAGCCGTTGCCCTAGTGGTTTGAGACACACCGCCAACGCTCAGTGCGGTGCTACCGACCCGAGGTCAAGTCCCGGGGGAGGAGAAGAGAGGCTTCCCG-CCTAGAGCATTTGCAAGTCAGGATTCTCTAATCCCTCTGGGAGAAGGGTATTCGGCTTGTCCGCTGT-TTTT

>AP019028 Human gammaherpesvirus 4 UPN105_tumor DNA, complete genome

AGGACAGCCGTTGCCCTAGTGGTTTGAGACACACCGCCAACGCTCAGTGCGGTGCTACCGACCCGAGGTCAAGTCCCGGGGGAGGAGAAGAGAGGCTTCCCG-CCTAGAGCATTTGCAAGTCAGGATTCTCTAATCCCTCTGGGAGAAGGGTATTCGGCTTGTCCGCTGT-TTTT

>AP019041 Human gammaherpesvirus 4 UPN1111_PBMC DNA, nearly complete genome

AGGACAGCCGTTGCCCTAGTGGTTTCGGACACACCGCCAACGCTAAGTGCGGTGCTACCGACCCGAGTTCAAGTCCCGGGGGAGGAGAAGAGAGGCTTCCCG-CCTAGAGCATTTGCAAGTCAGGATTCTCTAATCCCTCTGGGAGAAGGGTATTCGGCTTGTCCGCTGT-TTTT

>AP019087 Human gammaherpesvirus 4 UPN1926_PBMC DNA, complete genome

AGGACAGCCGTTGCCCTAGTGGTTTCGGACACACCGCCAACGCTCAGTGCGGTGCGACCGACCCGAGATCAAGTCCCGGGGGAGGAGAAGAGAGGCTTCCCG-CCTAGAGCATTTGCAAGTCAGGATTCTCTAATCCCTCTGGGAGAAGGGTATTCGGCTTGTCCGCTGT-TTTT

>KP195440 Human herpesvirus 4 isolate SDNPC11 EBER1 and EBER2 genes, complete sequence

AGGACAGCCGTTGCCCTAGTGGTTTGGGACACACCGCCAACGCTCAGTGCGGTGCTACCGACCCGAGTTCAAGTCCCGGGGGAGGAGAAGAGAGGCTTCCCG-CCTAGAGCATTTGCAAGTCAGGATTCTCTAATCCCTCTGGGAGAAGGGTATTCGGCTTGTCCGCTGT-TTTT

>KP195441 Human herpesvirus 4 isolate SDNPC12 EBER1 and EBER2 genes, complete sequence

AGGACAGCCGTTGCCCTAGTGGTTTCGCATACACCGCCAACGCTCAGTGCGGTGCTACCGACCCGAGGTCAAGTCCCGGGGGAGGAGAAGAGAGGCTTCCCG-CCTAGAGCATTTGCAAGTCAGGATTCTCTAATCCCTCTGGGAGAAGGGTATTCGGCTTGTCCGCTGT-TTTT

>KP195611 Human herpesvirus 4 isolate SDTW18 EBER1 and EBER2 genes, complete sequence

AGGTCAGCCGTTGCCCTAGTGGTTTCGGACACACCGCCAACGCTCAGTGCGGTGCTACCGACCCGAGGTCAAGTCCCGGGGGAGGAGAAGAGAGGCTTCCCG-CCTAGAGCATTTGCAAGTCAGGATTCTCTAATCCCTCTGGGAGAAGGGTATTCGGCCTGTCCGCTGT-TTTT

>KR063343 Human gammaherpesvirus 4 strain CV-ARG

AGGACAGCCGTTGCCCTAGTGGTTTCGGACAGCCCGCCAACGCTCAGTGCGGTGCTACCGACCCGAGGTCAAGTCCCGGGGGAGGAGAAGAGAGGCTTCCCG-CCTAGAGCATTTGCAAGTCAGGATTCTCTAATCCCTCTGGGAGAAGGGTATTCGGCTTGTCCGCTGT-TTTT

>KR063344 Human gammaherpesvirus 4 strain RPF

AGGACAGCCGTTGCCCTAGTGGTTTCGGACAGCCCGCCAACGCTCAGTGCGGTGCTACCGACCCGAGGTCAAGTCCCGGGGGAGGAGAAGAGAGGCTTCCCG-CCTAGAGCATTTGCAAGTCAGGATTCTCTAATCCCTCTGGGAGAAGGGTATTCGGCTTGTCCGCTGT-TTTT

>LN827556 Human herpesvirus 4 genome assembly Cheptages, segment : I

AGGACAGCCGTTGCCCTAGTGGTTTCGGACACACCGCCAACGCTCAGTGCGGTGCTACCGACCCGAGGTCAAATCCCGGGGGAGGAGAAGAGAGGCTTCCCG-CCTAGAGCATTTGCAAGTCAGGATTCTCTAATCCCTCTGGGAGAAGGGTATTCGGCTTGTCCGTTGT-TTTT

>LN827582 Human herpesvirus 4 genome assembly sLCL-BL1.03, segment : I

AGGACAGCCGTTGCCCTAGTGGTTTCGGACACACCGCCAACGCTCAGTGCGGTGCTACCGACCCGAGGTCAAATCCCGGGGGAGGAGAAGAGAGGCTTCCCG-CCTAGAGCATTTGCAAGTCAGGATTCTCTAATCCCTCTGGGAGAAGGGTATTCGGCTTGTCCGTTGT-TTTT

>LR813072 Human gammaherpesvirus 4 isolate eBL-Plasma-0051 genome assembly, chromosome: EBV

AGGACAGCCGTTGCCCTAGTGGTTTCGGACACACCGCCAACGCTCAGTGCGGTGCTACCGACCCGAGGTCAAATCCCGGGGGAGGAGAAGAGAGGCTTCCCG-CCTAGAGCATTTGCAAGTCAGGATTCTCTAATCCCTCTGGGAGAAGGGTATTCGGCTTGTCCGTTGT-TTTT

>MG298828 Human gammaherpesvirus 4 isolate DF_Tonsil_T47

AGGACAGCCGTTGCCCTAGTGGTTTCGGACAGCCCGCCAACGCTCAGTGCGGTGCTACCGACCCGAGGTCAAGTCCCGGGGGAGGAGAAGAGAGGCTTCCCG-CCTAGAGCATTTGCAAGTCAGGATTCTCTAATCCCTCTGGGAGAAGGGTATTCGGCTTGTCCGCTGT-TTTT

>MG298829 Human gammaherpesvirus 4 isolate DF_Tonsil_T49

AGGACAGCCGTTGCCCTAGTGGTTTCGGACAGCCCGCCAACGCTCAGTGCGGTGCTACCGACCCGAGGTCAAGTCCCGGGGGAGGAGAAGAGAGGCTTCCCG-CCTAGAGCATTTGCAAGTCAGGATTCTCTAATCCCTCTGGGAGAAGGGTATTCGGCTTGTCCGCTGT-TTTT

>MN921220 Human gammaherpesvirus 4 isolate OLP-16 EBER snRNA gene, complete sequence

AGGACAGCCGTTGCCCTAGTGGTTTTGGACACACCGCCAACGCTCAGTGCGGTGCTACCGACCCGAGGTCAAGTCCCGGGGGAGGAGAAGAGAGGCTTCCCG-CCTAGAGCATTTGCAAGTCAGGATTCTTTAATCCCTTTGGGAGAAGGGTATTCGGCTTGTCCGCTAT-TTTT

>KP195596 Human herpesvirus 4 isolate GDTW122 EBER1 and EBER2 genes, complete sequence

AGGACAGCCGTTGCCCTAGTGGTTTCGGACACACCGCCAACGCTCAGTGCGGTGCTACCGACCCGAGGTCAAGTCCCAAGGGAGGAGAAGAGAGGCTTCCCG-CCTAGAGCATTTGCAAGTCAGGATTCTCTAATCCCTCTAGGAGAAGGGTATTCGGCTTGTCCGCTGT-TTTT

>LR813005 Human gammaherpesvirus 4 isolate HC-0022 genome assembly, chromosome: EBV

AGGACAGCCGTTGCCCTAGTGGTTTCGGACACACCGCCAACGCTCAGTGCGGTGCTACCGACCCGAGGTCAAGTCCCGGGGGAGGAGAAGAGAGGCTTCCCG-CCTAGAGCATTTGCAAGTCAGGATTCTCTAATNNNNCTGGGAGAAGGGTATTCGGCTTGTCCGCT-------

>LC137018 Human herpesvirus 4 DNA, nearly complete genome, strain: HNNPC1

AGGACAGCCGTTGCCCTAGTGGTTTCGGACACACCGCCAACGCGCTGTGCGGTGCTGCCGTCCCGAGGTCAAGTCCCGGGGGAGGAGAAGAGCGGCTTCCCG-CCTAGAGCATTTGCAAGTCAGGATTCTCTAATCCCTCTGGGAGAAGGGTATTCGGCTTGTCCGCTAT-TTTT

>MK540362 Human gammaherpesvirus 4 isolate NPCT004

AGGACAGCCGTTGCCCTAGTGGTTTCGGACACACCGCCAACGCGCTGTGCGGTGCTGCCGTCCCGAGGTCAAGTCCCGGGGGAGGAGAAGAGCGGCTTCCCG-CCTAGAGCATTTGCAAGTCAGGATTCTCTAATCCCTCTGGGAGAAGGGTATTCGGCTTGTCCGCTAT-TTTT

>AB828190 Human herpesvirus 4 DNA, complete genome, strain: 1 LGY-C666-1

AGGACAGCCGTTGCCCTAGTGGTTTCGGACACACCGCCAACGCGCTGTGCGGTGCTGCCGTCCCGAGGTCAAGTCCCGGGGGAGGAGAAGAGCGGCTTCCCG-CCTAGAGCATTTGCAAGTCAGGATTCTCTAATCCCTCTGGGAGAAGGGTATTCGGCTTGTCCGCTGT-TTTT

>AB850643 Human herpesvirus 4 DNA, complete genome, strain: HN1

AGGACAGCCGTTGCCCTAGTGGTTTCGGACACACCGCCAACGCGCTGTGCGGTGCTGCCGTCCCGAGGTCAAGTCCCGGGGGAGGAGAAGAGCGGCTTCCCG-CCTAGAGCATTTGCAAGTCAGGATTCTCTAATCCCTCTGGGAGAAGGGTATTCGGCTTGTCCGCTGT-TTTT

>AB850646 Human herpesvirus 4 DNA, complete genome, strain: HN11

AGGACAGCCGTTGCCCTAGTGGTTTCGGACACACCGCCAACGCGCTGTGCGGTGCTGCCGTCCCGAGGTCAAGTCCCGGGGGAGGAGAAGAGCGGCTTCCCG-CCTAGAGCATTTGCAAGTCAGGATTCTCTAATCCCTCTGGGAGAAGGGTATTCGGCTTGTCCGCTGT-TTTT

>AB850649 Human herpesvirus 4 DNA, complete genome, strain: HN4

AGGACAGCCGTTGCCCTAGTGGTTTCGGACACACCGCCAACGCGCTGTGCGGTGCTGCCGTCCCGAGGTCAAGTCCCGGGGGAGGAGAAGAGCGGCTTCCCG-CCTAGAGCATTTGCAAGTCAGGATTCTCTAATCCCTCTGGGAGAAGGGTATTCGGCTTGTCCGCTGT-TTTT

>AB850651 Human herpesvirus 4 DNA, complete genome, strain: HN14

AGGACAGCCGTTGCCCTAGTGGTTTCGGACACACCGCCAACGCGCTGTGCGGTGCTGCCGTCCCGAGGTCAAGTCCCGGGGGAGGAGAAGAGCGGCTTCCCG-CCTAGAGCATTTGCAAGTCAGGATTCTCTAATCCCTCTGGGAGAAGGGTATTCGGCTTGTCCGCTGT-TTTT

>AB850652 Human herpesvirus 4 DNA, complete genome, strain: HN5

AGGACAGCCGTTGCCCTAGTGGTTTCGGACACACCGCCAACGCGCTGTGCGGTGCTGCCGTCCCGAGGTCAAGTCCCGGGGGAGGAGAAGAGCGGCTTCCCG-CCTAGAGCATTTGCAAGTCAGGATTCTCTAATCCCTCTGGGAGAAGGGTATTCGGCTTGTCCGCTGT-TTTT

>AB850655 Human herpesvirus 4 DNA, complete genome, strain: HN7

AGGACAGCCGTTGCCCTAGTGGTTTCGGACACACCGCCAACGCGCTGTGCGGTGCTGCCGTCCCGAGGTCAAGTCCCGGGGGAGGAGAAGAGCGGCTTCCCG-CCTAGAGCATTTGCAAGTCAGGATTCTCTAATCCCTCTGGGAGAAGGGTATTCGGCTTGTCCGCTGT-TTTT

>AB850656 Human herpesvirus 4 DNA, complete genome, strain: HN16

AGGACAGCCGTTGCCCTAGTGGTTTCGGACACACCGCCAACGCGCTGTGCGGTGCTGCCGTCCCGAGGTCAAGTCCCGGGGGAGGAGAAGAGCGGCTTCCCG-CCTAGAGCATTTGCAAGTCAGGATTCTCTAATCCCTCTGGGAGAAGGGTATTCGGCTTGTCCGCTGT-TTTT

>AB850657 Human herpesvirus 4 DNA, complete genome, strain: HN8

AGGACAGCCGTTGCCCTAGTGGTTTCGGACACACCGCCAACGCGCTGTGCGGTGCTGCCGTCCCGAGGTCAAGTCCCGGGGGAGGAGAAGAGCGGCTTCCCG-CCTAGAGCATTTGCAAGTCAGGATTCTCTAATCCCTCTGGGAGAAGGGTATTCGGCTTGTCCGCTGT-TTTT

>AB850660 Human herpesvirus 4 DNA, complete genome, strain: HN18

AGGACAGCCGTTGCCCTAGTGGTTTCGGACACACCGCCAACGCGCTGTGCGGTGCTGCCGTCCCGAGGTCAAGTCCCGGGGGAGGAGAAGAGCGGCTTCCCG-CCTAGAGCATTTGCAAGTCAGGATTCTCTAATCCCTCTGGGAGAAGGGTATTCGGCTTGTCCGCTGT-TTTT

>AP019116 Human gammaherpesvirus 4 UPN2723_PBMC DNA, nearly complete genome

AGGACAGCCGTTGCCCTAGTGGTTTCGGACACACCGCCAACGCGCTGTGCGGTGCTGCCGTCCCGAGGTCAAGTCCCGGGGGAGGAGAAGAGCGGCTTCCCG-CCTAGAGCATTTGCAAGTCAGGATTCTCTAATCCCTCTGGGAGAAGGGTATTCGGCTTGTCCGCTGT-TTTT

>DQ883818 Human herpesvirus 4 isolate SNU-20 EBER-1 and EBER-2 genes, complete sequence

AGGACAGCCGTTGCCCTAGTGGTTTCGGACACACCGCCAACGCGCTGTGCGGTGCTGCCGTCCCGAGGTCAAGTCCCGGGGGAGGAGAAGAGCGGCTTCCCG-CCTAGAGCATTTGCAAGTCAGGATTCTCTAATCCCTCTGGGAGAAGGGTATTCGGCTTGTCCGCTGT-TTTT

>DQ883819 Human herpesvirus 4 isolate SNU-265 EBER-1 and EBER-2 genes, complete sequence

AGGACAGCCGTTGCCCTAGTGGTTTCGGACACACCGCCAACGCGCTGTGCGGTGCTGCCGTCCCGAGGTCAAGTCCCGGGGGAGGAGAAGAGCGGCTTCCCG-CCTAGAGCATTTGCAAGTCAGGATTCTCTAATCCCTCTGGGAGAAGGGTATTCGGCTTGTCCGCTGT-TTTT

>JQ009376 Human herpesvirus 4 strain HKNPC1, genome

AGGACAGCCGTTGCCCTAGTGGTTTCGGACACACCGCCAACGCGCTGTGCGGTGCTGCCGTCCCGAGGTCAAGTCCCGGGGGAGGAGAAGAGCGGCTTCCCG-CCTAGAGCATTTGCAAGTCAGGATTCTCTAATCCCTCTGGGAGAAGGGTATTCGGCTTGTCCGCTGT-TTTT

>KC617875 Human herpesvirus 4 strain C666-1, partial genome

AGGACAGCCGTTGCCCTAGTGGTTTCGGACACACCGCCAACGCGCTGTGCGGTGCTGCCGTCCCGAGGTCAAGTCCCGGGGGAGGAGAAGAGCGGCTTCCCG-CCTAGAGCATTTGCAAGTCAGGATTCTCTAATCCCTCTGGGAGAAGGGTATTCGGCTTGTCCGCTTT-TTTT

>KF373730 Human herpesvirus 4 strain M81, complete genome

AGGACAGCCGTTGCCCTAGTGGTTTCGGACACACCGCCAACGCGCTGTGCGGTGCTGCCGTCCCGAGGTCAAGTCCCGGGGGAGGAGAAGAGCGGCTTCCCG-CCTAGAGCATTTGCAAGTCAGGATTCTCTAATCCCTCTGGGAGAAGGGTATTCGGCTTGTCCGCTGT-TTTT

>KF992564 Human herpesvirus 4 isolate HKNPC2, partial genome

AGGACAGCCGTTGCCCTAGTGGTTTCGGACACACCGCCAACGCGCTGTGCGGTGCTGCCGTCCCGAGGTCAAGTCCCGGGGGAGGAGAAGAGCGGCTTCCCG-CCTAGAGCATTTGCAAGTCAGGATTCTCTAATCCCTCTGGGAGAAGGGTATTCGGCTTGTCCGCTGT-TTTT

>KF992565 Human herpesvirus 4 isolate HKNPC3, partial genome

AGGACAGCCGTTGCCCTAGTGGTTTCGGACACACCGCCAACGCGCTGTGCGGTGCTGCCGTCCCGAGGTCAAGTCCCGGGGGAGGAGAAGAGCGGCTTCCCG-CCTAGAGCATTTGCAAGTCAGGATTCTCTAATCCCTCTGGGAGAAGGGTATTCGGCTTGTCCGCTGT-TTTT

>KF992566 Human herpesvirus 4 isolate HKNPC4, partial genome

AGGACAGCCGTTGCCCTAGTGGTTTCGGACACACCGCCAACGCGCTGTGCGGTGCTGCCGTCCCGAGGTCAAGTCCCGGGGGAGGAGAAGAGCGGCTTCCCG-CCTAGAGCATTTGCAAGTCAGGATTCTCTAATCCCTCTGGGAGAAGGGTATTCGGCTTGTCCGCTGT-TTTT

>KF992567 Human herpesvirus 4 isolate HKNPC5, partial genome

AGGACAGCCGTTGCCCTAGTGGTTTCGGACACACCGCCAACGCGCTGTGCGGTGCTGCCGTCCCGAGGTCAAGTCCCGGGGGAGGAGAAGAGCGGCTTCCCG-CCTAGAGCATTTGCAAGTCAGGATTCTCTAATCCCTCTGGGAGAAGGGTATTCGGCTTGTCCGCTGT-TTTT

>KF992568 Human herpesvirus 4 isolate HKNPC6, partial genome

AGGACAGCCGTTGCCCTAGTGGTTTCGGACACACCGCCAACGCGCTGTGCGGTGCTGCCGTCCCGAGGTCAAGTCCCGGGGGAGGAGAAGAGCGGCTTCCCG-CCTAGAGCATTTGCAAGTCAGGATTCTCTAATCCCTCTGGGAGAAGGGTATTCGGCTTGTCCGCTGT-TTTT

>KF992569 Human herpesvirus 4 isolate HKNPC7, partial genome

AGGACAGCCGTTGCCCTAGTGGTTTCGGACACACCGCCAACGCGCTGTGCGGTGCTGCCGTCCCGAGGTCAAGTCCCGGGGGAGGAGAAGAGCGGCTTCCCG-CCTAGAGCATTTGCAAGTCAGGATTCTCTAATCCCTCTGGGAGAAGGGTATTCGGCTTGTCCGCTGT-TTTT

>KF992570 Human herpesvirus 4 isolate HKNPC8, partial genome

AGGACAGCCGTTGCCCTAGTGGTTTCGGACACACCGCCAACGCGCTGTGCGGTGCTGCCGTCCCGAGGTCAAGTCCCGGGGGAGGAGAAGAGCGGCTTCCCG-CCTAGAGCATTTGCAAGTCAGGATTCTCTAATCCCTCTGGGAGAAGGGTATTCGGCTTGTCCGCTGT-TTTT

>KF992571 Human herpesvirus 4 isolate HKNPC9, partial genome

AGGACAGCCGTTGCCCTAGTGGTTTCGGACACACCGCCAACGCGCTGTGCGGTGCTGCCGTCCCGAGGTCAAGTCCCGGGGGAGGAGAAGAGCGGCTTCCCG-CCTAGAGCATTTGCAAGTCAGGATTCTCTAATCCCTCTGGGAGAAGGGTATTCGGCTTGTCCGCTGT-TTTT

>KJ411974 Human herpesvirus 4 isolate C666-1, partial genome

AGGACAGCCGTTGCCCTAGTGGTTTCGGACACACCGCCAACGCGCTGTGCGGTGCTGCCGTCCCGAGGTCAAGTCCCGGGGGAGGAGAAGAGCGGCTTCCCG-CCTAGAGCATTTGCAAGTCAGGATTCTCTAATCCCTCTGGGAGAAGGGTATTCGGCTTGTCCGCTGT-TTTT

>KP195385 Human herpesvirus 4 isolate GDNPC1 EBER1 and EBER2 genes, complete sequence

AGGACAGCCGTTGCCCTAGTGGTTTCGGACACACCGCCAACGCGCTGTGCGGTGCTGCCGTCCCGAGGTCAAGTCCCGGGGGAGGAGAAGAGCGGCTTCCCG-CCTAGAGCATTTGCAAGTCAGGATTCTCTAATCCCTCTGGGAGAAGGGTATTCGGCTTGTCCGCTGT-TTTT

>KP195392 Human herpesvirus 4 isolate GDNPC9 EBER1 and EBER2 genes, complete sequence

AGGACAGCCGTTGCCCTAGTGGTTTCGGACACACCGCCAACGCGCTGTGCGGTGCTGCCGTCCCGAGGTCAAGTCCCGGGGGAGGAGAAGAGCGGCTTCCCG-CCTAGAGCATTTGCAAGTCAGGATTCTCTAATCCCTCTGGGAGAAGGGTATTCGGCTTGTCCGCTGT-TTTT

>KP195399 Human herpesvirus 4 isolate GDNPC18 EBER1 and EBER2 genes, complete sequence

AGGACAGCCGTTGCCCTAGTGGTTTCGGACACACCGCCAACGCGCTGTGCGGTGCTGCCGTCCCGAGGTCAAGTCCCGGGGGAGGAGAAGAGCGGCTTCCCG-CCTAGAGCATTTGCAAGTCAGGATTCTCTAATCCCTCTGGGAGAAGGGTATTCGGCTTGTCCGCTGT-TTTT

>KP195407 Human herpesvirus 4 isolate GDNPC26 EBER1 and EBER2 genes, complete sequence

AGGACAGCCGTTGCCCTAGTGGTTTCGGACACACCGCCAACGCGCTGTGCGGTGCTGCCGTCCCGAGGTCAAGTCCCGGGGGAGGAGAAGAGCGGCTTCCCG-CCTAGAGCATTTGCAAGTCAGGATTCTCTAATCCCTCTGGGAGAAGGGTATTCGGCTTGTCCGCTGT-TTTT

>KP195410 Human herpesvirus 4 isolate GDNPC30 EBER1 and EBER2 genes, complete sequence

AGGACAGCCGTTGCCCTAGTGGTTTCGGACACACCGCCAACGCGCTGTGCGGTGCTGCCGTCCCGAGGTCAAGTCCCGGGGGAGGAGAAGAGCGGCTTCCCG-CCTAGAGCATTTGCAAGTCAGGATTCTCTAATCCCTCTGGGAGAAGGGTATTCGGCTTGTCCGCTGT-TTTT

>KP195418 Human herpesvirus 4 isolate GDNPC44 EBER1 and EBER2 genes, complete sequence

AGGACAGCCGTTGCCCTAGTGGTTTCGGACACACCGCCAACGCGCTGTGCGGTGCTGCCGTCCCGAGGTCAAGTCCCGGGGGAGGAGAAGAGCGGCTTCCCG-CCTAGAGCATTTGCAAGTCAGGATTCTCTAATCCCTCTGGGAGAAGGGTATTCGGCTTGTCCGCTGT-TTTT

>KP195422 Human herpesvirus 4 isolate GDNPC50 EBER1 and EBER2 genes, complete sequence

AGGACAGCCGTTGCCCTAGTGGTTTCGGACACACCGCCAACGCGCTGTGCGGTGCTGCCGTCCCGAGGTCAAGTCCCGGGGGAGGAGAAGAGCGGCTTCCCG-CCTAGAGCATTTGCAAGTCAGGATTCTCTAATCCCTCTGGGAGAAGGGTATTCGGCTTGTCCGCTGT-TTTT

>KP195428 Human herpesvirus 4 isolate GDNPC56 EBER1 and EBER2 genes, complete sequence

AGGACAGCCGTTGCCCTAGTGGTTTCGGACACACCGCCAACGCGCTGTGCGGTGCTGCCGTCCCGAGGTCAAGTCCCGGGGGAGGAGAAGAGCGGCTTCCCG-CCTAGAGCATTTGCAAGTCAGGATTCTCTAATCCCTCTGGGAGAAGGGTATTCGGCTTGTCCGCTGT-TTTT

>KP195438 Human herpesvirus 4 isolate SDNPC7 EBER1 and EBER2 genes, complete sequence

AGGACAGCCGTTGCCCTAGTGGTTTCGGACACACCGCCAACGCGCTGTGCGGTGCTGCCGTCCCGAGGTCAAGTCCCGGGGGAGGAGAAGAGCGGCTTCCCG-CCTAGAGCATTTGCAAGTCAGGATTCTCTAATCCCTCTGGGAGAAGGGTATTCGGCTTGTCCGCTGT-TTTT

>KP195450 Human herpesvirus 4 isolate SDNPC22 EBER1 and EBER2 genes, complete sequence

AGGACAGCCGTTGCCCTAGTGGTTTCGGACACACCGCCAACGCGCTGTGCGGTGCTGCCGTCCCGAGGTCAAGTCCCGGGGGAGGAGAAGAGCGGCTTCCCG-CCTAGAGCATTTGCAAGTCAGGATTCTCTAATCCCTCTGGGAGAAGGGTATTCGGCTTGTCCGCTGT-TTTT

>KP195459 Human herpesvirus 4 isolate SDNPC35 EBER1 and EBER2 genes, complete sequence

AGGACAGCCGTTGCCCTAGTGGTTTCGGACACACCGCCAACGCGCTGTGCGGTGCTGCCGTCCCGAGGTCAAGTCCCGGGGGAGGAGAAGAGCGGCTTCCCG-CCTAGAGCATTTGCAAGTCAGGATTCTCTAATCCCTCTGGGAGAAGGGTATTCGGCTTGTCCGCTGT-TTTT

>KP195514 Human herpesvirus 4 isolate SDNPC124 EBER1 and EBER2 genes, complete sequence

AGGACAGCCGTTGCCCTAGTGGTTTCGGACACACCGCCAACGCGCTGTGCGGTGCTGCCGTCCCGAGGTCAAGTCCCGGGGGAGGAGAAGAGCGGCTTCCCG-CCTAGAGCATTTGCAAGTCAGGATTCTCTAATCCCTCTGGGAGAAGGGTATTCGGCTTGTCCGCTGT-TTTT

>KP195518 Human herpesvirus 4 isolate SDNPC130 EBER1 and EBER2 genes, complete sequence

AGGACAGCCGTTGCCCTAGTGGTTTCGGACACACCGCCAACGCGCTGTGCGGTGCTGCCGTCCCGAGGTCAAGTCCCGGGGGAGGAGAAGAGCGGCTTCCCG-CCTAGAGCATTTGCAAGTCAGGATTCTCTAATCCCTCTGGGAGAAGGGTATTCGGCTTGTCCGCTGT-TTTT

>KP195576 Human herpesvirus 4 isolate GDTW70 EBER1 and EBER2 genes, complete sequence

AGGACAGCCGTTGCCCTAGTGGTTTCGGACACACCGCCAACGCGCTGTGCGGTGCTGCCGTCCCGAGGTCAAGTCCCGGGGGAGGAGAAGAGCGGCTTCCCG-CCTAGAGCATTTGCAAGTCAGGATTCTCTAATCCCTCTGGGAGAAGGGTATTCGGCTTGTCCGCTGT-TTTT

>KP968260 Human herpesvirus 4 strain VGO, partial genome

AGGACAGCCGTTGCCCTAGTGGTTTCGGACACACCGCCAACGCGCTGTGCGGTGCTGCCGTCCCGAGGTCAAGTCCCGGGGGAGGAGAAGAGCGGCTTCCCG-CCTAGAGCATTTGCAAGTCAGGATTCTCTAATCCCTCTGGGAGAAGGGTATTCGGCTTGTCCGCTGT-TTTT

>KT823507 Human gammaherpesvirus 4 isolate LC2

AGGACAGCCGTTGCCCTAGTGGTTTCGGACACACCGCCAACGCGCTGTGCGGTGCTGCCGTCCCGAGGTCAAGTCCCGGGGGAGGAGAAGAGCGGCTTCCCG-CCTAGAGCATTTGCAAGTCAGGATTCTCTAATCCCTCTGGGAGAAGGGTATTCGGCTTGTCCGCTGT-TTTT

>KX125050 Human gammaherpesvirus 4 isolate GC-EBV1

AGGACAGCCGTTGCCCTAGTGGTTTCGGACACACCGCCAACGCGCTGTGCGGTGCTGCCGTCCCGAGGTCAAGTCCCGGGGGAGGAGAAGAGCGGCTTCCCG-CCTAGAGCATTTGCAAGTCAGGATTCTCTAATCCCTCTGGGAGAAGGGTATTCGGCTTGTCCGCTGT-TTTT

>LC149491 Human gammaherpesvirus 4 DNA, nearly complete genome, strain: HNNPC2

AGGACAGCCGTTGCCCTAGTGGTTTCGGACACACCGCCAACGCGCTGTGCGGTGCTGCCGTCCCGAGGTCAAGTCCCGGGGGAGGAGAAGAGCGGCTTCCCG-CCTAGAGCATTTGCAAGTCAGGATTCTCTAATCCCTCTGGGAGAAGGGTATTCGGCTTGTCCGCTGT-TTTT

>LC150327 Human gammaherpesvirus 4 DNA, nearly complete genome, strain: HNNPC3

AGGACAGCCGTTGCCCTAGTGGTTTCGGACACACCGCCAACGCGCTGTGCGGTGCTGCCGTCCCGAGGTCAAGTCCCGGGGGAGGAGAAGAGCGGCTTCCCG-CCTAGAGCATTTGCAAGTCAGGATTCTCTAATCCCTCTGGGAGAAGGGTATTCGGCTTGTCCGCTGT-TTTT

>LC150337 Human gammaherpesvirus 4 DNA, nearly complete genome, strain: HNNPC4

AGGACAGCCGTTGCCCTAGTGGTTTCGGACACACCGCCAACGCGCTGTGCGGTGCTGCCGTCCCGAGGTCAAGTCCCGGGGGAGGAGAAGAGCGGCTTCCCG-CCTAGAGCATTTGCAAGTCAGGATTCTCTAATCCCTCTGGGAGAAGGGTATTCGGCTTGTCCGCTGT-TTTT

>LC150338 Human gammaherpesvirus 4 DNA, nearly complete genome, strain: HNNPC5

AGGACAGCCGTTGCCCTAGTGGTTTCGGACACACCGCCAACGCGCTGTGCGGTGCTGCCGTCCCGAGGTCAAGTCCCGGGGGAGGAGAAGAGCGGCTTCCCG-CCTAGAGCATTTGCAAGTCAGGATTCTCTAATCCCTCTGGGAGAAGGGTATTCGGCTTGTCCGCTGT-TTTT

>LC150741 Human gammaherpesvirus 4 DNA, nearly complete genome, strain: HNNPC6

AGGACAGCCGTTGCCCTAGTGGTTTCGGACACACCGCCAACGCGCTGTGCGGTGCTGCCGTCCCGAGGTCAAGTCCCGGGGGAGGAGAAGAGCGGCTTCCCG-CCTAGAGCATTTGCAAGTCAGGATTCTCTAATCCCTCTGGGAGAAGGGTATTCGGCTTGTCCGCTGT-TTTT

>LC150742 Human gammaherpesvirus 4 DNA, nearly complete genome, strain: HNNPC7

AGGACAGCCGTTGCCCTAGTGGTTTCGGACACACCGCCAACGCGCTGTGCGGTGCTGCCGTCCCGAGGTCAAGTCCCGGGGGAGGAGAAGAGCGGCTTCCCG-CCTAGAGCATTTGCAAGTCAGGATTCTCTAATCCCTCTGGGAGAAGGGTATTCGGCTTGTCCGCTGT-TTTT

>LC150743 Human gammaherpesvirus 4 DNA, nearly complete genome, strain: HNNPC8

AGGACAGCCGTTGCCCTAGTGGTTTCGGACACACCGCCAACGCGCTGTGCGGTGCTGCCGTCCCGAGGTCAAGTCCCGGGGGAGGAGAAGAGCGGCTTCCCG-CCTAGAGCATTTGCAAGTCAGGATTCTCTAATCCCTCTGGGAGAAGGGTATTCGGCTTGTCCGCTGT-TTTT

>LN824224 Human herpesvirus 4 genome assembly HKN19, segment : I

AGGACAGCCGTTGCCCTAGTGGTTTCGGACACACCGCCAACGCGCTGTGCGGTGCTGCCGTCCCGAGGTCAAGTCCCGGGGGAGGAGAAGAGCGGCTTCCCG-CCTAGAGCATTTGCAAGTCAGGATTCTCTAATCCCTCTGGGAGAAGGGTATTCGGCTTGTCCGCTGT-TTTT

>LN827525 Human herpesvirus 4 genome assembly C666-1, segment : I

AGGACAGCCGTTGCCCTAGTGGTTTCGGACACACCGCCAACGCGCTGTGCGGTGCTGCCGTCCCGAGGTCAAGTCCCGGGGGAGGAGAAGAGCGGCTTCCCG-CCTAGAGCATTTGCAAGTCAGGATTCTCTAATCCCTCTGGGAGAAGGGTATTCGGCTTGTCCGCTGT-TTTT

>LN827547 Human herpesvirus 4 genome assembly HKN15, segment : I

AGGACAGCCGTTGCCCTAGTGGTTTCGGACACACCGCCAACGCGCTGTGCGGTGCTGCCGTCCCGAGGTCAAGTCCCGGGGGAGGAGAAGAGCGGCTTCCCG-CCTAGAGCATTTGCAAGTCAGGATTCTCTAATCCCTCTGGGAGAAGGGTATTCGGCTTGTCCGCTGT-TTTT

>LN827549 Human herpesvirus 4 genome assembly D3201.2, segment : I

AGGACAGCCGTTGCCCTAGTGGTTTCGGACACACCGCCAACGCGCTGTGCGGTGCTGCCGTCCCGAGGTCAAGTCCCGGGGGAGGAGAAGAGCGGCTTCCCG-CCTAGAGCATTTGCAAGTCAGGATTCTCTAATCCCTCTGGGAGAAGGGTATTCGGCTTGTCCGCTGT-TTTT

>MG298823 Human gammaherpesvirus 4 isolate AH_Saliva_8192

AGGACAGCCGTTGCCCTAGTGGTTTCGGACACACCGCCAACGCGCTGTGCGGTGCTGCCGTCCCGAGGTCAAGTCCCGGGGGAGGAGAAGAGCGGCTTCCCG-CCTAGAGCATTTGCAAGTCAGGATTCTCTAATCCCTCTGGGAGAAGGGTATTCGGCTTGTCCGCTGT-TTTT

>MG298896 Human gammaherpesvirus 4 isolate JM_NPC_bru_L47

AGGACAGCCGTTGCCCTAGTGGTTTCGGACACACCGCCAACGCGCTGTGCGGTGCTGCCGTCCCGAGGTCAAGTCCCGGGGGAGGAGAAGAGCGGCTTCCCG-CCTAGAGCATTTGCAAGTCAGGATTCTCTAATCCCTCTGGGAGAAGGGTATTCGGCTTGTCCGCTGT-TTTT

>MG298906 Human gammaherpesvirus 4 isolate M81_gDNA

AGGACAGCCGTTGCCCTAGTGGTTTCGGACACACCGCCAACGCGCTGTGCGGTGCTGCCGTCCCGAGGTCAAGTCCCGGGGGAGGAGAAGAGCGGCTTCCCG-CCTAGAGCATTTGCAAGTCAGGATTCTCTAATCCCTCTGGGAGAAGGGTATTCGGCTTGTCCGCTGT-TTTT

>MH144212 Human gammaherpesvirus 4 isolate NKTCL-SG01

AGGACAGCCGTTGCCCTAGTGGTTTCGGACACACCGCCAACGCGCTGTGCGGTGCTGCCGTCCCGAGGTCAAGTCCCGGGGGAGGAGAAGAGCGGCTTCCCG-CCTAGAGCATTTGCAAGTCAGGATTCTCTAATCCCTCTGGGAGAAGGGTATTCGGCTTGTCCGCTGT-TTTT

>MH144223 Human gammaherpesvirus 4 isolate NKTCL-SG12

AGGACAGCCGTTGCCCTAGTGGTTTCGGACACACCGCCAACGCGCTGTGCGGTGCTGCCGTCCCGAGGTCAAGTCCCGGGGGAGGAGAAGAGCGGCTTCCCG-CCTAGAGCATTTGCAAGTCAGGATTCTCTAATCCCTCTGGGAGAAGGGTATTCGGCTTGTCCGCTGT-TTTT

>MH590370 Human gammaherpesvirus 4 isolate HKHD1

AGGACAGCCGTTGCCCTAGTGGTTTCGGACACACCGCCAACGCGCTGTGCGGTGCTGCCGTCCCGAGGTCAAGTCCCGGGGGAGGAGAAGAGCGGCTTCCCG-CCTAGAGCATTTGCAAGTCAGGATTCTCTAATCCCTCTGGGAGAAGGGTATTCGGCTTGTCCGCTGT-TTTT

>MH590371 Human gammaherpesvirus 4 isolate HKHD2

AGGACAGCCGTTGCCCTAGTGGTTTCGGACACACCGCCAACGCGCTGTGCGGTGCTGCCGTCCCGAGGTCAAGTCCCGGGGGAGGAGAAGAGCGGCTTCCCG-CCTAGAGCATTTGCAAGTCAGGATTCTCTAATCCCTCTGGGAGAAGGGTATTCGGCTTGTCCGCTGT-TTTT

>MH590372 Human gammaherpesvirus 4 isolate HKHD3

AGGACAGCCGTTGCCCTAGTGGTTTCGGACACACCGCCAACGCGCTGTGCGGTGCTGCCGTCCCGAGGTCAAGTCCCGGGGGAGGAGAAGAGCGGCTTCCCG-CCTAGAGCATTTGCAAGTCAGGATTCTCTAATCCCTCTGGGAGAAGGGTATTCGGCTTGTCCGCTGT-TTTT

>MH590377 Human gammaherpesvirus 4 isolate HKHD8

AGGACAGCCGTTGCCCTAGTGGTTTCGGACACACCGCCAACGCGCTGTGCGGTGCTGCCGTCCCGAGGTCAAGTCCCGGGGGAGGAGAAGAGCGGCTTCCCG-CCTAGAGCATTTGCAAGTCAGGATTCTCTAATCCCTCTGGGAGAAGGGTATTCGGCTTGTCCGCTGT-TTTT

>MH590379 Human gammaherpesvirus 4 isolate HKHD10

AGGACAGCCGTTGCCCTAGTGGTTTCGGACACACCGCCAACGCGCTGTGCGGTGCTGCCGTCCCGAGGTCAAGTCCCGGGGGAGGAGAAGAGCGGCTTCCCG-CCTAGAGCATTTGCAAGTCAGGATTCTCTAATCCCTCTGGGAGAAGGGTATTCGGCTTGTCCGCTGT-TTTT

>MH590383 Human gammaherpesvirus 4 isolate HKHD14

AGGACAGCCGTTGCCCTAGTGGTTTCGGACACACCGCCAACGCGCTGTGCGGTGCTGCCGTCCCGAGGTCAAGTCCCGGGGGAGGAGAAGAGCGGCTTCCCG-CCTAGAGCATTTGCAAGTCAGGATTCTCTAATCCCTCTGGGAGAAGGGTATTCGGCTTGTCCGCTGT-TTTT

>MH590385 Human gammaherpesvirus 4 isolate HKHD16

AGGACAGCCGTTGCCCTAGTGGTTTCGGACACACCGCCAACGCGCTGTGCGGTGCTGCCGTCCCGAGGTCAAGTCCCGGGGGAGGAGAAGAGCGGCTTCCCG-CCTAGAGCATTTGCAAGTCAGGATTCTCTAATCCCTCTGGGAGAAGGGTATTCGGCTTGTCCGCTGT-TTTT

>MH590386 Human gammaherpesvirus 4 isolate HKHD17

AGGACAGCCGTTGCCCTAGTGGTTTCGGACACACCGCCAACGCGCTGTGCGGTGCTGCCGTCCCGAGGTCAAGTCCCGGGGGAGGAGAAGAGCGGCTTCCCG-CCTAGAGCATTTGCAAGTCAGGATTCTCTAATCCCTCTGGGAGAAGGGTATTCGGCTTGTCCGCTGT-TTTT

>MH590388 Human gammaherpesvirus 4 isolate HKHD19

AGGACAGCCGTTGCCCTAGTGGTTTCGGACACACCGCCAACGCGCTGTGCGGTGCTGCCGTCCCGAGGTCAAGTCCCGGGGGAGGAGAAGAGCGGCTTCCCG-CCTAGAGCATTTGCAAGTCAGGATTCTCTAATCCCTCTGGGAGAAGGGTATTCGGCTTGTCCGCTGT-TTTT

>MH590389 Human gammaherpesvirus 4 isolate HKHD20

AGGACAGCCGTTGCCCTAGTGGTTTCGGACACACCGCCAACGCGCTGTGCGGTGCTGCCGTCCCGAGGTCAAGTCCCGGGGGAGGAGAAGAGCGGCTTCCCG-CCTAGAGCATTTGCAAGTCAGGATTCTCTAATCCCTCTGGGAGAAGGGTATTCGGCTTGTCCGCTGT-TTTT

>MH590390 Human gammaherpesvirus 4 isolate HKHD21

AGGACAGCCGTTGCCCTAGTGGTTTCGGACACACCGCCAACGCGCTGTGCGGTGCTGCCGTCCCGAGGTCAAGTCCCGGGGGAGGAGAAGAGCGGCTTCCCG-CCTAGAGCATTTGCAAGTCAGGATTCTCTAATCCCTCTGGGAGAAGGGTATTCGGCTTGTCCGCTGT-TTTT

>MH590392 Human gammaherpesvirus 4 isolate HKHD23

AGGACAGCCGTTGCCCTAGTGGTTTCGGACACACCGCCAACGCGCTGTGCGGTGCTGCCGTCCCGAGGTCAAGTCCCGGGGGAGGAGAAGAGCGGCTTCCCG-CCTAGAGCATTTGCAAGTCAGGATTCTCTAATCCCTCTGGGAGAAGGGTATTCGGCTTGTCCGCTGT-TTTT

>MH590397 Human gammaherpesvirus 4 isolate HKHD28

AGGACAGCCGTTGCCCTAGTGGTTTCGGACACACCGCCAACGCGCTGTGCGGTGCTGCCGTCCCGAGGTCAAGTCCCGGGGGAGGAGAAGAGCGGCTTCCCG-CCTAGAGCATTTGCAAGTCAGGATTCTCTAATCCCTCTGGGAGAAGGGTATTCGGCTTGTCCGCTGT-TTTT

>MH590402 Human gammaherpesvirus 4 isolate HKHD33

AGGACAGCCGTTGCCCTAGTGGTTTCGGACACACCGCCAACGCGCTGTGCGGTGCTGCCGTCCCGAGGTCAAGTCCCGGGGGAGGAGAAGAGCGGCTTCCCG-CCTAGAGCATTTGCAAGTCAGGATTCTCTAATCCCTCTGGGAGAAGGGTATTCGGCTTGTCCGCTGT-TTTT

>MH590403 Human gammaherpesvirus 4 isolate HKHD34

AGGACAGCCGTTGCCCTAGTGGTTTCGGACACACCGCCAACGCGCTGTGCGGTGCTGCCGTCCCGAGGTCAAGTCCCGGGGGAGGAGAAGAGCGGCTTCCCG-CCTAGAGCATTTGCAAGTCAGGATTCTCTAATCCCTCTGGGAGAAGGGTATTCGGCTTGTCCGCTGT-TTTT

>MH590404 Human gammaherpesvirus 4 isolate HKHD35

AGGACAGCCGTTGCCCTAGTGGTTTCGGACACACCGCCAACGCGCTGTGCGGTGCTGCCGTCCCGAGGTCAAGTCCCGGGGGAGGAGAAGAGCGGCTTCCCG-CCTAGAGCATTTGCAAGTCAGGATTCTCTAATCCCTCTGGGAGAAGGGTATTCGGCTTGTCCGCTGT-TTTT

>MH590406 Human gammaherpesvirus 4 isolate HKHD37

AGGACAGCCGTTGCCCTAGTGGTTTCGGACACACCGCCAACGCGCTGTGCGGTGCTGCCGTCCCGAGGTCAAGTCCCGGGGGAGGAGAAGAGCGGCTTCCCG-CCTAGAGCATTTGCAAGTCAGGATTCTCTAATCCCTCTGGGAGAAGGGTATTCGGCTTGTCCGCTGT-TTTT

>MH590408 Human gammaherpesvirus 4 isolate HKHD39

AGGACAGCCGTTGCCCTAGTGGTTTCGGACACACCGCCAACGCGCTGTGCGGTGCTGCCGTCCCGAGGTCAAGTCCCGGGGGAGGAGAAGAGCGGCTTCCCG-CCTAGAGCATTTGCAAGTCAGGATTCTCTAATCCCTCTGGGAGAAGGGTATTCGGCTTGTCCGCTGT-TTTT

>MH590409 Human gammaherpesvirus 4 isolate HKHD40

AGGACAGCCGTTGCCCTAGTGGTTTCGGACACACCGCCAACGCGCTGTGCGGTGCTGCCGTCCCGAGGTCAAGTCCCGGGGGAGGAGAAGAGCGGCTTCCCG-CCTAGAGCATTTGCAAGTCAGGATTCTCTAATCCCTCTGGGAGAAGGGTATTCGGCTTGTCCGCTGT-TTTT

>MH590411 Human gammaherpesvirus 4 isolate HKHD42

AGGACAGCCGTTGCCCTAGTGGTTTCGGACACACCGCCAACGCGCTGTGCGGTGCTGCCGTCCCGAGGTCAAGTCCCGGGGGAGGAGAAGAGCGGCTTCCCG-CCTAGAGCATTTGCAAGTCAGGATTCTCTAATCCCTCTGGGAGAAGGGTATTCGGCTTGTCCGCTGT-TTTT

>MH590414 Human gammaherpesvirus 4 isolate HKHD45

AGGACAGCCGTTGCCCTAGTGGTTTCGGACACACCGCCAACGCGCTGTGCGGTGCTGCCGTCCCGAGGTCAAGTCCCGGGGGAGGAGAAGAGCGGCTTCCCG-CCTAGAGCATTTGCAAGTCAGGATTCTCTAATCCCTCTGGGAGAAGGGTATTCGGCTTGTCCGCTGT-TTTT

>MH590416 Human gammaherpesvirus 4 isolate HKHD47

AGGACAGCCGTTGCCCTAGTGGTTTCGGACACACCGCCAACGCGCTGTGCGGTGCTGCCGTCCCGAGGTCAAGTCCCGGGGGAGGAGAAGAGCGGCTTCCCG-CCTAGAGCATTTGCAAGTCAGGATTCTCTAATCCCTCTGGGAGAAGGGTATTCGGCTTGTCCGCTGT-TTTT

>MH590418 Human gammaherpesvirus 4 isolate HKHD49

AGGACAGCCGTTGCCCTAGTGGTTTCGGACACACCGCCAACGCGCTGTGCGGTGCTGCCGTCCCGAGGTCAAGTCCCGGGGGAGGAGAAGAGCGGCTTCCCG-CCTAGAGCATTTGCAAGTCAGGATTCTCTAATCCCTCTGGGAGAAGGGTATTCGGCTTGTCCGCTGT-TTTT

>MH590423 Human gammaherpesvirus 4 isolate HKHD54

AGGACAGCCGTTGCCCTAGTGGTTTCGGACACACCGCCAACGCGCTGTGCGGTGCTGCCGTCCCGAGGTCAAGTCCCGGGGGAGGAGAAGAGCGGCTTCCCG-CCTAGAGCATTTGCAAGTCAGGATTCTCTAATCCCTCTGGGAGAAGGGTATTCGGCTTGTCCGCTGT-TTTT

>MH590426 Human gammaherpesvirus 4 isolate HKHD57

AGGACAGCCGTTGCCCTAGTGGTTTCGGACACACCGCCAACGCGCTGTGCGGTGCTGCCGTCCCGAGGTCAAGTCCCGGGGGAGGAGAAGAGCGGCTTCCCG-CCTAGAGCATTTGCAAGTCAGGATTCTCTAATCCCTCTGGGAGAAGGGTATTCGGCTTGTCCGCTGT-TTTT

>MH590432 Human gammaherpesvirus 4 isolate HKHD63

AGGACAGCCGTTGCCCTAGTGGTTTCGGACACACCGCCAACGCGCTGTGCGGTGCTGCCGTCCCGAGGTCAAGTCCCGGGGGAGGAGAAGAGCGGCTTCCCG-CCTAGAGCATTTGCAAGTCAGGATTCTCTAATCCCTCTGGGAGAAGGGTATTCGGCTTGTCCGCTGT-TTTT

>MH590433 Human gammaherpesvirus 4 isolate HKHD64

AGGACAGCCGTTGCCCTAGTGGTTTCGGACACACCGCCAACGCGCTGTGCGGTGCTGCCGTCCCGAGGTCAAGTCCCGGGGGAGGAGAAGAGCGGCTTCCCG-CCTAGAGCATTTGCAAGTCAGGATTCTCTAATCCCTCTGGGAGAAGGGTATTCGGCTTGTCCGCTGT-TTTT

>MH590434 Human gammaherpesvirus 4 isolate HKHD65

AGGACAGCCGTTGCCCTAGTGGTTTCGGACACACCGCCAACGCGCTGTGCGGTGCTGCCGTCCCGAGGTCAAGTCCCGGGGGAGGAGAAGAGCGGCTTCCCG-CCTAGAGCATTTGCAAGTCAGGATTCTCTAATCCCTCTGGGAGAAGGGTATTCGGCTTGTCCGCTGT-TTTT

>MH590440 Human gammaherpesvirus 4 isolate HKHD71

AGGACAGCCGTTGCCCTAGTGGTTTCGGACACACCGCCAACGCGCTGTGCGGTGCTGCCGTCCCGAGGTCAAGTCCCGGGGGAGGAGAAGAGCGGCTTCCCG-CCTAGAGCATTTGCAAGTCAGGATTCTCTAATCCCTCTGGGAGAAGGGTATTCGGCTTGTCCGCTGT-TTTT

>MH590442 Human gammaherpesvirus 4 isolate HKHD73

AGGACAGCCGTTGCCCTAGTGGTTTCGGACACACCGCCAACGCGCTGTGCGGTGCTGCCGTCCCGAGGTCAAGTCCCGGGGGAGGAGAAGAGCGGCTTCCCG-CCTAGAGCATTTGCAAGTCAGGATTCTCTAATCCCTCTGGGAGAAGGGTATTCGGCTTGTCCGCTGT-TTTT

>MH590447 Human gammaherpesvirus 4 isolate HKHD78

AGGACAGCCGTTGCCCTAGTGGTTTCGGACACACCGCCAACGCGCTGTGCGGTGCTGCCGTCCCGAGGTCAAGTCCCGGGGGAGGAGAAGAGCGGCTTCCCG-CCTAGAGCATTTGCAAGTCAGGATTCTCTAATCCCTCTGGGAGAAGGGTATTCGGCTTGTCCGCTGT-TTTT

>MH590450 Human gammaherpesvirus 4 isolate HKHD81

AGGACAGCCGTTGCCCTAGTGGTTTCGGACACACCGCCAACGCGCTGTGCGGTGCTGCCGTCCCGAGGTCAAGTCCCGGGGGAGGAGAAGAGCGGCTTCCCG-CCTAGAGCATTTGCAAGTCAGGATTCTCTAATCCCTCTGGGAGAAGGGTATTCGGCTTGTCCGCTGT-TTTT

>MH590451 Human gammaherpesvirus 4 isolate HKHD82

AGGACAGCCGTTGCCCTAGTGGTTTCGGACACACCGCCAACGCGCTGTGCGGTGCTGCCGTCCCGAGGTCAAGTCCCGGGGGAGGAGAAGAGCGGCTTCCCG-CCTAGAGCATTTGCAAGTCAGGATTCTCTAATCCCTCTGGGAGAAGGGTATTCGGCTTGTCCGCTGT-TTTT

>MH590454 Human gammaherpesvirus 4 isolate HKHD85

AGGACAGCCGTTGCCCTAGTGGTTTCGGACACACCGCCAACGCGCTGTGCGGTGCTGCCGTCCCGAGGTCAAGTCCCGGGGGAGGAGAAGAGCGGCTTCCCG-CCTAGAGCATTTGCAAGTCAGGATTCTCTAATCCCTCTGGGAGAAGGGTATTCGGCTTGTCCGCTGT-TTTT

>MH590455 Human gammaherpesvirus 4 isolate HKHD86

AGGACAGCCGTTGCCCTAGTGGTTTCGGACACACCGCCAACGCGCTGTGCGGTGCTGCCGTCCCGAGGTCAAGTCCCGGGGGAGGAGAAGAGCGGCTTCCCG-CCTAGAGCATTTGCAAGTCAGGATTCTCTAATCCCTCTGGGAGAAGGGTATTCGGCTTGTCCGCTGT-TTTT

>MH590456 Human gammaherpesvirus 4 isolate HKHD87

AGGACAGCCGTTGCCCTAGTGGTTTCGGACACACCGCCAACGCGCTGTGCGGTGCTGCCGTCCCGAGGTCAAGTCCCGGGGGAGGAGAAGAGCGGCTTCCCG-CCTAGAGCATTTGCAAGTCAGGATTCTCTAATCCCTCTGGGAGAAGGGTATTCGGCTTGTCCGCTGT-TTTT

>MH590457 Human gammaherpesvirus 4 isolate HKHD88

AGGACAGCCGTTGCCCTAGTGGTTTCGGACACACCGCCAACGCGCTGTGCGGTGCTGCCGTCCCGAGGTCAAGTCCCGGGGGAGGAGAAGAGCGGCTTCCCG-CCTAGAGCATTTGCAAGTCAGGATTCTCTAATCCCTCTGGGAGAAGGGTATTCGGCTTGTCCGCTGT-TTTT

>MH590460 Human gammaherpesvirus 4 isolate HKHD91

AGGACAGCCGTTGCCCTAGTGGTTTCGGACACACCGCCAACGCGCTGTGCGGTGCTGCCGTCCCGAGGTCAAGTCCCGGGGGAGGAGAAGAGCGGCTTCCCG-CCTAGAGCATTTGCAAGTCAGGATTCTCTAATCCCTCTGGGAGAAGGGTATTCGGCTTGTCCGCTGT-TTTT

>MH590461 Human gammaherpesvirus 4 isolate HKHD92

AGGACAGCCGTTGCCCTAGTGGTTTCGGACACACCGCCAACGCGCTGTGCGGTGCTGCCGTCCCGAGGTCAAGTCCCGGGGGAGGAGAAGAGCGGCTTCCCG-CCTAGAGCATTTGCAAGTCAGGATTCTCTAATCCCTCTGGGAGAAGGGTATTCGGCTTGTCCGCTGT-TTTT

>MH590462 Human gammaherpesvirus 4 isolate HKHD93

AGGACAGCCGTTGCCCTAGTGGTTTCGGACACACCGCCAACGCGCTGTGCGGTGCTGCCGTCCCGAGGTCAAGTCCCGGGGGAGGAGAAGAGCGGCTTCCCG-CCTAGAGCATTTGCAAGTCAGGATTCTCTAATCCCTCTGGGAGAAGGGTATTCGGCTTGTCCGCTGT-TTTT

>MH590463 Human gammaherpesvirus 4 isolate HKHD94

AGGACAGCCGTTGCCCTAGTGGTTTCGGACACACCGCCAACGCGCTGTGCGGTGCTGCCGTCCCGAGGTCAAGTCCCGGGGGAGGAGAAGAGCGGCTTCCCG-CCTAGAGCATTTGCAAGTCAGGATTCTCTAATCCCTCTGGGAGAAGGGTATTCGGCTTGTCCGCTGT-TTTT

>MH590469 Human gammaherpesvirus 4 isolate HKHD100

AGGACAGCCGTTGCCCTAGTGGTTTCGGACACACCGCCAACGCGCTGTGCGGTGCTGCCGTCCCGAGGTCAAGTCCCGGGGGAGGAGAAGAGCGGCTTCCCG-CCTAGAGCATTTGCAAGTCAGGATTCTCTAATCCCTCTGGGAGAAGGGTATTCGGCTTGTCCGCTGT-TTTT

>MH590471 Human gammaherpesvirus 4 isolate HKHD102

AGGACAGCCGTTGCCCTAGTGGTTTCGGACACACCGCCAACGCGCTGTGCGGTGCTGCCGTCCCGAGGTCAAGTCCCGGGGGAGGAGAAGAGCGGCTTCCCG-CCTAGAGCATTTGCAAGTCAGGATTCTCTAATCCCTCTGGGAGAAGGGTATTCGGCTTGTCCGCTGT-TTTT

>MH590474 Human gammaherpesvirus 4 isolate HKHD105

AGGACAGCCGTTGCCCTAGTGGTTTCGGACACACCGCCAACGCGCTGTGCGGTGCTGCCGTCCCGAGGTCAAGTCCCGGGGGAGGAGAAGAGCGGCTTCCCG-CCTAGAGCATTTGCAAGTCAGGATTCTCTAATCCCTCTGGGAGAAGGGTATTCGGCTTGTCCGCTGT-TTTT

>MH590477 Human gammaherpesvirus 4 isolate HKHD108

AGGACAGCCGTTGCCCTAGTGGTTTCGGACACACCGCCAACGCGCTGTGCGGTGCTGCCGTCCCGAGGTCAAGTCCCGGGGGAGGAGAAGAGCGGCTTCCCG-CCTAGAGCATTTGCAAGTCAGGATTCTCTAATCCCTCTGGGAGAAGGGTATTCGGCTTGTCCGCTGT-TTTT

>MH590479 Human gammaherpesvirus 4 isolate HKHD110

AGGACAGCCGTTGCCCTAGTGGTTTCGGACACACCGCCAACGCGCTGTGCGGTGCTGCCGTCCCGAGGTCAAGTCCCGGGGGAGGAGAAGAGCGGCTTCCCG-CCTAGAGCATTTGCAAGTCAGGATTCTCTAATCCCTCTGGGAGAAGGGTATTCGGCTTGTCCGCTGT-TTTT

>MH590480 Human gammaherpesvirus 4 isolate HKHD111

AGGACAGCCGTTGCCCTAGTGGTTTCGGACACACCGCCAACGCGCTGTGCGGTGCTGCCGTCCCGAGGTCAAGTCCCGGGGGAGGAGAAGAGCGGCTTCCCG-CCTAGAGCATTTGCAAGTCAGGATTCTCTAATCCCTCTGGGAGAAGGGTATTCGGCTTGTCCGCTGT-TTTT

>MH590497 Human gammaherpesvirus 4 isolate HKHD128

AGGACAGCCGTTGCCCTAGTGGTTTCGGACACACCGCCAACGCGCTGTGCGGTGCTGCCGTCCCGAGGTCAAGTCCCGGGGGAGGAGAAGAGCGGCTTCCCG-CCTAGAGCATTTGCAAGTCAGGATTCTCTAATCCCTCTGGGAGAAGGGTATTCGGCTTGTCCGCTGT-TTTT

>MH590498 Human gammaherpesvirus 4 isolate HKHD129

AGGACAGCCGTTGCCCTAGTGGTTTCGGACACACCGCCAACGCGCTGTGCGGTGCTGCCGTCCCGAGGTCAAGTCCCGGGGGAGGAGAAGAGCGGCTTCCCG-CCTAGAGCATTTGCAAGTCAGGATTCTCTAATCCCTCTGGGAGAAGGGTATTCGGCTTGTCCGCTGT-TTTT

>MH590499 Human gammaherpesvirus 4 isolate HKHD130

AGGACAGCCGTTGCCCTAGTGGTTTCGGACACACCGCCAACGCGCTGTGCGGTGCTGCCGTCCCGAGGTCAAGTCCCGGGGGAGGAGAAGAGCGGCTTCCCG-CCTAGAGCATTTGCAAGTCAGGATTCTCTAATCCCTCTGGGAGAAGGGTATTCGGCTTGTCCGCTGT-TTTT

>MH590500 Human gammaherpesvirus 4 isolate HKHD131

AGGACAGCCGTTGCCCTAGTGGTTTCGGACACACCGCCAACGCGCTGTGCGGTGCTGCCGTCCCGAGGTCAAGTCCCGGGGGAGGAGAAGAGCGGCTTCCCG-CCTAGAGCATTTGCAAGTCAGGATTCTCTAATCCCTCTGGGAGAAGGGTATTCGGCTTGTCCGCTGT-TTTT

>MH590501 Human gammaherpesvirus 4 isolate HKHD132

AGGACAGCCGTTGCCCTAGTGGTTTCGGACACACCGCCAACGCGCTGTGCGGTGCTGCCGTCCCGAGGTCAAGTCCCGGGGGAGGAGAAGAGCGGCTTCCCG-CCTAGAGCATTTGCAAGTCAGGATTCTCTAATCCCTCTGGGAGAAGGGTATTCGGCTTGTCCGCTGT-TTTT

>MH590502 Human gammaherpesvirus 4 isolate HKHD133

AGGACAGCCGTTGCCCTAGTGGTTTCGGACACACCGCCAACGCGCTGTGCGGTGCTGCCGTCCCGAGGTCAAGTCCCGGGGGAGGAGAAGAGCGGCTTCCCG-CCTAGAGCATTTGCAAGTCAGGATTCTCTAATCCCTCTGGGAGAAGGGTATTCGGCTTGTCCGCTGT-TTTT

>MH590504 Human gammaherpesvirus 4 isolate HKHD135

AGGACAGCCGTTGCCCTAGTGGTTTCGGACACACCGCCAACGCGCTGTGCGGTGCTGCCGTCCCGAGGTCAAGTCCCGGGGGAGGAGAAGAGCGGCTTCCCG-CCTAGAGCATTTGCAAGTCAGGATTCTCTAATCCCTCTGGGAGAAGGGTATTCGGCTTGTCCGCTGT-TTTT

>MH590509 Human gammaherpesvirus 4 isolate HKHD140

AGGACAGCCGTTGCCCTAGTGGTTTCGGACACACCGCCAACGCGCTGTGCGGTGCTGCCGTCCCGAGGTCAAGTCCCGGGGGAGGAGAAGAGCGGCTTCCCG-CCTAGAGCATTTGCAAGTCAGGATTCTCTAATCCCTCTGGGAGAAGGGTATTCGGCTTGTCCGCTGT-TTTT

>MH590512 Human gammaherpesvirus 4 isolate HKNPC1

AGGACAGCCGTTGCCCTAGTGGTTTCGGACACACCGCCAACGCGCTGTGCGGTGCTGCCGTCCCGAGGTCAAGTCCCGGGGGAGGAGAAGAGCGGCTTCCCG-CCTAGAGCATTTGCAAGTCAGGATTCTCTAATCCCTCTGGGAGAAGGGTATTCGGCTTGTCCGCTGT-TTTT

>MH590513 Human gammaherpesvirus 4 isolate HKNPC2

AGGACAGCCGTTGCCCTAGTGGTTTCGGACACACCGCCAACGCGCTGTGCGGTGCTGCCGTCCCGAGGTCAAGTCCCGGGGGAGGAGAAGAGCGGCTTCCCG-CCTAGAGCATTTGCAAGTCAGGATTCTCTAATCCCTCTGGGAGAAGGGTATTCGGCTTGTCCGCTGT-TTTT

>MH590514 Human gammaherpesvirus 4 isolate HKNPC3

AGGACAGCCGTTGCCCTAGTGGTTTCGGACACACCGCCAACGCGCTGTGCGGTGCTGCCGTCCCGAGGTCAAGTCCCGGGGGAGGAGAAGAGCGGCTTCCCG-CCTAGAGCATTTGCAAGTCAGGATTCTCTAATCCCTCTGGGAGAAGGGTATTCGGCTTGTCCGCTGT-TTTT

>MH590515 Human gammaherpesvirus 4 isolate HKNPC4

AGGACAGCCGTTGCCCTAGTGGTTTCGGACACACCGCCAACGCGCTGTGCGGTGCTGCCGTCCCGAGGTCAAGTCCCGGGGGAGGAGAAGAGCGGCTTCCCG-CCTAGAGCATTTGCAAGTCAGGATTCTCTAATCCCTCTGGGAGAAGGGTATTCGGCTTGTCCGCTGT-TTTT

>MH590516 Human gammaherpesvirus 4 isolate HKNPC5

AGGACAGCCGTTGCCCTAGTGGTTTCGGACACACCGCCAACGCGCTGTGCGGTGCTGCCGTCCCGAGGTCAAGTCCCGGGGGAGGAGAAGAGCGGCTTCCCG-CCTAGAGCATTTGCAAGTCAGGATTCTCTAATCCCTCTGGGAGAAGGGTATTCGGCTTGTCCGCTGT-TTTT

>MH590517 Human gammaherpesvirus 4 isolate HKNPC6

AGGACAGCCGTTGCCCTAGTGGTTTCGGACACACCGCCAACGCGCTGTGCGGTGCTGCCGTCCCGAGGTCAAGTCCCGGGGGAGGAGAAGAGCGGCTTCCCG-CCTAGAGCATTTGCAAGTCAGGATTCTCTAATCCCTCTGGGAGAAGGGTATTCGGCTTGTCCGCTGT-TTTT

>MH590518 Human gammaherpesvirus 4 isolate HKNPC7

AGGACAGCCGTTGCCCTAGTGGTTTCGGACACACCGCCAACGCGCTGTGCGGTGCTGCCGTCCCGAGGTCAAGTCCCGGGGGAGGAGAAGAGCGGCTTCCCG-CCTAGAGCATTTGCAAGTCAGGATTCTCTAATCCCTCTGGGAGAAGGGTATTCGGCTTGTCCGCTGT-TTTT

>MH590519 Human gammaherpesvirus 4 isolate HKNPC8

AGGACAGCCGTTGCCCTAGTGGTTTCGGACACACCGCCAACGCGCTGTGCGGTGCTGCCGTCCCGAGGTCAAGTCCCGGGGGAGGAGAAGAGCGGCTTCCCG-CCTAGAGCATTTGCAAGTCAGGATTCTCTAATCCCTCTGGGAGAAGGGTATTCGGCTTGTCCGCTGT-TTTT

>MH590520 Human gammaherpesvirus 4 isolate HKNPC9

AGGACAGCCGTTGCCCTAGTGGTTTCGGACACACCGCCAACGCGCTGTGCGGTGCTGCCGTCCCGAGGTCAAGTCCCGGGGGAGGAGAAGAGCGGCTTCCCG-CCTAGAGCATTTGCAAGTCAGGATTCTCTAATCCCTCTGGGAGAAGGGTATTCGGCTTGTCCGCTGT-TTTT

>MH590521 Human gammaherpesvirus 4 isolate HKNPC10

AGGACAGCCGTTGCCCTAGTGGTTTCGGACACACCGCCAACGCGCTGTGCGGTGCTGCCGTCCCGAGGTCAAGTCCCGGGGGAGGAGAAGAGCGGCTTCCCG-CCTAGAGCATTTGCAAGTCAGGATTCTCTAATCCCTCTGGGAGAAGGGTATTCGGCTTGTCCGCTGT-TTTT

>MH590522 Human gammaherpesvirus 4 isolate HKNPC11

AGGACAGCCGTTGCCCTAGTGGTTTCGGACACACCGCCAACGCGCTGTGCGGTGCTGCCGTCCCGAGGTCAAGTCCCGGGGGAGGAGAAGAGCGGCTTCCCG-CCTAGAGCATTTGCAAGTCAGGATTCTCTAATCCCTCTGGGAGAAGGGTATTCGGCTTGTCCGCTGT-TTTT

>MH590523 Human gammaherpesvirus 4 isolate HKNPC12

AGGACAGCCGTTGCCCTAGTGGTTTCGGACACACCGCCAACGCGCTGTGCGGTGCTGCCGTCCCGAGGTCAAGTCCCGGGGGAGGAGAAGAGCGGCTTCCCG-CCTAGAGCATTTGCAAGTCAGGATTCTCTAATCCCTCTGGGAGAAGGGTATTCGGCTTGTCCGCTGT-TTTT

>MH590524 Human gammaherpesvirus 4 isolate HKNPC13

AGGACAGCCGTTGCCCTAGTGGTTTCGGACACACCGCCAACGCGCTGTGCGGTGCTGCCGTCCCGAGGTCAAGTCCCGGGGGAGGAGAAGAGCGGCTTCCCG-CCTAGAGCATTTGCAAGTCAGGATTCTCTAATCCCTCTGGGAGAAGGGTATTCGGCTTGTCCGCTGT-TTTT

>MH590525 Human gammaherpesvirus 4 isolate HKNPC14

AGGACAGCCGTTGCCCTAGTGGTTTCGGACACACCGCCAACGCGCTGTGCGGTGCTGCCGTCCCGAGGTCAAGTCCCGGGGGAGGAGAAGAGCGGCTTCCCG-CCTAGAGCATTTGCAAGTCAGGATTCTCTAATCCCTCTGGGAGAAGGGTATTCGGCTTGTCCGCTGT-TTTT

>MH590526 Human gammaherpesvirus 4 isolate HKNPC15

AGGACAGCCGTTGCCCTAGTGGTTTCGGACACACCGCCAACGCGCTGTGCGGTGCTGCCGTCCCGAGGTCAAGTCCCGGGGGAGGAGAAGAGCGGCTTCCCG-CCTAGAGCATTTGCAAGTCAGGATTCTCTAATCCCTCTGGGAGAAGGGTATTCGGCTTGTCCGCTGT-TTTT

>MH590527 Human gammaherpesvirus 4 isolate HKNPC16

AGGACAGCCGTTGCCCTAGTGGTTTCGGACACACCGCCAACGCGCTGTGCGGTGCTGCCGTCCCGAGGTCAAGTCCCGGGGGAGGAGAAGAGCGGCTTCCCG-CCTAGAGCATTTGCAAGTCAGGATTCTCTAATCCCTCTGGGAGAAGGGTATTCGGCTTGTCCGCTGT-TTTT

>MH590528 Human gammaherpesvirus 4 isolate HKNPC17

AGGACAGCCGTTGCCCTAGTGGTTTCGGACACACCGCCAACGCGCTGTGCGGTGCTGCCGTCCCGAGGTCAAGTCCCGGGGGAGGAGAAGAGCGGCTTCCCG-CCTAGAGCATTTGCAAGTCAGGATTCTCTAATCCCTCTGGGAGAAGGGTATTCGGCTTGTCCGCTGT-TTTT

>MH590529 Human gammaherpesvirus 4 isolate HKNPC18

AGGACAGCCGTTGCCCTAGTGGTTTCGGACACACCGCCAACGCGCTGTGCGGTGCTGCCGTCCCGAGGTCAAGTCCCGGGGGAGGAGAAGAGCGGCTTCCCG-CCTAGAGCATTTGCAAGTCAGGATTCTCTAATCCCTCTGGGAGAAGGGTATTCGGCTTGTCCGCTGT-TTTT

>MH590530 Human gammaherpesvirus 4 isolate HKNPC19

AGGACAGCCGTTGCCCTAGTGGTTTCGGACACACCGCCAACGCGCTGTGCGGTGCTGCCGTCCCGAGGTCAAGTCCCGGGGGAGGAGAAGAGCGGCTTCCCG-CCTAGAGCATTTGCAAGTCAGGATTCTCTAATCCCTCTGGGAGAAGGGTATTCGGCTTGTCCGCTGT-TTTT

>MH590531 Human gammaherpesvirus 4 isolate HKNPC20

AGGACAGCCGTTGCCCTAGTGGTTTCGGACACACCGCCAACGCGCTGTGCGGTGCTGCCGTCCCGAGGTCAAGTCCCGGGGGAGGAGAAGAGCGGCTTCCCG-CCTAGAGCATTTGCAAGTCAGGATTCTCTAATCCCTCTGGGAGAAGGGTATTCGGCTTGTCCGCTGT-TTTT

>MH590532 Human gammaherpesvirus 4 isolate HKNPC21

AGGACAGCCGTTGCCCTAGTGGTTTCGGACACACCGCCAACGCGCTGTGCGGTGCTGCCGTCCCGAGGTCAAGTCCCGGGGGAGGAGAAGAGCGGCTTCCCG-CCTAGAGCATTTGCAAGTCAGGATTCTCTAATCCCTCTGGGAGAAGGGTATTCGGCTTGTCCGCTGT-TTTT

>MH590533 Human gammaherpesvirus 4 isolate HKNPC22

AGGACAGCCGTTGCCCTAGTGGTTTCGGACACACCGCCAACGCGCTGTGCGGTGCTGCCGTCCCGAGGTCAAGTCCCGGGGGAGGAGAAGAGCGGCTTCCCG-CCTAGAGCATTTGCAAGTCAGGATTCTCTAATCCCTCTGGGAGAAGGGTATTCGGCTTGTCCGCTGT-TTTT

>MH590534 Human gammaherpesvirus 4 isolate HKNPC23

AGGACAGCCGTTGCCCTAGTGGTTTCGGACACACCGCCAACGCGCTGTGCGGTGCTGCCGTCCCGAGGTCAAGTCCCGGGGGAGGAGAAGAGCGGCTTCCCG-CCTAGAGCATTTGCAAGTCAGGATTCTCTAATCCCTCTGGGAGAAGGGTATTCGGCTTGTCCGCTGT-TTTT

>MH590535 Human gammaherpesvirus 4 isolate HKNPC24

AGGACAGCCGTTGCCCTAGTGGTTTCGGACACACCGCCAACGCGCTGTGCGGTGCTGCCGTCCCGAGGTCAAGTCCCGGGGGAGGAGAAGAGCGGCTTCCCG-CCTAGAGCATTTGCAAGTCAGGATTCTCTAATCCCTCTGGGAGAAGGGTATTCGGCTTGTCCGCTGT-TTTT

>MH590536 Human gammaherpesvirus 4 isolate HKNPC25

AGGACAGCCGTTGCCCTAGTGGTTTCGGACACACCGCCAACGCGCTGTGCGGTGCTGCCGTCCCGAGGTCAAGTCCCGGGGGAGGAGAAGAGCGGCTTCCCG-CCTAGAGCATTTGCAAGTCAGGATTCTCTAATCCCTCTGGGAGAAGGGTATTCGGCTTGTCCGCTGT-TTTT

>MH590537 Human gammaherpesvirus 4 isolate HKNPC26

AGGACAGCCGTTGCCCTAGTGGTTTCGGACACACCGCCAACGCGCTGTGCGGTGCTGCCGTCCCGAGGTCAAGTCCCGGGGGAGGAGAAGAGCGGCTTCCCG-CCTAGAGCATTTGCAAGTCAGGATTCTCTAATCCCTCTGGGAGAAGGGTATTCGGCTTGTCCGCTGT-TTTT

>MH590538 Human gammaherpesvirus 4 isolate HKNPC27

AGGACAGCCGTTGCCCTAGTGGTTTCGGACACACCGCCAACGCGCTGTGCGGTGCTGCCGTCCCGAGGTCAAGTCCCGGGGGAGGAGAAGAGCGGCTTCCCG-CCTAGAGCATTTGCAAGTCAGGATTCTCTAATCCCTCTGGGAGAAGGGTATTCGGCTTGTCCGCTGT-TTTT

>MH590540 Human gammaherpesvirus 4 isolate HKNPC29

AGGACAGCCGTTGCCCTAGTGGTTTCGGACACACCGCCAACGCGCTGTGCGGTGCTGCCGTCCCGAGGTCAAGTCCCGGGGGAGGAGAAGAGCGGCTTCCCG-CCTAGAGCATTTGCAAGTCAGGATTCTCTAATCCCTCTGGGAGAAGGGTATTCGGCTTGTCCGCTGT-TTTT

>MH590541 Human gammaherpesvirus 4 isolate HKNPC30

AGGACAGCCGTTGCCCTAGTGGTTTCGGACACACCGCCAACGCGCTGTGCGGTGCTGCCGTCCCGAGGTCAAGTCCCGGGGGAGGAGAAGAGCGGCTTCCCG-CCTAGAGCATTTGCAAGTCAGGATTCTCTAATCCCTCTGGGAGAAGGGTATTCGGCTTGTCCGCTGT-TTTT

>MH590542 Human gammaherpesvirus 4 isolate HKNPC31

AGGACAGCCGTTGCCCTAGTGGTTTCGGACACACCGCCAACGCGCTGTGCGGTGCTGCCGTCCCGAGGTCAAGTCCCGGGGGAGGAGAAGAGCGGCTTCCCG-CCTAGAGCATTTGCAAGTCAGGATTCTCTAATCCCTCTGGGAGAAGGGTATTCGGCTTGTCCGCTGT-TTTT

>MH590543 Human gammaherpesvirus 4 isolate HKNPC32

AGGACAGCCGTTGCCCTAGTGGTTTCGGACACACCGCCAACGCGCTGTGCGGTGCTGCCGTCCCGAGGTCAAGTCCCGGGGGAGGAGAAGAGCGGCTTCCCG-CCTAGAGCATTTGCAAGTCAGGATTCTCTAATCCCTCTGGGAGAAGGGTATTCGGCTTGTCCGCTGT-TTTT

>MH590544 Human gammaherpesvirus 4 isolate HKNPC33

AGGACAGCCGTTGCCCTAGTGGTTTCGGACACACCGCCAACGCGCTGTGCGGTGCTGCCGTCCCGAGGTCAAGTCCCGGGGGAGGAGAAGAGCGGCTTCCCG-CCTAGAGCATTTGCAAGTCAGGATTCTCTAATCCCTCTGGGAGAAGGGTATTCGGCTTGTCCGCTGT-TTTT

>MH590545 Human gammaherpesvirus 4 isolate HKNPC34

AGGACAGCCGTTGCCCTAGTGGTTTCGGACACACCGCCAACGCGCTGTGCGGTGCTGCCGTCCCGAGGTCAAGTCCCGGGGGAGGAGAAGAGCGGCTTCCCG-CCTAGAGCATTTGCAAGTCAGGATTCTCTAATCCCTCTGGGAGAAGGGTATTCGGCTTGTCCGCTGT-TTTT

>MH590546 Human gammaherpesvirus 4 isolate HKNPC35

AGGACAGCCGTTGCCCTAGTGGTTTCGGACACACCGCCAACGCGCTGTGCGGTGCTGCCGTCCCGAGGTCAAGTCCCGGGGGAGGAGAAGAGCGGCTTCCCG-CCTAGAGCATTTGCAAGTCAGGATTCTCTAATCCCTCTGGGAGAAGGGTATTCGGCTTGTCCGCTGT-TTTT

>MH590547 Human gammaherpesvirus 4 isolate HKNPC36

AGGACAGCCGTTGCCCTAGTGGTTTCGGACACACCGCCAACGCGCTGTGCGGTGCTGCCGTCCCGAGGTCAAGTCCCGGGGGAGGAGAAGAGCGGCTTCCCG-CCTAGAGCATTTGCAAGTCAGGATTCTCTAATCCCTCTGGGAGAAGGGTATTCGGCTTGTCCGCTGT-TTTT

>MH590548 Human gammaherpesvirus 4 isolate HKNPC37

AGGACAGCCGTTGCCCTAGTGGTTTCGGACACACCGCCAACGCGCTGTGCGGTGCTGCCGTCCCGAGGTCAAGTCCCGGGGGAGGAGAAGAGCGGCTTCCCG-CCTAGAGCATTTGCAAGTCAGGATTCTCTAATCCCTCTGGGAGAAGGGTATTCGGCTTGTCCGCTGT-TTTT

>MH590549 Human gammaherpesvirus 4 isolate HKNPC38

AGGACAGCCGTTGCCCTAGTGGTTTCGGACACACCGCCAACGCGCTGTGCGGTGCTGCCGTCCCGAGGTCAAGTCCCGGGGGAGGAGAAGAGCGGCTTCCCG-CCTAGAGCATTTGCAAGTCAGGATTCTCTAATCCCTCTGGGAGAAGGGTATTCGGCTTGTCCGCTGT-TTTT

>MH590550 Human gammaherpesvirus 4 isolate HKNPC39

AGGACAGCCGTTGCCCTAGTGGTTTCGGACACACCGCCAACGCGCTGTGCGGTGCTGCCGTCCCGAGGTCAAGTCCCGGGGGAGGAGAAGAGCGGCTTCCCG-CCTAGAGCATTTGCAAGTCAGGATTCTCTAATCCCTCTGGGAGAAGGGTATTCGGCTTGTCCGCTGT-TTTT

>MH590552 Human gammaherpesvirus 4 isolate HKNPC41

AGGACAGCCGTTGCCCTAGTGGTTTCGGACACACCGCCAACGCGCTGTGCGGTGCTGCCGTCCCGAGGTCAAGTCCCGGGGGAGGAGAAGAGCGGCTTCCCG-CCTAGAGCATTTGCAAGTCAGGATTCTCTAATCCCTCTGGGAGAAGGGTATTCGGCTTGTCCGCTGT-TTTT

>MH590553 Human gammaherpesvirus 4 isolate HKNPC42

AGGACAGCCGTTGCCCTAGTGGTTTCGGACACACCGCCAACGCGCTGTGCGGTGCTGCCGTCCCGAGGTCAAGTCCCGGGGGAGGAGAAGAGCGGCTTCCCG-CCTAGAGCATTTGCAAGTCAGGATTCTCTAATCCCTCTGGGAGAAGGGTATTCGGCTTGTCCGCTGT-TTTT

>MH590555 Human gammaherpesvirus 4 isolate HKNPC44

AGGACAGCCGTTGCCCTAGTGGTTTCGGACACACCGCCAACGCGCTGTGCGGTGCTGCCGTCCCGAGGTCAAGTCCCGGGGGAGGAGAAGAGCGGCTTCCCG-CCTAGAGCATTTGCAAGTCAGGATTCTCTAATCCCTCTGGGAGAAGGGTATTCGGCTTGTCCGCTGT-TTTT

>MH590556 Human gammaherpesvirus 4 isolate HKNPC45

AGGACAGCCGTTGCCCTAGTGGTTTCGGACACACCGCCAACGCGCTGTGCGGTGCTGCCGTCCCGAGGTCAAGTCCCGGGGGAGGAGAAGAGCGGCTTCCCG-CCTAGAGCATTTGCAAGTCAGGATTCTCTAATCCCTCTGGGAGAAGGGTATTCGGCTTGTCCGCTGT-TTTT

>MH590557 Human gammaherpesvirus 4 isolate HKNPC46

AGGACAGCCGTTGCCCTAGTGGTTTCGGACACACCGCCAACGCGCTGTGCGGTGCTGCCGTCCCGAGGTCAAGTCCCGGGGGAGGAGAAGAGCGGCTTCCCG-CCTAGAGCATTTGCAAGTCAGGATTCTCTAATCCCTCTGGGAGAAGGGTATTCGGCTTGTCCGCTGT-TTTT

>MH590558 Human gammaherpesvirus 4 isolate HKNPC47

AGGACAGCCGTTGCCCTAGTGGTTTCGGACACACCGCCAACGCGCTGTGCGGTGCTGCCGTCCCGAGGTCAAGTCCCGGGGGAGGAGAAGAGCGGCTTCCCG-CCTAGAGCATTTGCAAGTCAGGATTCTCTAATCCCTCTGGGAGAAGGGTATTCGGCTTGTCCGCTGT-TTTT

>MH590559 Human gammaherpesvirus 4 isolate HKNPC48

AGGACAGCCGTTGCCCTAGTGGTTTCGGACACACCGCCAACGCGCTGTGCGGTGCTGCCGTCCCGAGGTCAAGTCCCGGGGGAGGAGAAGAGCGGCTTCCCG-CCTAGAGCATTTGCAAGTCAGGATTCTCTAATCCCTCTGGGAGAAGGGTATTCGGCTTGTCCGCTGT-TTTT

>MH590560 Human gammaherpesvirus 4 isolate HKNPC49

AGGACAGCCGTTGCCCTAGTGGTTTCGGACACACCGCCAACGCGCTGTGCGGTGCTGCCGTCCCGAGGTCAAGTCCCGGGGGAGGAGAAGAGCGGCTTCCCG-CCTAGAGCATTTGCAAGTCAGGATTCTCTAATCCCTCTGGGAGAAGGGTATTCGGCTTGTCCGCTGT-TTTT

>MH590561 Human gammaherpesvirus 4 isolate HKNPC50

AGGACAGCCGTTGCCCTAGTGGTTTCGGACACACCGCCAACGCGCTGTGCGGTGCTGCCGTCCCGAGGTCAAGTCCCGGGGGAGGAGAAGAGCGGCTTCCCG-CCTAGAGCATTTGCAAGTCAGGATTCTCTAATCCCTCTGGGAGAAGGGTATTCGGCTTGTCCGCTGT-TTTT

>MH590562 Human gammaherpesvirus 4 isolate HKNPC51

AGGACAGCCGTTGCCCTAGTGGTTTCGGACACACCGCCAACGCGCTGTGCGGTGCTGCCGTCCCGAGGTCAAGTCCCGGGGGAGGAGAAGAGCGGCTTCCCG-CCTAGAGCATTTGCAAGTCAGGATTCTCTAATCCCTCTGGGAGAAGGGTATTCGGCTTGTCCGCTGT-TTTT

>MH590563 Human gammaherpesvirus 4 isolate HKNPC52

AGGACAGCCGTTGCCCTAGTGGTTTCGGACACACCGCCAACGCGCTGTGCGGTGCTGCCGTCCCGAGGTCAAGTCCCGGGGGAGGAGAAGAGCGGCTTCCCG-CCTAGAGCATTTGCAAGTCAGGATTCTCTAATCCCTCTGGGAGAAGGGTATTCGGCTTGTCCGCTGT-TTTT

>MH590564 Human gammaherpesvirus 4 isolate HKNPC53

AGGACAGCCGTTGCCCTAGTGGTTTCGGACACACCGCCAACGCGCTGTGCGGTGCTGCCGTCCCGAGGTCAAGTCCCGGGGGAGGAGAAGAGCGGCTTCCCG-CCTAGAGCATTTGCAAGTCAGGATTCTCTAATCCCTCTGGGAGAAGGGTATTCGGCTTGTCCGCTGT-TTTT

>MH590565 Human gammaherpesvirus 4 isolate HKNPC54

AGGACAGCCGTTGCCCTAGTGGTTTCGGACACACCGCCAACGCGCTGTGCGGTGCTGCCGTCCCGAGGTCAAGTCCCGGGGGAGGAGAAGAGCGGCTTCCCG-CCTAGAGCATTTGCAAGTCAGGATTCTCTAATCCCTCTGGGAGAAGGGTATTCGGCTTGTCCGCTGT-TTTT

>MH590566 Human gammaherpesvirus 4 isolate HKNPC55

AGGACAGCCGTTGCCCTAGTGGTTTCGGACACACCGCCAACGCGCTGTGCGGTGCTGCCGTCCCGAGGTCAAGTCCCGGGGGAGGAGAAGAGCGGCTTCCCG-CCTAGAGCATTTGCAAGTCAGGATTCTCTAATCCCTCTGGGAGAAGGGTATTCGGCTTGTCCGCTGT-TTTT

>MH590567 Human gammaherpesvirus 4 isolate HKNPC56

AGGACAGCCGTTGCCCTAGTGGTTTCGGACACACCGCCAACGCGCTGTGCGGTGCTGCCGTCCCGAGGTCAAGTCCCGGGGGAGGAGAAGAGCGGCTTCCCG-CCTAGAGCATTTGCAAGTCAGGATTCTCTAATCCCTCTGGGAGAAGGGTATTCGGCTTGTCCGCTGT-TTTT

>MH590568 Human gammaherpesvirus 4 isolate HKNPC57

AGGACAGCCGTTGCCCTAGTGGTTTCGGACACACCGCCAACGCGCTGTGCGGTGCTGCCGTCCCGAGGTCAAGTCCCGGGGGAGGAGAAGAGCGGCTTCCCG-CCTAGAGCATTTGCAAGTCAGGATTCTCTAATCCCTCTGGGAGAAGGGTATTCGGCTTGTCCGCTGT-TTTT

>MH590569 Human gammaherpesvirus 4 isolate HKNPC58

AGGACAGCCGTTGCCCTAGTGGTTTCGGACACACCGCCAACGCGCTGTGCGGTGCTGCCGTCCCGAGGTCAAGTCCCGGGGGAGGAGAAGAGCGGCTTCCCG-CCTAGAGCATTTGCAAGTCAGGATTCTCTAATCCCTCTGGGAGAAGGGTATTCGGCTTGTCCGCTGT-TTTT

>MH590570 Human gammaherpesvirus 4 isolate HKNPC59

AGGACAGCCGTTGCCCTAGTGGTTTCGGACACACCGCCAACGCGCTGTGCGGTGCTGCCGTCCCGAGGTCAAGTCCCGGGGGAGGAGAAGAGCGGCTTCCCG-CCTAGAGCATTTGCAAGTCAGGATTCTCTAATCCCTCTGGGAGAAGGGTATTCGGCTTGTCCGCTGT-TTTT

>MH590571 Human gammaherpesvirus 4 isolate HKNPC60

AGGACAGCCGTTGCCCTAGTGGTTTCGGACACACCGCCAACGCGCTGTGCGGTGCTGCCGTCCCGAGGTCAAGTCCCGGGGGAGGAGAAGAGCGGCTTCCCG-CCTAGAGCATTTGCAAGTCAGGATTCTCTAATCCCTCTGGGAGAAGGGTATTCGGCTTGTCCGCTGT-TTTT

>MH590572 Human gammaherpesvirus 4 isolate HKNPC61

AGGACAGCCGTTGCCCTAGTGGTTTCGGACACACCGCCAACGCGCTGTGCGGTGCTGCCGTCCCGAGGTCAAGTCCCGGGGGAGGAGAAGAGCGGCTTCCCG-CCTAGAGCATTTGCAAGTCAGGATTCTCTAATCCCTCTGGGAGAAGGGTATTCGGCTTGTCCGCTGT-TTTT

>MH590573 Human gammaherpesvirus 4 isolate HKNPC62

AGGACAGCCGTTGCCCTAGTGGTTTCGGACACACCGCCAACGCGCTGTGCGGTGCTGCCGTCCCGAGGTCAAGTCCCGGGGGAGGAGAAGAGCGGCTTCCCG-CCTAGAGCATTTGCAAGTCAGGATTCTCTAATCCCTCTGGGAGAAGGGTATTCGGCTTGTCCGCTGT-TTTT

>MK540241 Human gammaherpesvirus 4 isolate BLT001

AGGACAGCCGTTGCCCTAGTGGTTTCGGACACACCGCCAACGCGCTGTGCGGTGCTGCCGTCCCGAGGTCAAGTCCCGGGGGAGGAGAAGAGCGGCTTCCCG-CCTAGAGCATTTGCAAGTCAGGATTCTCTAATCCCTCTGGGAGAAGGGTATTCGGCTTGTCCGCTATGTTTT

>MK540243 Human gammaherpesvirus 4 isolate C666

AGGACAGCCGTTGCCCTAGTGGTTTCGGACACACCGCCAACGCGCTGTGCGGTGCTGCCGTCCCGAGGTCAAGTCCCGGGGGAGGAGAAGAGCGGCTTCCCG-CCTAGAGCATTTGCAAGTCAGGATTCTCTAATCCCTCTGGGAGAAGGGTATTCGGCTTGTCCGCTATGTTTT

>MK540258 Human gammaherpesvirus 4 isolate HLT002

AGGACAGCCGTTGCCCTAGTGGTTTCGGACACACCGCCAACGCGCTGTGCGGTGCTGCCGTCCCGAGGTCAAGTCCCGGGGGAGGAGAAGAGCGGCTTCCCG-CCTAGAGCATTTGCAAGTCAGGATTCTCTAATCCCTCTGGGAGAAGGGTATTCGGCTTGTCCGCTATGTTTT

>MK540265 Human gammaherpesvirus 4 isolate HS003

AGGACAGCCGTTGCCCTAGTGGTTTCGGACACACCGCCAACGCGCTGTGCGGTGCTGCCGTCCCGAGGTCAAGTCCCGGGGGAGGAGAAGAGCGGCTTCCCG-CCTAGAGCATTTGCAAGTCAGGATTCTCTAATCCCTCTGGGAGAAGGGTATTCGGCTTGTCCGCTATGTTTT

>MK540266 Human gammaherpesvirus 4 isolate HS007

AGGACAGCCGTTGCCCTAGTGGTTTCGGACACACCGCCAACGCGCTGTGCGGTGCTGCCGTCCCGAGGTCAAGTCCCGGGGGAGGAGAAGAGCGGCTTCCCG-CCTAGAGCATTTGCAAGTCAGGATTCTCTAATCCCTCTGGGAGAAGGGTATTCGGCTTGTCCGCTATGTTTT

>MK540267 Human gammaherpesvirus 4 isolate HS008

AGGACAGCCGTTGCCCTAGTGGTTTCGGACACACCGCCAACGCGCTGTGCGGTGCTGCCGTCCCGAGGTCAAGTCCCGGGGGAGGAGAAGAGCGGCTTCCCG-CCTAGAGCATTTGCAAGTCAGGATTCTCTAATCCCTCTGGGAGAAGGGTATTCGGCTTGTCCGCTATGTTTT

>MK540268 Human gammaherpesvirus 4 isolate HS009

AGGACAGCCGTTGCCCTAGTGGTTTCGGACACACCGCCAACGCGCTGTGCGGTGCTGCCGTCCCGAGGTCAAGTCCCGGGGGAGGAGAAGAGCGGCTTCCCG-CCTAGAGCATTTGCAAGTCAGGATTCTCTAATCCCTCTGGGAGAAGGGTATTCGGCTTGTCCGCTATGTTTT

>MK540270 Human gammaherpesvirus 4 isolate HS012

AGGACAGCCGTTGCCCTAGTGGTTTCGGACACACCGCCAACGCGCTGTGCGGTGCTGCCGTCCCGAGGTCAAGTCCCGGGGGAGGAGAAGAGCGGCTTCCCG-CCTAGAGCATTTGCAAGTCAGGATTCTCTAATCCCTCTGGGAGAAGGGTATTCGGCTTGTCCGCTATGTTTT

>MK540271 Human gammaherpesvirus 4 isolate HS013

AGGACAGCCGTTGCCCTAGTGGTTTCGGACACACCGCCAACGCGCTGTGCGGTGCTGCCGTCCCGAGGTCAAGTCCCGGGGGAGGAGAAGAGCGGCTTCCCG-CCTAGAGCATTTGCAAGTCAGGATTCTCTAATCCCTCTGGGAGAAGGGTATTCGGCTTGTCCGCTATGTTTT

>MK540272 Human gammaherpesvirus 4 isolate HS014

AGGACAGCCGTTGCCCTAGTGGTTTCGGACACACCGCCAACGCGCTGTGCGGTGCTGCCGTCCCGAGGTCAAGTCCCGGGGGAGGAGAAGAGCGGCTTCCCG-CCTAGAGCATTTGCAAGTCAGGATTCTCTAATCCCTCTGGGAGAAGGGTATTCGGCTTGTCCGCTATGTTTT

>MK540274 Human gammaherpesvirus 4 isolate HS016

AGGACAGCCGTTGCCCTAGTGGTTTCGGACACACCGCCAACGCGCTGTGCGGTGCTGCCGTCCCGAGGTCAAGTCCCGGGGGAGGAGAAGAGCGGCTTCCCG-CCTAGAGCATTTGCAAGTCAGGATTCTCTAATCCCTCTGGGAGAAGGGTATTCGGCTTGTCCGCTATGTTTT

>MK540276 Human gammaherpesvirus 4 isolate HS019

AGGACAGCCGTTGCCCTAGTGGTTTCGGACACACCGCCAACGCGCTGTGCGGTGCTGCCGTCCCGAGGTCAAGTCCCGGGGGAGGAGAAGAGCGGCTTCCCG-CCTAGAGCATTTGCAAGTCAGGATTCTCTAATCCCTCTGGGAGAAGGGTATTCGGCTTGTCCGCTATGTTTT

>MK540277 Human gammaherpesvirus 4 isolate HS020

AGGACAGCCGTTGCCCTAGTGGTTTCGGACACACCGCCAACGCGCTGTGCGGTGCTGCCGTCCCGAGGTCAAGTCCCGGGGGAGGAGAAGAGCGGCTTCCCG-CCTAGAGCATTTGCAAGTCAGGATTCTCTAATCCCTCTGGGAGAAGGGTATTCGGCTTGTCCGCTATGTTTT

>MK540278 Human gammaherpesvirus 4 isolate HS021

AGGACAGCCGTTGCCCTAGTGGTTTCGGACACACCGCCAACGCGCTGTGCGGTGCTGCCGTCCCGAGGTCAAGTCCCGGGGGAGGAGAAGAGCGGCTTCCCG-CCTAGAGCATTTGCAAGTCAGGATTCTCTAATCCCTCTGGGAGAAGGGTATTCGGCTTGTCCGCTATGTTTT

>MK540282 Human gammaherpesvirus 4 isolate HS027

AGGACAGCCGTTGCCCTAGTGGTTTCGGACACACCGCCAACGCGCTGTGCGGTGCTGCCGTCCCGAGGTCAAGTCCCGGGGGAGGAGAAGAGCGGCTTCCCG-CCTAGAGCATTTGCAAGTCAGGATTCTCTAATCCCTCTGGGAGAAGGGTATTCGGCTTGTCCGCTATGTTTT

>MK540283 Human gammaherpesvirus 4 isolate HS029

AGGACAGCCGTTGCCCTAGTGGTTTCGGACACACCGCCAACGCGCTGTGCGGTGCTGCCGTCCCGAGGTCAAGTCCCGGGGGAGGAGAAGAGCGGCTTCCCG-CCTAGAGCATTTGCAAGTCAGGATTCTCTAATCCCTCTGGGAGAAGGGTATTCGGCTTGTCCGCTATGTTTT

>MK540289 Human gammaherpesvirus 4 isolate HS037

AGGACAGCCGTTGCCCTAGTGGTTTCGGACACACCGCCAACGCGCTGTGCGGTGCTGCCGTCCCGAGGTCAAGTCCCGGGGGAGGAGAAGAGCGGCTTCCCG-CCTAGAGCATTTGCAAGTCAGGATTCTCTAATCCCTCTGGGAGAAGGGTATTCGGCTTGTCCGCTATGTTTT

>MK540290 Human gammaherpesvirus 4 isolate HS038

AGGACAGCCGTTGCCCTAGTGGTTTCGGACACACCGCCAACGCGCTGTGCGGTGCTGCCGTCCCGAGGTCAAGTCCCGGGGGAGGAGAAGAGCGGCTTCCCG-CCTAGAGCATTTGCAAGTCAGGATTCTCTAATCCCTCTGGGAGAAGGGTATTCGGCTTGTCCGCTATGTTTT

>MK540293 Human gammaherpesvirus 4 isolate HS045

AGGACAGCCGTTGCCCTAGTGGTTTCGGACACACCGCCAACGCGCTGTGCGGTGCTGCCGTCCCGAGGTCAAGTCCCGGGGGAGGAGAAGAGCGGCTTCCCG-CCTAGAGCATTTGCAAGTCAGGATTCTCTAATCCCTCTGGGAGAAGGGTATTCGGCTTGTCCGCTATGTTTT

>MK540295 Human gammaherpesvirus 4 isolate HS050

AGGACAGCCGTTGCCCTAGTGGTTTCGGACACACCGCCAACGCGCTGTGCGGTGCTGCCGTCCCGAGGTCAAGTCCCGGGGGAGGAGAAGAGCGGCTTCCCG-CCTAGAGCATTTGCAAGTCAGGATTCTCTAATCCCTCTGGGAGAAGGGTATTCGGCTTGTCCGCTATGTTTT

>MK540296 Human gammaherpesvirus 4 isolate HS051

AGGACAGCCGTTGCCCTAGTGGTTTCGGACACACCGCCAACGCGCTGTGCGGTGCTGCCGTCCCGAGGTCAAGTCCCGGGGGAGGAGAAGAGCGGCTTCCCG-CCTAGAGCATTTGCAAGTCAGGATTCTCTAATCCCTCTGGGAGAAGGGTATTCGGCTTGTCCGCTATGTTTT

>MK540297 Human gammaherpesvirus 4 isolate HS052

AGGACAGCCGTTGCCCTAGTGGTTTCGGACACACCGCCAACGCGCTGTGCGGTGCTGCCGTCCCGAGGTCAAGTCCCGGGGGAGGAGAAGAGCGGCTTCCCG-CCTAGAGCATTTGCAAGTCAGGATTCTCTAATCCCTCTGGGAGAAGGGTATTCGGCTTGTCCGCTATGTTTT

>MK540298 Human gammaherpesvirus 4 isolate HS053

AGGACAGCCGTTGCCCTAGTGGTTTCGGACACACCGCCAACGCGCTGTGCGGTGCTGCCGTCCCGAGGTCAAGTCCCGGGGGAGGAGAAGAGCGGCTTCCCG-CCTAGAGCATTTGCAAGTCAGGATTCTCTAATCCCTCTGGGAGAAGGGTATTCGGCTTGTCCGCTATGTTTT

>MK540299 Human gammaherpesvirus 4 isolate HS054

AGGACAGCCGTTGCCCTAGTGGTTTCGGACACACCGCCAACGCGCTGTGCGGTGCTGCCGTCCCGAGGTCAAGTCCCGGGGGAGGAGAAGAGCGGCTTCCCG-CCTAGAGCATTTGCAAGTCAGGATTCTCTAATCCCTCTGGGAGAAGGGTATTCGGCTTGTCCGCTATGTTTT

>MK540306 Human gammaherpesvirus 4 isolate NKLT006

AGGACAGCCGTTGCCCTAGTGGTTTCGGACACACCGCCAACGCGCTGTGCGGTGCTGCCGTCCCGAGGTCAAGTCCCGGGGGAGGAGAAGAGCGGCTTCCCG-CCTAGAGCATTTGCAAGTCAGGATTCTCTAATCCCTCTGGGAGAAGGGTATTCGGCTTGTCCGCTATGTTTT

>MK540308 Human gammaherpesvirus 4 isolate NNPCT001

AGGACAGCCGTTGCCCTAGTGGTTTCGGACACACCGCCAACGCGCTGTGCGGTGCTGCCGTCCCGAGGTCAAGTCCCGGGGGAGGAGAAGAGCGGCTTCCCG-CCTAGAGCATTTGCAAGTCAGGATTCTCTAATCCCTCTGGGAGAAGGGTATTCGGCTTGTCCGCTATGTTTT

>MK540310 Human gammaherpesvirus 4 isolate NNPCT003

AGGACAGCCGTTGCCCTAGTGGTTTCGGACACACCGCCAACGCGCTGTGCGGTGCTGCCGTCCCGAGGTCAAGTCCCGGGGGAGGAGAAGAGCGGCTTCCCG-CCTAGAGCATTTGCAAGTCAGGATTCTCTAATCCCTCTGGGAGAAGGGTATTCGGCTTGTCCGCTATGTTTT

>MK540311 Human gammaherpesvirus 4 isolate NNPCT004

AGGACAGCCGTTGCCCTAGTGGTTTCGGACACACCGCCAACGCGCTGTGCGGTGCTGCCGTCCCGAGGTCAAGTCCCGGGGGAGGAGAAGAGCGGCTTCCCG-CCTAGAGCATTTGCAAGTCAGGATTCTCTAATCCCTCTGGGAGAAGGGTATTCGGCTTGTCCGCTATGTTTT

>MK540313 Human gammaherpesvirus 4 isolate NPCP001

AGGACAGCCGTTGCCCTAGTGGTTTCGGACACACCGCCAACGCGCTGTGCGGTGCTGCCGTCCCGAGGTCAAGTCCCGGGGGAGGAGAAGAGCGGCTTCCCG-CCTAGAGCATTTGCAAGTCAGGATTCTCTAATCCCTCTGGGAGAAGGGTATTCGGCTTGTCCGCTATGTTTT

>MK540314 Human gammaherpesvirus 4 isolate NPCS001

AGGACAGCCGTTGCCCTAGTGGTTTCGGACACACCGCCAACGCGCTGTGCGGTGCTGCCGTCCCGAGGTCAAGTCCCGGGGGAGGAGAAGAGCGGCTTCCCG-CCTAGAGCATTTGCAAGTCAGGATTCTCTAATCCCTCTGGGAGAAGGGTATTCGGCTTGTCCGCTATGTTTT

>MK540315 Human gammaherpesvirus 4 isolate NPCS002

AGGACAGCCGTTGCCCTAGTGGTTTCGGACACACCGCCAACGCGCTGTGCGGTGCTGCCGTCCCGAGGTCAAGTCCCGGGGGAGGAGAAGAGCGGCTTCCCG-CCTAGAGCATTTGCAAGTCAGGATTCTCTAATCCCTCTGGGAGAAGGGTATTCGGCTTGTCCGCTATGTTTT

>MK540316 Human gammaherpesvirus 4 isolate NPCS003-2

AGGACAGCCGTTGCCCTAGTGGTTTCGGACACACCGCCAACGCGCTGTGCGGTGCTGCCGTCCCGAGGTCAAGTCCCGGGGGAGGAGAAGAGCGGCTTCCCG-CCTAGAGCATTTGCAAGTCAGGATTCTCTAATCCCTCTGGGAGAAGGGTATTCGGCTTGTCCGCTATGTTTT

>MK540317 Human gammaherpesvirus 4 isolate NPCS005

AGGACAGCCGTTGCCCTAGTGGTTTCGGACACACCGCCAACGCGCTGTGCGGTGCTGCCGTCCCGAGGTCAAGTCCCGGGGGAGGAGAAGAGCGGCTTCCCG-CCTAGAGCATTTGCAAGTCAGGATTCTCTAATCCCTCTGGGAGAAGGGTATTCGGCTTGTCCGCTATGTTTT

>MK540318 Human gammaherpesvirus 4 isolate NPCS006

AGGACAGCCGTTGCCCTAGTGGTTTCGGACACACCGCCAACGCGCTGTGCGGTGCTGCCGTCCCGAGGTCAAGTCCCGGGGGAGGAGAAGAGCGGCTTCCCG-CCTAGAGCATTTGCAAGTCAGGATTCTCTAATCCCTCTGGGAGAAGGGTATTCGGCTTGTCCGCTATGTTTT

>MK540319 Human gammaherpesvirus 4 isolate NPCS007

AGGACAGCCGTTGCCCTAGTGGTTTCGGACACACCGCCAACGCGCTGTGCGGTGCTGCCGTCCCGAGGTCAAGTCCCGGGGGAGGAGAAGAGCGGCTTCCCG-CCTAGAGCATTTGCAAGTCAGGATTCTCTAATCCCTCTGGGAGAAGGGTATTCGGCTTGTCCGCTATGTTTT

>MK540320 Human gammaherpesvirus 4 isolate NPCS008

AGGACAGCCGTTGCCCTAGTGGTTTCGGACACACCGCCAACGCGCTGTGCGGTGCTGCCGTCCCGAGGTCAAGTCCCGGGGGAGGAGAAGAGCGGCTTCCCG-CCTAGAGCATTTGCAAGTCAGGATTCTCTAATCCCTCTGGGAGAAGGGTATTCGGCTTGTCCGCTATGTTTT

>MK540321 Human gammaherpesvirus 4 isolate NPCS009

AGGACAGCCGTTGCCCTAGTGGTTTCGGACACACCGCCAACGCGCTGTGCGGTGCTGCCGTCCCGAGGTCAAGTCCCGGGGGAGGAGAAGAGCGGCTTCCCG-CCTAGAGCATTTGCAAGTCAGGATTCTCTAATCCCTCTGGGAGAAGGGTATTCGGCTTGTCCGCTATGTTTT

>MK540322 Human gammaherpesvirus 4 isolate NPCS010

AGGACAGCCGTTGCCCTAGTGGTTTCGGACACACCGCCAACGCGCTGTGCGGTGCTGCCGTCCCGAGGTCAAGTCCCGGGGGAGGAGAAGAGCGGCTTCCCG-CCTAGAGCATTTGCAAGTCAGGATTCTCTAATCCCTCTGGGAGAAGGGTATTCGGCTTGTCCGCTATGTTTT

>MK540323 Human gammaherpesvirus 4 isolate NPCS011

AGGACAGCCGTTGCCCTAGTGGTTTCGGACACACCGCCAACGCGCTGTGCGGTGCTGCCGTCCCGAGGTCAAGTCCCGGGGGAGGAGAAGAGCGGCTTCCCG-CCTAGAGCATTTGCAAGTCAGGATTCTCTAATCCCTCTGGGAGAAGGGTATTCGGCTTGTCCGCTATGTTTT

>MK540324 Human gammaherpesvirus 4 isolate NPCS012

AGGACAGCCGTTGCCCTAGTGGTTTCGGACACACCGCCAACGCGCTGTGCGGTGCTGCCGTCCCGAGGTCAAGTCCCGGGGGAGGAGAAGAGCGGCTTCCCG-CCTAGAGCATTTGCAAGTCAGGATTCTCTAATCCCTCTGGGAGAAGGGTATTCGGCTTGTCCGCTATGTTTT

>MK540325 Human gammaherpesvirus 4 isolate NPCS013

AGGACAGCCGTTGCCCTAGTGGTTTCGGACACACCGCCAACGCGCTGTGCGGTGCTGCCGTCCCGAGGTCAAGTCCCGGGGGAGGAGAAGAGCGGCTTCCCG-CCTAGAGCATTTGCAAGTCAGGATTCTCTAATCCCTCTGGGAGAAGGGTATTCGGCTTGTCCGCTATGTTTT

>MK540326 Human gammaherpesvirus 4 isolate NPCS014

AGGACAGCCGTTGCCCTAGTGGTTTCGGACACACCGCCAACGCGCTGTGCGGTGCTGCCGTCCCGAGGTCAAGTCCCGGGGGAGGAGAAGAGCGGCTTCCCG-CCTAGAGCATTTGCAAGTCAGGATTCTCTAATCCCTCTGGGAGAAGGGTATTCGGCTTGTCCGCTATGTTTT

>MK540327 Human gammaherpesvirus 4 isolate NPCS016

AGGACAGCCGTTGCCCTAGTGGTTTCGGACACACCGCCAACGCGCTGTGCGGTGCTGCCGTCCCGAGGTCAAGTCCCGGGGGAGGAGAAGAGCGGCTTCCCG-CCTAGAGCATTTGCAAGTCAGGATTCTCTAATCCCTCTGGGAGAAGGGTATTCGGCTTGTCCGCTATGTTTT

>MK540328 Human gammaherpesvirus 4 isolate NPCS017

AGGACAGCCGTTGCCCTAGTGGTTTCGGACACACCGCCAACGCGCTGTGCGGTGCTGCCGTCCCGAGGTCAAGTCCCGGGGGAGGAGAAGAGCGGCTTCCCG-CCTAGAGCATTTGCAAGTCAGGATTCTCTAATCCCTCTGGGAGAAGGGTATTCGGCTTGTCCGCTATGTTTT

>MK540329 Human gammaherpesvirus 4 isolate NPCS018

AGGACAGCCGTTGCCCTAGTGGTTTCGGACACACCGCCAACGCGCTGTGCGGTGCTGCCGTCCCGAGGTCAAGTCCCGGGGGAGGAGAAGAGCGGCTTCCCG-CCTAGAGCATTTGCAAGTCAGGATTCTCTAATCCCTCTGGGAGAAGGGTATTCGGCTTGTCCGCTATGTTTT

>MK540330 Human gammaherpesvirus 4 isolate NPCS019

AGGACAGCCGTTGCCCTAGTGGTTTCGGACACACCGCCAACGCGCTGTGCGGTGCTGCCGTCCCGAGGTCAAGTCCCGGGGGAGGAGAAGAGCGGCTTCCCG-CCTAGAGCATTTGCAAGTCAGGATTCTCTAATCCCTCTGGGAGAAGGGTATTCGGCTTGTCCGCTATGTTTT

>MK540331 Human gammaherpesvirus 4 isolate NPCS021

AGGACAGCCGTTGCCCTAGTGGTTTCGGACACACCGCCAACGCGCTGTGCGGTGCTGCCGTCCCGAGGTCAAGTCCCGGGGGAGGAGAAGAGCGGCTTCCCG-CCTAGAGCATTTGCAAGTCAGGATTCTCTAATCCCTCTGGGAGAAGGGTATTCGGCTTGTCCGCTATGTTTT

>MK540332 Human gammaherpesvirus 4 isolate NPCS022

AGGACAGCCGTTGCCCTAGTGGTTTCGGACACACCGCCAACGCGCTGTGCGGTGCTGCCGTCCCGAGGTCAAGTCCCGGGGGAGGAGAAGAGCGGCTTCCCG-CCTAGAGCATTTGCAAGTCAGGATTCTCTAATCCCTCTGGGAGAAGGGTATTCGGCTTGTCCGCTATGTTTT

>MK540333 Human gammaherpesvirus 4 isolate NPCS023

AGGACAGCCGTTGCCCTAGTGGTTTCGGACACACCGCCAACGCGCTGTGCGGTGCTGCCGTCCCGAGGTCAAGTCCCGGGGGAGGAGAAGAGCGGCTTCCCG-CCTAGAGCATTTGCAAGTCAGGATTCTCTAATCCCTCTGGGAGAAGGGTATTCGGCTTGTCCGCTATGTTTT

>MK540334 Human gammaherpesvirus 4 isolate NPCS024

AGGACAGCCGTTGCCCTAGTGGTTTCGGACACACCGCCAACGCGCTGTGCGGTGCTGCCGTCCCGAGGTCAAGTCCCGGGGGAGGAGAAGAGCGGCTTCCCG-CCTAGAGCATTTGCAAGTCAGGATTCTCTAATCCCTCTGGGAGAAGGGTATTCGGCTTGTCCGCTATGTTTT

>MK540335 Human gammaherpesvirus 4 isolate NPCS025

AGGACAGCCGTTGCCCTAGTGGTTTCGGACACACCGCCAACGCGCTGTGCGGTGCTGCCGTCCCGAGGTCAAGTCCCGGGGGAGGAGAAGAGCGGCTTCCCG-CCTAGAGCATTTGCAAGTCAGGATTCTCTAATCCCTCTGGGAGAAGGGTATTCGGCTTGTCCGCTATGTTTT

>MK540336 Human gammaherpesvirus 4 isolate NPCS026

AGGACAGCCGTTGCCCTAGTGGTTTCGGACACACCGCCAACGCGCTGTGCGGTGCTGCCGTCCCGAGGTCAAGTCCCGGGGGAGGAGAAGAGCGGCTTCCCG-CCTAGAGCATTTGCAAGTCAGGATTCTCTAATCCCTCTGGGAGAAGGGTATTCGGCTTGTCCGCTATGTTTT

>MK540337 Human gammaherpesvirus 4 isolate NPCS027

AGGACAGCCGTTGCCCTAGTGGTTTCGGACACACCGCCAACGCGCTGTGCGGTGCTGCCGTCCCGAGGTCAAGTCCCGGGGGAGGAGAAGAGCGGCTTCCCG-CCTAGAGCATTTGCAAGTCAGGATTCTCTAATCCCTCTGGGAGAAGGGTATTCGGCTTGTCCGCTATGTTTT

>MK540338 Human gammaherpesvirus 4 isolate NPCS028

AGGACAGCCGTTGCCCTAGTGGTTTCGGACACACCGCCAACGCGCTGTGCGGTGCTGCCGTCCCGAGGTCAAGTCCCGGGGGAGGAGAAGAGCGGCTTCCCG-CCTAGAGCATTTGCAAGTCAGGATTCTCTAATCCCTCTGGGAGAAGGGTATTCGGCTTGTCCGCTATGTTTT

>MK540339 Human gammaherpesvirus 4 isolate NPCS029

AGGACAGCCGTTGCCCTAGTGGTTTCGGACACACCGCCAACGCGCTGTGCGGTGCTGCCGTCCCGAGGTCAAGTCCCGGGGGAGGAGAAGAGCGGCTTCCCG-CCTAGAGCATTTGCAAGTCAGGATTCTCTAATCCCTCTGGGAGAAGGGTATTCGGCTTGTCCGCTATGTTTT

>MK540342 Human gammaherpesvirus 4 isolate NPCS033

AGGACAGCCGTTGCCCTAGTGGTTTCGGACACACCGCCAACGCGCTGTGCGGTGCTGCCGTCCCGAGGTCAAGTCCCGGGGGAGGAGAAGAGCGGCTTCCCG-CCTAGAGCATTTGCAAGTCAGGATTCTCTAATCCCTCTGGGAGAAGGGTATTCGGCTTGTCCGCTATGTTTT

>MK540343 Human gammaherpesvirus 4 isolate NPCS034

AGGACAGCCGTTGCCCTAGTGGTTTCGGACACACCGCCAACGCGCTGTGCGGTGCTGCCGTCCCGAGGTCAAGTCCCGGGGGAGGAGAAGAGCGGCTTCCCG-CCTAGAGCATTTGCAAGTCAGGATTCTCTAATCCCTCTGGGAGAAGGGTATTCGGCTTGTCCGCTATGTTTT

>MK540345 Human gammaherpesvirus 4 isolate NPCS038

AGGACAGCCGTTGCCCTAGTGGTTTCGGACACACCGCCAACGCGCTGTGCGGTGCTGCCGTCCCGAGGTCAAGTCCCGGGGGAGGAGAAGAGCGGCTTCCCG-CCTAGAGCATTTGCAAGTCAGGATTCTCTAATCCCTCTGGGAGAAGGGTATTCGGCTTGTCCGCTATGTTTT

>MK540346 Human gammaherpesvirus 4 isolate NPCS039

AGGACAGCCGTTGCCCTAGTGGTTTCGGACACACCGCCAACGCGCTGTGCGGTGCTGCCGTCCCGAGGTCAAGTCCCGGGGGAGGAGAAGAGCGGCTTCCCG-CCTAGAGCATTTGCAAGTCAGGATTCTCTAATCCCTCTGGGAGAAGGGTATTCGGCTTGTCCGCTATGTTTT

>MK540347 Human gammaherpesvirus 4 isolate NPCS040

AGGACAGCCGTTGCCCTAGTGGTTTCGGACACACCGCCAACGCGCTGTGCGGTGCTGCCGTCCCGAGGTCAAGTCCCGGGGGAGGAGAAGAGCGGCTTCCCG-CCTAGAGCATTTGCAAGTCAGGATTCTCTAATCCCTCTGGGAGAAGGGTATTCGGCTTGTCCGCTATGTTTT

>MK540349 Human gammaherpesvirus 4 isolate NPCS044

AGGACAGCCGTTGCCCTAGTGGTTTCGGACACACCGCCAACGCGCTGTGCGGTGCTGCCGTCCCGAGGTCAAGTCCCGGGGGAGGAGAAGAGCGGCTTCCCG-CCTAGAGCATTTGCAAGTCAGGATTCTCTAATCCCTCTGGGAGAAGGGTATTCGGCTTGTCCGCTATGTTTT

>MK540350 Human gammaherpesvirus 4 isolate NPCS045

AGGACAGCCGTTGCCCTAGTGGTTTCGGACACACCGCCAACGCGCTGTGCGGTGCTGCCGTCCCGAGGTCAAGTCCCGGGGGAGGAGAAGAGCGGCTTCCCG-CCTAGAGCATTTGCAAGTCAGGATTCTCTAATCCCTCTGGGAGAAGGGTATTCGGCTTGTCCGCTATGTTTT

>MK540351 Human gammaherpesvirus 4 isolate NPCS046

AGGACAGCCGTTGCCCTAGTGGTTTCGGACACACCGCCAACGCGCTGTGCGGTGCTGCCGTCCCGAGGTCAAGTCCCGGGGGAGGAGAAGAGCGGCTTCCCG-CCTAGAGCATTTGCAAGTCAGGATTCTCTAATCCCTCTGGGAGAAGGGTATTCGGCTTGTCCGCTATGTTTT

>MK540352 Human gammaherpesvirus 4 isolate NPCS047

AGGACAGCCGTTGCCCTAGTGGTTTCGGACACACCGCCAACGCGCTGTGCGGTGCTGCCGTCCCGAGGTCAAGTCCCGGGGGAGGAGAAGAGCGGCTTCCCG-CCTAGAGCATTTGCAAGTCAGGATTCTCTAATCCCTCTGGGAGAAGGGTATTCGGCTTGTCCGCTATGTTTT

>MK540353 Human gammaherpesvirus 4 isolate NPCS048

AGGACAGCCGTTGCCCTAGTGGTTTCGGACACACCGCCAACGCGCTGTGCGGTGCTGCCGTCCCGAGGTCAAGTCCCGGGGGAGGAGAAGAGCGGCTTCCCG-CCTAGAGCATTTGCAAGTCAGGATTCTCTAATCCCTCTGGGAGAAGGGTATTCGGCTTGTCCGCTATGTTTT

>MK540355 Human gammaherpesvirus 4 isolate NPCS050

AGGACAGCCGTTGCCCTAGTGGTTTCGGACACACCGCCAACGCGCTGTGCGGTGCTGCCGTCCCGAGGTCAAGTCCCGGGGGAGGAGAAGAGCGGCTTCCCG-CCTAGAGCATTTGCAAGTCAGGATTCTCTAATCCCTCTGGGAGAAGGGTATTCGGCTTGTCCGCTATGTTTT

>MK540356 Human gammaherpesvirus 4 isolate NPCS051

AGGACAGCCGTTGCCCTAGTGGTTTCGGACACACCGCCAACGCGCTGTGCGGTGCTGCCGTCCCGAGGTCAAGTCCCGGGGGAGGAGAAGAGCGGCTTCCCG-CCTAGAGCATTTGCAAGTCAGGATTCTCTAATCCCTCTGGGAGAAGGGTATTCGGCTTGTCCGCTATGTTTT

>MK540357 Human gammaherpesvirus 4 isolate NPCS052

AGGACAGCCGTTGCCCTAGTGGTTTCGGACACACCGCCAACGCGCTGTGCGGTGCTGCCGTCCCGAGGTCAAGTCCCGGGGGAGGAGAAGAGCGGCTTCCCG-CCTAGAGCATTTGCAAGTCAGGATTCTCTAATCCCTCTGGGAGAAGGGTATTCGGCTTGTCCGCTATGTTTT

>MK540358 Human gammaherpesvirus 4 isolate NPCS054

AGGACAGCCGTTGCCCTAGTGGTTTCGGACACACCGCCAACGCGCTGTGCGGTGCTGCCGTCCCGAGGTCAAGTCCCGGGGGAGGAGAAGAGCGGCTTCCCG-CCTAGAGCATTTGCAAGTCAGGATTCTCTAATCCCTCTGGGAGAAGGGTATTCGGCTTGTCCGCTATGTTTT

>MK540360 Human gammaherpesvirus 4 isolate NPCT002

AGGACAGCCGTTGCCCTAGTGGTTTCGGACACACCGCCAACGCGCTGTGCGGTGCTGCCGTCCCGAGGTCAAGTCCCGGGGGAGGAGAAGAGCGGCTTCCCG-CCTAGAGCATTTGCAAGTCAGGATTCTCTAATCCCTCTGGGAGAAGGGTATTCGGCTTGTCCGCTATGTTTT

>MK540361 Human gammaherpesvirus 4 isolate NPCT003

AGGACAGCCGTTGCCCTAGTGGTTTCGGACACACCGCCAACGCGCTGTGCGGTGCTGCCGTCCCGAGGTCAAGTCCCGGGGGAGGAGAAGAGCGGCTTCCCG-CCTAGAGCATTTGCAAGTCAGGATTCTCTAATCCCTCTGGGAGAAGGGTATTCGGCTTGTCCGCTATGTTTT

>MK540363 Human gammaherpesvirus 4 isolate NPCT005

AGGACAGCCGTTGCCCTAGTGGTTTCGGACACACCGCCAACGCGCTGTGCGGTGCTGCCGTCCCGAGGTCAAGTCCCGGGGGAGGAGAAGAGCGGCTTCCCG-CCTAGAGCATTTGCAAGTCAGGATTCTCTAATCCCTCTGGGAGAAGGGTATTCGGCTTGTCCGCTATGTTTT

>MK540364 Human gammaherpesvirus 4 isolate NPCT006

AGGACAGCCGTTGCCCTAGTGGTTTCGGACACACCGCCAACGCGCTGTGCGGTGCTGCCGTCCCGAGGTCAAGTCCCGGGGGAGGAGAAGAGCGGCTTCCCG-CCTAGAGCATTTGCAAGTCAGGATTCTCTAATCCCTCTGGGAGAAGGGTATTCGGCTTGTCCGCTATGTTTT

>MK540365 Human gammaherpesvirus 4 isolate NPCT007

AGGACAGCCGTTGCCCTAGTGGTTTCGGACACACCGCCAACGCGCTGTGCGGTGCTGCCGTCCCGAGGTCAAGTCCCGGGGGAGGAGAAGAGCGGCTTCCCG-CCTAGAGCATTTGCAAGTCAGGATTCTCTAATCCCTCTGGGAGAAGGGTATTCGGCTTGTCCGCTATGTTTT

>MK540366 Human gammaherpesvirus 4 isolate NPCT008

AGGACAGCCGTTGCCCTAGTGGTTTCGGACACACCGCCAACGCGCTGTGCGGTGCTGCCGTCCCGAGGTCAAGTCCCGGGGGAGGAGAAGAGCGGCTTCCCG-CCTAGAGCATTTGCAAGTCAGGATTCTCTAATCCCTCTGGGAGAAGGGTATTCGGCTTGTCCGCTATGTTTT

>MK540368 Human gammaherpesvirus 4 isolate NPCT010

AGGACAGCCGTTGCCCTAGTGGTTTCGGACACACCGCCAACGCGCTGTGCGGTGCTGCCGTCCCGAGGTCAAGTCCCGGGGGAGGAGAAGAGCGGCTTCCCG-CCTAGAGCATTTGCAAGTCAGGATTCTCTAATCCCTCTGGGAGAAGGGTATTCGGCTTGTCCGCTATGTTTT

>MK540370 Human gammaherpesvirus 4 isolate NPCT012

AGGACAGCCGTTGCCCTAGTGGTTTCGGACACACCGCCAACGCGCTGTGCGGTGCTGCCGTCCCGAGGTCAAGTCCCGGGGGAGGAGAAGAGCGGCTTCCCG-CCTAGAGCATTTGCAAGTCAGGATTCTCTAATCCCTCTGGGAGAAGGGTATTCGGCTTGTCCGCTATGTTTT

>MK540371 Human gammaherpesvirus 4 isolate NPCT013

AGGACAGCCGTTGCCCTAGTGGTTTCGGACACACCGCCAACGCGCTGTGCGGTGCTGCCGTCCCGAGGTCAAGTCCCGGGGGAGGAGAAGAGCGGCTTCCCG-CCTAGAGCATTTGCAAGTCAGGATTCTCTAATCCCTCTGGGAGAAGGGTATTCGGCTTGTCCGCTATGTTTT

>MK540373 Human gammaherpesvirus 4 isolate NPCT015

AGGACAGCCGTTGCCCTAGTGGTTTCGGACACACCGCCAACGCGCTGTGCGGTGCTGCCGTCCCGAGGTCAAGTCCCGGGGGAGGAGAAGAGCGGCTTCCCG-CCTAGAGCATTTGCAAGTCAGGATTCTCTAATCCCTCTGGGAGAAGGGTATTCGGCTTGTCCGCTATGTTTT

>MK540374 Human gammaherpesvirus 4 isolate NPCT017

AGGACAGCCGTTGCCCTAGTGGTTTCGGACACACCGCCAACGCGCTGTGCGGTGCTGCCGTCCCGAGGTCAAGTCCCGGGGGAGGAGAAGAGCGGCTTCCCG-CCTAGAGCATTTGCAAGTCAGGATTCTCTAATCCCTCTGGGAGAAGGGTATTCGGCTTGTCCGCTATGTTTT

>MK540375 Human gammaherpesvirus 4 isolate NPCT018

AGGACAGCCGTTGCCCTAGTGGTTTCGGACACACCGCCAACGCGCTGTGCGGTGCTGCCGTCCCGAGGTCAAGTCCCGGGGGAGGAGAAGAGCGGCTTCCCG-CCTAGAGCATTTGCAAGTCAGGATTCTCTAATCCCTCTGGGAGAAGGGTATTCGGCTTGTCCGCTATGTTTT

>MK540376 Human gammaherpesvirus 4 isolate NPCT019

AGGACAGCCGTTGCCCTAGTGGTTTCGGACACACCGCCAACGCGCTGTGCGGTGCTGCCGTCCCGAGGTCAAGTCCCGGGGGAGGAGAAGAGCGGCTTCCCG-CCTAGAGCATTTGCAAGTCAGGATTCTCTAATCCCTCTGGGAGAAGGGTATTCGGCTTGTCCGCTATGTTTT

>MK540377 Human gammaherpesvirus 4 isolate NPCT020-2

AGGACAGCCGTTGCCCTAGTGGTTTCGGACACACCGCCAACGCGCTGTGCGGTGCTGCCGTCCCGAGGTCAAGTCCCGGGGGAGGAGAAGAGCGGCTTCCCG-CCTAGAGCATTTGCAAGTCAGGATTCTCTAATCCCTCTGGGAGAAGGGTATTCGGCTTGTCCGCTATGTTTT

>MK540379 Human gammaherpesvirus 4 isolate NPCT022

AGGACAGCCGTTGCCCTAGTGGTTTCGGACACACCGCCAACGCGCTGTGCGGTGCTGCCGTCCCGAGGTCAAGTCCCGGGGGAGGAGAAGAGCGGCTTCCCG-CCTAGAGCATTTGCAAGTCAGGATTCTCTAATCCCTCTGGGAGAAGGGTATTCGGCTTGTCCGCTATGTTTT

>MK540380 Human gammaherpesvirus 4 isolate NPCT023

AGGACAGCCGTTGCCCTAGTGGTTTCGGACACACCGCCAACGCGCTGTGCGGTGCTGCCGTCCCGAGGTCAAGTCCCGGGGGAGGAGAAGAGCGGCTTCCCG-CCTAGAGCATTTGCAAGTCAGGATTCTCTAATCCCTCTGGGAGAAGGGTATTCGGCTTGTCCGCTATGTTTT

>MK540381 Human gammaherpesvirus 4 isolate NPCT024

AGGACAGCCGTTGCCCTAGTGGTTTCGGACACACCGCCAACGCGCTGTGCGGTGCTGCCGTCCCGAGGTCAAGTCCCGGGGGAGGAGAAGAGCGGCTTCCCG-CCTAGAGCATTTGCAAGTCAGGATTCTCTAATCCCTCTGGGAGAAGGGTATTCGGCTTGTCCGCTATGTTTT

>MK540382 Human gammaherpesvirus 4 isolate NPCT025

AGGACAGCCGTTGCCCTAGTGGTTTCGGACACACCGCCAACGCGCTGTGCGGTGCTGCCGTCCCGAGGTCAAGTCCCGGGGGAGGAGAAGAGCGGCTTCCCG-CCTAGAGCATTTGCAAGTCAGGATTCTCTAATCCCTCTGGGAGAAGGGTATTCGGCTTGTCCGCTATGTTTT

>MK540383 Human gammaherpesvirus 4 isolate NPCT027

AGGACAGCCGTTGCCCTAGTGGTTTCGGACACACCGCCAACGCGCTGTGCGGTGCTGCCGTCCCGAGGTCAAGTCCCGGGGGAGGAGAAGAGCGGCTTCCCG-CCTAGAGCATTTGCAAGTCAGGATTCTCTAATCCCTCTGGGAGAAGGGTATTCGGCTTGTCCGCTATGTTTT

>MK540386 Human gammaherpesvirus 4 isolate NPCT031

AGGACAGCCGTTGCCCTAGTGGTTTCGGACACACCGCCAACGCGCTGTGCGGTGCTGCCGTCCCGAGGTCAAGTCCCGGGGGAGGAGAAGAGCGGCTTCCCG-CCTAGAGCATTTGCAAGTCAGGATTCTCTAATCCCTCTGGGAGAAGGGTATTCGGCTTGTCCGCTATGTTTT

>MK540387 Human gammaherpesvirus 4 isolate NPCT032

AGGACAGCCGTTGCCCTAGTGGTTTCGGACACACCGCCAACGCGCTGTGCGGTGCTGCCGTCCCGAGGTCAAGTCCCGGGGGAGGAGAAGAGCGGCTTCCCG-CCTAGAGCATTTGCAAGTCAGGATTCTCTAATCCCTCTGGGAGAAGGGTATTCGGCTTGTCCGCTATGTTTT

>MK540388 Human gammaherpesvirus 4 isolate NPCT033

AGGACAGCCGTTGCCCTAGTGGTTTCGGACACACCGCCAACGCGCTGTGCGGTGCTGCCGTCCCGAGGTCAAGTCCCGGGGGAGGAGAAGAGCGGCTTCCCG-CCTAGAGCATTTGCAAGTCAGGATTCTCTAATCCCTCTGGGAGAAGGGTATTCGGCCTGTCCGCTAT-TTTT

>MK540389 Human gammaherpesvirus 4 isolate NPCT035

AGGACAGCCGTTGCCCTAGTGGTTTCGGACACACCGCCAACGCGCTGTGCGGTGCTGCCGTCCCGAGGTCAAGTCCCGGGGGAGGAGAAGAGCGGCTTCCCG-CCTAGAGCATTTGCAAGTCAGGATTCTCTAATCCCTCTGGGAGAAGGGTATTCGGCTTGTCCGCTATGTTTT

>MK540390 Human gammaherpesvirus 4 isolate NPCT036

AGGACAGCCGTTGCCCTAGTGGTTTCGGACACACCGCCAACGCGCTGTGCGGTGCTGCCGTCCCGAGGTCAAGTCCCGGGGGAGGAGAAGAGCGGCTTCCCG-CCTAGAGCATTTGCAAGTCAGGATTCTCTAATCCCTCTGGGAGAAGGGTATTCGGCTTGTCCGCTATGTTTT

>MK540391 Human gammaherpesvirus 4 isolate NPCT037

AGGACAGCCGTTGCCCTAGTGGTTTCGGACACACCGCCAACGCGCTGTGCGGTGCTGCCGTCCCGAGGTCAAGTCCCGGGGGAGGAGAAGAGCGGCTTCCCG-CCTAGAGCATTTGCAAGTCAGGATTCTCTAATCCCTCTGGGAGAAGGGTATTCGGCTTGTCCGCTATGTTTT

>MK540392 Human gammaherpesvirus 4 isolate NPCT038

AGGACAGCCGTTGCCCTAGTGGTTTCGGACACACCGCCAACGCGCTGTGCGGTGCTGCCGTCCCGAGGTCAAGTCCCGGGGGAGGAGAAGAGCGGCTTCCCG-CCTAGAGCATTTGCAAGTCAGGATTCTCTAATCCCTCTGGGAGAAGGGTATTCGGCTTGTCCGCTATGTTTT

>MK540393 Human gammaherpesvirus 4 isolate NPCT039

AGGACAGCCGTTGCCCTAGTGGTTTCGGACACACCGCCAACGCGCTGTGCGGTGCTGCCGTCCCGAGGTCAAGTCCCGGGGGAGGAGAAGAGCGGCTTCCCG-CCTAGAGCATTTGCAAGTCAGGATTCTCTAATCCCTCTGGGAGAAGGGTATTCGGCTTGTCCGCTATGTTTT

>MK540394 Human gammaherpesvirus 4 isolate NPCT040

AGGACAGCCGTTGCCCTAGTGGTTTCGGACACACCGCCAACGCGCTGTGCGGTGCTGCCGTCCCGAGGTCAAGTCCCGGGGGAGGAGAAGAGCGGCTTCCCG-CCTAGAGCATTTGCAAGTCAGGATTCTCTAATCCCTCTGGGAGAAGGGTATTCGGCTTGTCCGCTATGTTTT

>MK540395 Human gammaherpesvirus 4 isolate NPCT041

AGGACAGCCGTTGCCCTAGTGGTTTCGGACACACCGCCAACGCGCTGTGCGGTGCTGCCGTCCCGAGGTCAAGTCCCGGGGGAGGAGAAGAGCGGCTTCCCG-CCTAGAGCATTTGCAAGTCAGGATTCTCTAATCCCTCTGGGAGAAGGGTATTCGGCTTGTCCGCTATGTTTT

>MK540396 Human gammaherpesvirus 4 isolate NPCT042

AGGACAGCCGTTGCCCTAGTGGTTTCGGACACACCGCCAACGCGCTGTGCGGTGCTGCCGTCCCGAGGTCAAGTCCCGGGGGAGGAGAAGAGCGGCTTCCCG-CCTAGAGCATTTGCAAGTCAGGATTCTCTAATCCCTCTGGGAGAAGGGTATTCGGCTTGTCCGCTATGTTTT

>MK540398 Human gammaherpesvirus 4 isolate NPCT045

AGGACAGCCGTTGCCCTAGTGGTTTCGGACACACCGCCAACGCGCTGTGCGGTGCTGCCGTCCCGAGGTCAAGTCCCGGGGGAGGAGAAGAGCGGCTTCCCG-CCTAGAGCATTTGCAAGTCAGGATTCTCTAATCCCTCTGGGAGAAGGGTATTCGGCTTGTCCGCTATGTTTT

>MK540399 Human gammaherpesvirus 4 isolate NPCT046

AGGACAGCCGTTGCCCTAGTGGTTTCGGACACACCGCCAACGCGCTGTGCGGTGCTGCCGTCCCGAGGTCAAGTCCCGGGGGAGGAGAAGAGCGGCTTCCCG-CCTAGAGCATTTGCAAGTCAGGATTCTCTAATCCCTCTGGGAGAAGGGTATTCGGCTTGTCCGCTATGTTTT

>MK540401 Human gammaherpesvirus 4 isolate NPCT048

AGGACAGCCGTTGCCCTAGTGGTTTCGGACACACCGCCAACGCGCTGTGCGGTGCTGCCGTCCCGAGGTCAAGTCCCGGGGGAGGAGAAGAGCGGCTTCCCG-CCTAGAGCATTTGCAAGTCAGGATTCTCTAATCCCTCTGGGAGAAGGGTATTCGGCTTGTCCGCTATGTTTT

>MK540403 Human gammaherpesvirus 4 isolate NPCT050

AGGACAGCCGTTGCCCTAGTGGTTTCGGACACACCGCCAACGCGCTGTGCGGTGCTGCCGTCCCGAGGTCAAGTCCCGGGGGAGGAGAAGAGCGGCTTCCCG-CCTAGAGCATTTGCAAGTCAGGATTCTCTAATCCCTCTGGGAGAAGGGTATTCGGCTTGTCCGCTATGTTTT

>MK540404 Human gammaherpesvirus 4 isolate NPCT052

AGGACAGCCGTTGCCCTAGTGGTTTCGGACACACCGCCAACGCGCTGTGCGGTGCTGCCGTCCCGAGGTCAAGTCCCGGGGGAGGAGAAGAGCGGCTTCCCG-CCTAGAGCATTTGCAAGTCAGGATTCTCTAATCCCTCTGGGAGAAGGGTATTCGGCTTGTCCGCTATGTTTT

>MK540405 Human gammaherpesvirus 4 isolate NPCT053

AGGACAGCCGTTGCCCTAGTGGTTTCGGACACACCGCCAACGCGCTGTGCGGTGCTGCCGTCCCGAGGTCAAGTCCCGGGGGAGGAGAAGAGCGGCTTCCCG-CCTAGAGCATTTGCAAGTCAGGATTCTCTAATCCCTCTGGGAGAAGGGTATTCGGCTTGTCCGCTATGTTTT

>MK540406 Human gammaherpesvirus 4 isolate NPCT054

AGGACAGCCGTTGCCCTAGTGGTTTCGGACACACCGCCAACGCGCTGTGCGGTGCTGCCGTCCCGAGGTCAAGTCCCGGGGGAGGAGAAGAGCGGCTTCCCG-CCTAGAGCATTTGCAAGTCAGGATTCTCTAATCCCTCTGGGAGAAGGGTATTCGGCTTGTCCGCTATGTTTT

>MK540408 Human gammaherpesvirus 4 isolate NPCT055

AGGACAGCCGTTGCCCTAGTGGTTTCGGACACACCGCCAACGCGCTGTGCGGTGCTGCCGTCCCGAGGTCAAGTCCCGGGGGAGGAGAAGAGCGGCTTCCCG-CCTAGAGCATTTGCAAGTCAGGATTCTCTAATCCCTCTGGGAGAAGGGTATTCGGCTTGTCCGCTATGTTTT

>MK540410 Human gammaherpesvirus 4 isolate NPCT056

AGGACAGCCGTTGCCCTAGTGGTTTCGGACACACCGCCAACGCGCTGTGCGGTGCTGCCGTCCCGAGGTCAAGTCCCGGGGGAGGAGAAGAGCGGCTTCCCG-CCTAGAGCATTTGCAAGTCAGGATTCTCTAATCCCTCTGGGAGAAGGGTATTCGGCTTGTCCGCTATGTTTT

>MK540412 Human gammaherpesvirus 4 isolate NPCT057

AGGACAGCCGTTGCCCTAGTGGTTTCGGACACACCGCCAACGCGCTGTGCGGTGCTGCCGTCCCGAGGTCAAGTCCCGGGGGAGGAGAAGAGCGGCTTCCCG-CCTAGAGCATTTGCAAGTCAGGATTCTCTAATCCCTCTGGGAGAAGGGTATTCGGCTTGTCCGCTATGTTTT

>MK540414 Human gammaherpesvirus 4 isolate NPCT058

AGGACAGCCGTTGCCCTAGTGGTTTCGGACACACCGCCAACGCGCTGTGCGGTGCTGCCGTCCCGAGGTCAAGTCCCGGGGGAGGAGAAGAGCGGCTTCCCG-CCTAGAGCATTTGCAAGTCAGGATTCTCTAATCCCTCTGGGAGAAGGGTATTCGGCTTGTCCGCTATGTTTT

>MK540416 Human gammaherpesvirus 4 isolate NPCT059

AGGACAGCCGTTGCCCTAGTGGTTTCGGACACACCGCCAACGCGCTGTGCGGTGCTGCCGTCCCGAGGTCAAGTCCCGGGGGAGGAGAAGAGCGGCTTCCCG-CCTAGAGCATTTGCAAGTCAGGATTCTCTAATCCCTCTGGGAGAAGGGTATTCGGCTTGTCCGCTATGTTTT

>MK540420 Human gammaherpesvirus 4 isolate NPCT063

AGGACAGCCGTTGCCCTAGTGGTTTCGGACACACCGCCAACGCGCTGTGCGGTGCTGCCGTCCCGAGGTCAAGTCCCGGGGGAGGAGAAGAGCGGCTTCCCG-CCTAGAGCATTTGCAAGTCAGGATTCTCTAATCCCTCTGGGAGAAGGGTATTCGGCTTGTCCGCTATGTTTT

>MK540421 Human gammaherpesvirus 4 isolate NPCT064

AGGACAGCCGTTGCCCTAGTGGTTTCGGACACACCGCCAACGCGCTGTGCGGTGCTGCCGTCCCGAGGTCAAGTCCCGGGGGAGGAGAAGAGCGGCTTCCCG-CCTAGAGCATTTGCAAGTCAGGATTCTCTAATCCCTCTGGGAGAAGGGTATTCGGCTTGTCCGCTATGTTTT

>MK540422 Human gammaherpesvirus 4 isolate NPCT065

AGGACAGCCGTTGCCCTAGTGGTTTCGGACACACCGCCAACGCGCTGTGCGGTGCTGCCGTCCCGAGGTCAAGTCCCGGGGGAGGAGAAGAGCGGCTTCCCG-CCTAGAGCATTTGCAAGTCAGGATTCTCTAATCCCTCTGGGAGAAGGGTATTCGGCTTGTCCGCTATGTTTT

>MK540423 Human gammaherpesvirus 4 isolate NPCT066

AGGACAGCCGTTGCCCTAGTGGTTTCGGACACACCGCCAACGCGCTGTGCGGTGCTGCCGTCCCGAGGTCAAGTCCCGGGGGAGGAGAAGAGCGGCTTCCCG-CCTAGAGCATTTGCAAGTCAGGATTCTCTAATCCCTCTGGGAGAAGGGTATTCGGCTTGTCCGCTATGTTTT

>MK540424 Human gammaherpesvirus 4 isolate NPCT067

AGGACAGCCGTTGCCCTAGTGGTTTCGGACACACCGCCAACGCGCTGTGCGGTGCTGCCGTCCCGAGGTCAAGTCCCGGGGGAGGAGAAGAGCGGCTTCCCG-CCTAGAGCATTTGCAAGTCAGGATTCTCTAATCCCTCTGGGAGAAGGGTATTCGGCTTGTCCGCTATGTTTT

>MK540425 Human gammaherpesvirus 4 isolate NPCT068

AGGACAGCCGTTGCCCTAGTGGTTTCGGACACACCGCCAACGCGCTGTGCGGTGCTGCCGTCCCGAGGTCAAGTCCCGGGGGAGGAGAAGAGCGGCTTCCCG-CCTAGAGCATTTGCAAGTCAGGATTCTCTAATCCCTCTGGGAGAAGGGTATTCGGCTTGTCCGCTATGTTTT

>MK540426 Human gammaherpesvirus 4 isolate NPCT069

AGGACAGCCGTTGCCCTAGTGGTTTCGGACACACCGCCAACGCGCTGTGCGGTGCTGCCGTCCCGAGGTCAAGTCCCGGGGGAGGAGAAGAGCGGCTTCCCG-CCTAGAGCATTTGCAAGTCAGGATTCTCTAATCCCTCTGGGAGAAGGGTATTCGGCTTGTCCGCTATGTTTT

>MK540427 Human gammaherpesvirus 4 isolate NPCT070

AGGACAGCCGTTGCCCTAGTGGTTTCGGACACACCGCCAACGCGCTGTGCGGTGCTGCCGTCCCGAGGTCAAGTCCCGGGGGAGGAGAAGAGCGGCTTCCCG-CCTAGAGCATTTGCAAGTCAGGATTCTCTAATCCCTCTGGGAGAAGGGTATTCGGCTTGTCCGCTATGTTTT

>MK540428 Human gammaherpesvirus 4 isolate NPCT071

AGGACAGCCGTTGCCCTAGTGGTTTCGGACACACCGCCAACGCGCTGTGCGGTGCTGCCGTCCCGAGGTCAAGTCCCGGGGGAGGAGAAGAGCGGCTTCCCG-CCTAGAGCATTTGCAAGTCAGGATTCTCTAATCCCTCTGGGAGAAGGGTATTCGGCTTGTCCGCTATGTTTT

>MK540429 Human gammaherpesvirus 4 isolate NPCT072

AGGACAGCCGTTGCCCTAGTGGTTTCGGACACACCGCCAACGCGCTGTGCGGTGCTGCCGTCCCGAGGTCAAGTCCCGGGGGAGGAGAAGAGCGGCTTCCCG-CCTAGAGCATTTGCAAGTCAGGATTCTCTAATCCCTCTGGGAGAAGGGTATTCGGCTTGTCCGCTATGTTTT

>MK540430 Human gammaherpesvirus 4 isolate NPCT073

AGGACAGCCGTTGCCCTAGTGGTTTCGGACACACCGCCAACGCGCTGTGCGGTGCTGCCGTCCCGAGGTCAAGTCCCGGGGGAGGAGAAGAGCGGCTTCCCG-CCTAGAGCATTTGCAAGTCAGGATTCTCTAATCCCTCTGGGAGAAGGGTATTCGGCTTGTCCGCTATGTTTT

>MK540431 Human gammaherpesvirus 4 isolate NPCT074

AGGACAGCCGTTGCCCTAGTGGTTTCGGACACACCGCCAACGCGCTGTGCGGTGCTGCCGTCCCGAGGTCAAGTCCCGGGGGAGGAGAAGAGCGGCTTCCCG-CCTAGAGCATTTGCAAGTCAGGATTCTCTAATCCCTCTGGGAGAAGGGTATTCGGCTTGTCCGCTATGTTTT

>MK540433 Human gammaherpesvirus 4 isolate NPCT075

AGGACAGCCGTTGCCCTAGTGGTTTCGGACACACCGCCAACGCGCTGTGCGGTGCTGCCGTCCCGAGGTCAAGTCCCGGGGGAGGAGAAGAGCGGCTTCCCG-CCTAGAGCATTTGCAAGTCAGGATTCTCTAATCCCTCTGGGAGAAGGGTATTCGGCTTGTCCGCTATGTTTT

>MK540434 Human gammaherpesvirus 4 isolate NPCT076

AGGACAGCCGTTGCCCTAGTGGTTTCGGACACACCGCCAACGCGCTGTGCGGTGCTGCCGTCCCGAGGTCAAGTCCCGGGGGAGGAGAAGAGCGGCTTCCCG-CCTAGAGCATTTGCAAGTCAGGATTCTCTAATCCCTCTGGGAGAAGGGTATTCGGCTTGTCCGCTATGTTTT

>MK540435 Human gammaherpesvirus 4 isolate NPCT077

AGGACAGCCGTTGCCCTAGTGGTTTCGGACACACCGCCAACGCGCTGTGCGGTGCTGCCGTCCCGAGGTCAAGTCCCGGGGGAGGAGAAGAGCGGCTTCCCG-CCTAGAGCATTTGCAAGTCAGGATTCTCTAATCCCTCTGGGAGAAGGGTATTCGGCTTGTCCGCTATGTTTT

>MK540436 Human gammaherpesvirus 4 isolate NPCT078

AGGACAGCCGTTGCCCTAGTGGTTTCGGACACACCGCCAACGCGCTGTGCGGTGCTGCCGTCCCGAGGTCAAGTCCCGGGGGAGGAGAAGAGCGGCTTCCCG-CCTAGAGCATTTGCAAGTCAGGATTCTCTAATCCCTCTGGGAGAAGGGTATTCGGCTTGTCCGCTATGTTTT

>MK540437 Human gammaherpesvirus 4 isolate NPCT080

AGGACAGCCGTTGCCCTAGTGGTTTCGGACACACCGCCAACGCGCTGTGCGGTGCTGCCGTCCCGAGGTCAAGTCCCGGGGGAGGAGAAGAGCGGCTTCCCG-CCTAGAGCATTTGCAAGTCAGGATTCTCTAATCCCTCTGGGAGAAGGGTATTCGGCTTGTCCGCTATGTTTT

>MK540438 Human gammaherpesvirus 4 isolate NPCT081

AGGACAGCCGTTGCCCTAGTGGTTTCGGACACACCGCCAACGCGCTGTGCGGTGCTGCCGTCCCGAGGTCAAGTCCCGGGGGAGGAGAAGAGCGGCTTCCCG-CCTAGAGCATTTGCAAGTCAGGATTCTCTAATCCCTCTGGGAGAAGGGTATTCGGCTTGTCCGCTATGTTTT

>MK540439 Human gammaherpesvirus 4 isolate NPCT082

AGGACAGCCGTTGCCCTAGTGGTTTCGGACACACCGCCAACGCGCTGTGCGGTGCTGCCGTCCCGAGGTCAAGTCCCGGGGGAGGAGAAGAGCGGCTTCCCG-CCTAGAGCATTTGCAAGTCAGGATTCTCTAATCCCTCTGGGAGAAGGGTATTCGGCTTGTCCGCTATGTTTT

>MK540440 Human gammaherpesvirus 4 isolate NPCT083

AGGACAGCCGTTGCCCTAGTGGTTTCGGACACACCGCCAACGCGCTGTGCGGTGCTGCCGTCCCGAGGTCAAGTCCCGGGGGAGGAGAAGAGCGGCTTCCCG-CCTAGAGCATTTGCAAGTCAGGATTCTCTAATCCCTCTGGGAGAAGGGTATTCGGCTTGTCCGCTATGTTTT

>MK540441 Human gammaherpesvirus 4 isolate NPCT084

AGGACAGCCGTTGCCCTAGTGGTTTCGGACACACCGCCAACGCGCTGTGCGGTGCTGCCGTCCCGAGGTCAAGTCCCGGGGGAGGAGAAGAGCGGCTTCCCG-CCTAGAGCATTTGCAAGTCAGGATTCTCTAATCCCTCTGGGAGAAGGGTATTCGGCTTGTCCGCTATGTTTT

>MK540442 Human gammaherpesvirus 4 isolate NPCT085

AGGACAGCCGTTGCCCTAGTGGTTTCGGACACACCGCCAACGCGCTGTGCGGTGCTGCCGTCCCGAGGTCAAGTCCCGGGGGAGGAGAAGAGCGGCTTCCCG-CCTAGAGCATTTGCAAGTCAGGATTCTCTAATCCCTCTGGGAGAAGGGTATTCGGCTTGTCCGCTATGTTTT

>MK540443 Human gammaherpesvirus 4 isolate NPCT086

AGGACAGCCGTTGCCCTAGTGGTTTCGGACACACCGCCAACGCGCTGTGCGGTGCTGCCGTCCCGAGGTCAAGTCCCGGGGGAGGAGAAGAGCGGCTTCCCG-CCTAGAGCATTTGCAAGTCAGGATTCTCTAATCCCTCTGGGAGAAGGGTATTCGGCTTGTCCGCTATGTTTT

>MK540444 Human gammaherpesvirus 4 isolate NPCT087

AGGACAGCCGTTGCCCTAGTGGTTTCGGACACACCGCCAACGCGCTGTGCGGTGCTGCCGTCCCGAGGTCAAGTCCCGGGGGAGGAGAAGAGCGGCTTCCCG-CCTAGAGCATTTGCAAGTCAGGATTCTCTAATCCCTCTGGGAGAAGGGTATTCGGCTTGTCCGCTATGTTTT

>MK540445 Human gammaherpesvirus 4 isolate NPCT088

AGGACAGCCGTTGCCCTAGTGGTTTCGGACACACCGCCAACGCGCTGTGCGGTGCTGCCGTCCCGAGGTCAAGTCCCGGGGGAGGAGAAGAGCGGCTTCCCG-CCTAGAGCATTTGCAAGTCAGGATTCTCTAATCCCTCTGGGAGAAGGGTATTCGGCTTGTCCGCTATGTTTT

>MK540447 Human gammaherpesvirus 4 isolate NPCT090

AGGACAGCCGTTGCCCTAGTGGTTTCGGACACACCGCCAACGCGCTGTGCGGTGCTGCCGTCCCGAGGTCAAGTCCCGGGGGAGGAGAAGAGCGGCTTCCCG-CCTAGAGCATTTGCAAGTCAGGATTCTCTAATCCCTCTGGGAGAAGGGTATTCGGCTTGTCCGCTATGTTTT

>MK540448 Human gammaherpesvirus 4 isolate NPCT091

AGGACAGCCGTTGCCCTAGTGGTTTCGGACACACCGCCAACGCGCTGTGCGGTGCTGCCGTCCCGAGGTCAAGTCCCGGGGGAGGAGAAGAGCGGCTTCCCG-CCTAGAGCATTTGCAAGTCAGGATTCTCTAATCCCTCTGGGAGAAGGGTATTCGGCTTGTCCGCTATGTTTT

>MK540449 Human gammaherpesvirus 4 isolate NPCT092

AGGACAGCCGTTGCCCTAGTGGTTTCGGACACACCGCCAACGCGCTGTGCGGTGCTGCCGTCCCGAGGTCAAGTCCCGGGGGAGGAGAAGAGCGGCTTCCCG-CCTAGAGCATTTGCAAGTCAGGATTCTCTAATCCCTCTGGGAGAAGGGTATTCGGCTTGTCCGCTATGTTTT

>MK540450 Human gammaherpesvirus 4 isolate NPCT093

AGGACAGCCGTTGCCCTAGTGGTTTCGGACACACCGCCAACGCGCTGTGCGGTGCTGCCGTCCCGAGGTCAAGTCCCGGGGGAGGAGAAGAGCGGCTTCCCG-CCTAGAGCATTTGCAAGTCAGGATTCTCTAATCCCTCTGGGAGAAGGGTATTCGGCTTGTCCGCTATGTTTT

>MK540451 Human gammaherpesvirus 4 isolate NPCT094

AGGACAGCCGTTGCCCTAGTGGTTTCGGACACACCGCCAACGCGCTGTGCGGTGCTGCCGTCCCGAGGTCAAGTCCCGGGGGAGGAGAAGAGCGGCTTCCCG-CCTAGAGCATTTGCAAGTCAGGATTCTCTAATCCCTCTGGGAGAAGGGTATTCGGCTTGTCCGCTATGTTTT

>MK540452 Human gammaherpesvirus 4 isolate NPCT096

AGGACAGCCGTTGCCCTAGTGGTTTCGGACACACCGCCAACGCGCTGTGCGGTGCTGCCGTCCCGAGGTCAAGTCCCGGGGGAGGAGAAGAGCGGCTTCCCG-CCTAGAGCATTTGCAAGTCAGGATTCTCTAATCCCTCTGGGAGAAGGGTATTCGGCTTGTCCGCTATGTTTT

>MK540453 Human gammaherpesvirus 4 isolate NPCT098

AGGACAGCCGTTGCCCTAGTGGTTTCGGACACACCGCCAACGCGCTGTGCGGTGCTGCCGTCCCGAGGTCAAGTCCCGGGGGAGGAGAAGAGCGGCTTCCCG-CCTAGAGCATTTGCAAGTCAGGATTCTCTAATCCCTCTGGGAGAAGGGTATTCGGCTTGTCCGCTATGTTTT

>MK540454 Human gammaherpesvirus 4 isolate NPCT099

AGGACAGCCGTTGCCCTAGTGGTTTCGGACACACCGCCAACGCGCTGTGCGGTGCTGCCGTCCCGAGGTCAAGTCCCGGGGGAGGAGAAGAGCGGCTTCCCG-CCTAGAGCATTTGCAAGTCAGGATTCTCTAATCCCTCTGGGAGAAGGGTATTCGGCTTGTCCGCTATGTTTT

>MK540455 Human gammaherpesvirus 4 isolate NPCT100

AGGACAGCCGTTGCCCTAGTGGTTTCGGACACACCGCCAACGCGCTGTGCGGTGCTGCCGTCCCGAGGTCAAGTCCCGGGGGAGGAGAAGAGCGGCTTCCCG-CCTAGAGCATTTGCAAGTCAGGATTCTCTAATCCCTCTGGGAGAAGGGTATTCGGCTTGTCCGCTATGTTTT

>MK540456 Human gammaherpesvirus 4 isolate NPCT101

AGGACAGCCGTTGCCCTAGTGGTTTCGGACACACCGCCAACGCGCTGTGCGGTGCTGCCGTCCCGAGGTCAAGTCCCGGGGGAGGAGAAGAGCGGCTTCCCG-CCTAGAGCATTTGCAAGTCAGGATTCTCTAATCCCTCTGGGAGAAGGGTATTCGGCTTGTCCGCTATGTTTT

>MK540457 Human gammaherpesvirus 4 isolate NPCT102

AGGACAGCCGTTGCCCTAGTGGTTTCGGACACACCGCCAACGCGCTGTGCGGTGCTGCCGTCCCGAGGTCAAGTCCCGGGGGAGGAGAAGAGCGGCTTCCCG-CCTAGAGCATTTGCAAGTCAGGATTCTCTAATCCCTCTGGGAGAAGGGTATTCGGCTTGTCCGCTATGTTTT

>MK540458 Human gammaherpesvirus 4 isolate NPCT103

AGGACAGCCGTTGCCCTAGTGGTTTCGGACACACCGCCAACGCGCTGTGCGGTGCTGCCGTCCCGAGGTCAAGTCCCGGGGGAGGAGAAGAGCGGCTTCCCG-CCTAGAGCATTTGCAAGTCAGGATTCTCTAATCCCTCTGGGAGAAGGGTATTCGGCTTGTCCGCTATGTTTT

>MK540459 Human gammaherpesvirus 4 isolate NPCT104

AGGACAGCCGTTGCCCTAGTGGTTTCGGACACACCGCCAACGCGCTGTGCGGTGCTGCCGTCCCGAGGTCAAGTCCCGGGGGAGGAGAAGAGCGGCTTCCCG-CCTAGAGCATTTGCAAGTCAGGATTCTCTAATCCCTCTGGGAGAAGGGTATTCGGCTTGTCCGCTATGTTTT

>MK540460 Human gammaherpesvirus 4 isolate NPCT105

AGGACAGCCGTTGCCCTAGTGGTTTCGGACACACCGCCAACGCGCTGTGCGGTGCTGCCGTCCCGAGGTCAAGTCCCGGGGGAGGAGAAGAGCGGCTTCCCG-CCTAGAGCATTTGCAAGTCAGGATTCTCTAATCCCTCTGGGAGAAGGGTATTCGGCTTGTCCGCTATGTTTT

>MK540461 Human gammaherpesvirus 4 isolate NPCT106

AGGACAGCCGTTGCCCTAGTGGTTTCGGACACACCGCCAACGCGCTGTGCGGTGCTGCCGTCCCGAGGTCAAGTCCCGGGGGAGGAGAAGAGCGGCTTCCCG-CCTAGAGCATTTGCAAGTCAGGATTCTCTAATCCCTCTGGGAGAAGGGTATTCGGCTTGTCCGCTATGTTTT

>MK540462 Human gammaherpesvirus 4 isolate NPCT107

AGGACAGCCGTTGCCCTAGTGGTTTCGGACACACCGCCAACGCGCTGTGCGGTGCTGCCGTCCCGAGGTCAAGTCCCGGGGGAGGAGAAGAGCGGCTTCCCG-CCTAGAGCATTTGCAAGTCAGGATTCTCTAATCCCTCTGGGAGAAGGGTATTCGGCTTGTCCGCTATGTTTT

>MK540463 Human gammaherpesvirus 4 isolate NPCT108

AGGACAGCCGTTGCCCTAGTGGTTTCGGACACACCGCCAACGCGCTGTGCGGTGCTGCCGTCCCGAGGTCAAGTCCCGGGGGAGGAGAAGAGCGGCTTCCCG-CCTAGAGCATTTGCAAGTCAGGATTCTCTAATCCCTCTGGGAGAAGGGTATTCGGCTTGTCCGCTATGTTTT

>MK540464 Human gammaherpesvirus 4 isolate NPCT109

AGGACAGCCGTTGCCCTAGTGGTTTCGGACACACCGCCAACGCGCTGTGCGGTGCTGCCGTCCCGAGGTCAAGTCCCGGGGGAGGAGAAGAGCGGCTTCCCG-CCTAGAGCATTTGCAAGTCAGGATTCTCTAATCCCTCTGGGAGAAGGGTATTCGGCTTGTCCGCTATGTTTT

>MK540465 Human gammaherpesvirus 4 isolate NPCT110

AGGACAGCCGTTGCCCTAGTGGTTTCGGACACACCGCCAACGCGCTGTGCGGTGCTGCCGTCCCGAGGTCAAGTCCCGGGGGAGGAGAAGAGCGGCTTCCCG-CCTAGAGCATTTGCAAGTCAGGATTCTCTAATCCCTCTGGGAGAAGGGTATTCGGCTTGTCCGCTATGTTTT

>MK540466 Human gammaherpesvirus 4 isolate NPCT111

AGGACAGCCGTTGCCCTAGTGGTTTCGGACACACCGCCAACGCGCTGTGCGGTGCTGCCGTCCCGAGGTCAAGTCCCGGGGGAGGAGAAGAGCGGCTTCCCG-CCTAGAGCATTTGCAAGTCAGGATTCTCTAATCCCTCTGGGAGAAGGGTATTCGGCTTGTCCGCTATGTTTT

>MK540467 Human gammaherpesvirus 4 isolate NPCT112

AGGACAGCCGTTGCCCTAGTGGTTTCGGACACACCGCCAACGCGCTGTGCGGTGCTGCCGTCCCGAGGTCAAGTCCCGGGGGAGGAGAAGAGCGGCTTCCCG-CCTAGAGCATTTGCAAGTCAGGATTCTCTAATCCCTCTGGGAGAAGGGTATTCGGCTTGTCCGCTATGTTTT

>MK540468 Human gammaherpesvirus 4 isolate NPCT113

AGGACAGCCGTTGCCCTAGTGGTTTCGGACACACCGCCAACGCGCTGTGCGGTGCTGCCGTCCCGAGGTCAAGTCCCGGGGGAGGAGAAGAGCGGCTTCCCG-CCTAGAGCATTTGCAAGTCAGGATTCTCTAATCCCTCTGGGAGAAGGGTATTCGGCTTGTCCGCTATGTTTT

>MK540469 Human gammaherpesvirus 4 isolate NPCT114

AGGACAGCCGTTGCCCTAGTGGTTTCGGACACACCGCCAACGCGCTGTGCGGTGCTGCCGTCCCGAGGTCAAGTCCCGGGGGAGGAGAAGAGCGGCTTCCCG-CCTAGAGCATTTGCAAGTCAGGATTCTCTAATCCCTCTGGGAGAAGGGTATTCGGCTTGTCCGCTATGTTTT

>MK540470 Human gammaherpesvirus 4 isolate NPCT115

AGGACAGCCGTTGCCCTAGTGGTTTCGGACACACCGCCAACGCGCTGTGCGGTGCTGCCGTCCCGAGGTCAAGTCCCGGGGGAGGAGAAGAGCGGCTTCCCG-CCTAGAGCATTTGCAAGTCAGGATTCTCTAATCCCTCTGGGAGAAGGGTATTCGGCTTGTCCGCTATGTTTT

>AB850659 Human herpesvirus 4 DNA, complete genome, strain: HN9

AGGACAGCCGTTGCCCTAGTGGTTTCGGACACACCGCCAACGCGCTGTGCGGTGCTGCCGTCCCGAGGTCAAGTCCCGGGGGAGGAGAAGAGCGGCTTCCCG-CCTAGAGCATTTGCAAGTCAGGATTCTCTAATCCCTCTGGGAGAAGGGTATTCGGCTTGTCCGCTGT-TTT-

>AB850648 Human herpesvirus 4 DNA, complete genome, strain: HN3

AGGACAGCCGTTGCCCTAGTGGTTTCGGACACACCGCCAACGCGCTGTGCGGTGCTGCCGTCCCGAGGTCAAGTCCCGGGGGAGGAGAAGAGCGGCTTCCCG-CCTAGAGCATTTGCAAGTCAGGATTCTCTAATCCCTCTGGGAAAAGGGTATTCGGCTTGTCCGCTGT-TTTT

>KP195394 Human herpesvirus 4 isolate GDNPC13 EBER1 and EBER2 genes, complete sequence

AGGACAGCCGTTGCCCTAGTGGTTTCGGACACACCGCCAACGCGCTGTGCGGTGCTGCCGTCCCGAGTTCAAGTCCCGGGGGAGGAGAAGAGCGGCTTCCCG-CCTAGAGCATTTGCAAGTCAGGATTCTCTAATCCCTCTGGGAGAAGGGTATTCGGCTTGTCCGCTGT-TTTT

>KP195397 Human herpesvirus 4 isolate GDNPC16 EBER1 and EBER2 genes, complete sequence

AGGACAGCCGTTGCCCTAGTGGTTTCGGACACACCGCCAACGCGCTGTGCGGTGCTGCCGTCCCGAGGTCGAGTCCCGGGGGAGGAGAAGAGCGGCTTCCCG-CCTAGAGCATTTGCAAGTCAGGATTCTCTAATCCCTCTGGGAGAAGGGTATTCGGCTTGTCCGCTGT-TTTT

>KP195406 Human herpesvirus 4 isolate GDNPC25 EBER1 and EBER2 genes, complete sequence

AGGACAGCCGTTGCCCTAGTGGTTTCGGAGACACCGCCAACGCGCTGTGCGGTGCTGCCGTCCCGAGGTCAAGTCCCGGGGGAGGAGAAGAGCGGCTTCCCG-CCTAGAGCATTTGCAAGTCAGGATTCTCTAATCCCTCTGGGAGAAGGGTATTCGGCTTGTCCGCTGT-TTTT

>KP195483 Human herpesvirus 4 isolate SDNPC64 EBER1 and EBER2 genes, complete sequence

AGGACAGCCGTTGCCCTAGTGGTTTCGGACACACCGCCAAAGCGCTGTGCGGTGCTGCCGTCCCGAGGTCAAGTCCCGGGGGAGGAGAAGAGCGGCTTCCCG-CCTAGAGCATTTGCAAGTCAGGATTCTCTAATCCCTCTGGGAGAAGGGTATTCGGCTTGTCCGCTGT-TTTT

>KP195547 Human herpesvirus 4 isolate GDTW28 EBER1 and EBER2 genes, complete sequence

AGGACAGCCGTTGCCCTAGTGGTTTCGGACACACCGCCAACGCGCTGTGCGGTGCTGCCGTCCCGAGGTCAAGTCCCGCGGGAGGAGAAGAGCGGCTTCCCG-CCTAGAGCATTTGCAAGTCAGGATTCTCTAATCCCTCTGGGAGAAGGGTATTCGGCTTGTCCGCTGT-TTTT

>KP195606 Human herpesvirus 4 isolate GDTW148 EBER1 and EBER2 genes, complete sequence

AGGACAGCCGTTGCCCTAGTGGTTTCGGACACACCGCCAACGCGCTGTGCGGTGCTGCCGTCACGAGGTCAAGTCCCGGGGGAGGAGAAGAGCGGCTTCCCG-CCTAGAGCATTTGCAAGTCAGGATTCTCTAATCCCTCTGGGAGAAGGGTATTCGGCTTGTCCGCTGT-TTTT

>MG298883 Human gammaherpesvirus 4 isolate JM_NPC_bru_178

AGGACAGCCGTTGCCCTAGTGGTTTCGGACACACCGCCAACGCGCTGTGCGGTGCTGCCGTCCCGAGGTCAAGTCCCGGGGGAGGAGAAAAGCGGCTTCCCG-CCTAGAGCATTTGCAAGTCAGGATTCTCTAATCCCTCTGGGAGAAGGGTATTCGGCTTGTCCGCTGT-TTTT

>MG298888 Human gammaherpesvirus 4 isolate JM_NPC_bru_L16

AGGACAGCCGTTGCCCTAGTGGTTTCGGACACACCGCCAACGCGCTGTGCGGTGCTGCCGTCCCGAGGTCAAGTCCCGGGGGAGGAGAAAAGCGGCTTCCCG-CCTAGAGCATTTGCAAGTCAGGATTCTCTAATCCCTCTGGGAGAAGGGTATTCGGCTTGTCCGCTGT-TTTT

>MG298897 Human gammaherpesvirus 4 isolate JM_NPC_bru_L5

AGGACAGCCGTTGCCCTAGTGGTTTCGGACACACCGCCAACGCGCTGTGCGGTGCTGCCGTCCCGAGGTCAAGTCCCGGGGGAGGAGAAAAGCGGCTTCCCG-CCTAGAGCATTTGCAAGTCAGGATTCTCTAATCCCTCTGGGAGAAGGGTATTCGGCTTGTCCGCTGT-TTTT

>MH590375 Human gammaherpesvirus 4 isolate HKHD6

AGGACAGCCGTTGCCCTAGTGGTTTCGTACACACCGCCAACGCGCTGTGCGGTGCTGCCGTCCCGAGGTCAAGTCCCGGGGGAGGAGAAGAGCGGCTTCCCG-CCTAGAGCATTTGCAAGTCAGGATTCTCTAATCCCTCTGGGAGAAGGGTATTCGGCTTGTCCGCTGT-TTTT

>MH590395 Human gammaherpesvirus 4 isolate HKHD26

AGGACAGCCGTTGCCCTAGTGGTTTCGGACACACCGCCAACGCGCTGTGCGGTGCTGCCGTCCCGAGGTCAAGTCCCGGGGAAGGAGAAGAGCGGCTTCCCG-CCTAGAGCATTTGCAAGTCAGGATTCTCTAATCCCTCTGGGAGAAGGGTATTCGGCTTGTCCGCTGT-TTTT

>MH590417 Human gammaherpesvirus 4 isolate HKHD48

AGGACAGCCGTTGCCCTAGTGGTTTCAGACACACCGCCAACGCGCTGTGCGGTGCTGCCGTCCCGAGGTCAAGTCCCGGGGGAGGAGAAGAGCGGCTTCCCG-CCTAGAGCATTTGCAAGTCAGGATTCTCTAATCCCTCTGGGAGAAGGGTATTCGGCTTGTCCGCTGT-TTTT

>MH590551 Human gammaherpesvirus 4 isolate HKNPC40

AGGACAGCCGTTGCCCTAGTGGTTTGGGACACACCGCCAACGCGCTGTGCGGTGCTGCCGTCCCGAGGTCAAGTCCCGGGGGAGGAGAAGAGCGGCTTCCCG-CCTAGAGCATTTGCAAGTCAGGATTCTCTAATCCCTCTGGGAGAAGGGTATTCGGCTTGTCCGCTGT-TTTT

>MK540281 Human gammaherpesvirus 4 isolate HS025

AGGACAGCCGTTGCCCTAGTGGTTTCGGACACACCGCCAACGCGCTGTGCGGTGCTGCCGTCCCGAGGTCAAGTCCCGGGGGAGGAGAAGAGCGGCTTCCCG-CCTAGAGCATTTGCAAGTCAGGATTCTCTAATCCCTCTGGGAAAAGGGTATTCGGCTTGTCCGCTATGTTTT

>MK540340 Human gammaherpesvirus 4 isolate NPCS030

AGGACAGCCGTTGCCCTAGTGGTTTCGGACACAGCGCCAACGCGCTGTGCGGTGCTGCCGTCCCGAGGTCAAGTCCCGGGGGAGGAGAAGAGCGGCTTCCCG-CCTAGAGCATTTGCAAGTCAGGATTCTCTAATCCCTCTGGGAGAAGGGTATTCGGCTTGTCCGCTATGTTTT

>MK540344 Human gammaherpesvirus 4 isolate NPCS035

AGGACAGCCGTTGCCCTAGTGGTTTCGGACACACCGCCAACGCGCTGTGCCGTGCTGCCGTCCCGAGGTCAAGTCCCGGGGGAGGAGAAGAGCGGCTTCCCG-CCTAGAGCATTTGCAAGTCAGGATTCTCTAATCCCTCTGGGAGAAGGGTATTCGGCTTGTCCGCTATGTTTT

>MK540348 Human gammaherpesvirus 4 isolate NPCS042

AGGACAGCCGTTGCCCTAGTGGTTTCGGATACACCGCCAACGCGCTGTGCGGTGCTGCCGTCCCGAGGTCAAGTCCCGGGGGAGGAGAAGAGCGGCTTCCCG-CCTAGAGCATTTGCAAGTCAGGATTCTCTAATCCCTCTGGGAGAAGGGTATTCGGCTTGTCCGCTATGTTTT

>MK540372 Human gammaherpesvirus 4 isolate NPCT014

AGGACAGCCGTTGCCCTAGTGGTTTCGGACACACCGCCAACGCGCTGTGCGGTGCTGCCGTCCCGAGGTCGAGTCCCGGGGGAGGAGAAGAGCGGCTTCCCG-CCTAGAGCATTTGCAAGTCAGGATTCTCTAATCCCTCTGGGAGAAGGGTATTCGGCTTGTCCGCTATGTTTT

>MK540384 Human gammaherpesvirus 4 isolate NPCT028-2

AGGACAGCCGTTGCCCTAGTGGTTTCGGACACACCGCCAACGCGCTGTGCGGTGCTGCCGTCCCGAGTTCAAGTCCCGGGGGAGGAGAAGAGCGGCTTCCCG-CCTAGAGCATTTGCAAGTCAGGATTCTCTAATCCCTCTGGGAGAAGGGTATTCGGCTTGTCCGCTATGTTTT

>MK540385 Human gammaherpesvirus 4 isolate NPCT029

AGGACAGCCGTTGCCCTAGTGGTTTCGGACACACCGCCAACGCGCTGTGCGGTGCTGCCGTCCCGAGTTCAAGTCCCGGGGGAGGAGAAGAGCGGCTTCCCG-CCTAGAGCATTTGCAAGTCAGGATTCTCTAATCCCTCTGGGAGAAGGGTATTCGGCTTGTCCGCTATGTTTT

>MK540400 Human gammaherpesvirus 4 isolate NPCT047

AGGACAGCCGTTGCCCTAGTGGTTTCGGACACACCGCCAACGCGCTGTGCGGTGCTGCCGTCACGAGGTCAAGTCCCGGGGGAGGAGAAGAGCGGCTTCCCG-CCTAGAGCATTTGCAAGTCAGGATTCTCTAATCCCTCTGGGAGAAGGGTATTCGGCTTGTCCGCTATGTTTT

>MK540397 Human gammaherpesvirus 4 isolate NPCT043

AGGACAGCCGTTGCCCTAGTGGTTTCGGACGCACCGCCAACGCGCTGTGAGGTGCTGCCGTCCCGAGGTCAAGTCCCGGGGGAGGAGAAGAGCGGCTTCCCG-CCTAGAGCATTTGCAAGTCAGGATTCTCTAATCCCTCTGGGAGAAGGGTATTCGGCTTGTCCGCTATGTTTT

>MG298916 Human gammaherpesvirus 4 isolate RK_LCL_L5

AGGACAGCCGTTGCCCTAGCGGTTTCGGACACACCGCCAACGCGCTGTGCGGTGCTGCCGTCCCGAGGTCAAGTCCCGGGGGAGGAGAAGAGCGGCTTCCCG-CCTAGAGCAATTGAAAGTCAGGATTCTCTAATCCCTCTGGGAGAAGGGTATTCGGCTTGTCCGCTGT-TTTT

>MG298912 Human gammaherpesvirus 4 isolate RK_LCL_L2

AGGACAGCCGTTGCCCTAGCGGTTTCGGACACACCGCCAACGCGCTGTGCGGTGCTGCCGTCCCGAGGTCAAGTCCCGGGGGAGGAGAAGAGCGGCTTCCCG-CCTAGAGCAATTGAAAGTCAGGATTCTCTAATCCCTCTGGGAGAAGGGTGTTCGGCTTGTCCGCTGT-TTTT
