# Supplementary figures and images for: A Polymorphism in the Epstein-Barr Virus EBER2 Noncoding RNA Drives In Vivo Expansion of Latently Infected B Cells
Source: mBio. 2022 Jun 1;13(3):e00836-22. doi: 10.1128/mbio.00836-22 (PMC9239156; doi:10.1128/mbio.00836-22)

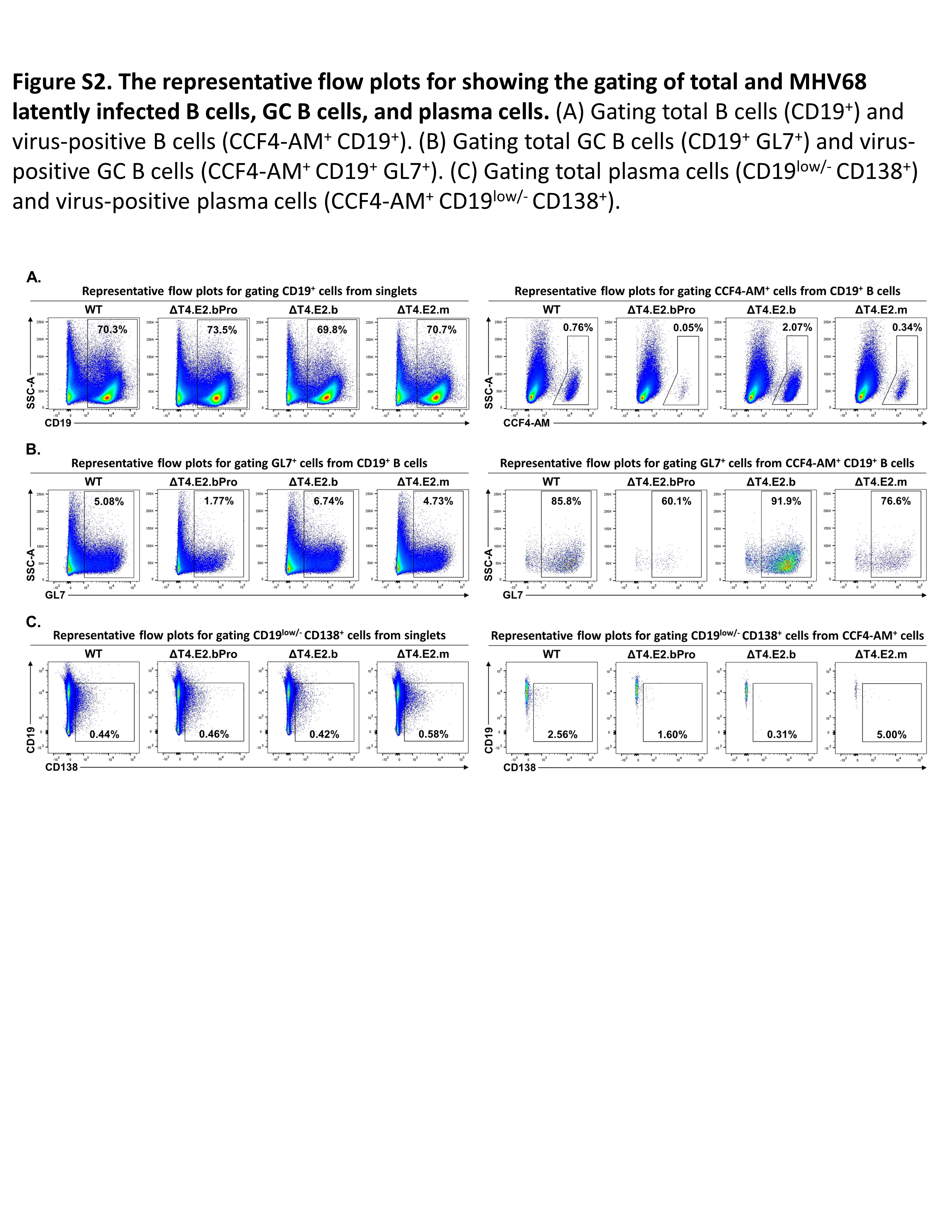

Supplement: FIG S2 [file mbio.00836-22-s0002.tif]

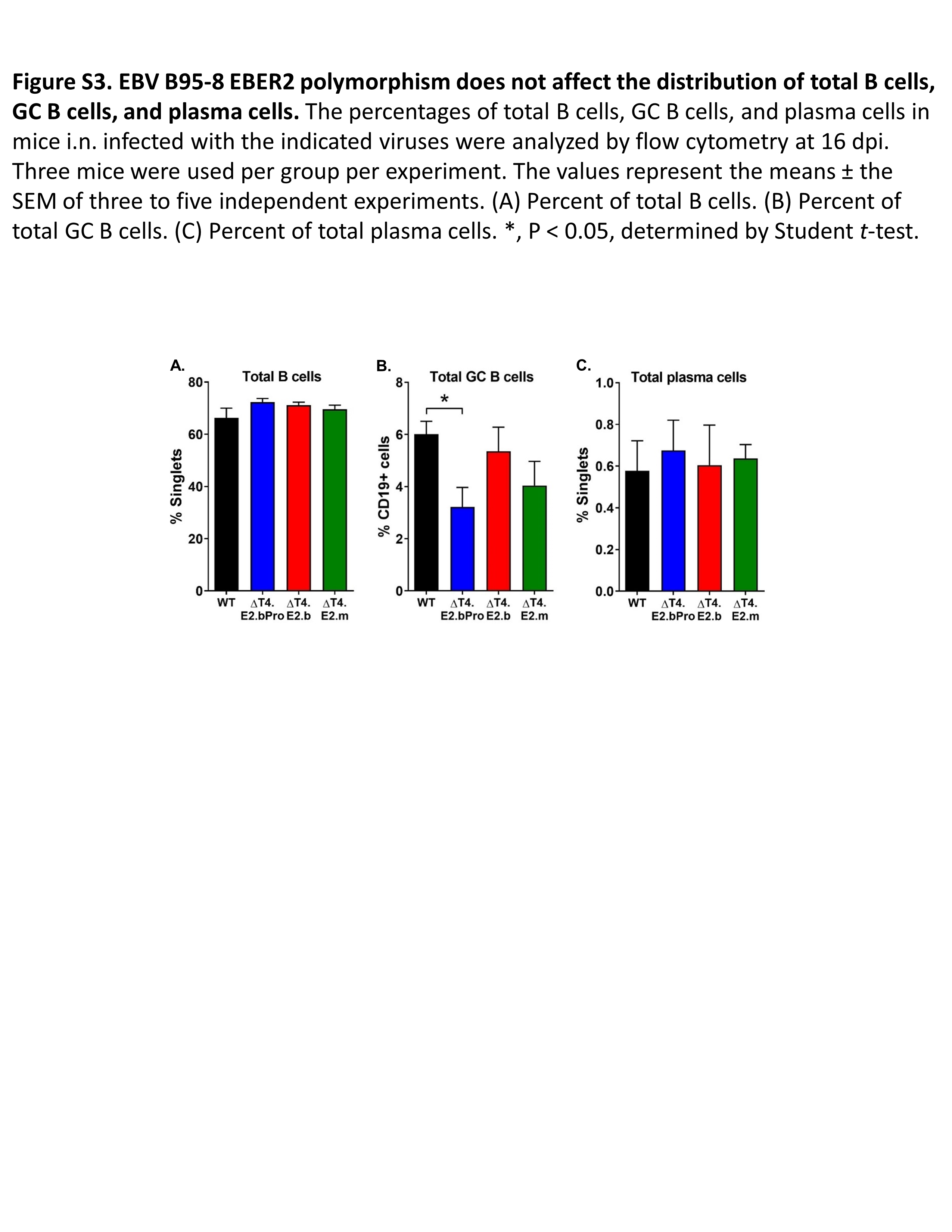

Supplement: FIG S3 [file mbio.00836-22-s0003.tif]
